# Decelerated genome evolution in modern vertebrates revealed by analysis of multiple lancelet genomes

**DOI:** 10.1038/ncomms6896

**Published:** 2014-12-19

**Authors:** Shengfeng Huang, Zelin Chen, Xinyu Yan, Ting Yu, Guangrui Huang, Qingyu Yan, Pierre Antoine Pontarotti, Hongchen Zhao, Jie Li, Ping Yang, Ruihua Wang, Rui Li, Xin Tao, Ting Deng, Yiquan Wang, Guang Li, Qiujin Zhang, Sisi Zhou, Leiming You, Shaochun Yuan, Yonggui Fu, Fenfang Wu, Meiling Dong, Shangwu Chen, Anlong Xu

**Affiliations:** 1 State Key Laboratory of Biocontrol, Guangdong Key Laboratory of Pharmaceutical Functional Genes, School of Life Sciences, Sun Yat-sen University, Guangzhou 510275, China; 2 Evolution Biologique et Modélisation UMR 7353 Aix Marseille Université/CNRS, 3 Place Victor Hugo, 13331 Marseille, France; 3 School of Life Sciences, Xiamen University, Xiamen 361005, China; 4 Shenzhen Research Institute of Xiamen University, Shenzhen 518058, China; 5 Fujian Key Laboratory of Developmental and Neuron Biology, College of Life Sciences, Fujian Normal University, Fuzhou 350108, China; 6 Beijing University of Chinese Medicine, Dong San Huang Road, Chao-yang District, Beijing 100029, China

## Abstract

Vertebrates diverged from other chordates ~500 Myr ago and experienced successful innovations and adaptations, but the genomic basis underlying vertebrate origins are not fully understood. Here we suggest, through comparison with multiple lancelet (amphioxus) genomes, that ancient vertebrates experienced high rates of protein evolution, genome rearrangement and domain shuffling and that these rates greatly slowed down after the divergence of jawed and jawless vertebrates. Compared with lancelets, modern vertebrates retain, at least relatively, less protein diversity, fewer nucleotide polymorphisms, domain combinations and conserved non-coding elements (CNE). Modern vertebrates also lost substantial transposable element (TE) diversity, whereas lancelets preserve high TE diversity that includes even the long-sought RAG transposon. Lancelets also exhibit rapid gene turnover, pervasive transcription, fastest exon shuffling in metazoans and substantial TE methylation not observed in other invertebrates. These new lancelet genome sequences provide new insights into the chordate ancestral state and the vertebrate evolution.

## Abstract (web summary)

The lancelet, or amphioxus, is an extant basal chordate that diverged from other chordate lineages about 550 million years ago. Here the authors sequence and assemble the diploid genome of a male adult of the Chinese lancelet, *B. belcheri*, and highlight genomic features that may have played an important role in the origin and evolution of vertebrates.

## Graphical abstract

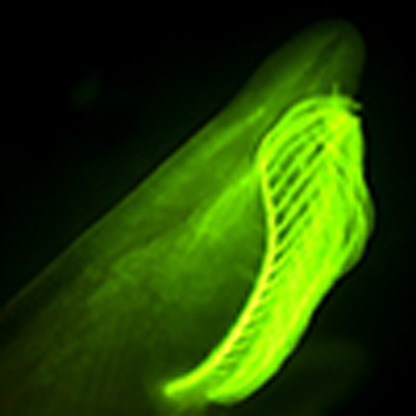

The lancelet, or amphioxus, is the extant basal chordate (cephalochordate), which diverged from other chordate lineages (urochordate and vertebrate) some 550 Myr ago and retains a body plan and morphology most similar to fossil Cambrian chordates^1^,^2^,^3^. Analyses of the genome of the Florida lancelet *Branchiostoma floridae* have shown that this chordate did not undergo the two rounds of whole-genome duplication (2R-WGD) but shares extensive genomic conservation with vertebrates^4^,^5^, emphasizing the lancelet’s role as one of the best proxies for the chordate ancestral state.

Here we sequence and assemble the diploid genome of a male adult of the Chinese lancelet *B. belcheri*, a subtropical species native to Chinese seas and a promising experimental model (Supplementary Note 1). In parallel, we generate 14 transcriptomes representing different developmental stages, tissues and immune responses and carried out whole-genome resequencing and bisulfite sequencing of five additional individuals. Combining these new data with the Florida lancelet draft genome, we re-evaluate the evolutionary rates of different genetic events within lancelets and among major chordate lineages. The new information reveals the genomic features that may have driven the origin and subsequent evolution of vertebrates.

## Results

### Two separate haploid assemblies

**Figure 1:**
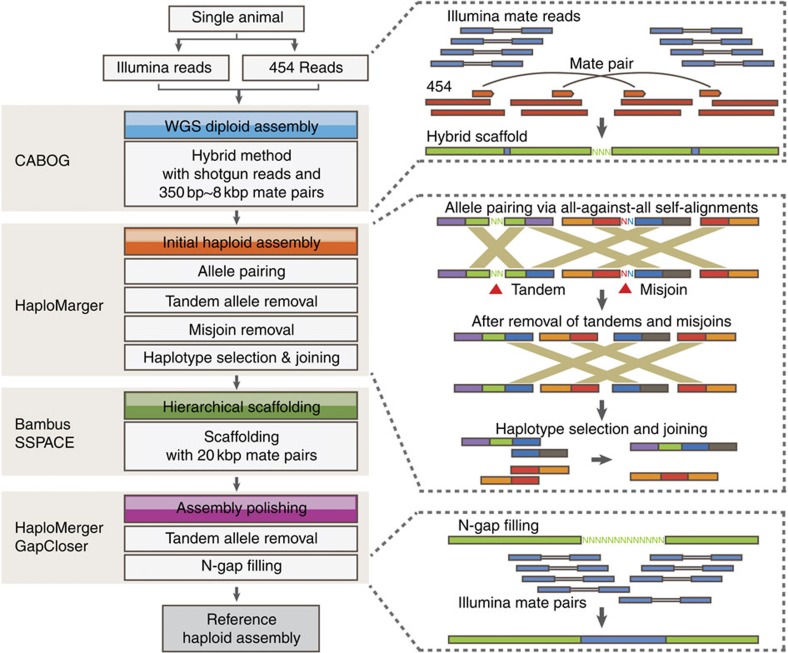
A novel whole-genome shotgun (WGS) assembly pipeline for highly polymorphic diploid genomes. The pipeline was gradually set-up to achieve optimal assembly quality through testing and combining algorithms and data sets. An upgraded version of HaploMerger^7^ was used to monitor assembly quality, to correct major assembly errors such as misjoins and tandem misassemblies and to separate and reconstruct haploid assemblies. We chose the assembler CABOG^44^ for *de novo* hybrid assembly to compensate for the short-read lengths and different sequencing error types by combining the advantages of 454 reads and Illumina reads. We conducted further hierarchical scaffolding of pre-assembled contigs using SSPACE^45^. GapCloser^46^ was employed to close N-gaps. Details of the pipeline and its development, application and assessment are described in Supplementary Note 2.

The wild Chinese lancelet exhibits a high level of polymorphism. Generating a polymorphic diploid genome is difficult using whole-genome shotgun assembly^6^, particularly when using short-read (next-generation) sequencing^7^,^8^. We reasoned that haplotypes could be better resolved using longer reads, whereas base-level errors could be rectified by a high depth of short reads. We therefore generated 30 × long 454 reads and 70 × short Illumina reads and assembled them using a novel pipeline (Fig. 1; Supplementary Table 1; Supplementary Note 2). This pipeline allowed the separation and reconstruction of two haploid assemblies: the reference assembly (426 Mb), and the alternative assembly (416 Mb) that contains alleles not included in the reference assembly. Both assemblies have a scaffold N50 size of 2.3 Mb and a contig N50 size of 46 kb (Table 1). Such separate haploid assemblies facilitate accurate allele comparison and reliable gene prediction.

### Decelerated amino-acid substitution in vertebrates

**Figure 2:**
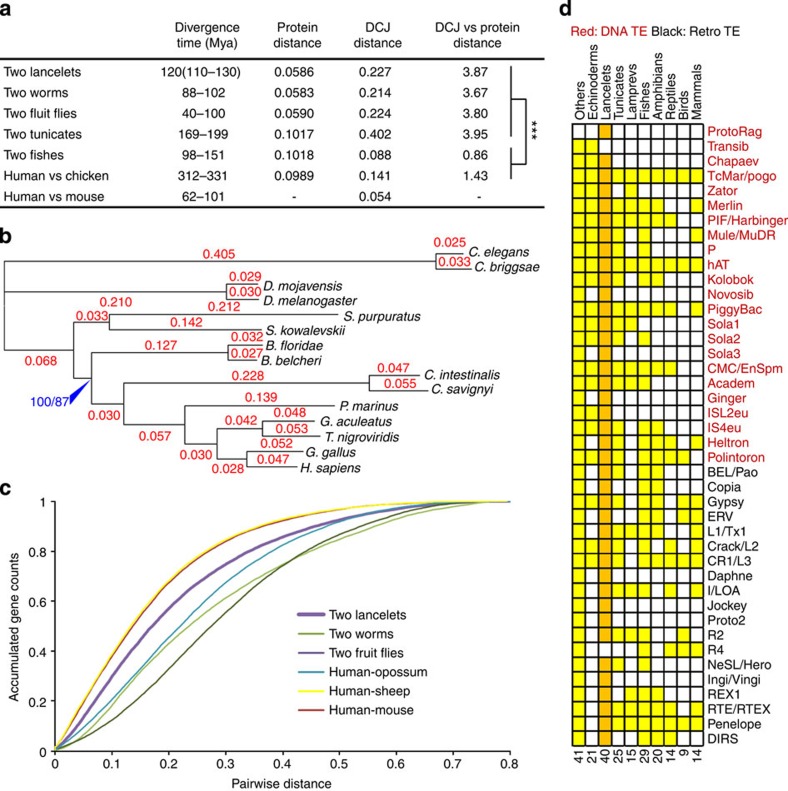
Comparative analysis of molecular divergence and TEs. (**a**) Comparison of divergence times of selected species pairs (see Supplementary Table 4 and Supplementary Note 3 for the source of divergence times), protein distances (based on the conserved amino-acid sites of 729 orthologous genes present in 15 widely divergent species), DCJ distances (based on all orthologous protein genes of the species pair) and relative DCJ distances (DCJ distance divided by protein distance). *** indicates significant difference (*P*<1e−16, *χ*^2^-test). (**b**) Maximum-likelihood (ML) phylogenetic tree containing the numbers of expected substitutions per amino-acid position, using 245,205 conserved sites from a concatenated alignment of 729 orthologous protein genes. Both Bayesian supports and ML bootstrap supports were 100% for all nodes but one, whose statistical support (Bayesian/ML) is indicated in blue colour. Supplementary Fig. 3 and Supplementary Note 3 provide details of this phylogenetic analysis. (**c**) The cumulative distribution of the pairwise protein distances of all 1:1 orthologues in the six species pairs. Note that the curve of human versus mouse largely overlaps with that of human versus sheep. The orthologous protein distance between the two lancelet species falls midway between those of human versus sheep (divergence time: 95–113 Myr) and human versus opossum (divergence time: 125–138 Myr). More information is provided in Supplementary Note 3. (**d**) Distribution of the ATE superfamilies in the major animal lineages. For lancelets, ATE families are required to be present in both Florida and Chinese lancelets; for the other lineages, TE families are required to be present in at least one species of that lineage. Data for other lineages were taken from RepBase and the literature. More information is provided in Supplementary Note 6.

We performed phylogenetic analyses on a set of 729 orthologous protein-coding genes that are present in Chinese and Florida lancelets and thirteen other divergent species (Fig. 2a,b; Supplementary Fig. 3; Supplementary Note 3). Both maximum-likelihood and Bayesian methods recovered the same deuterostome phylogeny^1^,^5^,^9^, in which lancelets represent the most basal extant chordate lineage, and echinoderms and hemichordates represent the most basal extant deuterostome lineage. Bayesian molecular dating suggests that Chinese and Florida lancelets diverged 120±10 Myr ago (Supplementary Fig. 3; Supplementary Table 4). This result agrees with the 112-Myr divergence time calculated based on lancelet mitochondrial genomes and the 100–130 Myr split time between the Atlantic and Pacific oceans^10^,^11^. Consistent with early reports^1^,^5^, lancelets show fewer amino-acid substitutions (shorter branches) than urochordates and vertebrates (Fig. 2b). However, our new data show that, with respect to the 729 proteins, lancelets evolved not only at least as rapidly as tetrapods, but also at a steady pace, in other words, the substitution rates before and after the split of two lancelet species are similar (Supplementary Table 4; Supplementary Note 3). The pairwise distances of all orthologous protein pairs in lancelets falls between those for human versus sheep (95–113 Myr divergence) and human versus opossum (125–138 Myr divergence), confirming that lancelets and tetrapods have similar rates of amino-acid substitution (Fig. 2c). In contrast, the substitution rates in vertebrates before the separation of jawed and jawless vertebrates were two to four times higher than those after the separation, indicating that amino-acid substitution was accelerated in ancient vertebrates but rapidly slowed down in modern vertebrates (Fig. 2b; Supplementary Table 4; Supplementary Note 3).

### Extreme polymorphism rate and population size of lancelets

We analysed allelic variation in the assembled diploid genome (Supplementary Figs 7–14; Supplementary Tables 5–7; Supplementary Notes 4 and 5). The polymorphism rates for SNPs and small insertions and deletions (indels; ≤300 bp, with 96.4% ≤50 bp) were 4.39 and 0.98%, respectively. The total length of the small indels accounts for 9.29% (or 4.90% for indels ≤50 bp) of the genome length. These rates are ~50 times the rates in humans and were corroborated by resequencing the data from five unrelated lancelet individuals. For large indels (300–10,000 bp), 36,859 events were identified, covering 6.51% of the genome. Approximately 65–77% of the large indels appear to result from transposable element (TE) activity. We also detected 10,190 translocations and inversions that cover 5.15% of the genome; this rate is ~30 times that for human versus chimpanzee and is the highest reported in metazoans thus far. These numbers confirm that the wild Chinese lancelet is one of the most genetically diverse animals sequenced to date.

The distribution of local polymorphism over short-length scales in the assembled genome obeys a geometric distribution, suggesting that the genome is drawn from a population with nearly random mating (Supplementary Figs 7–9). According to the neutral theory, high heterozygosity in a population may reflect a large effective population size, an increased mutation rate or both. Lancelets show the fewest amino-acid substitutions among the three chordate lineages (Fig. 2b), and hence are not likely to have accelerated mutation rates. The average synonymous substitution rate for lancelet genes was estimated to be 0.070–0.075, depending on the criteria used, and the corresponding *d*_*N*_*/d*_*S*_ ratio was 0.067–0.089, as compared with 0.07 for *Ciona savignyi*^12^, 0.15 for *Drosophila melanogaster*^13^, 0.14 for zebrafish^14^ and 0.35 for humans^15^ (Supplementary Table 7; Supplementary Notes 4 and 5). This ratio suggests that it is not relaxed selection constraints but strong natural selection (a common feature of large populations) that most likely accounts for the lancelet’s high level of heterozygosity. We estimated Chinese lancelets to have an effective population size of 1.3–13 million, depending on the mutation rate (10^−8^ to 10^−9^ per year) used for the calculation. Indeed, Chinese lancelets inhabit an area that extends over 1,200 km along the coastline of Southern China and potentially contains billions of individuals (Supplementary Fig. 1a; Supplementary Note 1). This population shows no obvious genetic structure, as revealed by comparing the mitochondrial DNA and the sequenced genomes of multiple lancelet individuals collected from distant locations over a 1000-km apart (Supplementary Fig. 1b; Supplementary Tables 8 and 9; Supplementary Notes 1 and 5).

### TE diversity lost in vertebrates but preserved in lancelets

TEs and repetitive DNA constitute >30% of the assembled genome, and we identified at least 40 known autonomous TE (ATE) superfamilies (Supplementary Table 10; Supplementary Note 6). The 40 superfamilies are present in both Chinese and Florida lancelets, but none accounts for more than 2.7% of the genome in either species. And there is no obvious bias to obviously biased to DNA transposons or retrotransposons (Supplementary Fig. 15). In contrast, jawed vertebrates have 31 ATE superfamilies and mammals have no more than 14 (Fig. 2d). In a vertebrate species, the ATE content is dominated by a few families. For example, in human, LINE1 elements comprise 17% of the genome, ERV elements account for 5% and DNA TEs represent <3% (ref. 16). These facts suggest that modern vertebrates may have lost a large degree of TE diversity. Remarkably, we discovered the RAG transposon (designated *ProtoRAG*) in the lancelet genomes. Recombination-activating genes 1 and 2 (*RAG1/2*) encode the key enzyme responsible for the somatic VDJ rearrangement of antigen receptors; therefore, their emergence is a milestone in the genesis of vertebrate adaptive immunity^17^. The origin of *RAG1/2* may be a horizontal gene transfer event from a transposon, a virus or a bacterium^18^,^19^,^20^. Our discovery of *ProtoRAG* not only substantiates the transposon-origin hypothesis that was first proposed by Tonegawa in late 1970s (ref. 21) but also highlights the extraordinary TE diversity in lancelets.

Most lancelet ATE superfamilies appear to be active (Supplementary Note 6). First, 65–77% of large polymorphic indels could be ascribed to recent TE insertions (only three ATEs had no copies in these indels). In addition, our analysis of RNA-seq data identified transcripts from 26–36 (depending on the criteria) ATE superfamilies, covering ~70% of the 2,715 retrotranscriptase and transposase fragments in the genome assembly. Genome-wide high-level DNA methylation is the major means of silencing TEs in plants and vertebrates. In urochordates and other invertebrates, however, TEs are hypomethylated, and there is little evidence that methylation inhibits TE activity^22^. Here we created base-resolution methylomes for two lancelet individuals. These data show that TEs are the second-most methylated sequences in the genomes, after protein-coding exons (discussed in the section pervasive transcription versus genome-wide methylation). Therefore, the lancelet is the first invertebrate reported to exhibit substantial TE methylation. We propose that TE methylation be considered an ancestral chordate feature that was enhanced in vertebrates but lost in urochordates. In lancelets, TE silencing by methylation may be inefficient because the methylation level is low, with only 17% of TE-related CG sites methylated at 80–100%. Nevertheless, high TE diversity and activity could provide potential benefits to lancelets over evolutionary time: a toolbox of diverse regulatory elements; the rapid generation of indels, alternative splice sites, new exons and genes; and increased rates of gene duplication, exon shuffling and gene rearrangement.

### Decelerated genome restructuring in vertebrates

**Figure 3:**
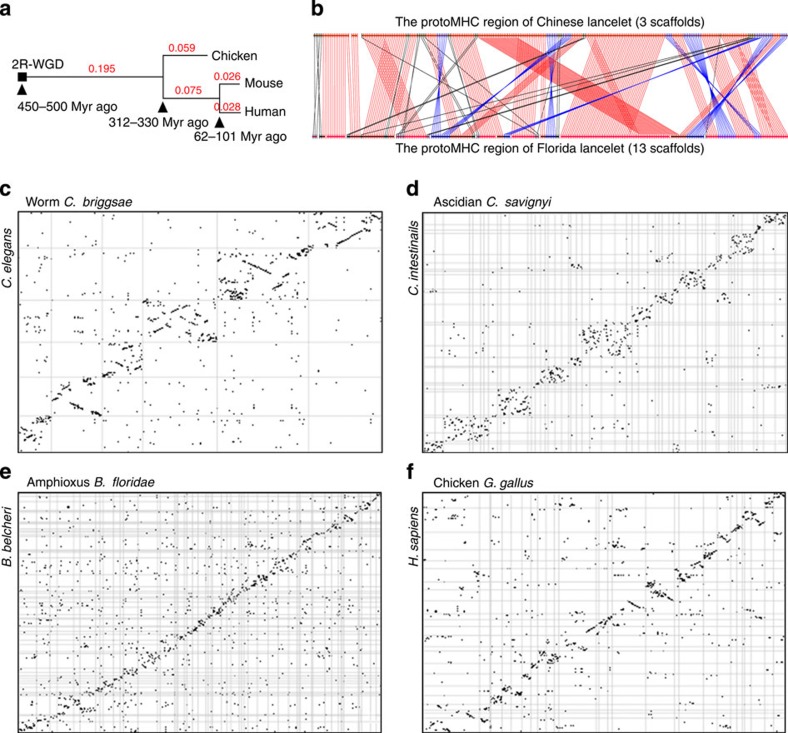
Comparative analysis of gene synteny and rearrangements. (**a**) A distance tree (DCJ distance) showing that the genome-wide gene rearrangement rates in modern vertebrates (chicken, human and mouse) sharply decreased after the 2R-WGD. (**b**) Comparison of the gene order in the protoMHC region between the Chinese and Florida lancelets. A total of 269 genes conserved between lancelet and human are shown in the analysis. The DCJ rearrangement rate between the protoMHC regions of the two lancelets is 120/269=0.45, which is almost twice the average genome-wide rate (0.23) between the two lancelets (*P*<1e−8, *χ*^2^-test), indicating highly active local gene order scrambling in the protoMHC region. (**c**–**f**) Dot plots of gene synteny and rearrangements between closely related genomes. Scaffolds and chromosomes were bidirectionally clustered according to their similarity in gene synteny conservation. Two additional species pairs (fruit flies and bony fishes) and the high-resolution figures are presented in Supplementary Figs 16–21. More information is provided in Supplementary Note 7.

We computed pairwise gene rearrangement rates for six species pairs using the ‘double cut and join’ (DCJ) distance method (Fig. 2a; Supplementary Tables 11 and 12; Supplementary Note 7). Three invertebrate pairs, lancelets, worms and fruit flies, exhibited similar relative rearrangement rates (rearrangement rate divided by protein sequence divergence; Fig. 2a). Tunicates are known for their dramatic genome restructuring, but their rearrangement rate is still in proportion to their protein evolution. Vertebrates, however, show significantly lower relative rearrangement rates than do invertebrates (as shown in the last column of Fig. 2a). This difference in rearrangement rates between vertebrates and invertebrates can be further increased to four- to eightfold if the rate is divided by the divergence time (Fig. 2a; Supplementary Note 7). Using an improved algorithm for genome aliquoting^23^, we confirmed that the rearrangement rates in vertebrates dropped sharply after the 2R-WGD (Fig. 3a; Supplementary Fig. 22; Supplementary Note 7). We visually examined the rearrangement pattern and found that vertebrates show long conserved syntenies with many gene translocations to other chromosomes, whereas lancelets and other invertebrates favour local gene order scrambling (Fig. 3b–f; Supplementary Figs 16–21).

Lancelets and vertebrates share extensive synteny conservation, allowing for the reconstruction of 17 ancestral chordate linkage groups^5^,^24^. The current explanation for this conservation is the slow evolution of lancelets^24^,^25^,^26^. Our new findings show that this conservation is instead primarily attributable to the slowed-down rearrangement rates in vertebrates and to the local gene-scrambling pattern in lancelets. Fewer rearrangement events in vertebrates could be due to low rearrangement occurrence rates or to strong functional constraints. Though the true scenario remains elusive, we speculate that a large number of gene syntenies were gradually formed and became essential for survival during the evolution of vertebrates, such that purifying selection had to act intensively against rearrangements to maintain these syntenies. On the other hand, the lancelet genome is more amenable to local gene scrambling. A prominent example is the protoMHC region^27^. Our sequence analysis recovered the complete protoMHC region in lancelets, which shares high syntenic conservation with the human MHC regions. However, the lancelet protoMHC region displays a local rearrangement rate twice that of the average genome-wide rearrangement rate (Fig. 3b; Supplementary Note 7). This new observation is consistent with the MHC ‘big bang’ hypothesis, which proposes that many novel domains and domain combinations arose in this region and contributed to the origin of adaptive immunity^27^,^28^.

### Pervasive transcription versus genome-wide methylation

**Figure 4:**
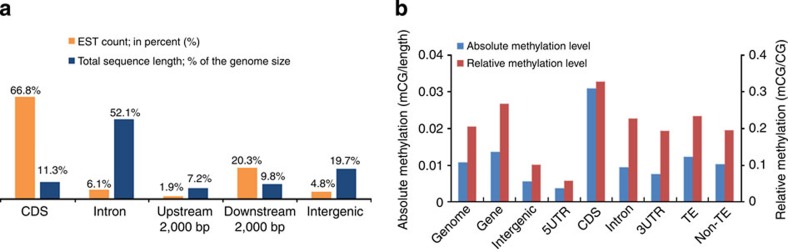
Genome-wide transcription and methylation profiles of the Chinese lancelet. (**a**) The fraction of ESTs mapped to the five genomic regions. (**b**) Methylation level of several function regions. The difference between any two function regions is highly significant (*P*<1e−16, Student’s *t*-test).

Pervasive transcription is virtually absent in fruit flies^29^ but is observed in humans, with 62% of the human genome covered by mature mRNAs^30^. However, a large amount of random transcription in humans occurs at very low levels and in non-normal tissues (for example, cell lines) with atypically low DNA methylation. Here we show that ~70% of the Chinese lancelet reference genome was covered by reads derived from 14 transcriptomes representing different development stages, tissues and immune responses (Supplementary Notes 8–10). Approximately 67, 6, 5 and 22% of ESTs mapped to coding sequences, introns, intergenic regions and the up/downstream regions of the genes, respectively (Fig. 4a; Supplementary Fig. 23). Considering our use of only 14 RNA-seq samples and the low RNA-Seq depth (~120 × ), lancelets may have an even higher level of pervasive transcription.

Extensive high-level DNA methylation is the major means of suppressing random transcription in vertebrates and plants^22^. Here we created base-resolution whole-body methylomes for two unrelated adult Chinese lancelets (Supplementary Table 14; Supplementary Note 10). A low methylation level (21%) was observed in both lancelet methylomes. Coding exons showed the highest methylation levels (33%), whereas introns (23%), sequences downstream of genes (19%), intergenic regions (10%) and sequences upstream of genes (5.8%) showed lower methylation levels (Fig. 4b). Notably, lancelet TE sequences exhibit higher methylation than do introns (Fig. 4b), which conflicts with the current knowledge that TEs are not methylated in invertebrates^22^. We suspect that the relatively low methylation level and pervasive transcription in lancelets facilitated the expression of new genes and shuffled exons, thereby increasing their exposure to natural selection.

### High proteome diversity in lancelets

**Figure 5:**
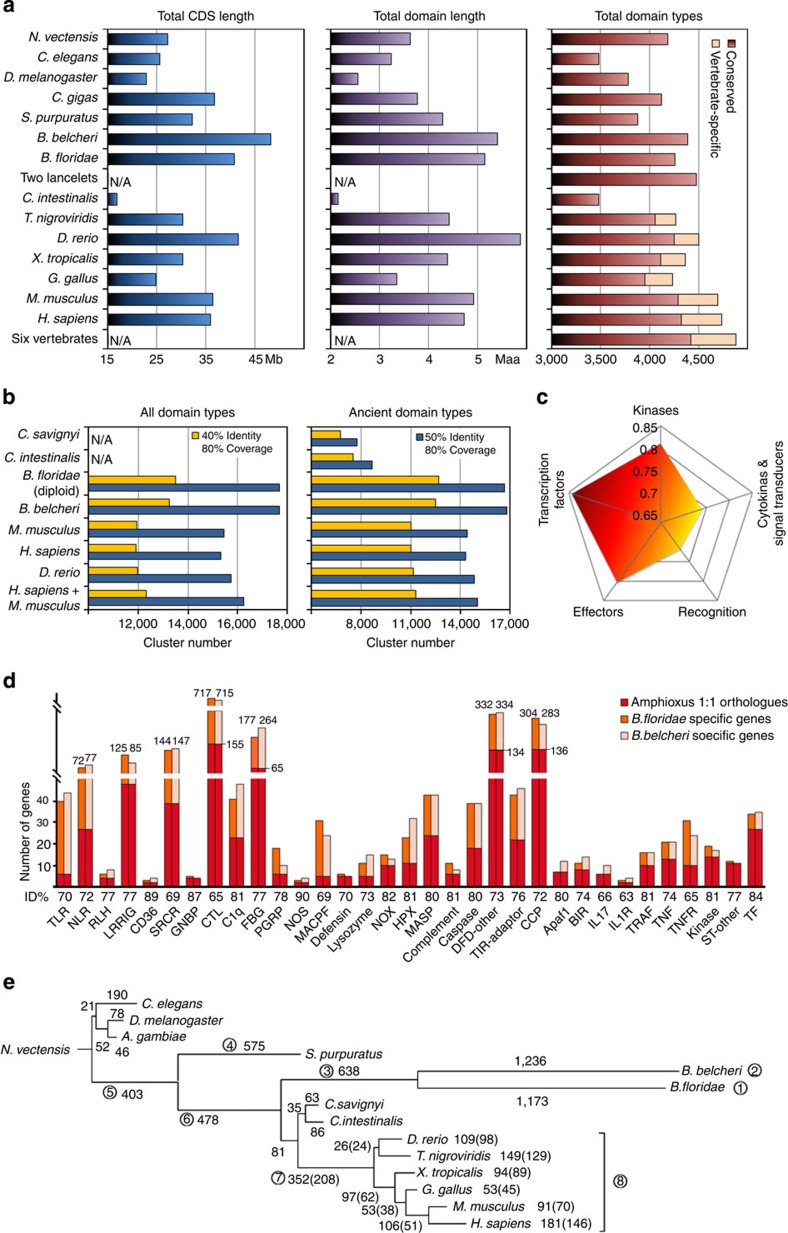
Comparative analysis of protein diversity. (**a**) Comparison of total CDS length, total Pfam-A domain length and total Pfam-A domain type numbers from the sequenced genomes of a variety of species. All known spliced isoforms were included. (**b**) Comparison of domain sequence diversity between lancelets and vertebrates. The diversity was directly measured using the numbers of sequence clusters created using BLASTCLUST. All (Pfam-A) domain types and ancient domain types (that is, non-vertebrate-specific domain types) were analysed separately. (**b**) The increasing trend of average sequence identity of proteins in five sequential phases of the immune response, from recognition to transcription factors. (**d**) The expansion and diversification pattern of the immune and stress protein gene repertoire. Average protein identity and the number of 1:1 orthologue proteins versus species-specific proteins are shown. (**e**) The number of novel domain pairs gained by different lineages. Branch length is proportional to the number of novel domain pairs. Numbers outside and within parentheses represent all novel domain pairs and the novel domain pairs containing no vertebrate-specific domains, respectively. Numbers in circles represent the eight important lineages: *B. floridae*, *B. belcheri*, amphioxus ancestor, *S. purpuratus*, deuterostome ancestor, chordate ancestor, vertebrate ancestor and all six vertebrates. More information is provided in Supplementary Notes 11 and 12.

On the basis of ~300 million EST read pairs, we predicted 30,392 protein-coding genes in the Chinese lancelet genome (Supplementary Table 13), of which 27,615 have homologues (*E*<1e−5) in other model species, and 18,167 have orthologues in the Florida lancelet (Supplementary Note 8). The mean identities of orthologous proteins and coding DNA sequences (CDS) between the two lancelet species were 81.2 and 79.5%, respectively, and there was virtually no similarity between orthologous intron sequences, suggesting that the divergence time of 100–130 Myr eliminated any similarity in the neutral sites (Supplementary Figs 4 and 5; Supplementary Note 9). The total predicted CDS size of the Chinese lancelet is 48 Mb, with 95, 92 and 86% supported by ≥1, ≥2 and ≥5 ESTs, respectively (Supplementary Fig. 23). A similar CDS volume could be detected in the Florida lancelet genome assembly (Supplementary Note 11). Therefore, lancelets appear to have a larger CDS volume than do vertebrates and other invertebrates, even when all of the known spliced isoforms were included for the comparison (Fig. 5a and Supplementary Table 15).

Using the Pfam-A domain data set, we detected domain structures in 22,927 Chinese lancelet proteins, yielding a total domain length of ~5.4 M amino acids, larger than that of any other investigated animal except the zebrafish, which is known to retain excess protein duplicates from a recent teleost-specific genome duplication (Fig. 5a and Supplementary Tables 16 and 17). We detected 4,471 ancient domain types (that is, non-vertebrate-specific domains) in the lancelet, which is a higher number than in any examined vertebrate (Fig. 5a; Supplementary Tables 16 and 17). Lancelets also preserve 144–193 (depending on criteria) ancient domains that were not found in several investigated vertebrates (Supplementary Tables 18–20; Supplementary Note 11). Because the Pfam database is biased towards vertebrates, we expect that there may be many undiscovered domain types present in lancelets and other invertebrates that are absent in vertebrates. Using a *de novo* method, we identified 941 candidate novel domains that are conserved in the two lancelets but absent in vertebrates; the 375 most confident candidates were distributed in 1,884 proteins (Supplementary Figs 30 and 31; Supplementary Note 11). We functionally verified one of the candidates, the ApeC domain (deposited in the Pfam database under accession PF16977), as a novel pattern recognition domain for bacterial peptidoglycan^31^. We also used a BLAST-clustering method to directly measure the sequence diversity of all protein domains (vertebrate-specific domains included) in humans, mice, zebrafish, tunicates and lancelets (Supplementary Note 11). Our results suggest that lancelets have the highest domain sequence diversity (Fig. 5b). These findings suggest that lancelets have higher protein diversity than many (if not all) vertebrates, which is particularly striking considering the lancelet’s compact genome size.

### Protein diversification and the immune and stress repertoire

Many gene families in the Florida lancelet displayed rapid expansion and diversification^4^. This expansion and diversification was also observed in the Chinese lancelet, but between the two lancelet species there are substantial differences in the expansion magnitude, the proportions of orthologous pairs and the protein divergence in different gene families. A notable case is the immune and stress repertoire (Fig. 5c,d; Supplementary Note 11), in which expansion comprises >1/10 lancelet proteins, nearly 10 times higher than the human counterpart^32^. This interspecies variation is not equal in all categories of proteins. For example, the protein divergence in different phases of the immune process shows a narrowing trend from extracellular spaces to nuclei, suggesting an important role for functional constraints in protein diversification (Fig. 5c). Toll-like receptor (TLR), probably the most prominent innate receptor in chordates, displays perhaps the most extreme protein turnover and diversification rate in lancelets: 85% of lancelet TLRs became species specific (having no corresponding orthologs in the other lancelet species) within 130 Myr. In sharp contrast, most vertebrates have one orthologue of each vertebrate TLR lineage, despite the vertebrate divergence time of ~450 Myr. Other lancelet receptors with evolutionary patterns similar to lancelet TLRs include NLR, SRCR, CTL, FBG and other LRR genes (Fig. 5d; Supplementary Note 11).

### High domain recombination in lancelets but not vertebrates

We created phylogenetic trees using the presence–absence status of domain combinations in various species. All Pfam-A domains, including vertebrate-specific domains, were considered in this analysis. The trees revealed higher domain combination turnover rates in the deuterostome lineage, suggesting that new domain combinations may have been a driving force in the speciation and organismal complexity of deuterostomes (Supplementary Figs 33 and 34; Supplementary Note 12). This became more evident when we counted the domain combinations gained on each branch of the speciation tree. Similar to the patterns in the evolution of protein and genome architecture (Figs 2 and 3), the rates of gaining new domain combinations were elevated during early vertebrate evolution (branch 5, 6 and 7) but reduced in jawed vertebrates (branch 8; Fig. 5e; Supplementary Fig. 35). In contrast to vertebrates, lancelets evolved rapidly and continuously, ultimately acquiring threefold more domain combinations than any vertebrate (Fig. 5e; Supplementary Table 21). We estimate that lancelets gained new domain pairs (that is, two-domain combinations) at a rate of >10 per Myr, which is 10- to 100-fold higher than that normally observed in metazoans (0.1~1 per Myr (ref. 33)). Lancelets also appear to lose domain pairs as quickly as they gain them (Supplementary Note 12).

A common set of domains is frequently present in novel domain pairs on major deuterostome branches (Supplementary Table 22). Early reports called these domains as promiscuous domains^34^,^35^. In lancelets, an analysis of the immune-related domains indicates that domain-pair formation is biased towards certain promiscuous domains, and that natural selection plays an important role in shaping the repertoire of domain combinations (Supplementary Figs 36 and 37; Supplementary Note 12). We observed that immunoglobulin (Ig) domains are not only the most promiscuous domains in vertebrates, but also the only domains frequently used by all major deuterostome branches (Supplementary Fig. 37). This may provide an evolutionary explanation for the widespread presence of Ig domains in vertebrate biology (discussed below; Supplementary Note 11). In metazoans, promiscuous domains are enriched in the signal transduction pathways and the extracellular matrix^35^,^36^,^37^. We observed that promiscuous domains in lancelets have stronger preferences for receptor activity, signal transduction, catalytic activity and the extracellular matrix compared with those used in other metazoans (Supplementary Figs 38 and 39). Normally, domain promiscuity is a volatile, rapidly changing feature that is not conserved in different lineages^35^. Lancelets exhibit a usage pattern similar to that of the deuterostome and chordate ancestors, while jawed vertebrates display a different pattern (Supplementary Tables 22 and 23). We suggest that the rapid generation of new domain pairs could be an ancestral feature of chordates that has been conserved in lancelets but lost in jawed vertebrates.

### Extreme exon shuffling, expansion and phase bias in lancelets

**Figure 6:**
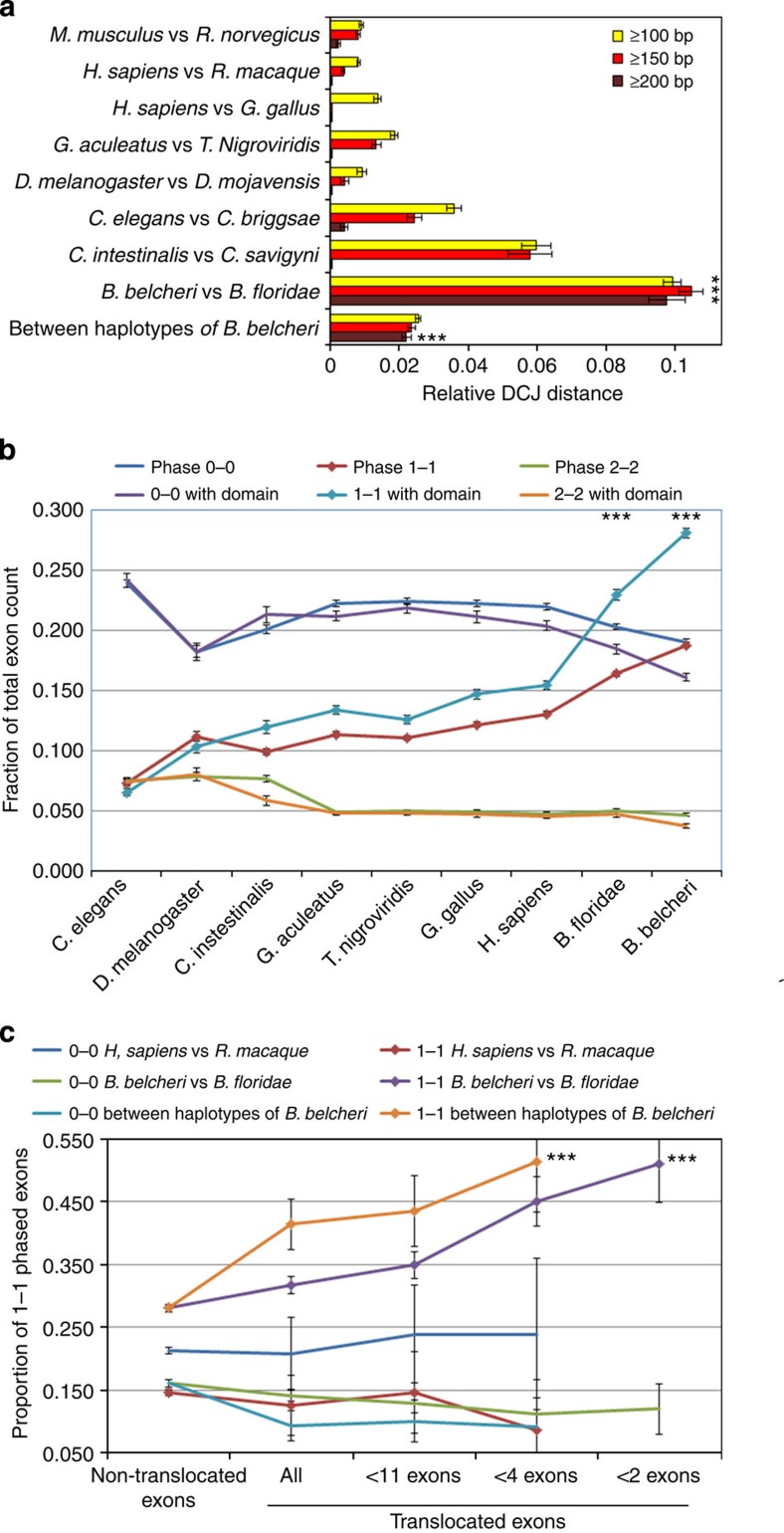
Comparative analysis of exon shuffling and exon phase bias. (**a**) Comparison of the DCJ distances contributed solely by rearrangements occurring at the subgenic (exon) level in several species pairs. *** indicates significant differences between lancelets and other species pairs (*P*<1e−16, *χ*^2^-test). (**b**) Comparison of the proportions of internal exon phases 0–0, 1–1 and 2–2 in different species. Only data for exons larger than 100 bp are shown. For all comparisons of 1–1 phased exons between lancelets and other species, *P*<1e−16 (***; *χ*^2^-tests). (**c**) Comparison of the exon phase biases of non-translocated and translocated domain-containing exons. For all comparisons between non-translocated and translocated 1–1 phased exons in lancelets, *P*<1e−16 (***; *χ*^2^-tests). The error bars show the 95% confidence intervals. More information is provided in Supplementary Note 13.

Subgenic rearrangements produce exon shuffling and may lead to new domain combinations. We discovered thousands of coding exon (that is, CDS) rearrangements between the two lancelet species, a frequency that is 2- to 100-fold (depending on the criteria) higher than that observed in vertebrates, urochordates (known for drastic genome rearrangement) and other investigated animals (Fig. 6a; Supplementary Table 24; Supplementary Note 13). High rates were also detected between the haploid genome assemblies of the Chinese lancelet. This situation is in contrast with the gene-level rearrangement pattern (Figs 2a and 3). An explanation is that the subgenic rearrangements are under a different selection regime than gene rearrangements, possibly because subgenic sequences lack the independent function and regulatory signals as are present in complete genes.

Exon shuffling and expansion in metazoans favours symmetrical phases, especially the 1–1 phase combination^38^,^39^. Here we showed that the internal exons of lancelets display a higher proportion of 1–1 phase combinations than other examined species. This proportion is even higher for exons encoding known protein domains (Fig. 6b; Supplementary Fig. 41; Supplementary Note 13). Because there is no reason to assume that the mechanisms of exon shuffling and expanding favour domain exons, the higher 1–1 phase bias of domain exons may be the result of natural selection, as domain exons are easier to adapt to new functions. We observed that the most abundant domain types encoded in 1–1 phased exons are conserved between lancelets and humans, and the promiscuous domains involved in novel domain combinations were preferentially disseminated via the 1–1 phase exons (Supplementary Tables 22 and 25–26). For example, the unprecedented expansion of Ig domains in both vertebrates and lancelets occurred almost entirely through the 1–1 phased exons (Supplementary Table 25; Supplementary Note 13). This result can also explain the widespread presence of Ig domains in vertebrate biology.

We identified and examined individual shuffled exons in lancelets using a conservative method (Supplementary Note 13). Between the two lancelet species, 40% of shuffled exons and 51% of shuffled domain exons are biased to the 1–1 phase combination, which is higher than the overall phase bias (~28%) in non-shuffled exons. This phase bias is even higher in exons shuffled between the haploid genome assemblies of Chinese lancelet (Fig. 6c and Supplementary Tables 27 and 28). In contrast, there is no 1–1 phase bias in exons shuffled between human and rhesus (Fig. 6c), suggesting that the identified exons were false positives or that the exon shuffling pattern was altered in the primate lineage. Moreover, the shuffled exons in lancelets preferentially encode the promiscuous domains used in novel domain combinations (Supplementary Tables 22, 25 and 29). Finally, high TE diversity and activity in lancelets may have played a role in exon shuffling, because there is an enrichment of transposase (12%) and retrotranscriptase (16%) fragments in lancelet translocation regions, which is 10- to 30-fold higher than the corresponding enrichment in the translocation regions of rhesus versus human (Supplementary Table 30). Our data suggest that lancelets exhibit an active exon shuffling process that is typically biased towards 1–1 phased exons (an ancient feature of metazoans^38^,^39^) and has made an essential contribution to their novel domain combination repertoire.

### High CNE diversity in lancelets

Using a pairwise genome alignment method, we identified abundant CNEs in the lancelet genomes (10.6–14.8% depending on criteria), whereas the same method revealed lower fractions of CNEs in *C. elegans* (3.0–5.2%), *D. melanogaster* (4.0–6.2%) and human (1.5–3.4%; Supplementary Fig. 43; Table 2; Supplementary Tables 31–32; Supplementary Note 14). Notably, the total CNE length is higher between the two lancelets (45.4 Mb) than between human and opossum (33.5 Mb), despite the similar divergence time of the two species pairs. Anyway, our method recovered 96% of the known lancelet microRNA genes (Supplementary Table 33). The top 30 CNE-enriched regions in lancelets cover 3% (1040) of protein-coding gene models, 5% (22.5 Mb) of the genome length and 16% of CNEs (18,697; Supplementary Table 34). Notably, the fourth highest CNE-enriched region contains the entire HOX gene cluster. We identified 1,086 (>45 bp) or 3,553 (>30 bp) CNEs that are highly conserved among lancelets and humans and opossums—three to 10 times higher than previously reported for the lancelet and mouse^40^. The enrichment of these CNEs was enhanced in the vicinity of protein-coding genes for adhesion, signalling, development, regulation and cellular component organization or biogenesis, similar to the situation in humans (Supplementary Table 35 and Supplementary Note 14).

## Discussion

Lancelets have been shown to share extensive genomic conservation with vertebrates^4^,^5^. Here we further reveal that lancelets exhibit a gene rearrangement rate and pattern similar to other invertebrates, a steady amino-acid substitution rate not slower than in modern vertebrates, and the highest rates of exon shuffling and domain combination acquisition known so far in metazoans. In addition, lancelets have an enormous population size, a highly polymorphic genome, vast TE diversity, abundant CNE content, active gene expansion, pervasive transcription and substantial TE methylation. Since these lancelet genomic features could be observed in outgroup lineages and/or in the stem of vertebrate lineage according to our phylogenomic analyses, we suspect that many of these features might represent the ancestral chordate states.

The observed faster genome evolution in the early history of vertebrates could be caused by elevated mutation rates, or fast adaptation, or relaxed purifying selection, or any combination of these mechanisms. It is not known what evolutionary event triggered these mechanisms in early vertebrates, but in theory, a genomic shock may be suffice^41^. Both genome duplications and erratic transposon activities can be drastic responses to genomic shocks. Interestingly, early vertebrates underwent both 2R-WGD and the domestication of the RAG transposon.

Here we show that compared with the closely related lancelet species, modern vertebrates have (at least relatively) lower genome diversity with respect to nucleotide polymorphisms, protein number and diversity, protein domain types, domain combinations, TE superfamilies and even CNE content. Several evolutionary mechanisms that may increase the genetic diversity were also suppressed in modern vertebrates, including effective population sizes, genome rearrangements, exon shuffling, pervasive transcription and diverse TE activity. It is therefore remarkable that modern vertebrates are still successful at adapting and diversifying. Other new mechanisms may compensate for the lost genome diversity in modern vertebrates. For example, despite having a small innate gene repertoire, vertebrates produce adaptive immune receptors that are capable of somatic diversification. Besides, it is believed that the vertebrate 2R-WGD could increase morphological complexity by instantly creating many spare modules for gene regulatory networks^42^,^43^. Finally, we expect that lancelets and their genome sequences will continue to provide new insights into the origins and evolution of vertebrates.

## Methods

### Genome sequence and assembly

The sequenced animal is a single outbred male adult of the Chinese lancelet *Branchiostoma belcheri* collected from Xiamen bay, China. Over 100 × raw shotgun and paired-end reads were generated using both the 454 FLX titanium platform (~30 × , including shotgun libraries and 2–20-kb paired-end libraries) and the Illumina GAIIx platform (~70 × , including 340–600-bp paired-end libraries). The *de novo* hybrid assembly of all reads was created using the Celera assembler^44^. hierarchical scaffolding with 20-kb mater-pair reads was conducted using HaploMerger^7^ and SSPACE^45^. The separation of two haploid assemblies was performed using HaploMerger^7^. The N-gaps in the assemblies were filled in a conservative way using GapCloser^46^.

### Whole-genome resequencing and alignment

Additional adult Chinese lancelets, two from Xiamen and three from Zhangjiang (Supplementary Fig. 1), were sequenced to over 60 × using the Ilumina Hiseq2000/2500 platform and then subjected to *de novo* assembly using the Celera assembler^44^. A multiple whole-genome alignment for these resequenced assemblies and the reference assembly was created using the LASTZ-chainNet-TBA pipeline^47^,^48^. The alignment was further refined using MUSCLE^49^.

### Whole-genome bisulfite sequencing and analysis

The two resequenced lancelets from Xiamen were also subjected to whole-genome sodium bisulfite (BS) sequencing using the Illumina Hiseq2000 platform. Over 30 × BS reads were obtained for each individual, and these BS reads were mapped to its own individual *de novo* genome assembly using GSNAP^50^. The methylated cytosines were called using the default procedure of Bis-SNP^51^ and then projected to the reference genome by consulting the whole-genome alignment between the individual assembly and the reference assembly.

### Repeat analysis

Both homology-based and *de novo* prediction analyses were used to identify the repeat content in both the Chinese lancelet genome and the Florida lancelet genome. The homology-based search was performed using RepeatMasker^52^ (the RMBlast engine) and the repeat library of *B. floridae* from the JGI website ( http://genome.jgi-psf.org/Brafl1/Brafl1.download.ftp.html) and the RepBase library version 20130422. The *de novo* prediction was carried out using both RepeatModeler ( http://www.repeatmasker.org/RepeatModeler.html) and REPET^53^. All repeats and TE families were subjected to both automated curation and manual inspection. The curated repetitive and TE sequences were used to annotate and mask the genome sequences by using RepeatMasker^52^. For comparison, window-based genome masking was also performed using WinMasker^54^.

### RNA-seq

Transcriptomes from multiple Chinese lancelets representing different developmental stages, tissues were sequenced to a total of ~120 × using both the Illumina GAIIx platform and the 454 platform. The *de novo* transcript assemblies were created using Newbler and Trinity^55^. All reads were mapped to the reference genome using GMAP/GSNAP^50^ to accommodate high polymorphism. Genome-based transcript assemblies were created from mapped reads using Cufflinks^56^.

### Gene prediction and functional annotation

Protein gene models were obtained by integrating the results of *de novo* gene prediction, homology-based and transcriptome-based prediction. Multiple prediction sets, including cDNA alignments by PASA^57^, protein alignments by GeneWise^58^, RNA-seq alignments by Cufflinks^56^, *ab initio* data sets from Augustus^59^ and GlimmerHMM^60^ and RNA-seq-based predictions by Augustus^59^, were combined into a non-redundant gene set using EVidenceModeler^57^. The initial combined prediction set was fed to Augustus^59^ for a new round of evidence-based prediction for alternatively spliced isoforms. Proteins were annotated by sesearching against the InterPro database^61^, the Pfam domain database^62^, the gene ontology database^63^ and the KEGG database^64^.

### Polymorphism and population structure

SNPs, indels and translocations were called based on the refined whole-genome alignments between haploid assemblies and individual assemblies using customed Perl scripts. Synonymous versus non-synonymous polymorphism rates, nature selection and population structure were analysed using PAML^65^ and MEGA^66^. Amplified mitochordial sequence fragments from lancelet populations were analysed using MEGA^66^.

### Divergence and phylogenetic analysis

Sequence divergence analysis was based on gene orthologues. Putative orthologous gene families were identified from all-against-all protein similarities using BLASTP^67^ and a modified reciprocal best hit (RBH) method. Twenty-five species were analysed, including the Chinese and Florida lancelets, *Nematostella vectensis*, *Caenorhabditis elegans*, *Caenorhabditis briggsae*, *Drosophila melanogaster*, *Drosophila mojavensis*, *Crassostrea gigas*, *Strongylocentrotus purpuratus*, *Saccoglossus kowalevskii*, *Ciona savignyi*, *Ciona instestinalis*, *Perkinsus marinus*, tetraodon, stickleback, zebrafish, *Xenopus tropicalis,* chicken, opossum, mouse, rat, sheep, *Rhesus macaque* and human. Multiple protein and DNA alignments were created using CLUSTALW. A concatenated protein alignment of 729 orthologue families from 15 species was created for phylogenetic reconstruction and molecular dating. Gblocks^68^ was used to remove the less-conserved sites. Bayesian and maximum-likelihood analyses were used using Phylobayes^69^ and PhyML^70^, respectively. Molecular dating was carried out using both Phylobayes and PhyML (Phytime).

### Proteome diversity and domain combinations

Protein sets from up to 25 species (aforementioned) were analysed by sesearching against the Pfam database^62^ (both Pfam-A and Pfam-B). All protein isoforms of a gene were used for analysis. Different cutoff criteria (E-value and alignment coverage) were used for comparison. The Pfam database is biased towards the vertebrates (particularly mammals); hence, we separated the vertebrate-specific domain types from those ancient protein domain types that are present in non-lancelet invertebrates. We also performed a direct comparison of domain diversity between human, mouse, zebrafish, ascidians and lancelet using a Blastclust-based method. Using the same Blastclust-based method, we carried out *de novo* novel domain identification from between the Chinese and Florida lancelets.

### Gene rearrangement

Gene rearrangement analysis was based on the putative orthologous gene families identified using a BLASTP-based, modified RBH method. For a gene with multiple protein variants, all variants were subjected to BLASTP^67^ but only the best hit among all variants was selected to represent the gene. Segments of alignments between the two genes were concatenated, and the cutoff criteria were set to 60% identity and 40% coverage. For synteny analysis, because the draft genomes of *B. belcheri* and *B. floridae* are only available at the scaffold level, we used the dissimilarity criteria (defined as −log(*P*), where *P* is the *P* value of Fisher’s exact test for the pair of scaffolds) to cluster the scaffolds bidirectionally and hierarchically. DCJ distance was used to measure the gene rearrangement rates between genomes, as was implemented in AliquotG^23^.

### Exon shuffling

The rates of exon shuffling were treated as the rates of exon rearrangement and evaluated in the same way as gene arrangement. Shuffled exons were identified using both the RBH method and the t:whole-genome chainNet method. The results of the two methods were compared with each other. Unlike the RBH method, which intends to find the best hit between individual exon sequences, the whole-genome chainNet method takes into account both non-exon sequences and syntenic information. Hence, the chainNet method generally reports fewer but higher-confidence rearrangements. In addition, the chainNet method is not affected by errors in gene and domain annotations that can occur in draft genomes.

### Conserved non-coding elements

CNE were identified between Chinese and Florida lancelets, human versus mouse, human versus opossum, the worms *C. elegans* versus *C. briggsae* and the insects *D. melanogaster* versus *D. mojavensis*. A reciprocal-best whole-genome alignment method (that is, the aforementioned LASTZ-chainNet method^47^) was used to identify CNEs between two genome sequences. Only *cis*-regulatory elements, microRNAs and long non-coding RNAs were retained in the CNE data sets, whereas all the other entities such as coding regions, pseudogenes, TEs and other RNA genes were filtered.

### Software and data

Genome data of the Chinese lancelet, including reference and alternative assemblies, annotations, proteins, transcripts and reconstructed TE sequences, are accessible on our website: http://mosas.sysu.edu.cn/genome/download_data.php. The newest version of the HaploMerger^7^ and AliquotG^23^ software can be downloaded from our website: http://mosas.sysu.edu.cn/genome/download_softwares.php.

## Author contributions

Y.W., G.L., G.H., X.Y. and S.H. prepared the genomic DNA and created the BAC libraries. S.H., Q.Y., Y.F., X.Y., Y.W. and G.L. coordinated and conducted the sequencing. S.H. developed the genome assembly pipeline. S.H. and J.L. produced the assemblies. Z.C. and S.H. developed AliquotG and performed the gene rearrangement analyses. Z.C. and S.H. performed the phylogenetic analyses. S.H. and T.Y. conducted the transposon element analysis and transcriptome analysis. T.Y. and S.H. performed the genome annotation. S.H. produced the genome alignments and analysed the protein diversity, domain combinations, subgenic rearrangements, exon shuffling, conserved non-coding elements and methylomes. S.Z. and J.L. sequenced and analysed the BAC-end reads. T.D. cloned and analysed the mitochondrial sequences from multiple lancelet populations. X.Y., T.Y., G.H., H.Z., P.Y., R.W., X.T., R.L., S.Y., F.W., M.D., S.H. and S.C. were involved in gene and gene family annotation. G.H., R.L. and S.Y. performed the functional validation. P.P., Y.W., G.L., Q.Z., L.Y. and S.Y. reviewed and edited the manuscript. L.Y. provided the art support. A.X. and S.H. conceived the study, coordinated the work and drafted the manuscript.

## Additional information

**How to cite this article:** Huang, S. *et al.* Decelerated genome evolution in modern vertebrates revealed by analysis of multiple lancelet genomes. *Nat. Commun.* 5:5896 doi: 10.1038/ncomms6896 (2014).

**Accession codes:** All sequence data from the Chinese lancelet genome project have been deposited in GenBank/EMBL/DDBJ under accession code PRJNA214454. RNA-seq data have been deposited in GenBank/EMBL/DDBJ under the accession codes SRX344152 to SRX344156. Resequenced data have been deposited in GenBank/EMBL/DDBJ under the accession codes SRX475053 to SRX475057. Bisulfite sequencing data have been deposited in GenBank/EMBL/DDBJ under the accession codes SRX475059 and SRX475060.

## Supplementary Material

Supplementary Information Supplementary Figures 1-43, Supplementary Tables 1-35, Supplementary Notes 1-14, Supplementary References

## Figures and Tables

**Table 1:** Assembly statistics*.

**Version**	**v7**†	**v15**†	**v18**†	
*Diploid*				
Span (Mb)	708	702	707	
Scaffold N50 (kb)	232	150	264	
Contig N50 (kb)	73	16	30	
				
			**Reference**	**Alternative**
*Haploid*				
Span (Mb)	416‡	451‡	426‡	417‡
Scaffold N50 (kb)	834	1,497	2,326	2,395
Contig N50 (kb)	104	25	46	46
N-gap size (%)	1.06	2.70	1.30	5.50
Misjoins§	<189	<300	<66	<66

^*^More information is provided in Supplementary Table 2.

^†^Assemblies were created using 30 × 454 reads and 70 × Illumina reads. The three assembly versions illustrate the major improvement of the assembly strategy.

^‡^The ssembly spans are close to the haploid genome size (442 Mb) estimated by cytometry analysis and k-mer counting.

^§^Potential misjoins (>100 kb) estimated by genome alignments (Supplementary Table 3).

**Table 2:** Total length of refined CNE candidates in five species pairs.

	***B. belcheri*** **(versus** ***B. floridae*****)**	***C. elegans*** **(versus** ***C. briggsae*****)**	***D. melanogaster*** **(versus** ***D. mojavensis*****)**	**human (versus mouse)**	**human (versus opossum)**
Genome size	426,108,443	100,286,070	168,736,537	3,101,788,170	3,101,788,170
Coarse CNE length	45,440,901	3,027,725	6,670,794	106,174,711*	33,471,985†
<75 bp	6,782,290	1,375,417	3,432,906	12,433,719	4,006,304
Adjacent to CDS	6,179,707	248,689	83,839	6,956,979	1,675,110
With blast hit‡	2,337,567§	12,073	28,716	755,567	247,049
Refined CNE length∥	30,003,722	1,353,843	2,839,649	85,319,227	27,436,584
Refined CNE length (%)	7.04	1.35	1.68	2.75	0.88
Refined CNE count	135,046	9,763	25,211	369,079	124,195
Average length	222.2	138.7	112.6	231.2	220.9

CDS, coding DNA sequences; CNE, conserved non-coding elements. Sequence length is shown in base pairs (bp). More details are shown in Supplementary Tables 31–33.

^*^If all protein-coding exons are removed, this value decreases to 96,465,841 bp (~9.7 Mb smaller).

^†^If all protein-coding exons are removed, this value decreases to 29,744,189 bp (~3.7 Mb smaller).

^‡^CNEs with clear blast hits (1e-5) to known proteins, tRNAs, rRNAs and so on.

^§^Protein hits accounted for 2,272,249 bp.

^∥^CNE candidates with <70% identity, <75 bp long, adjacent to CDS or homologous to known proteins/ tRNAs/rRNAs/snoRNAs/scRNAs/snlRNAs were removed.
