# Supplementary material for: Decelerated genome evolution in modern vertebrates revealed by analysis of multiple lancelet genomes
Source: Nat Commun. 2014 Dec 19;5:5896. doi: 10.1038/ncomms6896 (PMC4284660; doi:10.1038/ncomms6896)
Supplement: Supplementary Information — Supplementary Figures 1-43, Supplementary Tables 1-35, Supplementary Notes 1-14, Supplementary References [file ncomms6896-s1.pdf]

Supplementary figures

Supplementary Figure 1. Distribution of the Chinese lancelet populations

A) Amphioxus is distributed along the Chinese coastal line. Three typical habitats are Qingdao, Xiamen and Beihai. *Branchiostma japonicum* is mainly distributed from Qingdao to Xiamen; *B. belcheri* mainly occupies the area from Xiamen to Beihai. The distribution of foreign species *B. malayanum* is occasionally found in the seashore of southern China, such as Hongkong.

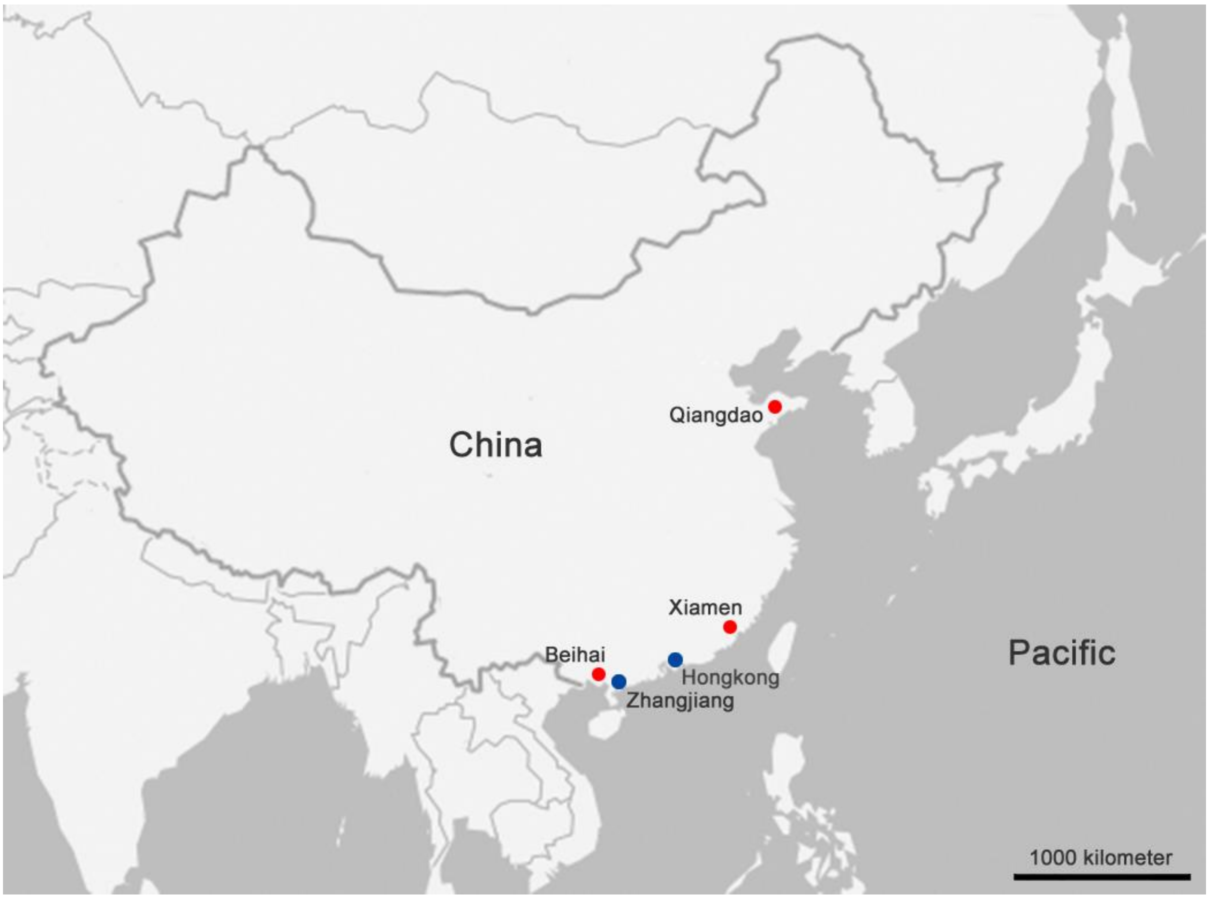

B) The Neighbor-Joining tree for amplified mitochordial sequence fragments of sampled lancelet individuals (BjQD=yellow, *B. japonicum* from Qingdao; BjXM=blue, *B. japonicum* from Xiamen; BbXM=purple, *B. belcheri* from Xiamen; BbZJ=green, *B. belcheri* from Zhangjiang; BbBH=red, *B. belcheri* from Beihai; BlFRA=grey, *B. lanceolatum*).

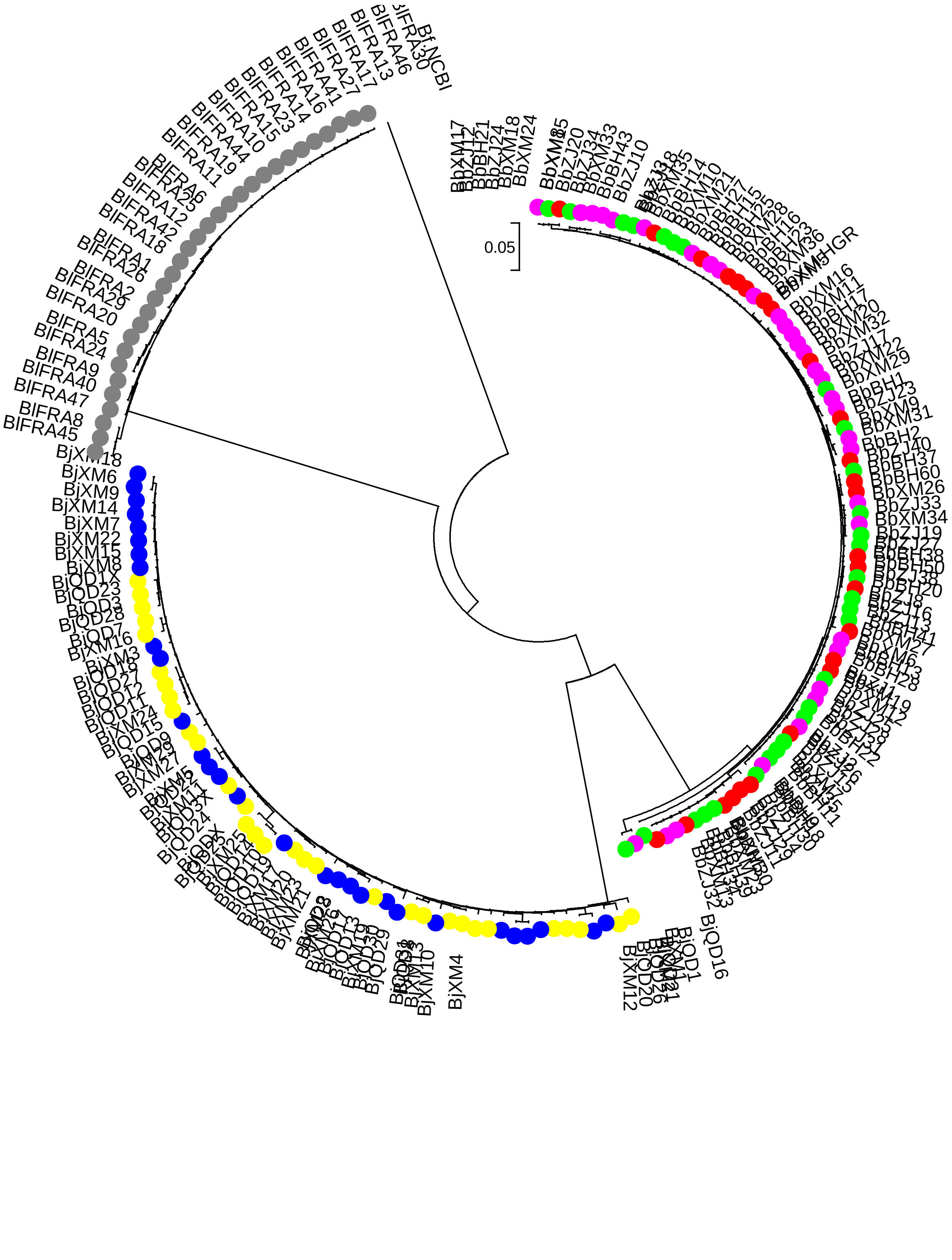

Supplementary Figure 2. The assembly pipeline

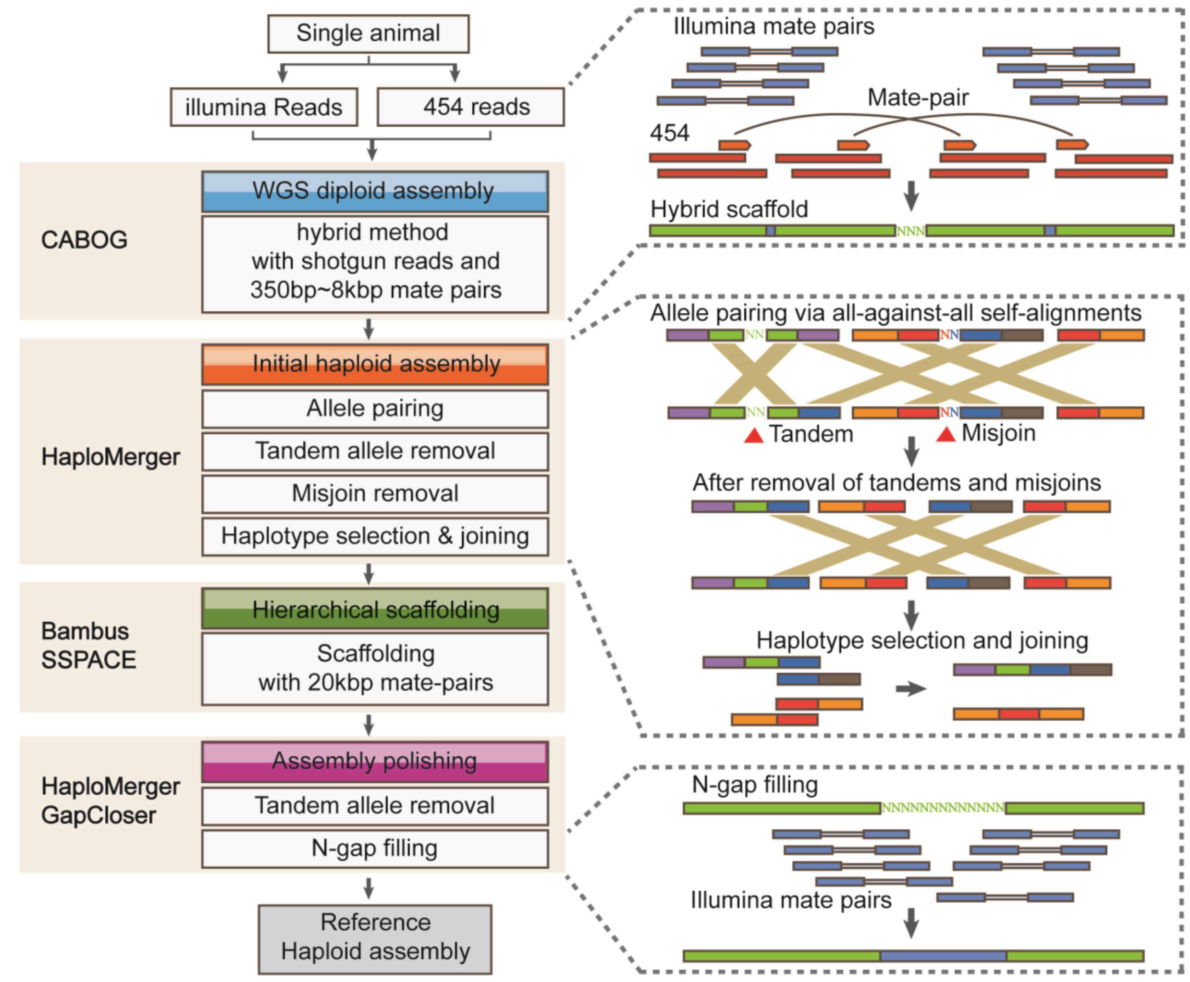

Supplementary Figure 3. Protein-based phylogenetic analysis of two lancelet species

A. Bayesian phylogenetic analysis of alignment 3 (containing 513 genes and 72,795 indel-free sites). Statistical supports and branch length are shown in blue and red color respectively. See Supplementary Note 3 for details.

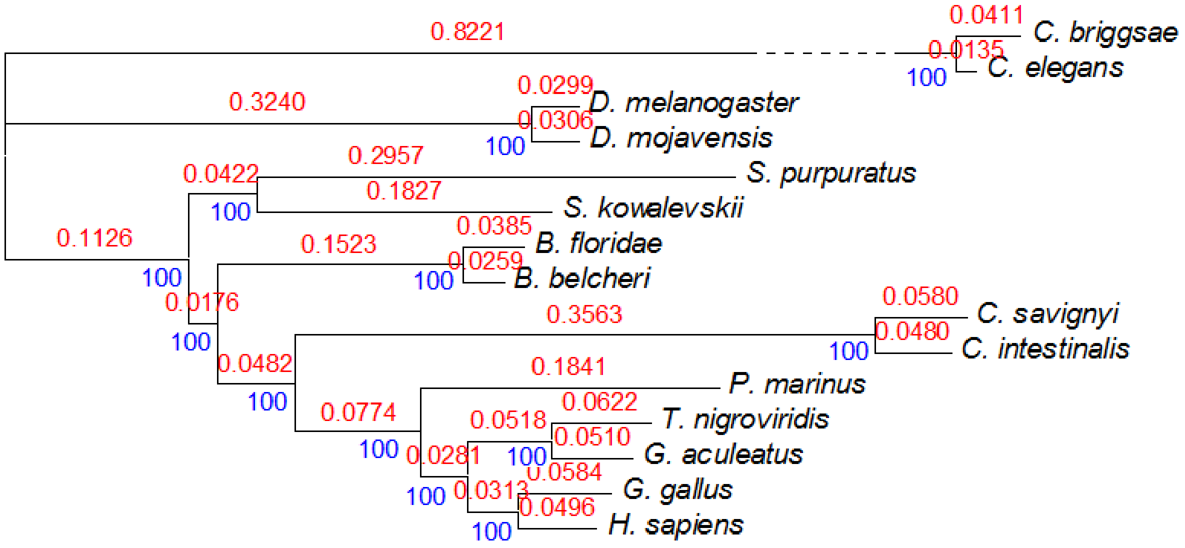

B. Maximum likelihood phylogenetic analysis of alignment 3 (containing 513 genes and 72,795 indel-free sites). Statistical supports and branch length are shown in blue and red color respectively. See Supplementary Note 3 for details.

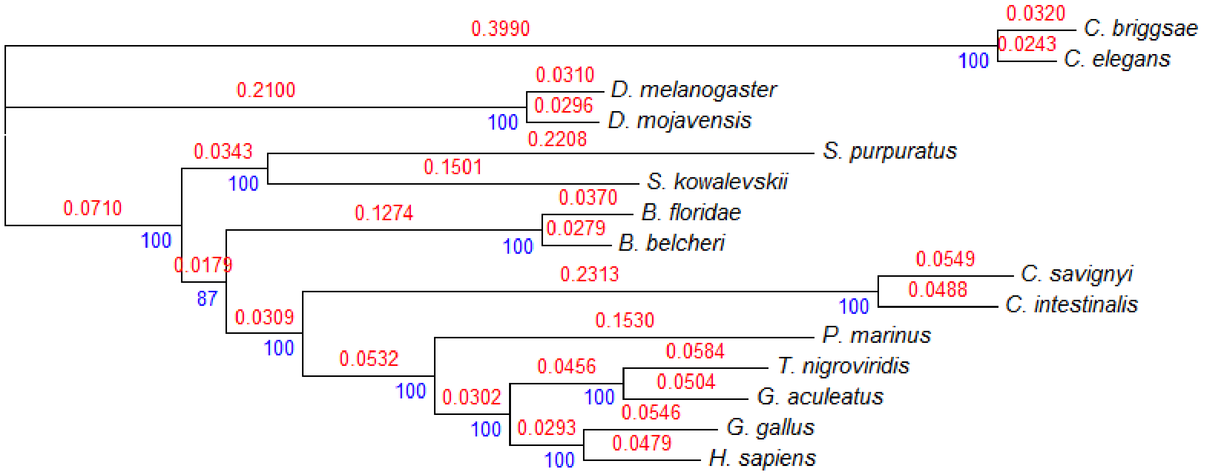

C. Maximum likelihood phylogenetic analysis of alignment 2 (containing 729 genes and 245,205 sites). Only branch length was estimated. See Supplementary Note 3 for details.

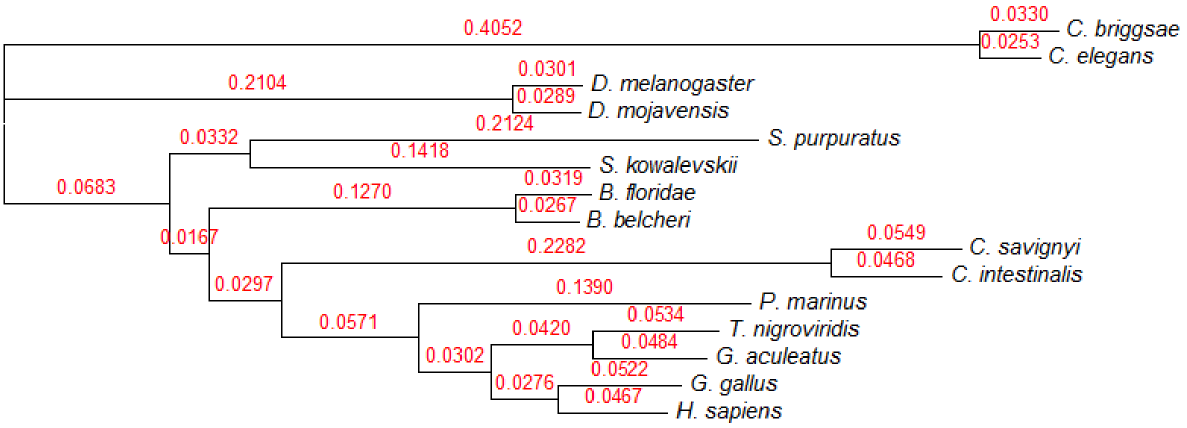

D. Maximum likelihood phylogenetic analysis of alignment 1 (containing 729 genes and 403,674 sites). Only branch length was estimated. See Supplementary Note 3 for details.

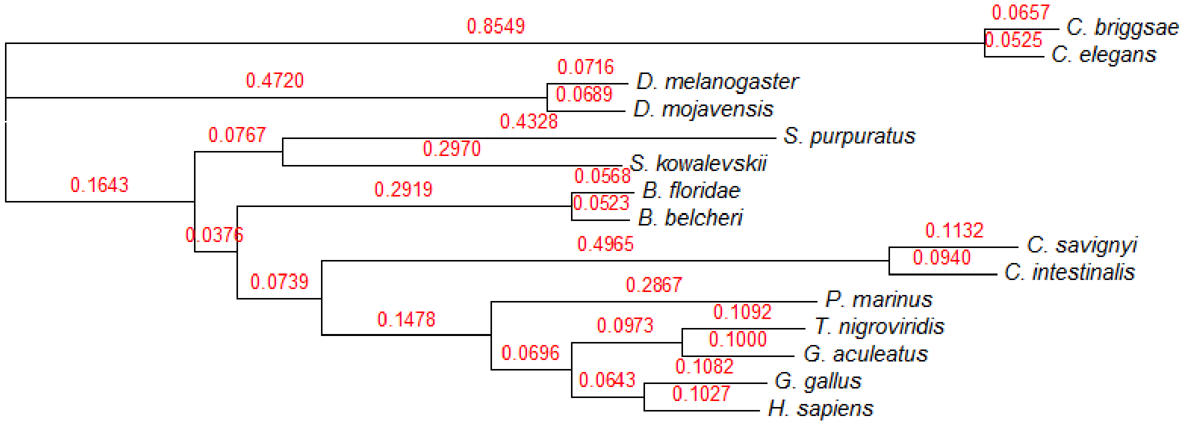

E. Bayesian molecular dating analysis using PhyTime and Phynobayes. The analysis was based on alignment 3 (containing 513 genes and 72,795 indel-free sites) and the obtained tree topology. See Supplementary Note 3 for details.

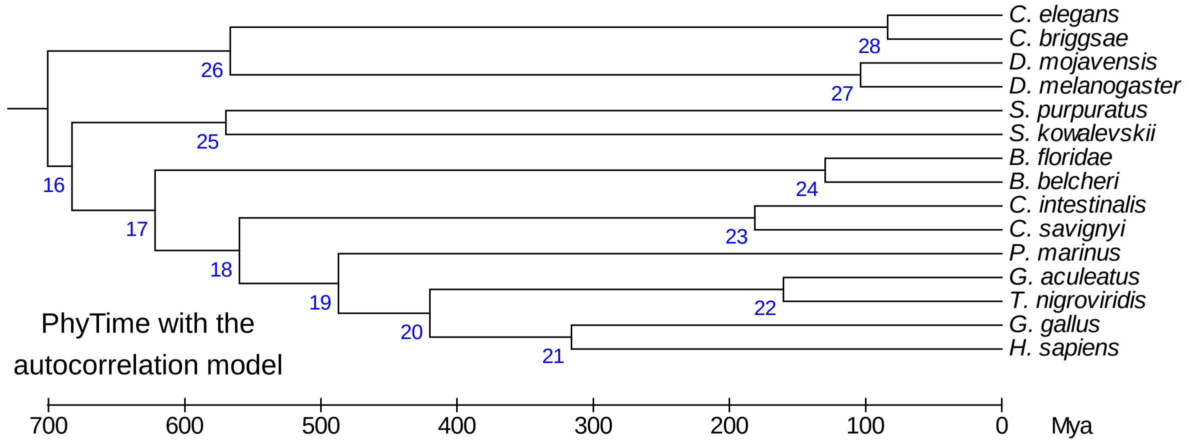

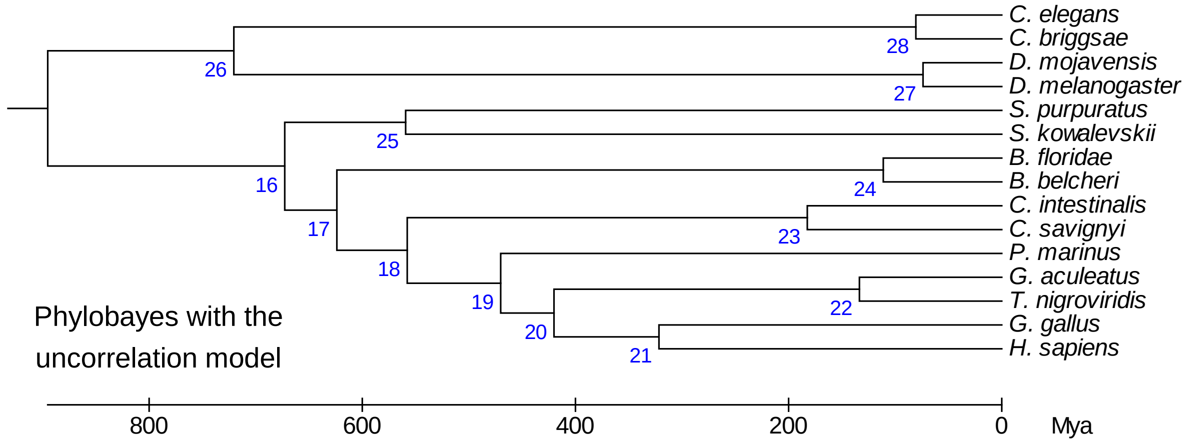

Table: The inferred divergence times and their 90% HPD.

Node ID	Autocorrelated model (PhyTime)*			Uncorrelation model (Phylobayes)**			Reconciled date
	meandate	inf95%	sup95%	Meandate	inf95%	sup95%	(average over two models)
root	700.7	625.9	725.0	895.0	747.1	1264.1	797.8
16	682.7	623	699.99	672.5	627.1	698.6	677.6
17	621.8	587.27	649.76	623.3	574.2	648.9	622.6
18	560.0	528.1	586.9	557.8	509.6	595.9	558.9
19	487.4	468.43	503.41	470.0	441.4	510.3	478.7
20	419.9	416.02	424.47	420.1	416.1	424.7	420.0
21	315.9	312.01	327.63	321.5	312.4	330.5	318.7
22	160.2	143.98	166	133.5	100.6	164.4	146.9
23	181.3	169.22	197.15	182.2	169.5	198.0	181.8
**24**	**129.6**	**109.2**	**139.98**	**110.9**	**90.8**	**137.8**	**120.3**
25	569.8	532	600.3	559.1	489.9	635.9	564.4
26	566.7	541.1	600.7	720.4	580.2	797.5	643.5
27	103.8	86.26	109.97	73.8	42.4	107.6	88.8
28	84.0	60.04	108.02	80.4	60.7	109.9	82.2

* Autocorrelated model of clock relaxation, with LG matrix, 16 gamma categories and the GBS model.

**Ucorrelated gamma multipliers model of clock relaxation, with the birth-death prior on divergence time.

Supplementary Figure 4. Sequence identity of orthologous genes between two lancelets

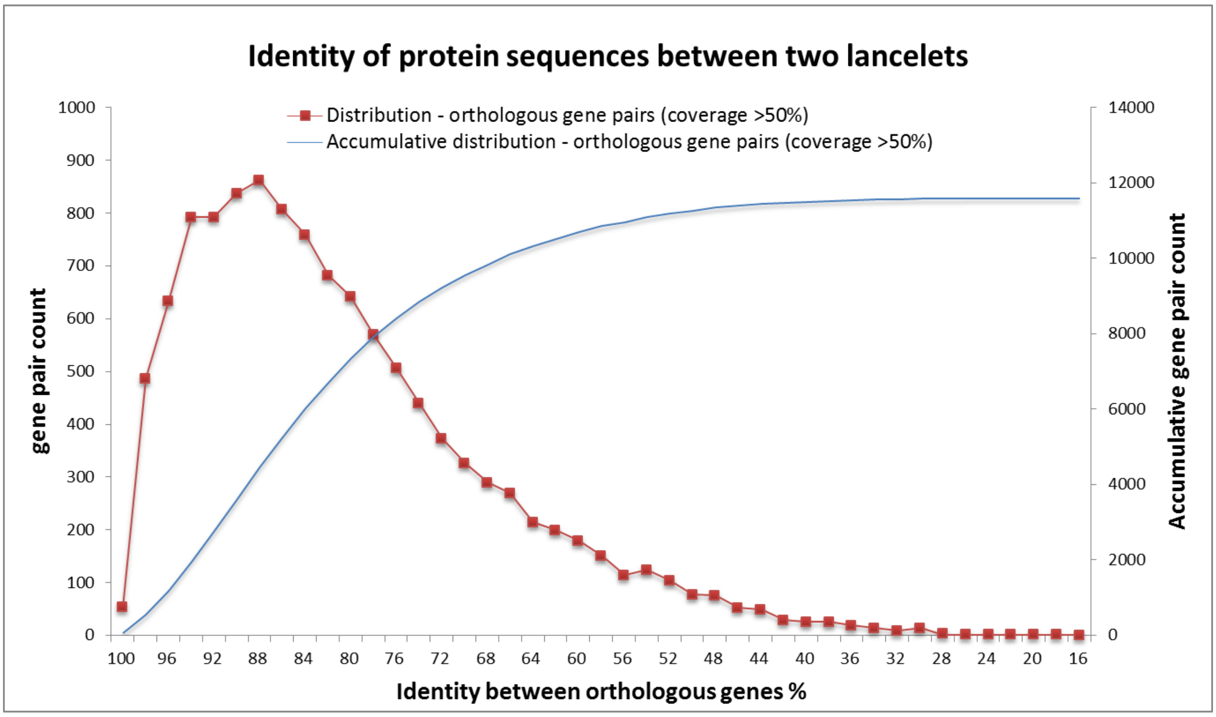

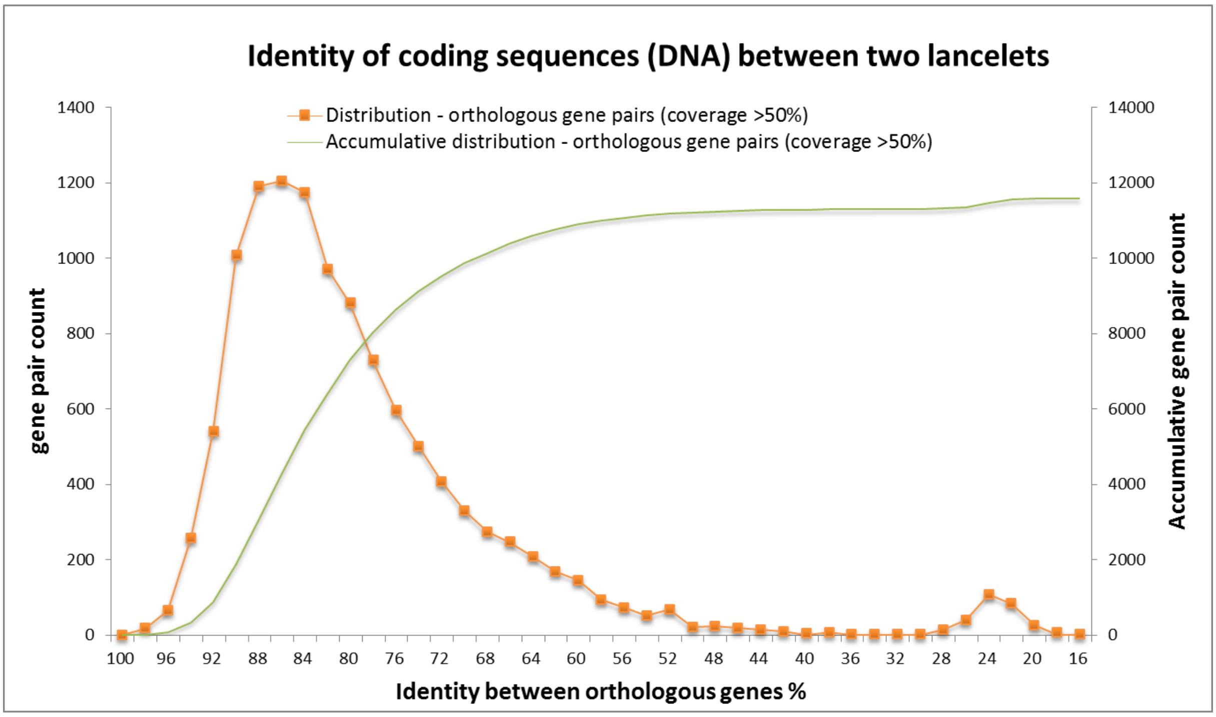

Supplementary Figure 5. Distribution of sequence identity and alignment coverage of orthologous introns between two lancelets

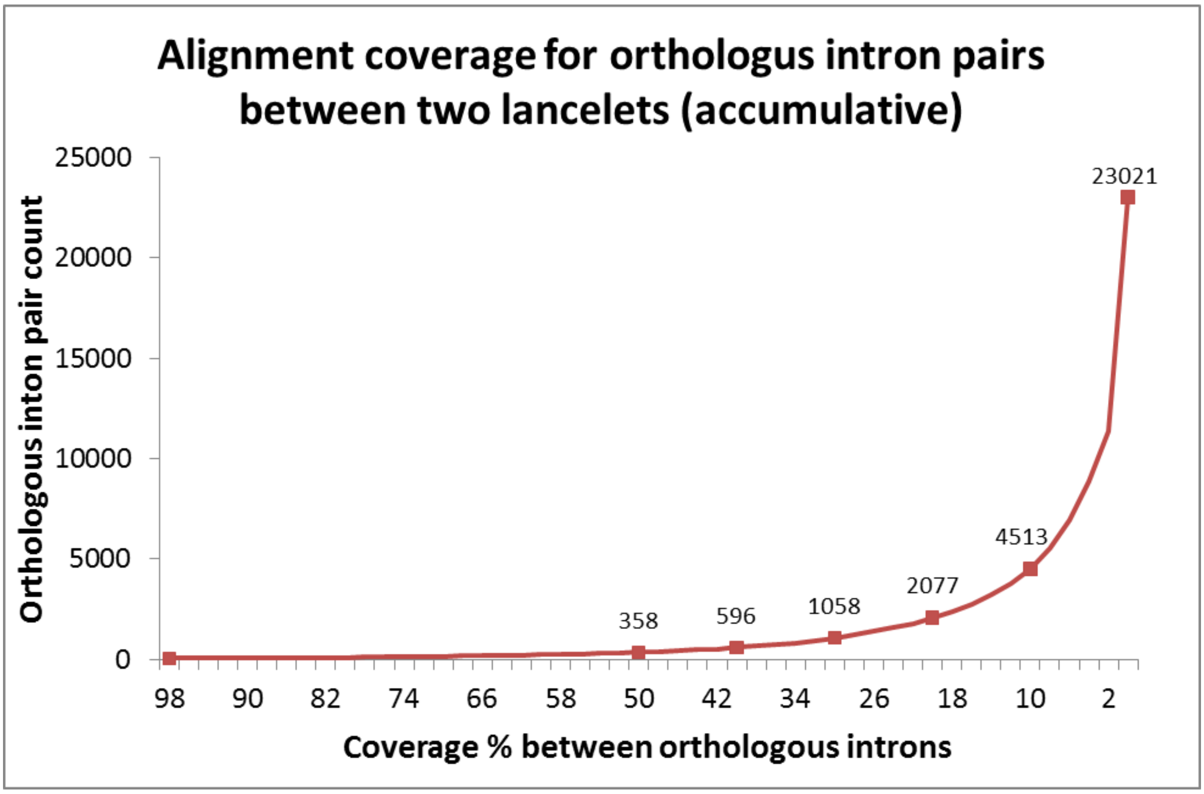

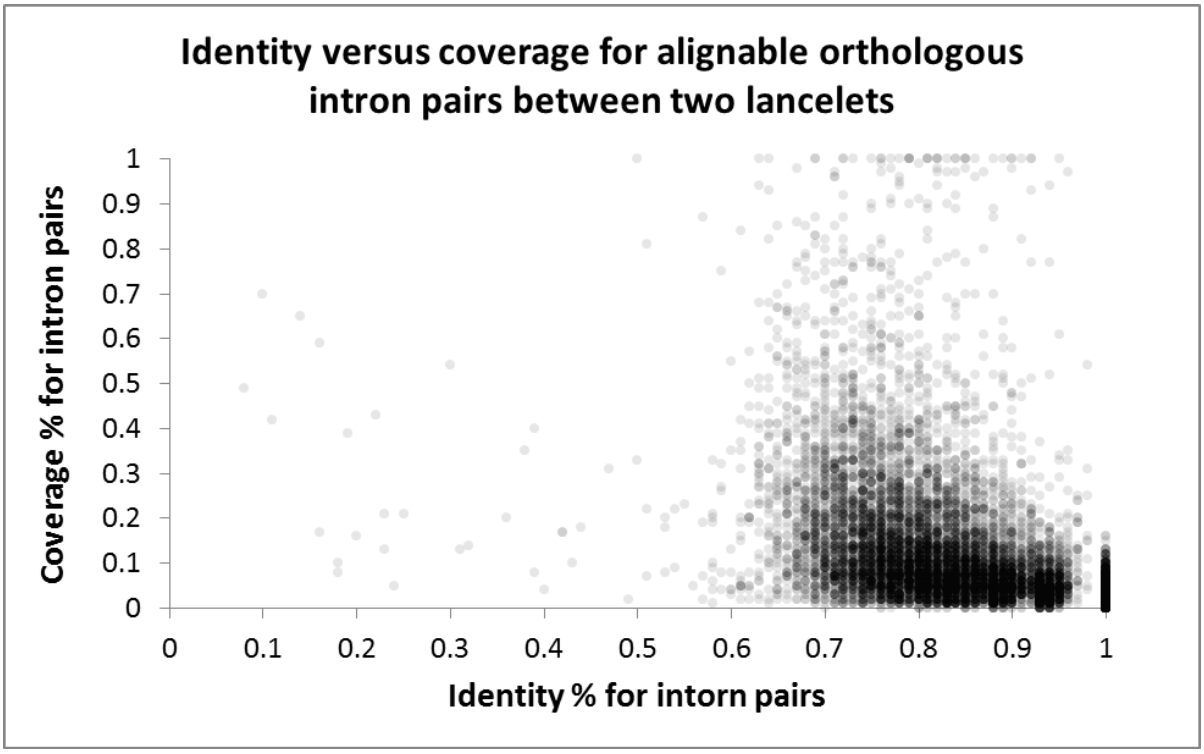

Supplementary Figure 6. The cumulative distribution of the parwise distance of 1:1 ortholog proteins of six species pairs.

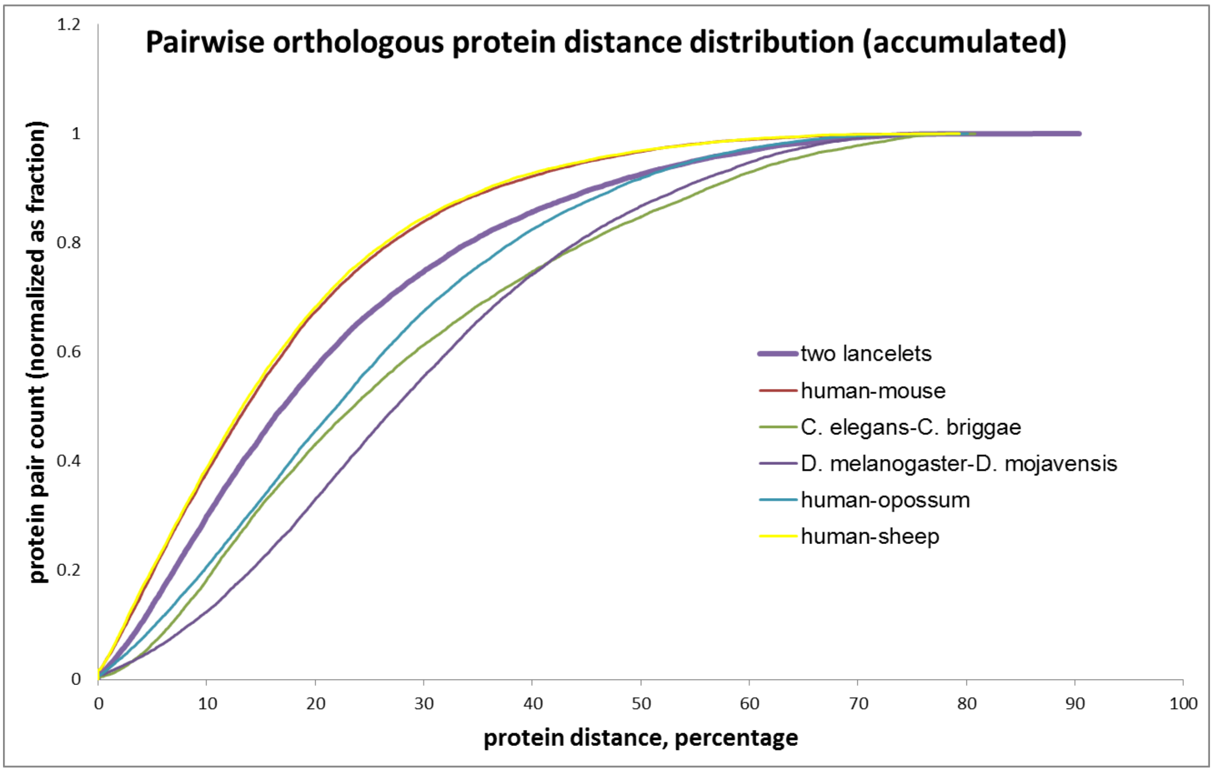

Supplementary Figure 7. Distribution of mismatches in 50bp windows in alignments

A stepping size of 25bp is used for the sliding window analysis. The poisson distribution and the geometric distribution having the same mean numbers are superimposed on the actual distribution of mismatches, showing that the mismatch distribution fits better to the geometric distribution.

A. Indels are not considered. The mean mismatches for each window is 2.18

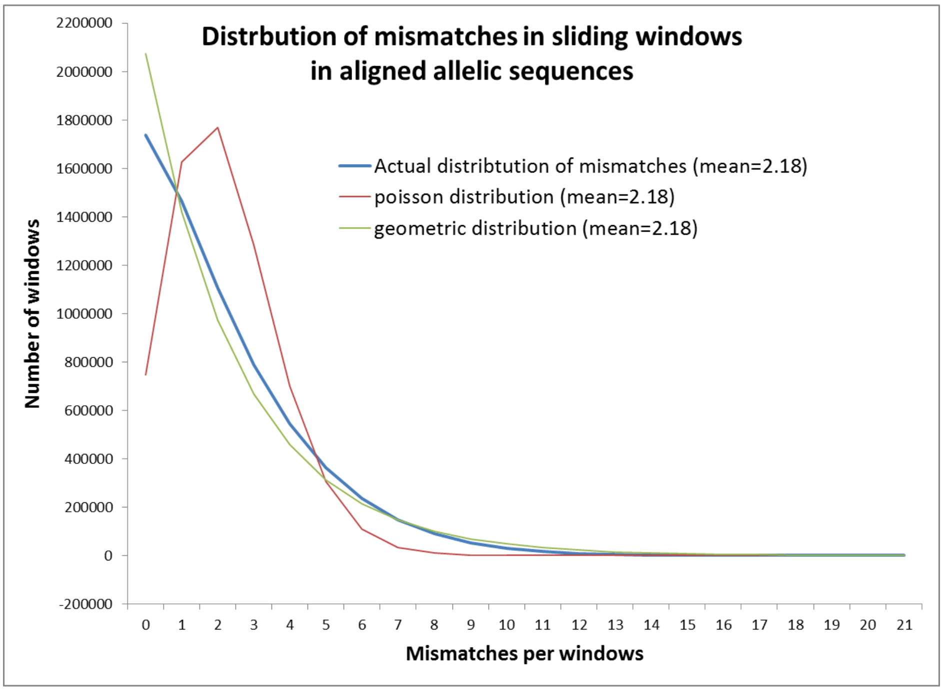

B. Indels are treated as point mismatches. The mean mismatches for each window is 2.67

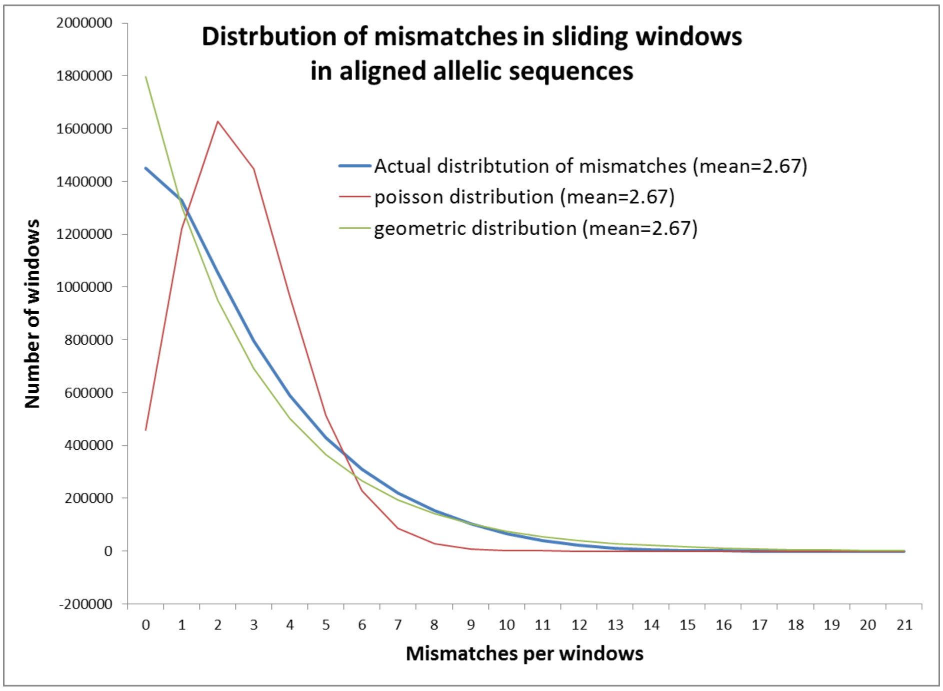

Supplementary Figure 8. Distribution of mismatches in 100bp windows in alignments

A stepping size of 25bp is used for the sliding window analysis. The poisson distribution and the geometric distribution having the same mean numbers are superimposed on the actual distribution of mismatches, showing that the mismatch distribution fits better to the geometric distribution.

A. Indels are not considered. The mean mismatches for each window is 4.32

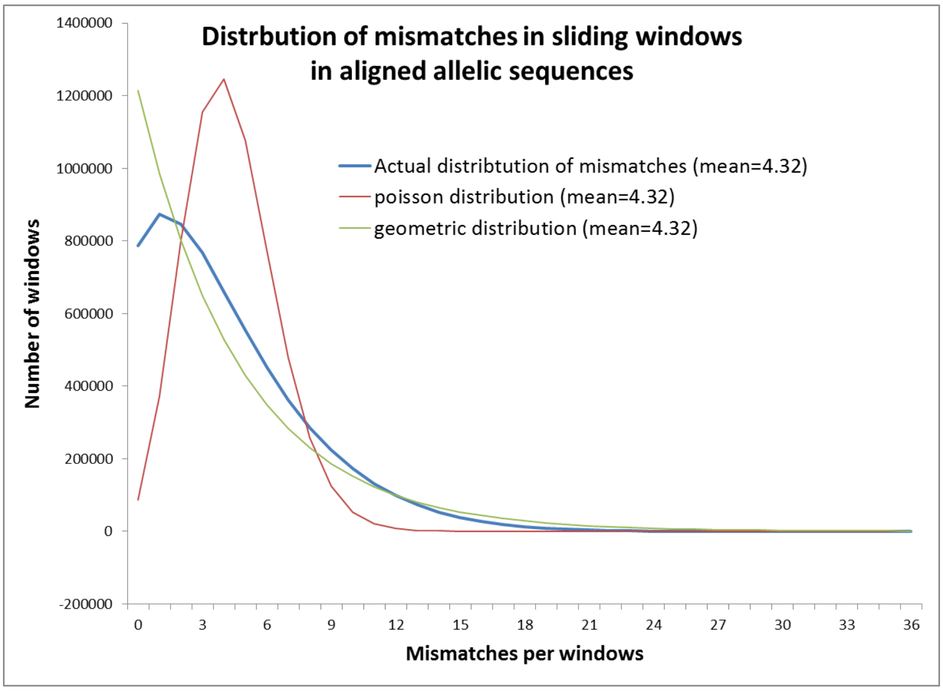

B. Indels are treated as point mismatches. The mean mismatches for each window is 5.29

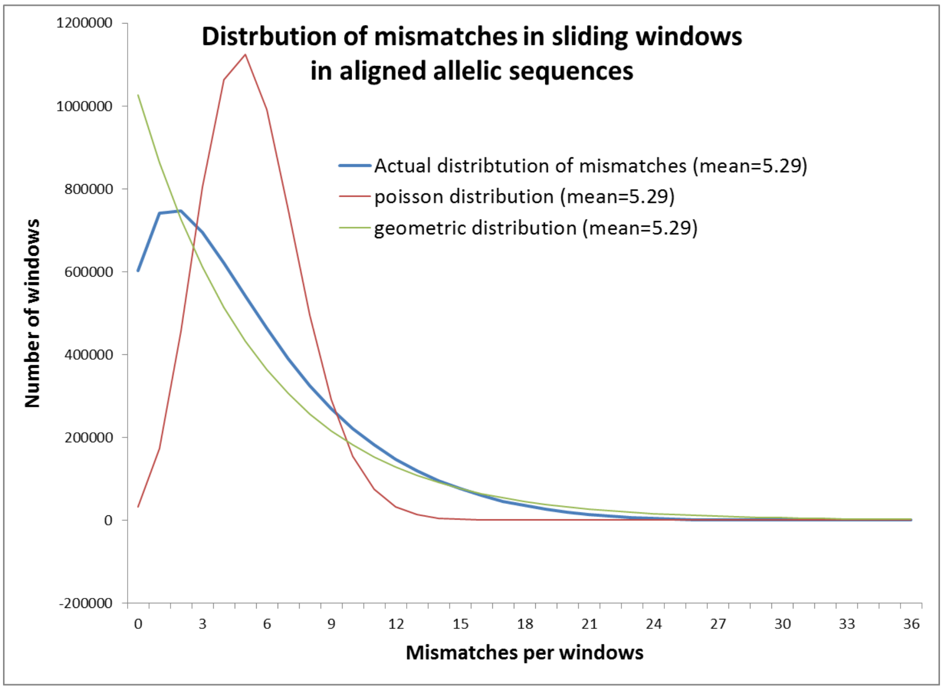

Supplementary Figure 9. Distribution of mismatches in 200bp windows in alignments

A stepping size of 25bp is used for the sliding window analysis. The poisson distribution and the geometric distribution having the same mean numbers are superimposed on the actual distribution of mismatches, showing that the mismatch distribution fits better to the geometric distribution.

A. Indels are not considered. The mean mismatches for each window is 8.54

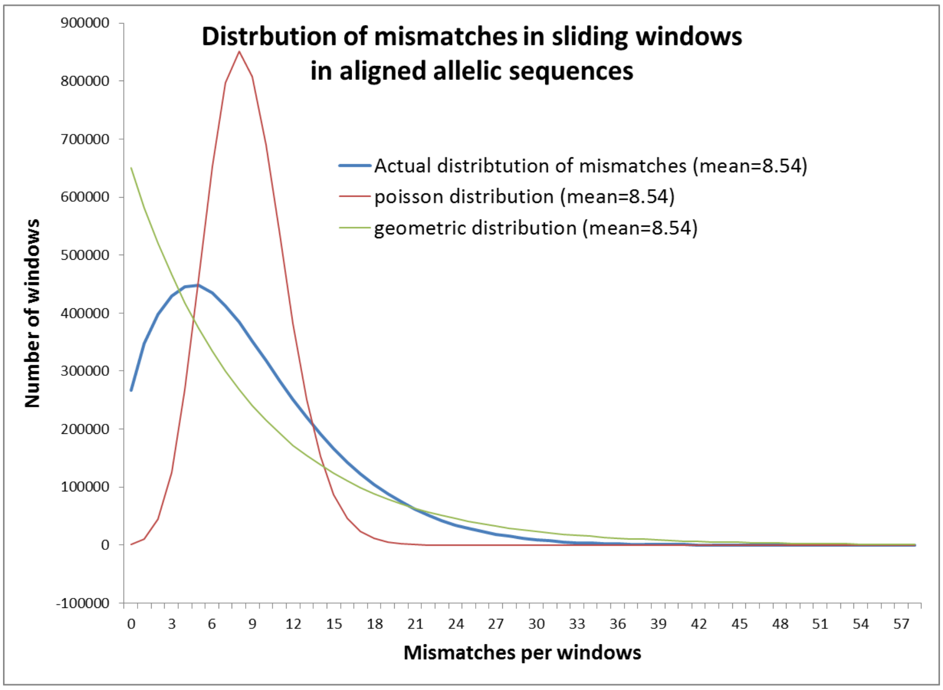

B. Indels are treated as point mismatches. The mean mismatches for each window is 10.46

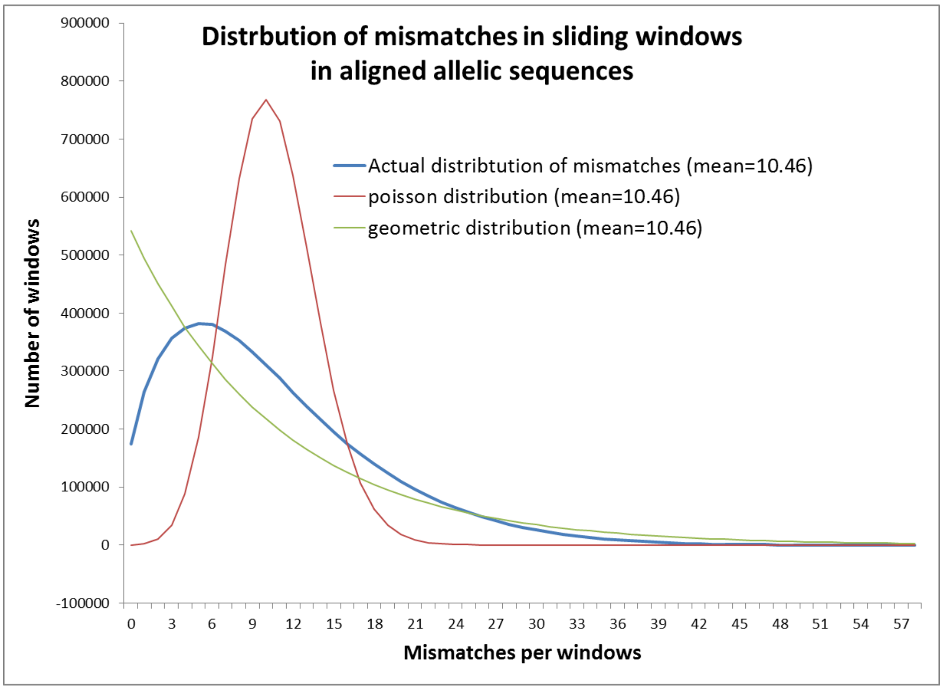

Supplementary Figure 10. Distribution of sizes of polymorphic indels

A. Indel sizes of 1-1500bp

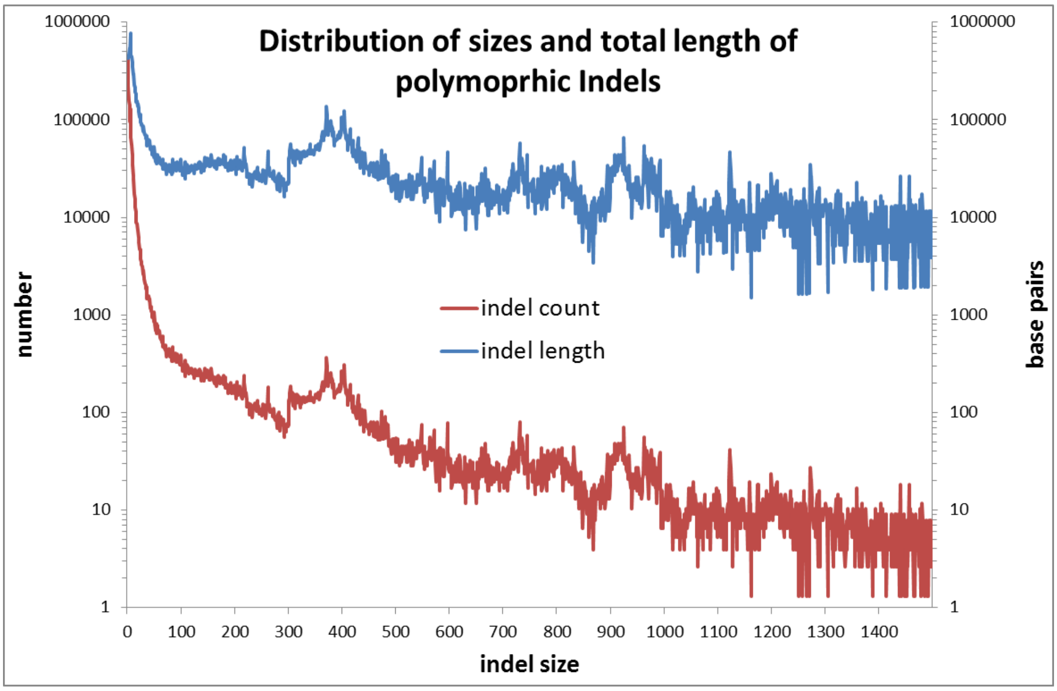

B. Indel sizes of 1-50bp

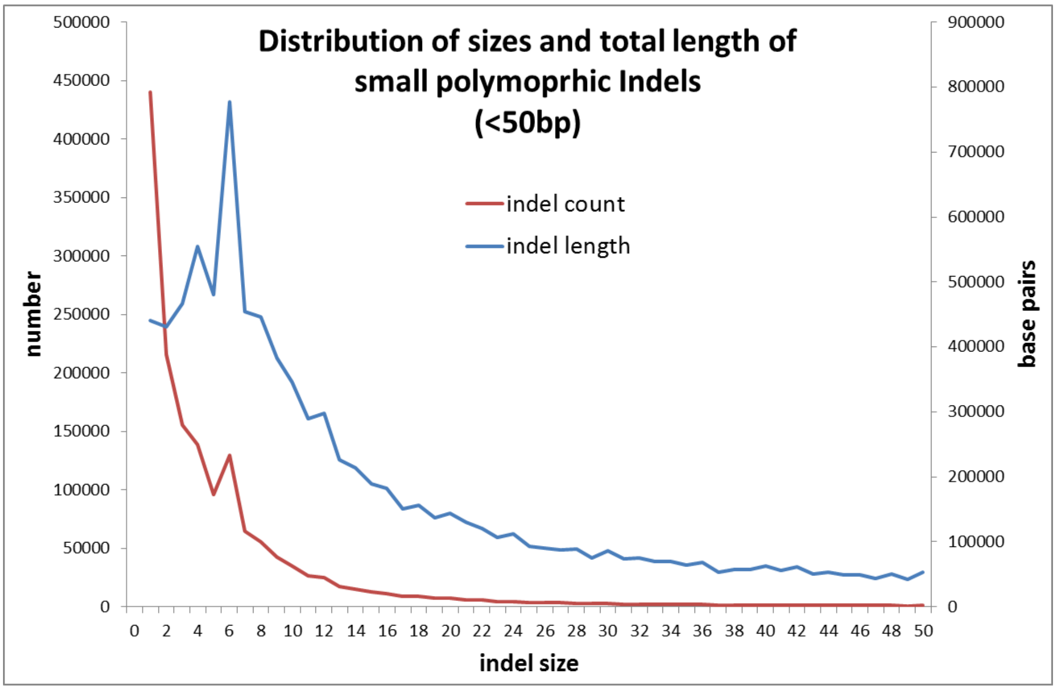

Supplementary Figure 11. Distribution of length of ungapped alignments

A. All ungapped alignments between haplotypes

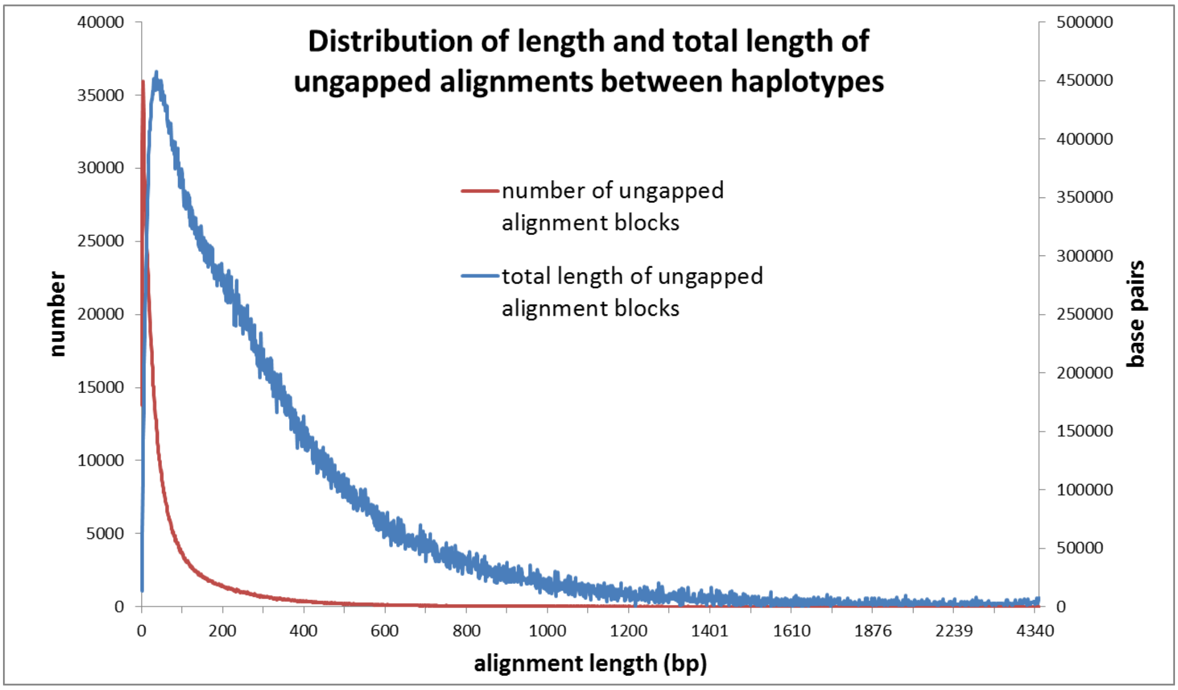

B. Small (<400bp) ungapped alignments between haplotypes

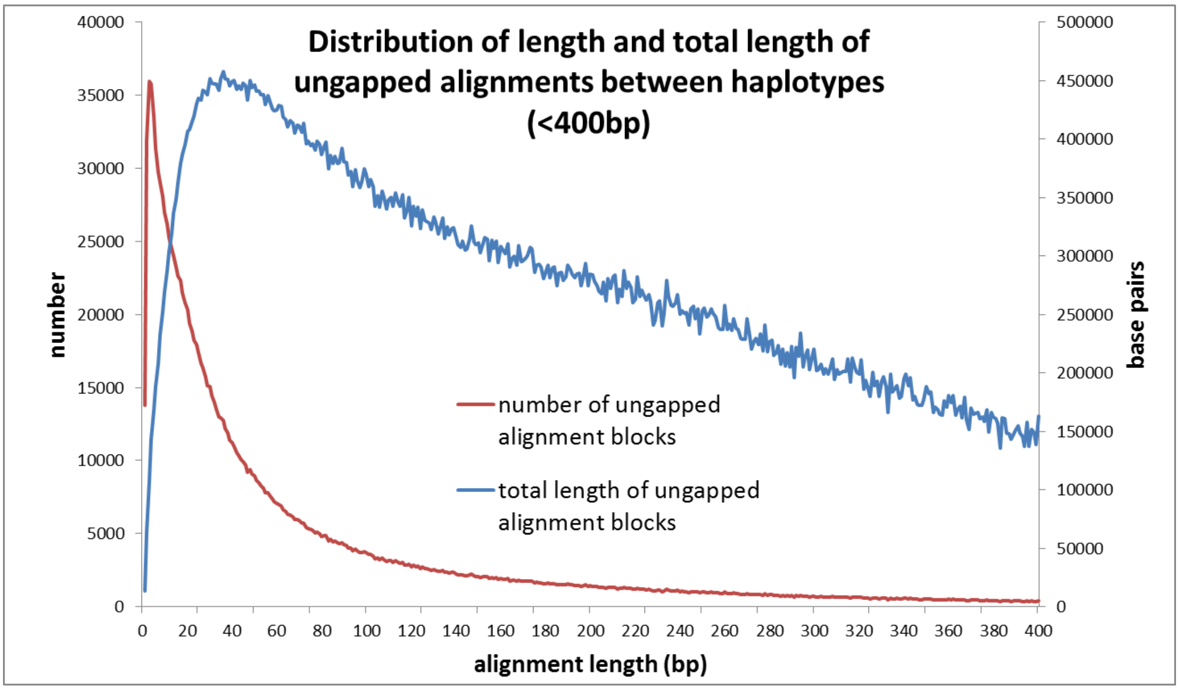

Supplementary Figure 12. Distribution of sizes of translocations (>100bp)

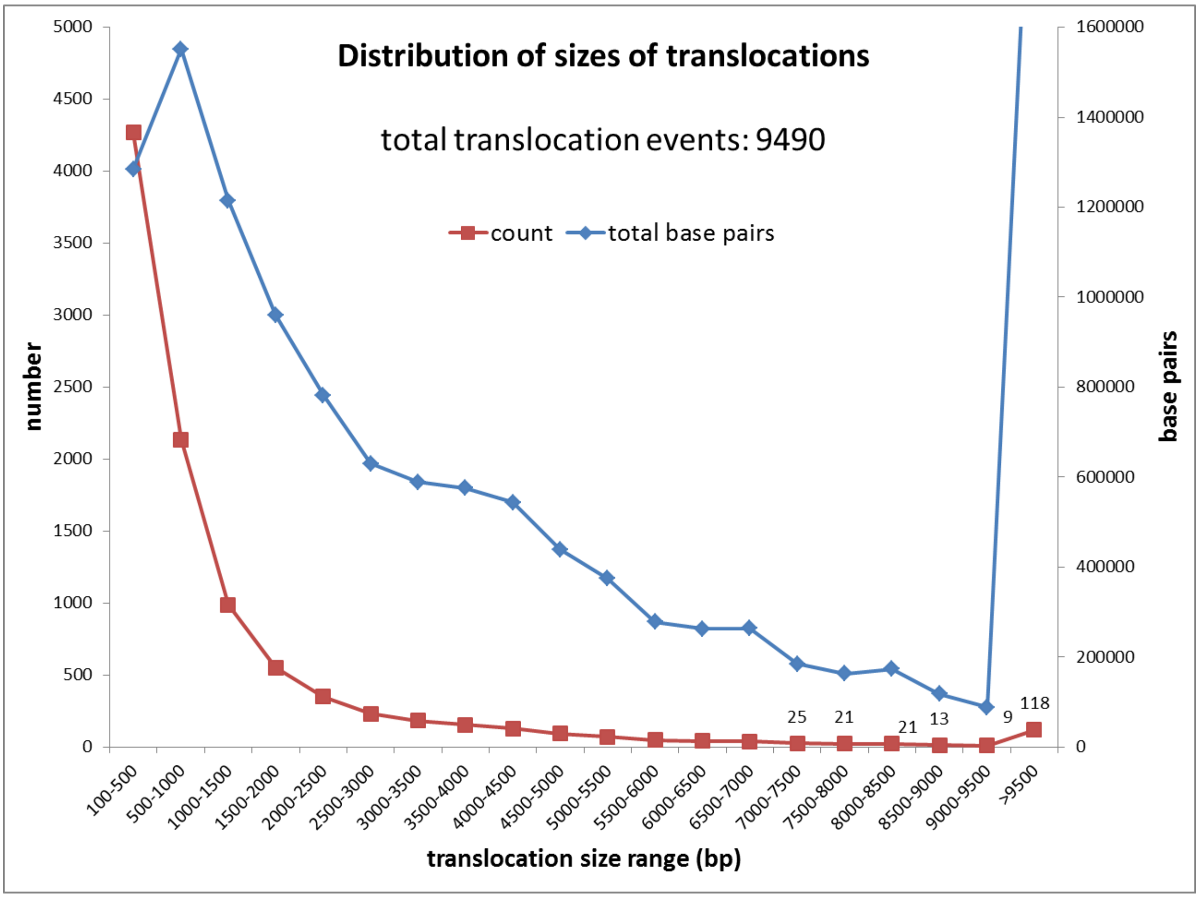

Supplementary Figure 13. Distribution of sizes of inversions (>100bp)

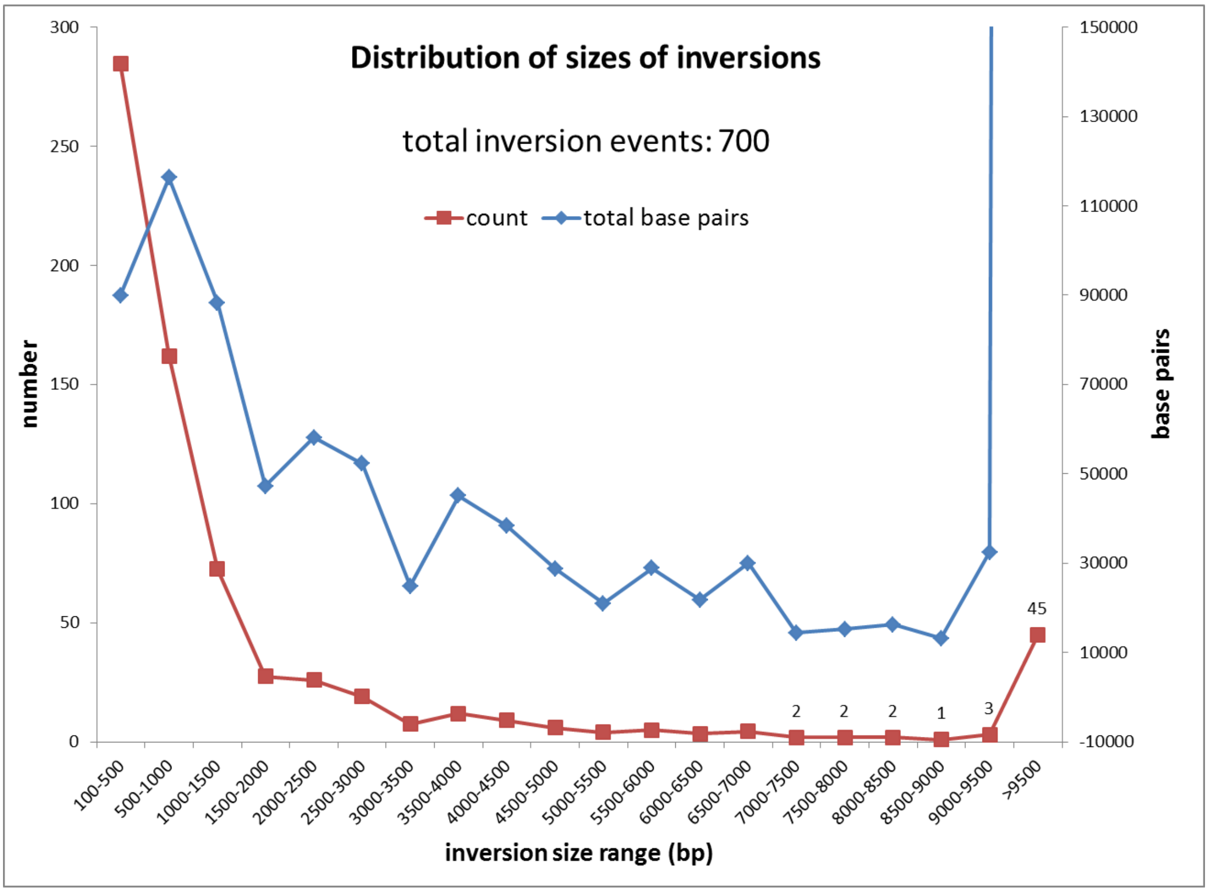

Supplementary Figure 14. Examples of large polymorphic indels versus repetitive DNA

Repetitive DNA regions are superimposed on the pairwise alignments between alleles.

1. scf220164597062(X) versus scf220164596780

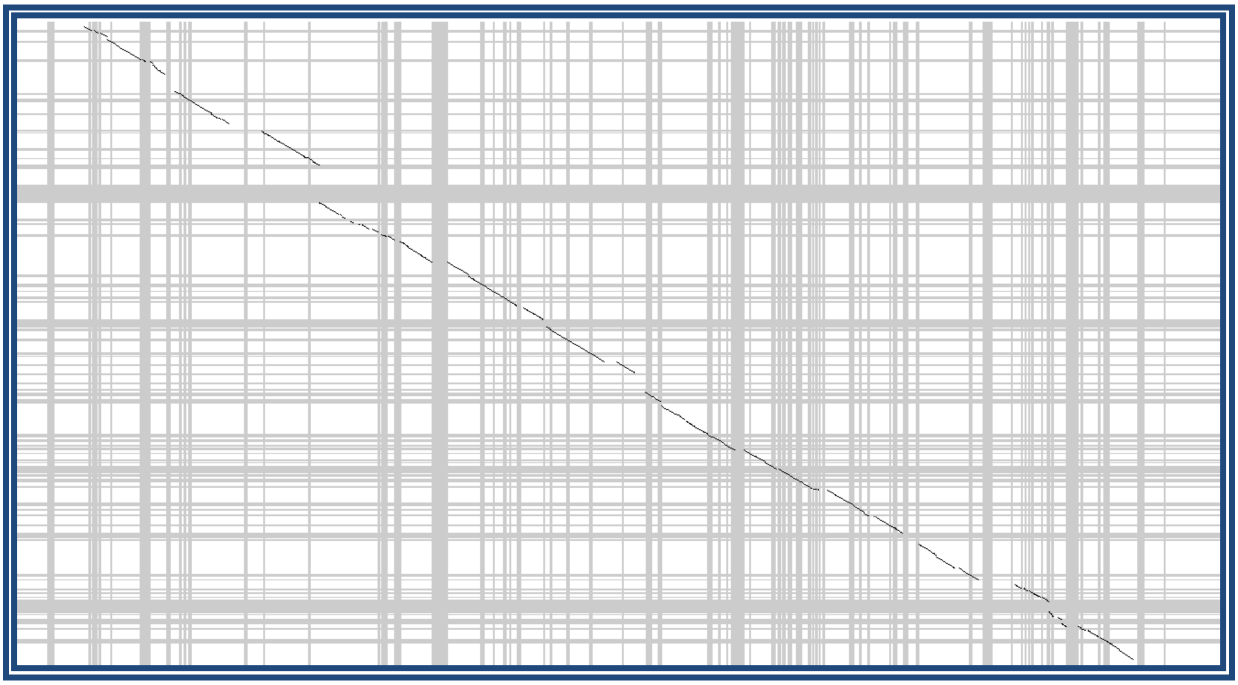

2. scf220164596568(X) versus scf220164596683(Y)

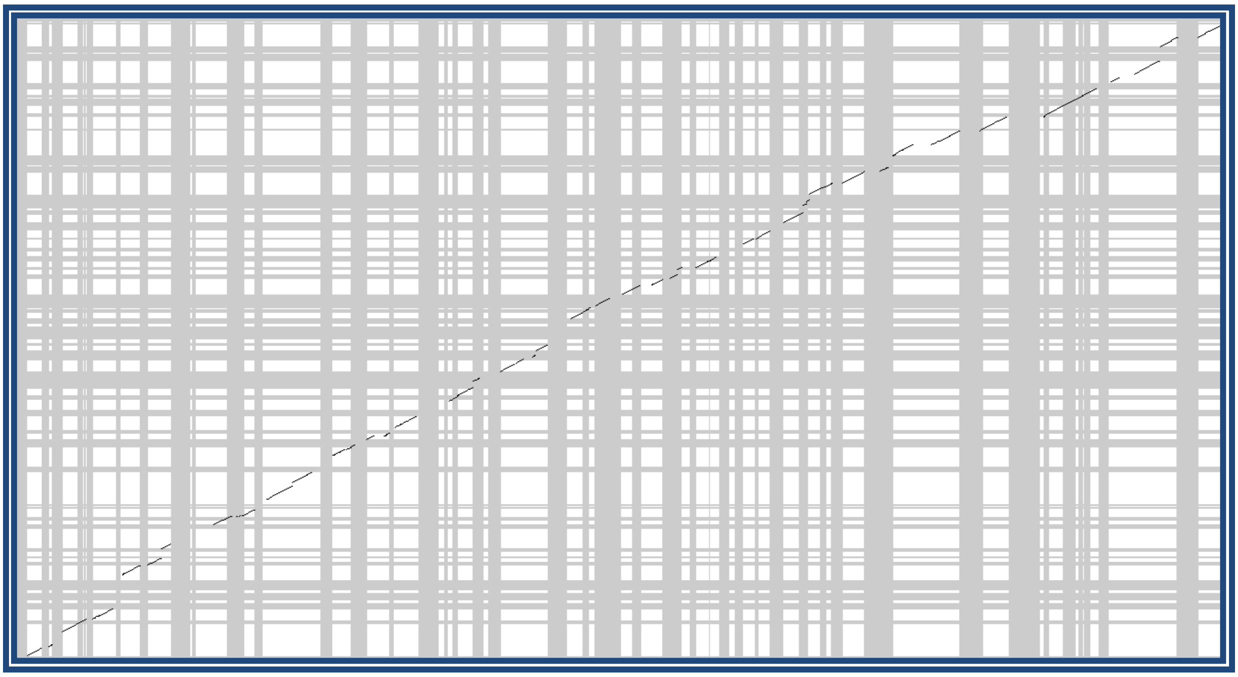

3. scf220164597061(X) versus scf220164595321(Y)

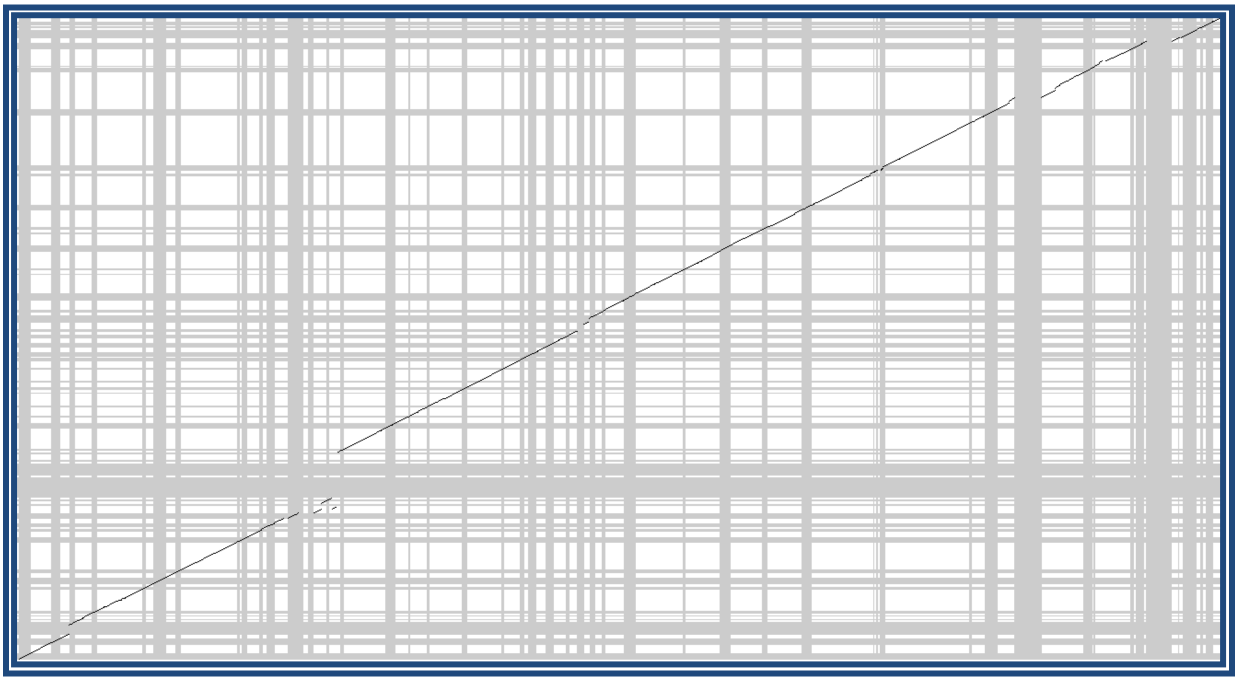

4. scf220164597055(X) versus scf220164595736(Y)

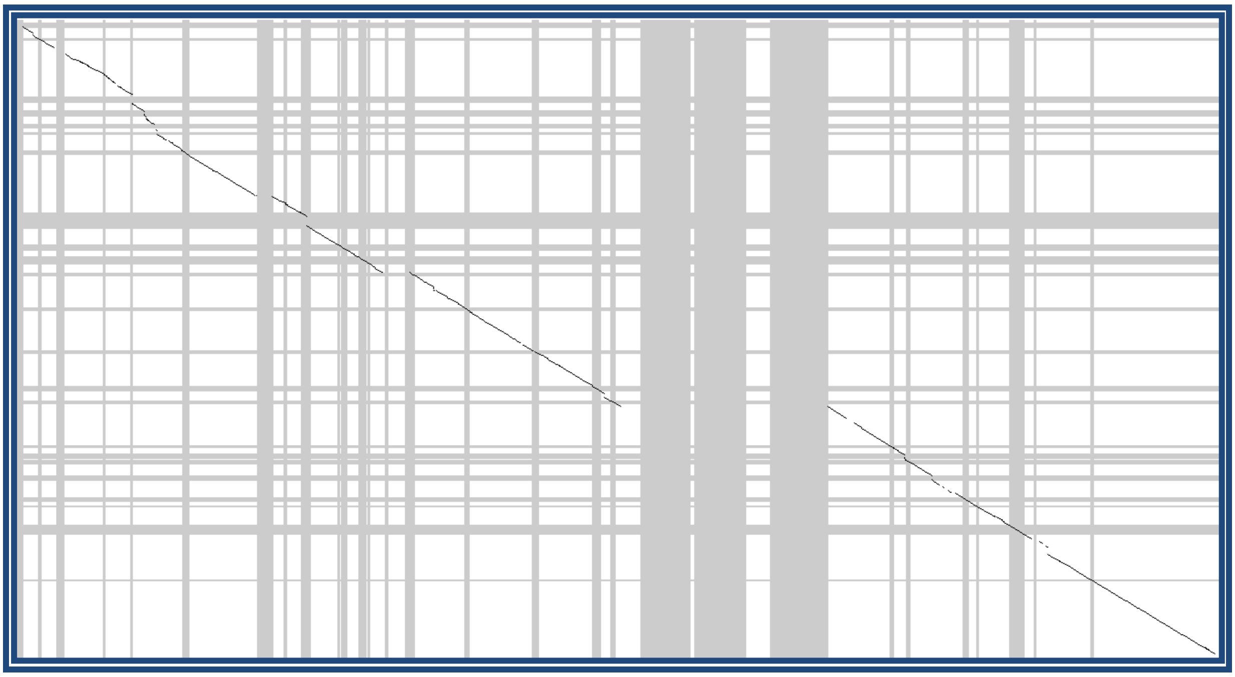

Supplementary Figure 15. The relative contribution of DNA transposons and retrotransposons

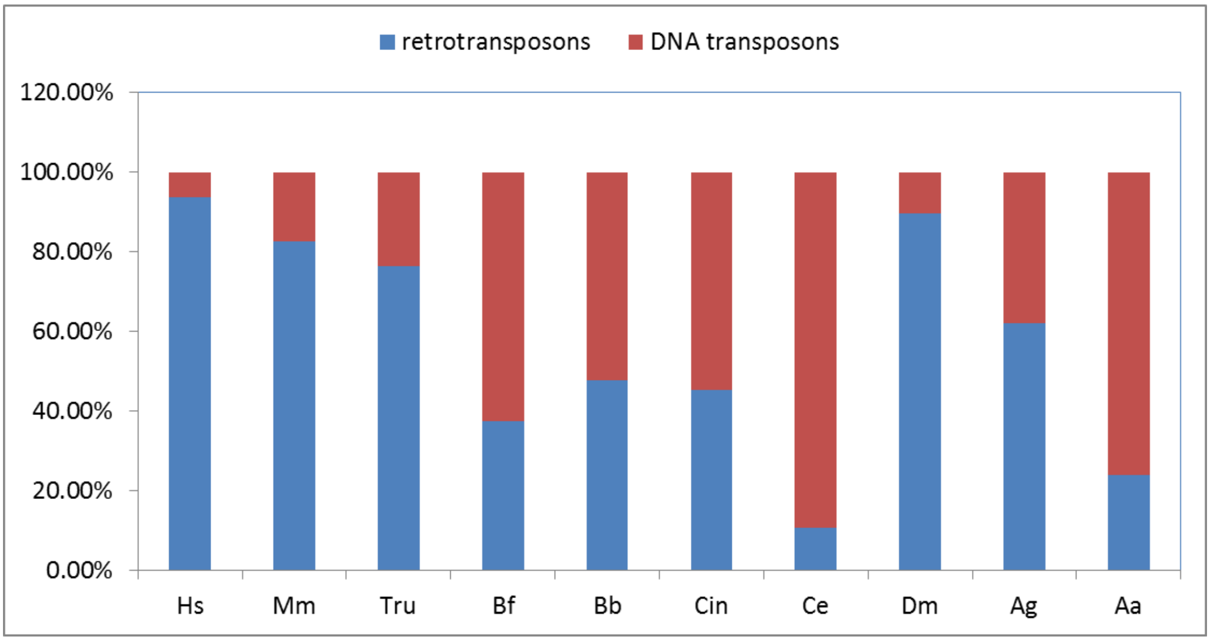

Hs: *Homo Sapiens*, Mm: *Mus musculus*, Tru: *Takifugu rubripes*, Bf:*Branchiostoma floridae*, Bb: *Branchiostoma belcheri*, Cin: *Ciona intestinalis*, Ag: *Anopheles gambiae*, Aa: *Aedes aegypti*, Dm: *Drosophila melanogaster*, Ce: *Caenorhabditis elegans*.

Supplementary Figure 16. Rearrangements between two urochordates

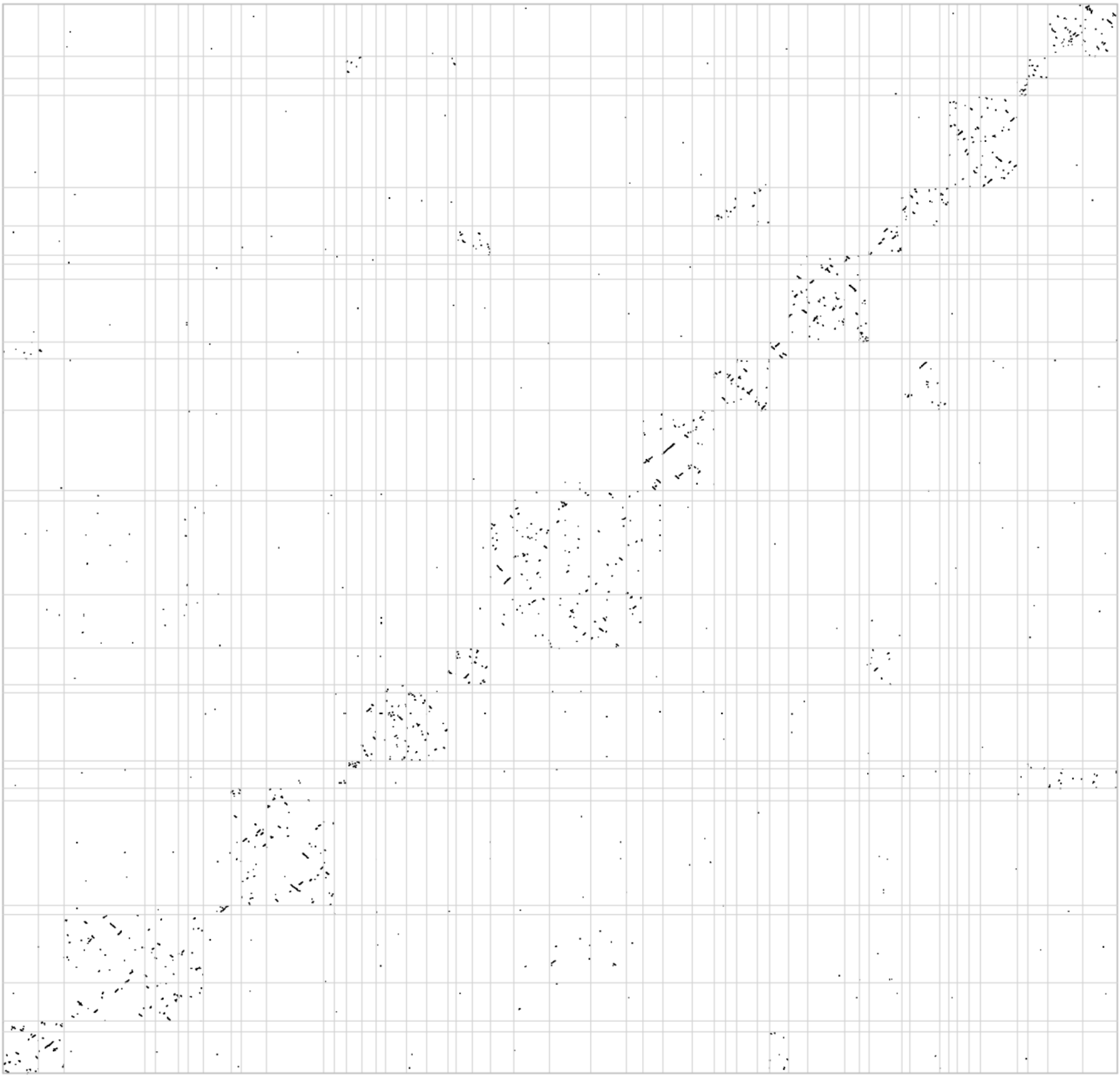

C. savignyi is on X-axis and *C. intestinalis* on Y-axis.

Supplementary Figure 17. Rearrangements between two fishes

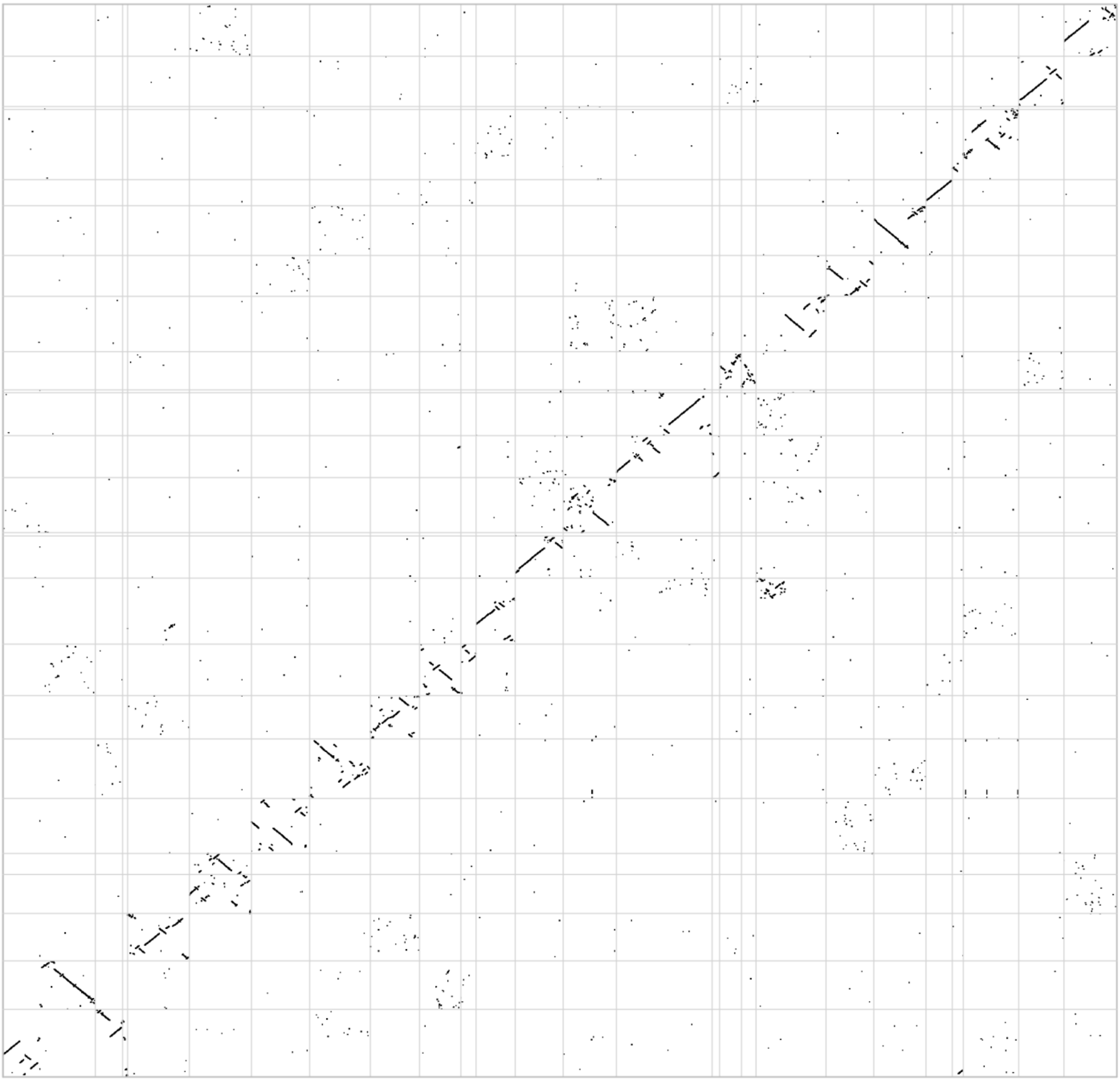

T. nigroviridis is on X-axis and *G. aculeatus* on Y-axis.

Supplementary Figure 18. Rearrangements between two tetrapods

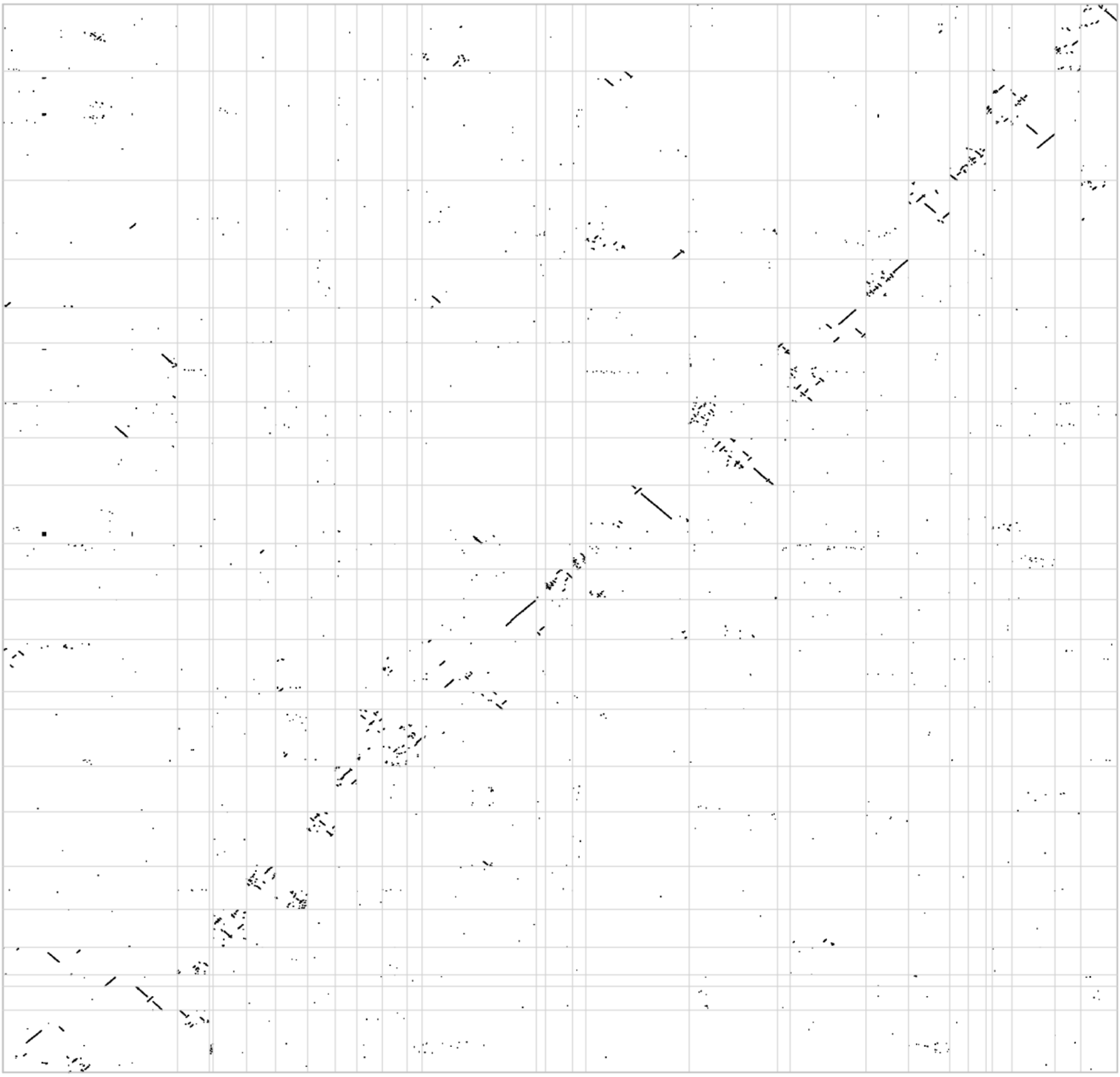

G.gallus is on X-axis and *H. sapiens* on Y-axis.

Supplementary Figure 19. Rearrangements between two lancelets

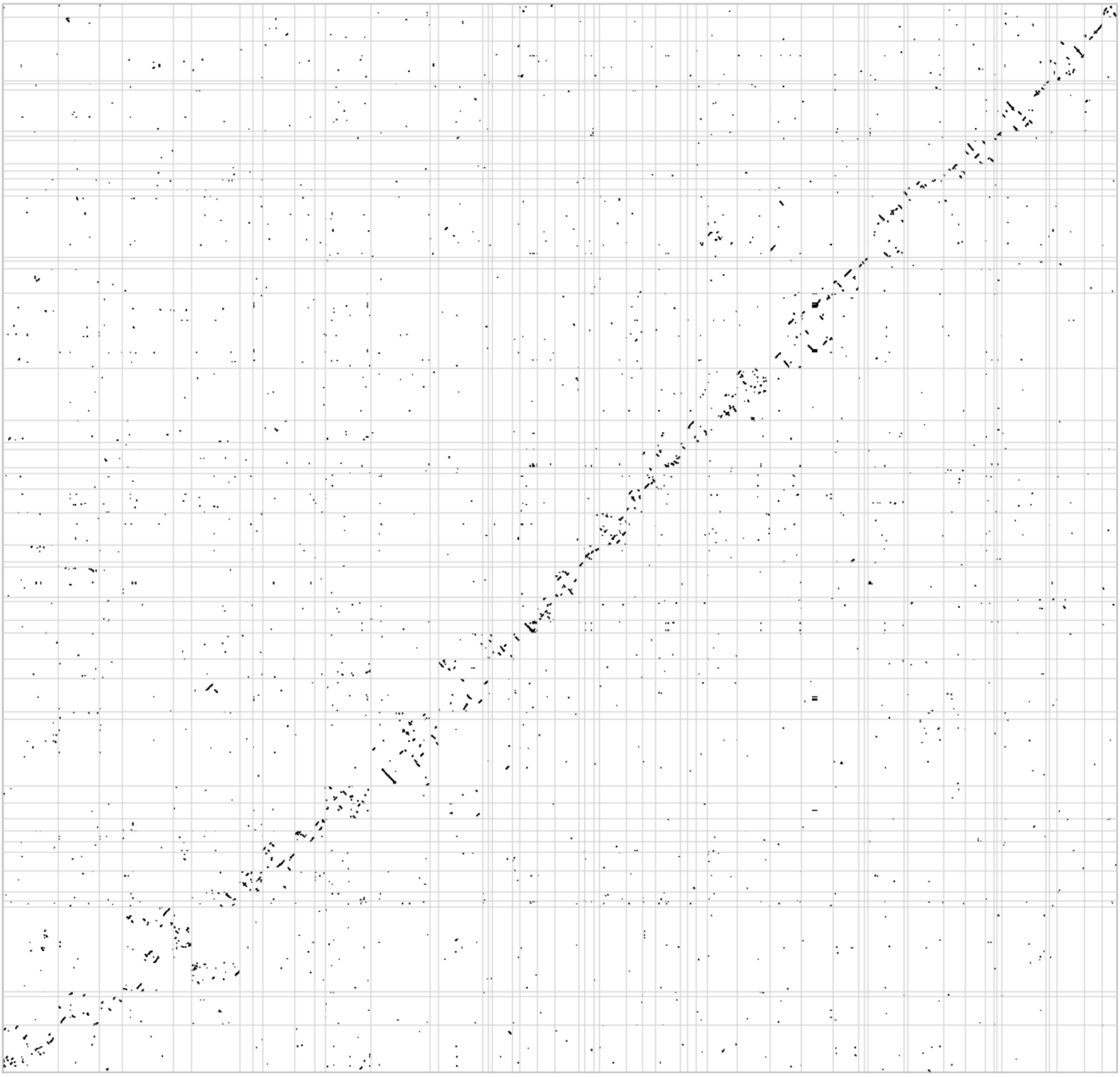

B. floridae is on X-axis and *B. belcheri* on Y-axis.

Supplementary Figure 20. Rearrangements between two worms

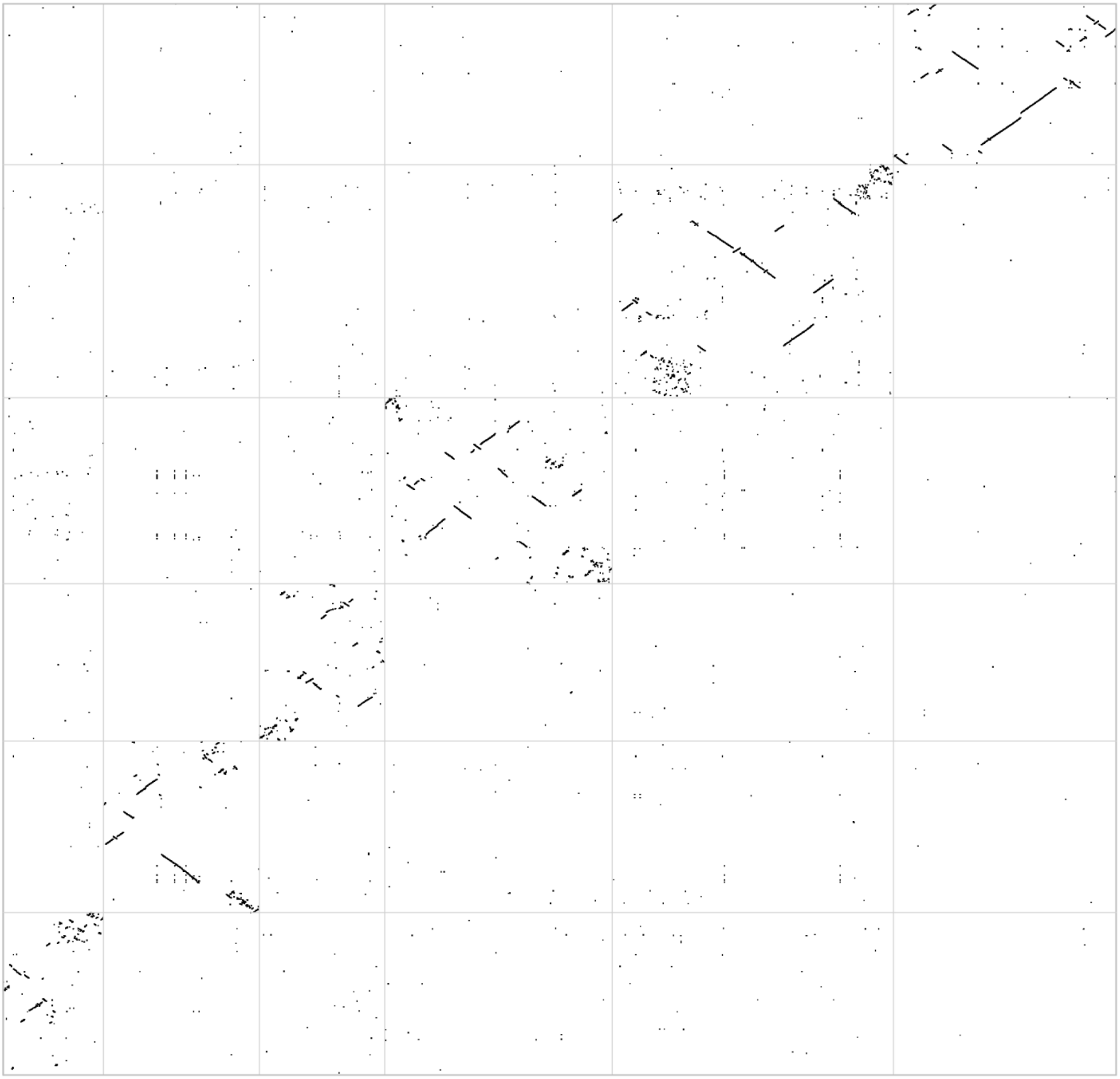

C. briggsae is on X-axis and *C.elegans* is on Y-axis.

Supplementary Figure 21. Rearrangements between two fruit flies

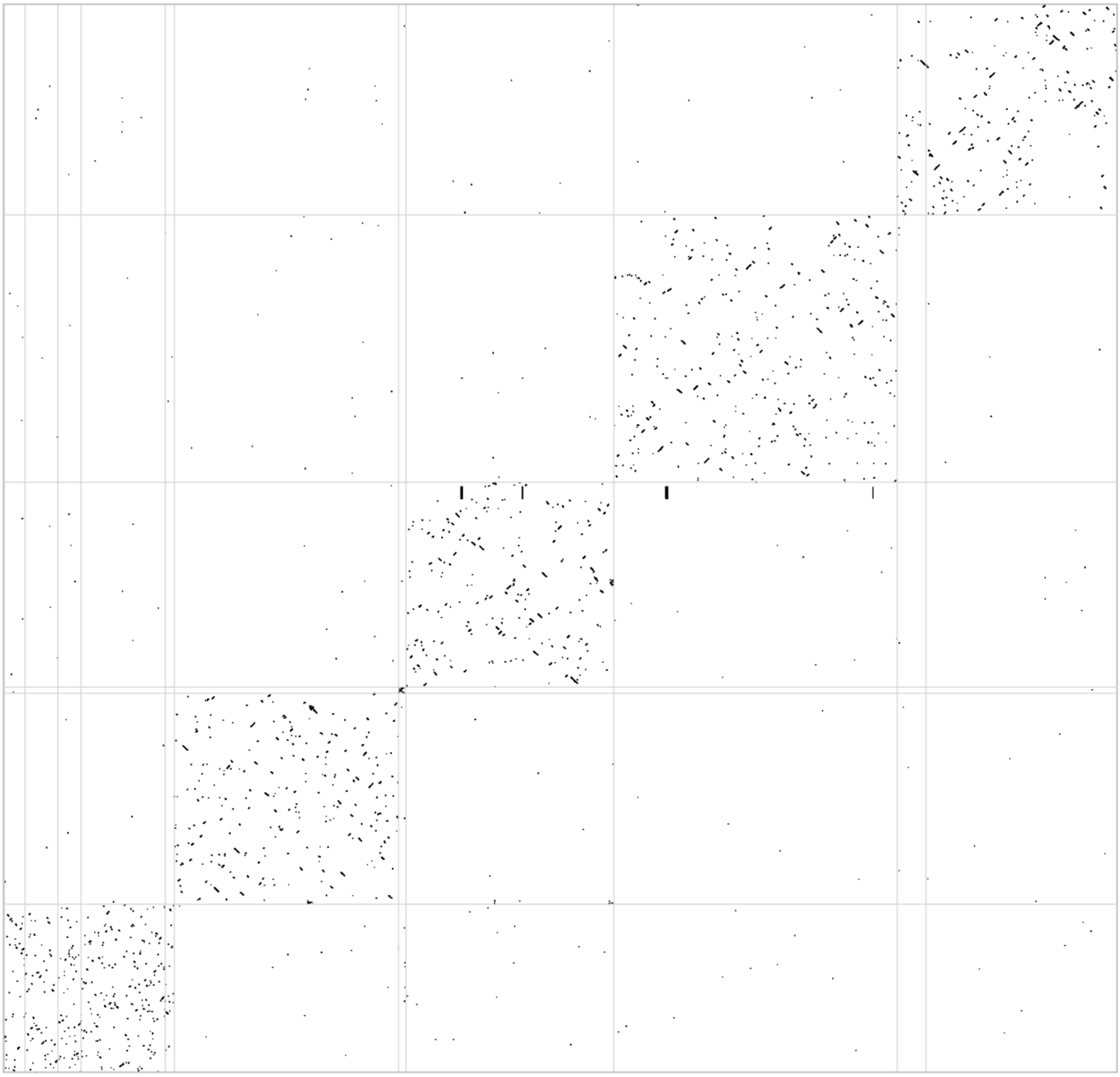

D.melanogaster is on X-axis and *D. mojavensis* on Y-axis.

Supplementary Figure 22. The decelerated gene rearrangement rates in vertebrates after 2R-WGD.

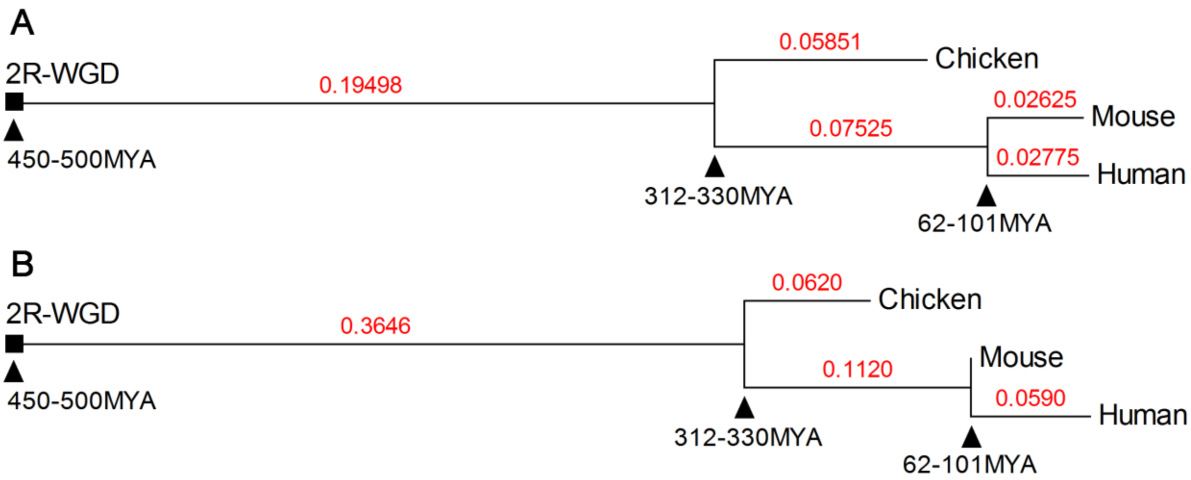

(A) The DCJ rearrangement distances from 2R-WGD to the current genomes (chicken, mouse and human) were calculated using genes with 2-4 ohnologs. **(B)** The DCJ rearrangement distances from 2R-WGD to the current genomes (chicken, mouse and human) were calculated using genes with 3-4 ohnologs. The following tables show the raw data used for this NJ distance tree reconstruction.

Total families of genes (human)			Number of genes in filled single copy	rearrangement	Relative rearrangement
1:2	1:3	1:4			
883	264	53	1200	1474	1474/(1200*4)= 0.307
	209	44	253	622	622/(253*4) = 0.615

Total families of genes (mouse)			Number of genes in filled single copy	rearrangement	Relative rearrangement
1:2	1:3	1:4			
916	283	74	1392	1601	1601/(1392*4)= 0.287
	233	61	395	742	742/(395*4) = 0.470

Total families of genes (chick)			Number of genes in filled single copy	rearrangement	Relative rearrangement
1:2	1:3	1:4			
780	178	20	1122	1137	1137/(1122*4)= 0.253
	124	16	237	403	403/(237*4)=0.425

Pairwise distance (1:1 ortholog number)	Mouse	Chicken
Human	0.054 (14058)	0.152 (9729)
Mouse		0.169 (9983)

Supplementary Figure 23. Statistics of the EST mapping against genome

A. The fraction of total CDS nucleotides covered by one or more ESTs

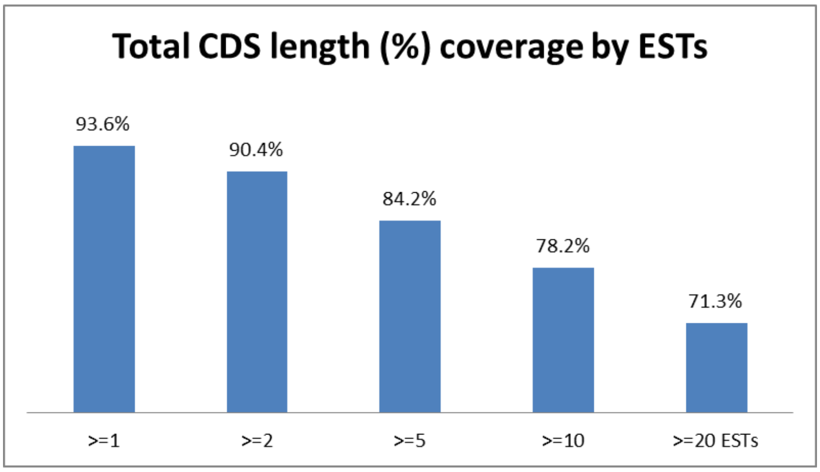

B. The fraction of total CDS number covered by one or more ESTs (note that a CDS is considered covered only if >50% of its length are covered)

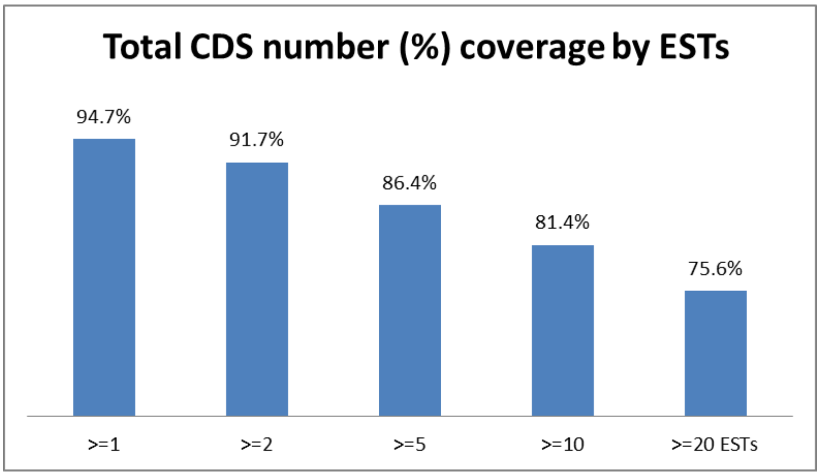

C. The fraction of ESTs mapped to five difference genomic regions. The genome is divided into five regions, including CDS, intron, intergenic and the up- and down-stream 2000bp of every gene. Note that we assigned EST to a certain region following this priority: CDS, intron, downstream, upstream and intergenic region, which clearly biased the count to CDS and genic regions.

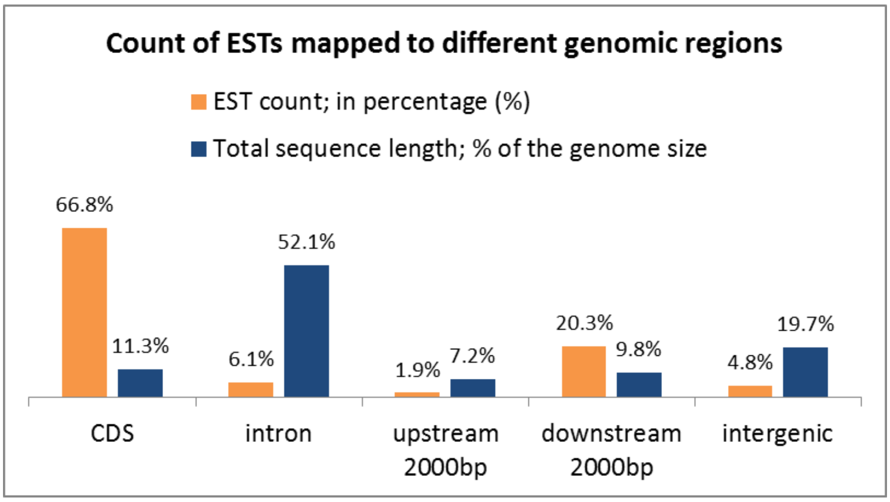

Supplementary Figure 24. Gene counts in different KEGG pathways

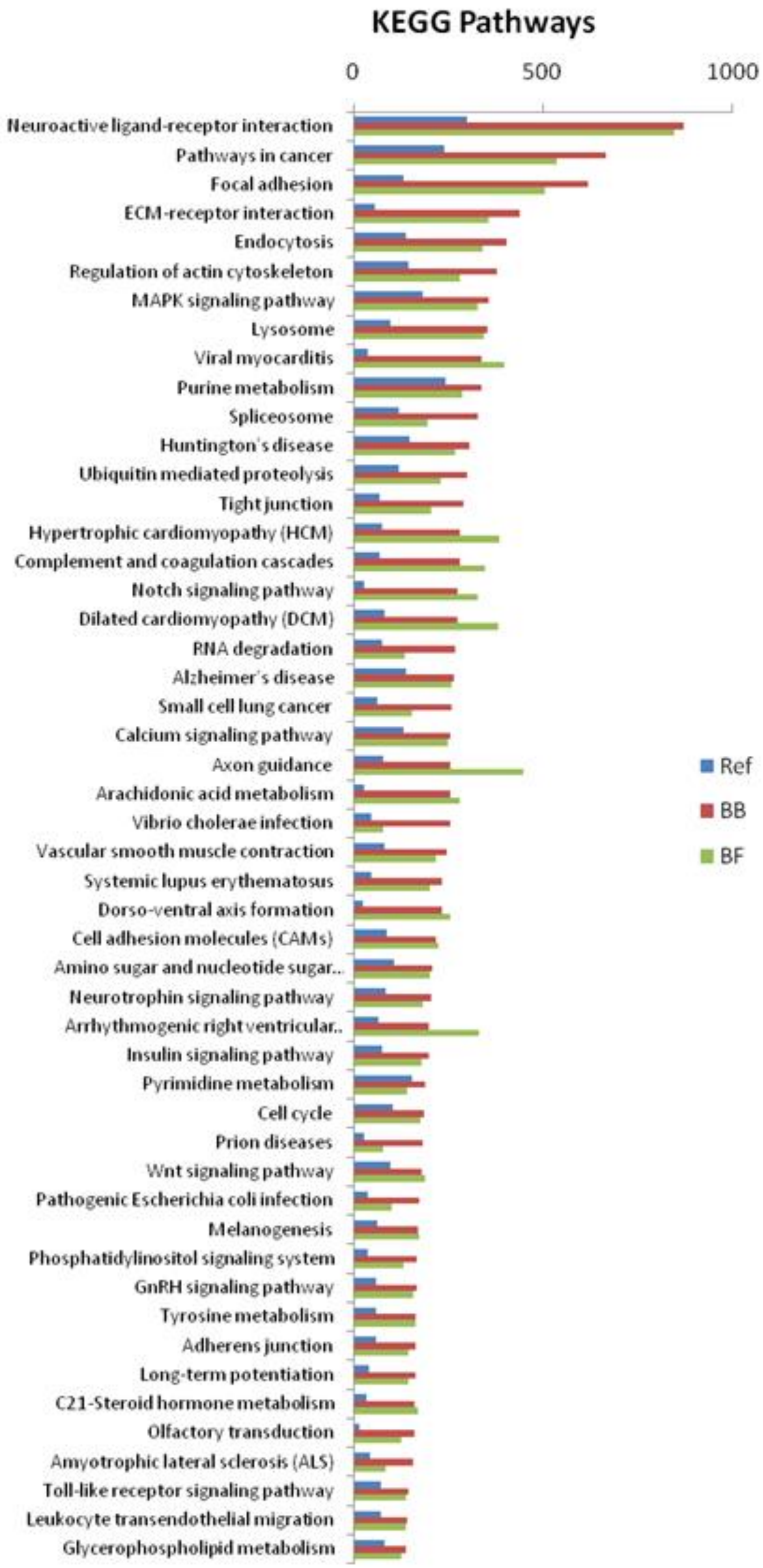

Ref=human; BB=*B. belcheri* haploid assembly V18; BF=*B. floridae* haploid assembly V2.

Supplementary Figure 25. Size comparison of orthologous introns between two lancelet species

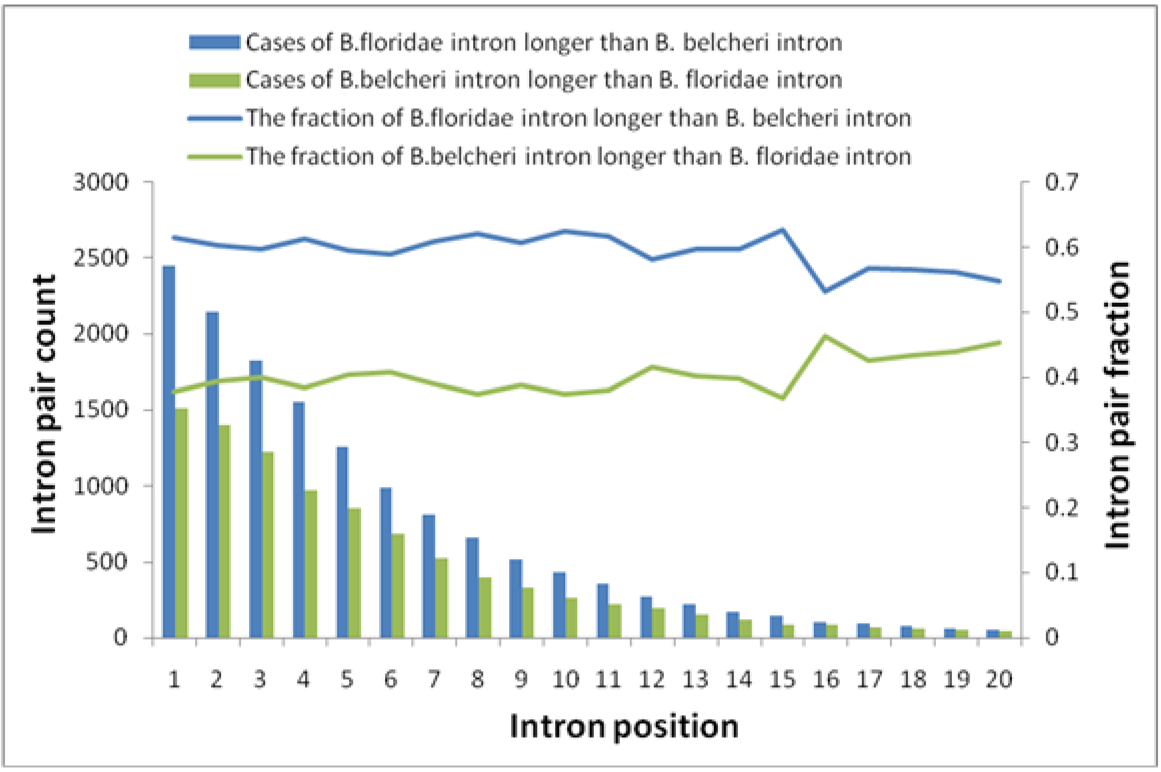

Supplementary Figure 26. Distribution of the methylation level in CpG sequence context.

*There are totally ~31 million CG sites in each lancelet diploid genome assembly, with ~30% of them showing methylation (passed the default filtering of Bis-SNP).

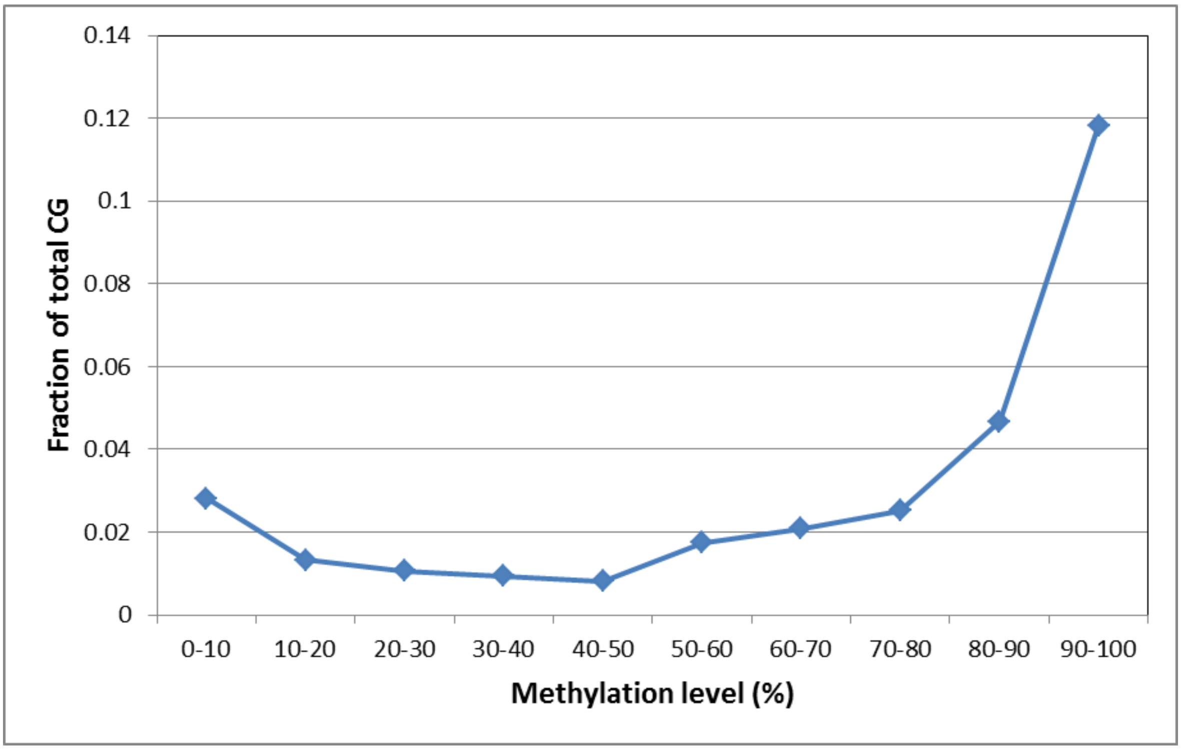

Supplementary Figure 27. Methylation levels (mCG) of different functional regions.

(A) Total methylation level divided by total number of CG sites. CDS=coding DNA sequences; TE=transposable elements, 5-upstream=1500bp 5'-upstream of the first CDS; 3-downstream=1500bp 3'-downstream of the last CDS. ***The difference between any two function regions is extremely significant (*P*<1e-16, t-test).

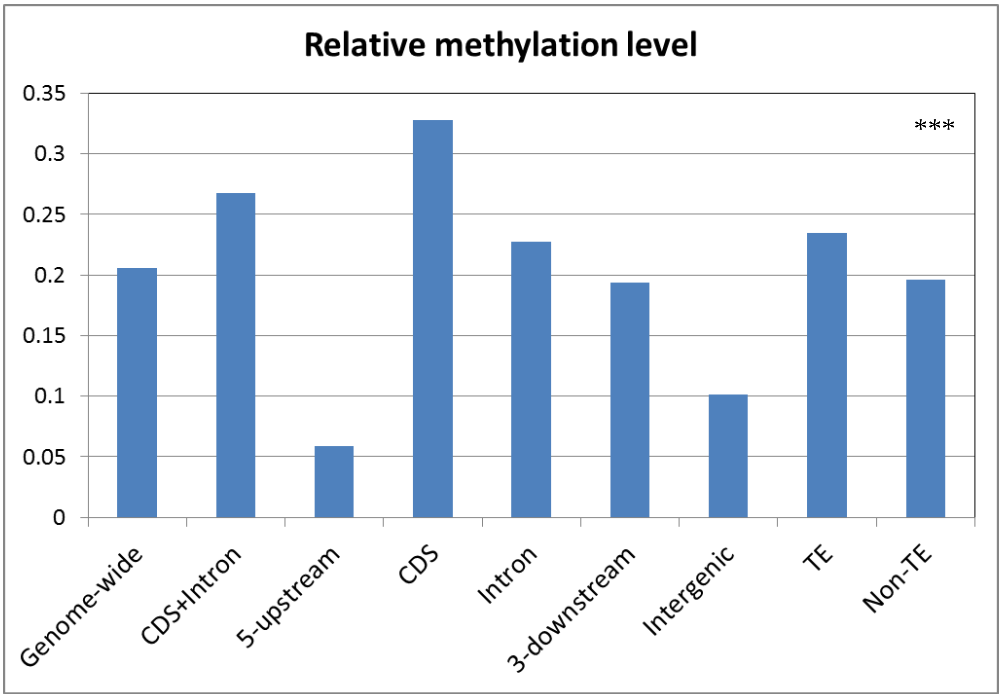

(B) Total methylation level divided by sequence length. CDS=coding DNA sequences; TE=transposable elements, 5-upstream=1500bp 5'-upstream of the first CDS; 3-downstream=1500bp 3'-downstream of the last CDS. ***The difference between any two function regions is extremely significant (*P*<1e-16, t-test).

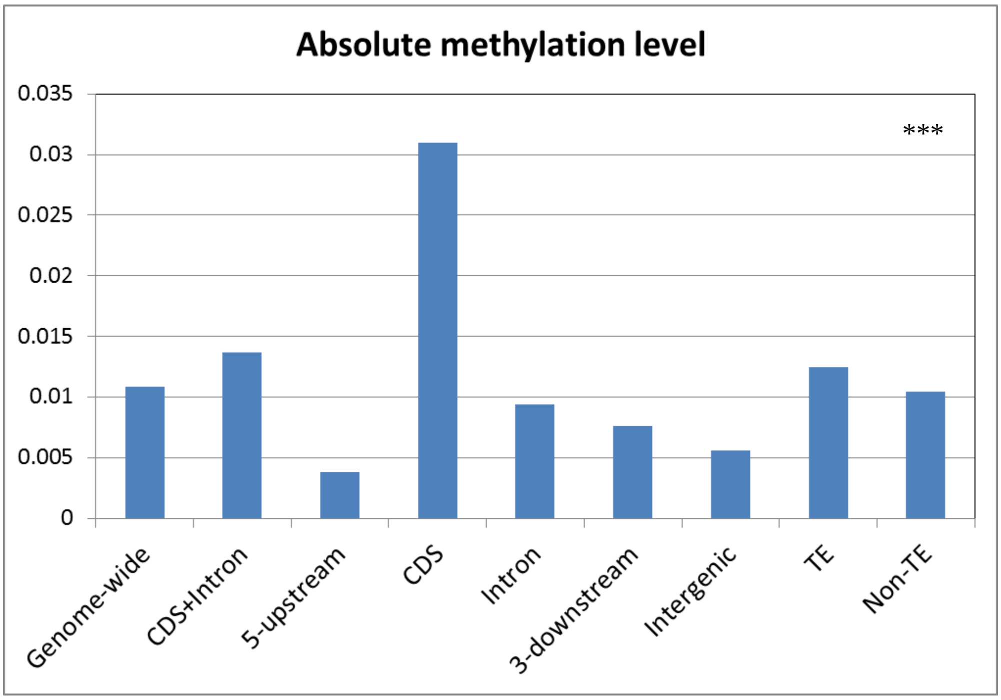

Supplementary Figure 28. Clustering analysis of sequences of all protein domain types

Protein sequences of all domain types from a species are clustered using Blastclust.

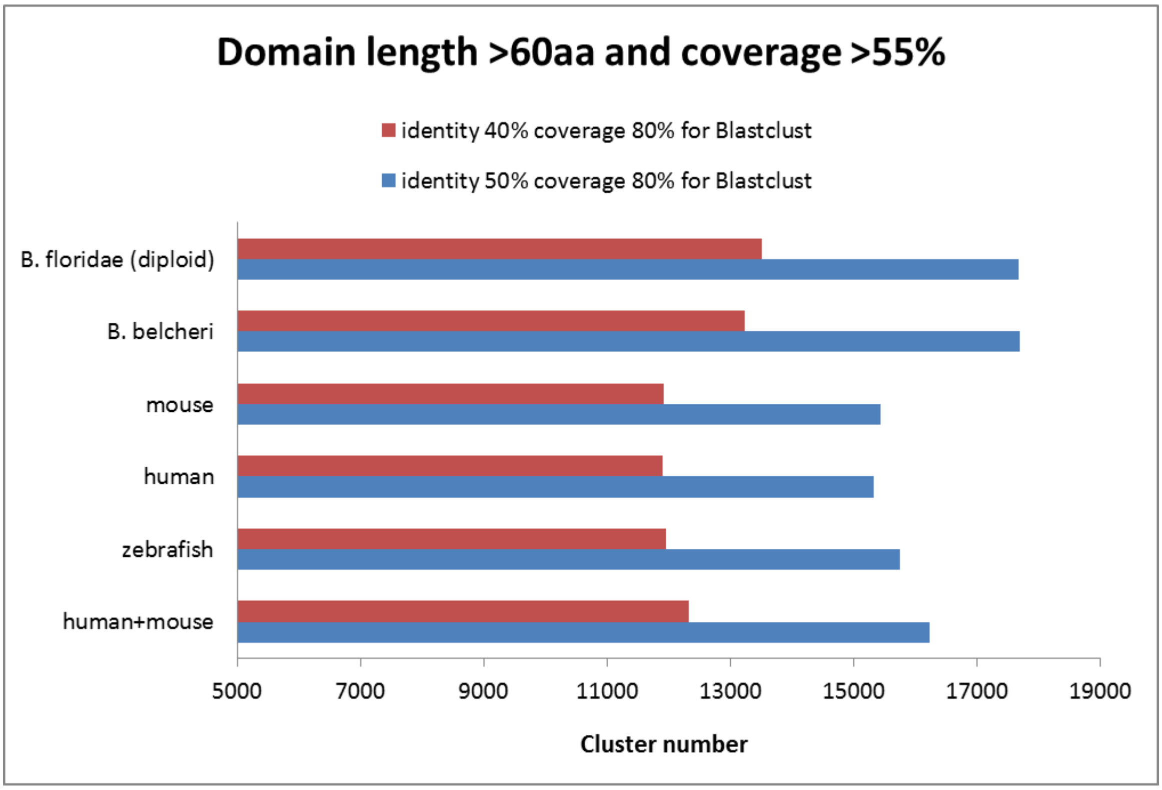

Supplementary Figure 29. Clustering analysis of sequences of ancient domain types

Protein sequences of ancient domain types from a species are clustered using Blastclust.

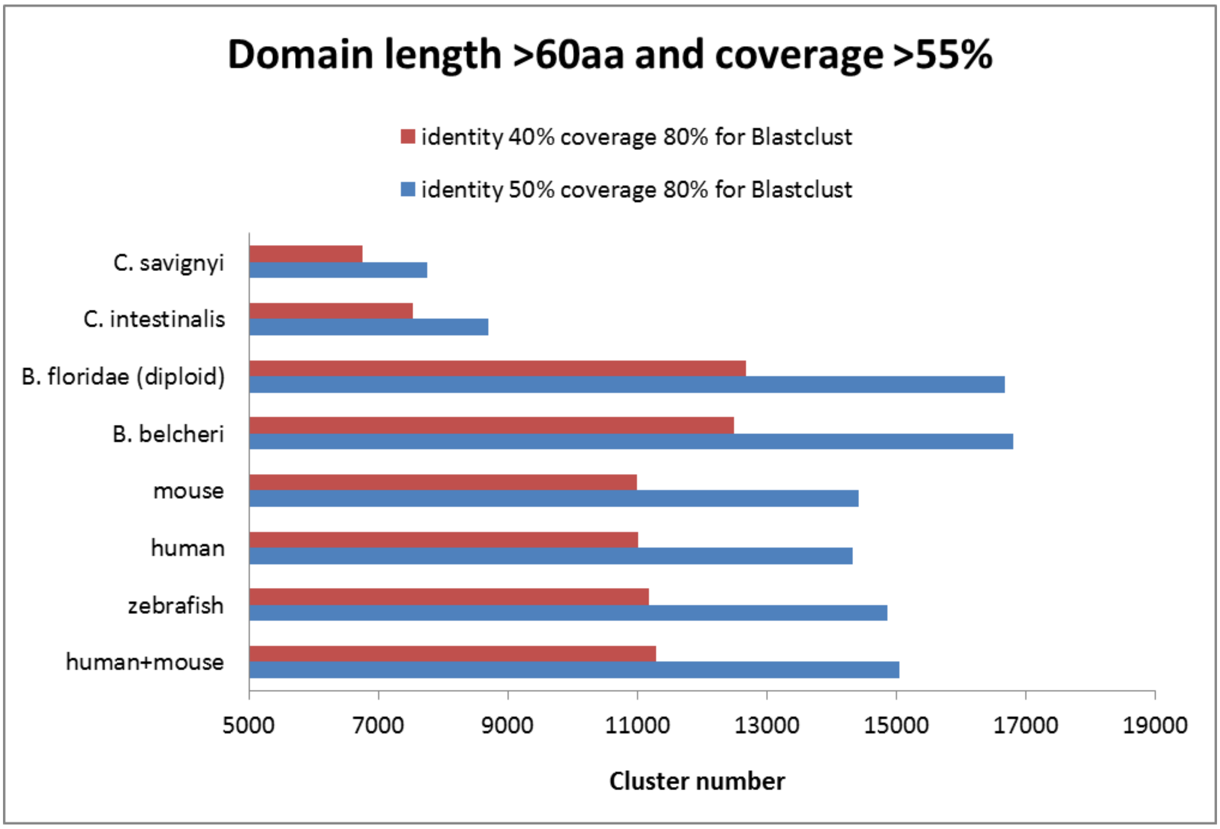

Supplementary Figure 30. The protein architectures related to the 20 longest candidate novel domain families

1. 0121_23_489_a8_b15, 8 instances in *B.belcheri*, average length 489aa.

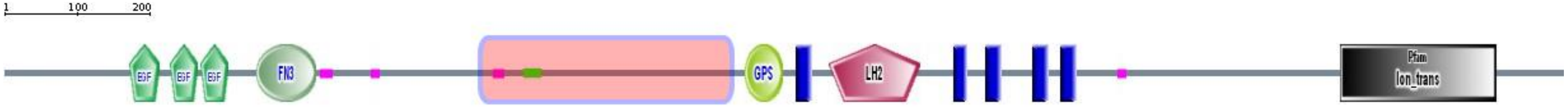

2. 0155_19_542_a8_b11, 8 instances in *B.belcheri*, average length 542aa.

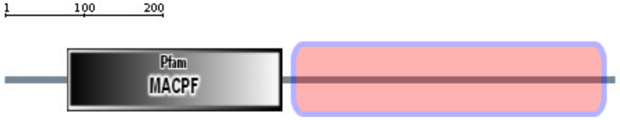

3. 0156_19_646_a6_b13, 6 instances in *B.belcheri*, average length 646aa.

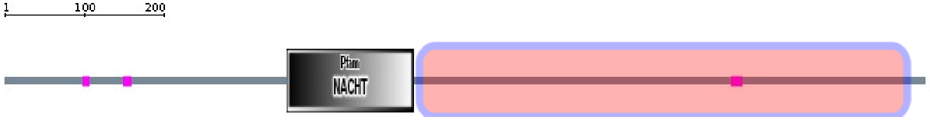

4. 0162_18_488_a11_b7, 11 instances in *B.belcheri*, average length 488aa.

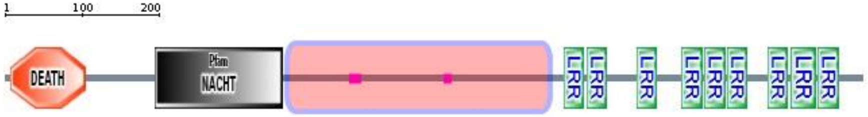

5. 0187_16_519_a3_b13, 3 instances in *B.belcheri*, average length 519aa.

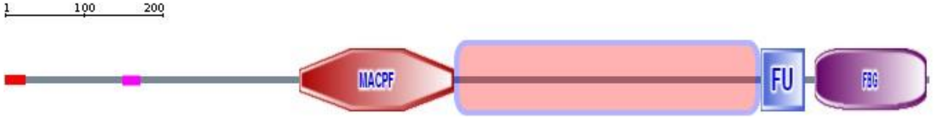

6. 0188_16_371_a4_b12, 4 instances in *B.belcheri*, average length 371aa.

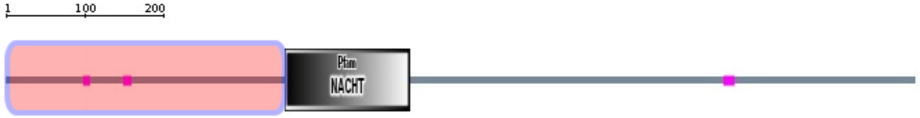

7. 0229_14_427_a5_b9, 5 instances in *B.belcheri*, average length 427aa.

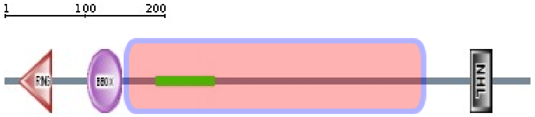

8. 0243_13_348_a4_b9, 4 instances in *B.belcheri*, average length 348aa.

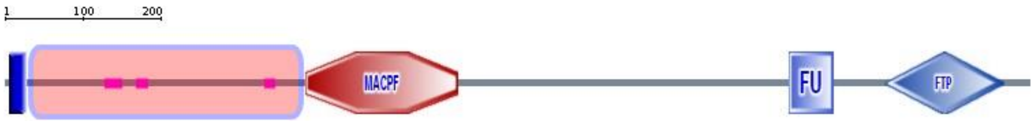

9. 0300_11_494_a2_b9, 2 instances in *B.belcheri*, average length 494aa.

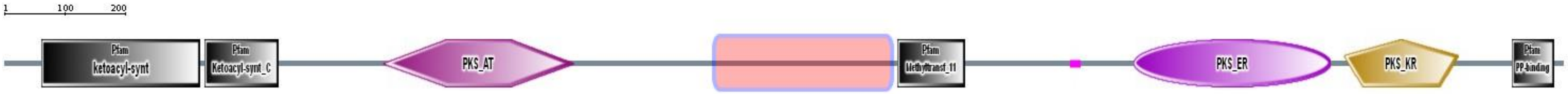

10. 0304_11_304_a3_b8_merge, 3 instances in *B.belcheri*, average length 304aa.

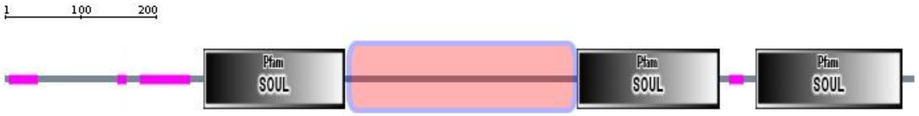

11. 0306_11_287_a2_b9, 2 instances in *B.belcheri*, average length 287aa.

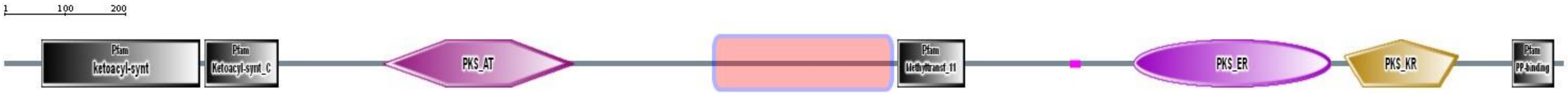

12. 0348_10_706_a5_b5, 5 instances in *B.belcheri*, average length 706aa.

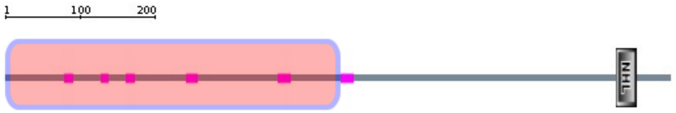

13. 0391_9_529_a5_b4, 5 instances in *B.belcheri*, average length 529aa.

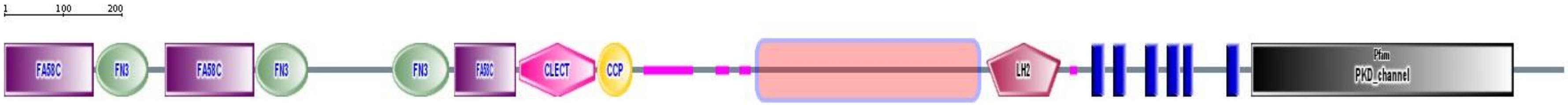

14. 0392_9_543_a2_b7, 2 instances in *B.belcheri*, average length 543aa.

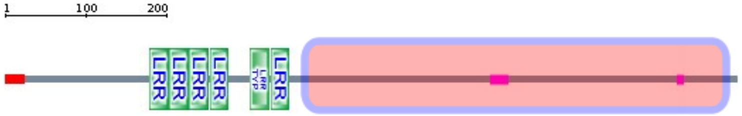

15. 0466_8_373_a3_b5, 3 instances in *B.belcheri*, average length 373aa.

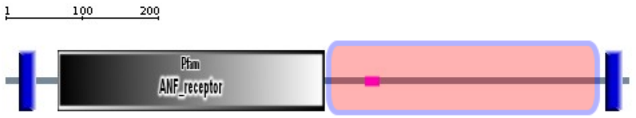

16. 0552_7_487_a3_b4, 3 instances in *B.belcheri*, average length 487aa.

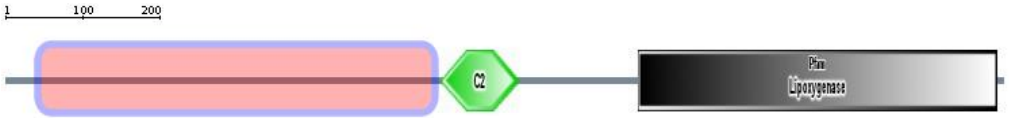

17. 0646_6_427_a2_b4, 2 instances in *B.belcheri*, average length 427aa.

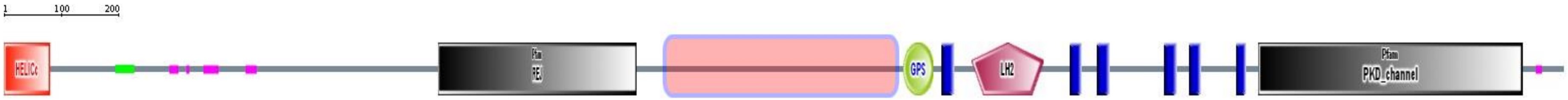

18. 0649_6_429_a2_b4, 2 instances in *B.belcheri*, average length 429aa.

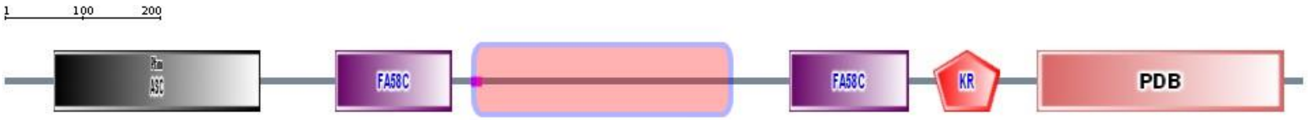

19. 0654_6_338_a2_b4, 2 instances in *B.belcheri*, average length 338aa.

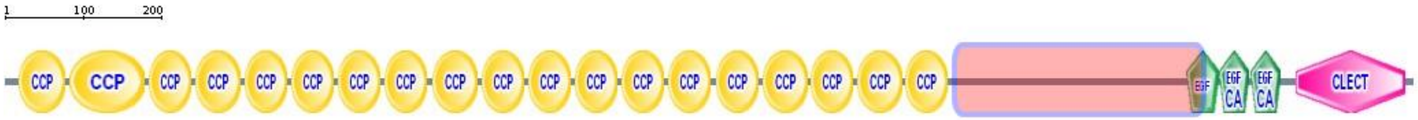

20. 0804_5_578_a2_b3, 2 instances in *B.belcheri*, average length 578aa.

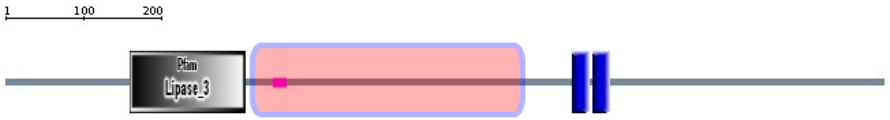

Supplementary Figure 31. The protein architectures related to the 10 largest candidate novel domain families

1. 0013_81_33_a10_b71, 10 instances in *B.belcheri*, average length 33 aa.

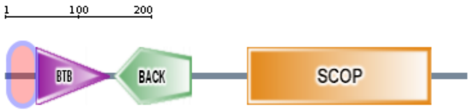

2. 0019_71_224_a47_b24, 47 instances in *B.belcheri*, average length 224 aa.

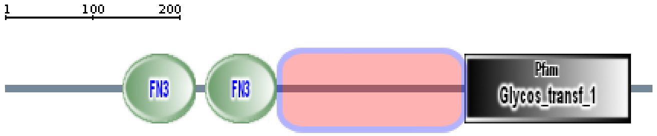

3. 0034_52_153_a28_b24, 28 instances in *B.belcheri*, average length 153 aa.

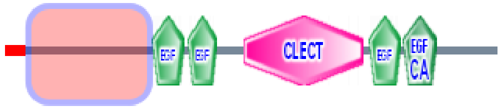

4. 0056_40_101_a18_b22, 18 instances in *B.belcheri*, average length 101 aa.

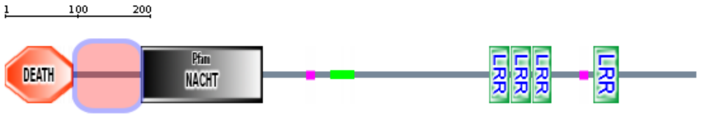

5. 0062_37_73_a15_b22, 15 instances in *B.belcheri*, average length 73 aa.

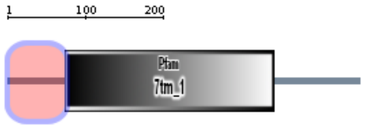

6. 0076_33_55_a10_b23, 10 instances in *B.belcheri*, average length 55 aa.

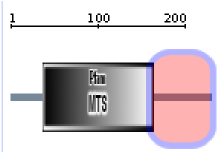

7. 0084_31_58_a13_b18, 13 instances in *B.belcheri*, average length 58 aa.

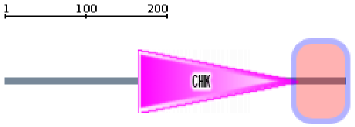

8. 0091_28_384_a10_b18, 10 instances in *B.belcheri*, average length 384 aa.

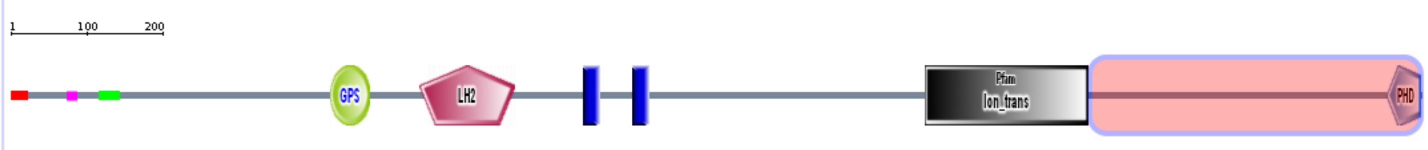

9. 0117_24_69_a13_b11, 13 instances in *B.belcheri*, average length 69 aa.

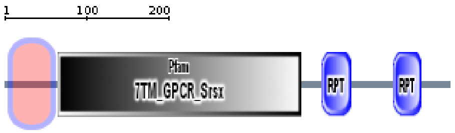

10. 0147_21_64_a11_b10, 11 instances in *B.belcheri*, average length 64 aa.

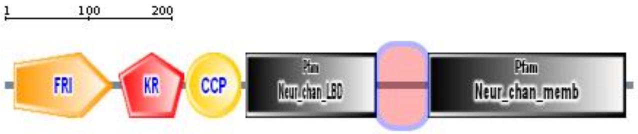

Supplementary Figure 32. The repertoire of putative immune-related gene families.

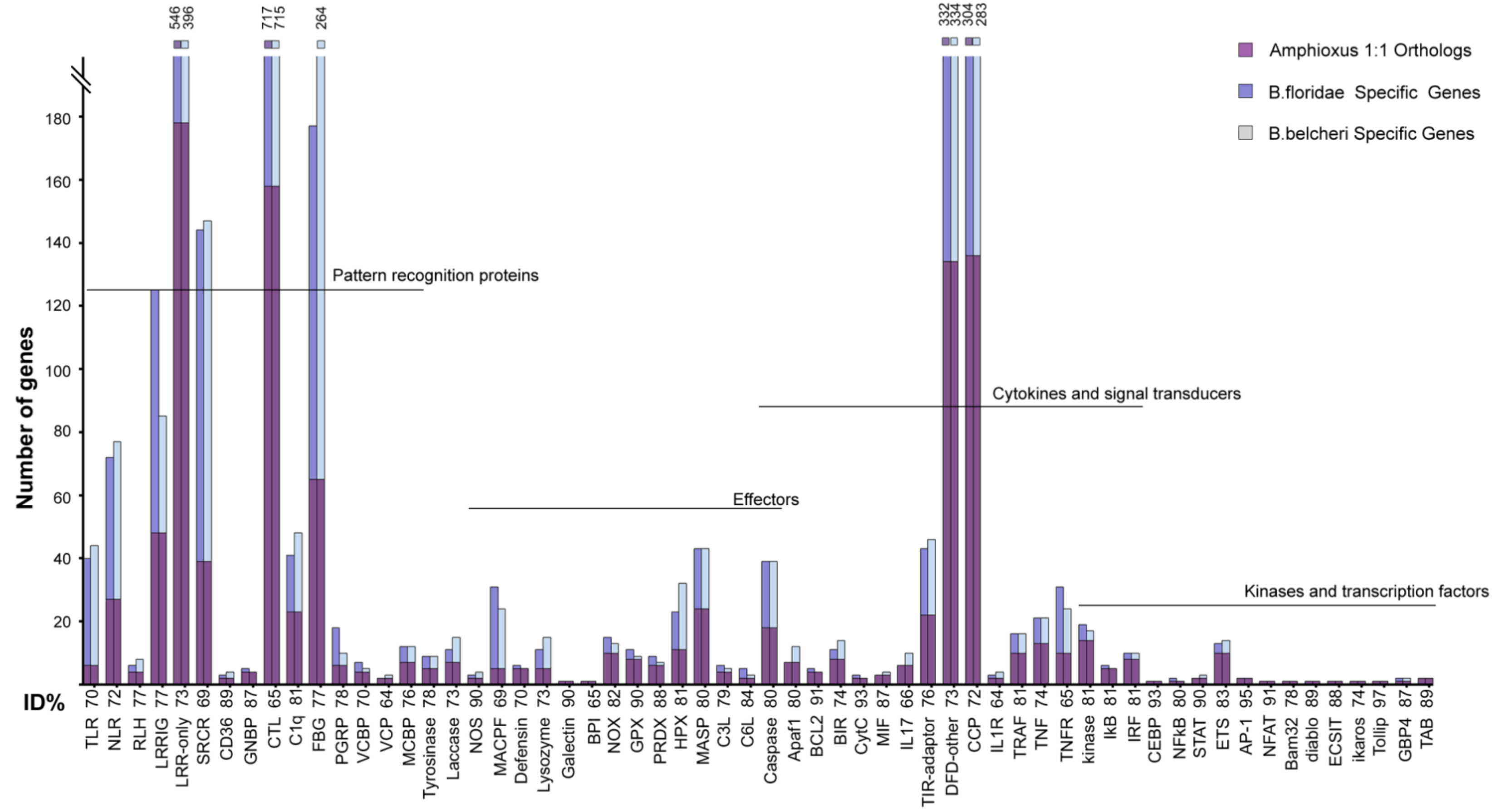

Note: the ID% line shows the average sequence identity between orthologous protein pairs.

Supplementary Figure 33. Phylogenetic reconstruction (using the minimum evolution method) based on domain combinations

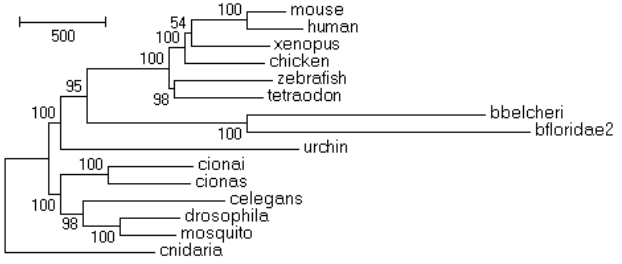

Evalue=1, two-domain combination

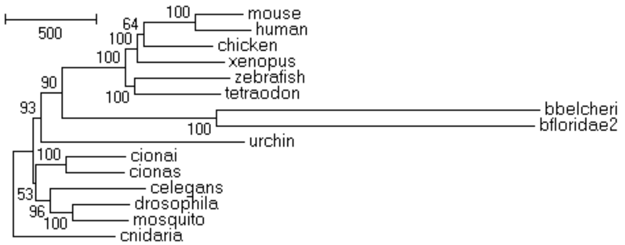

Evalue=1, three-domain combination

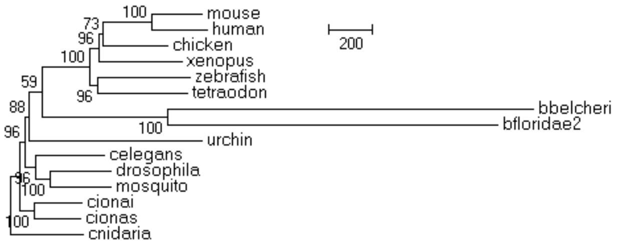

Evalue=1, four-domain combination

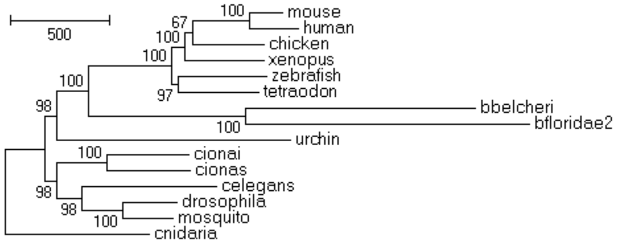

Evalue=1e-5, two-domain combination

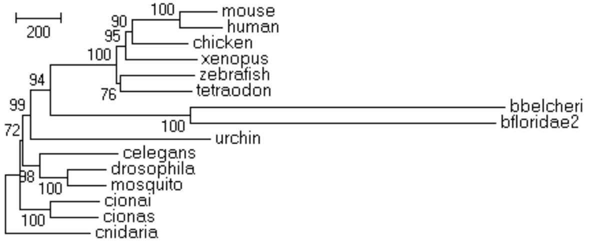

Evalue=1e-5, three-domain combination

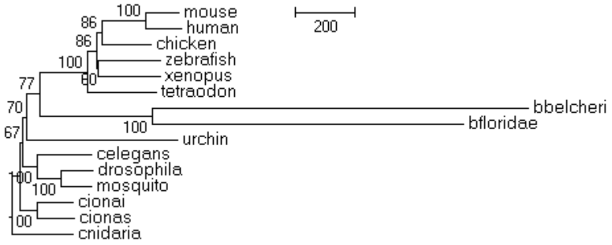

Evalue=1e-5, four-domain combination

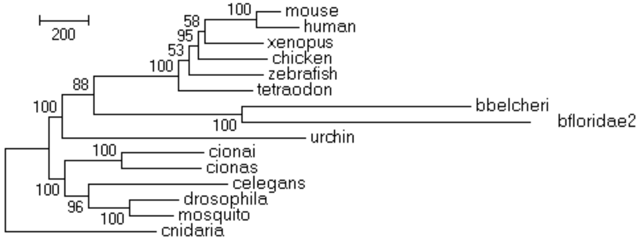

Evalue=1, two-domain combination, clan mode

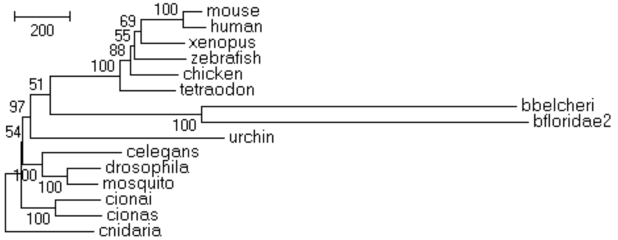

Evalue=1, three-domain combination, clan mode

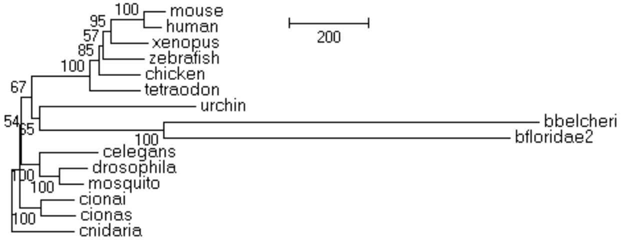

Evalue=1, four-domain combination, clan mode

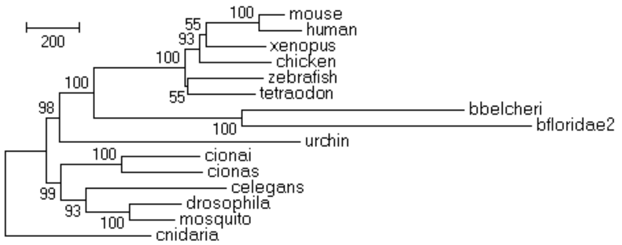

Evalue=1e-5, two-domain combination, clan mode

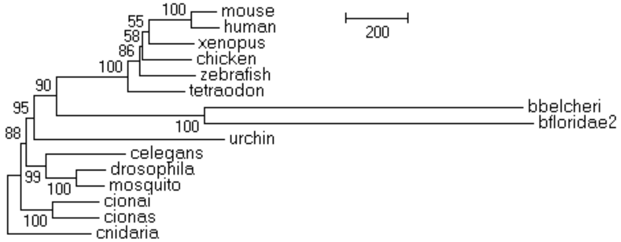

Evalue=1e-5, three-domain combination, clan mode

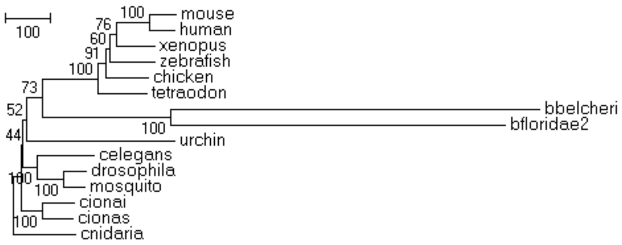

Evalue=1e-5, four-domain combination, clan mode

Supplementary Figure 34. The turnover rates of domain combinations in different species (based on the maximal likelihood method)

The presence and absence of domain combinations are superimposed on the known species tree. The branch length is estimated using PAML, ML method, model JC69.

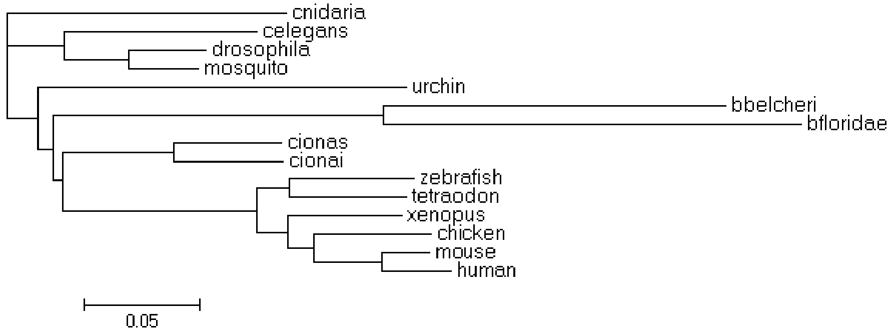

Evalue=1, two-domain combination

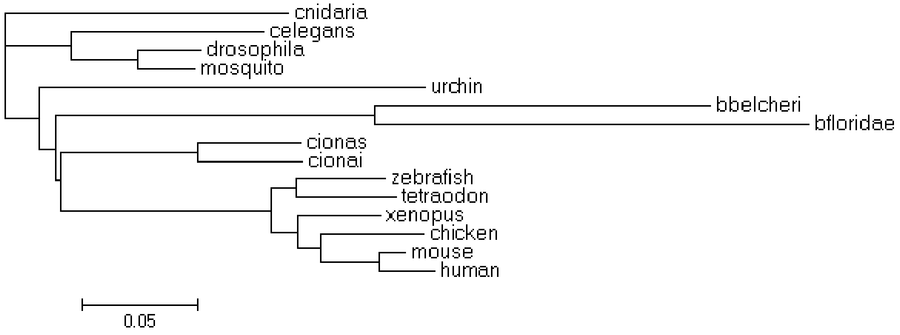

Evalue=1, two-domain combination, clan mode

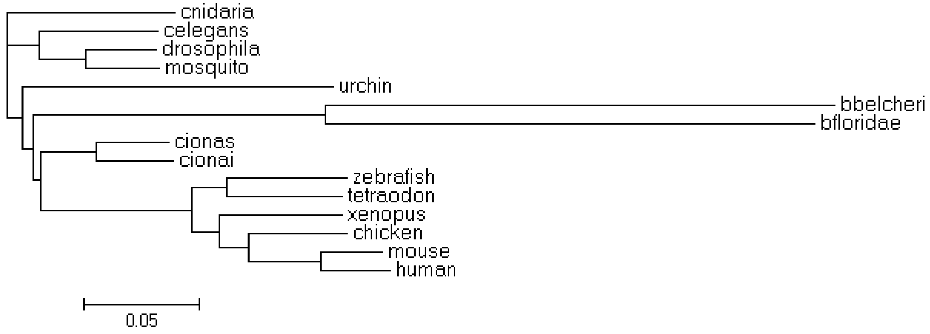

Evalue=1, three-domain combination

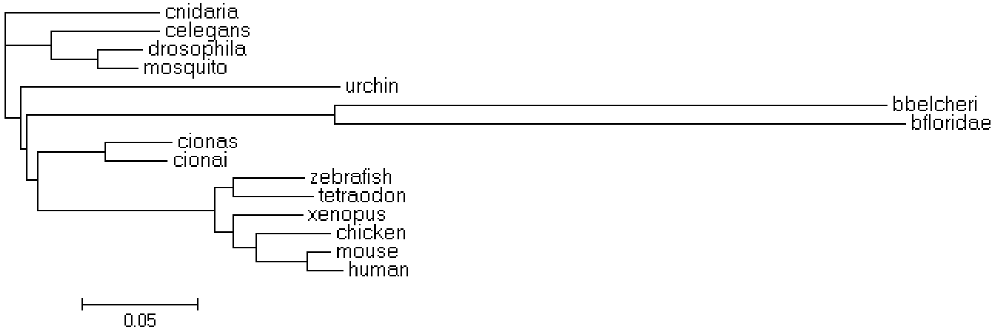

Evalue=1, three-domain combination, clan mode

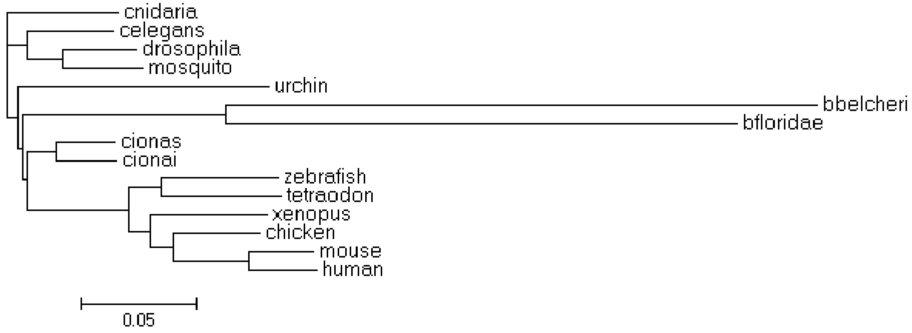

Evalue=1, four-domain combination

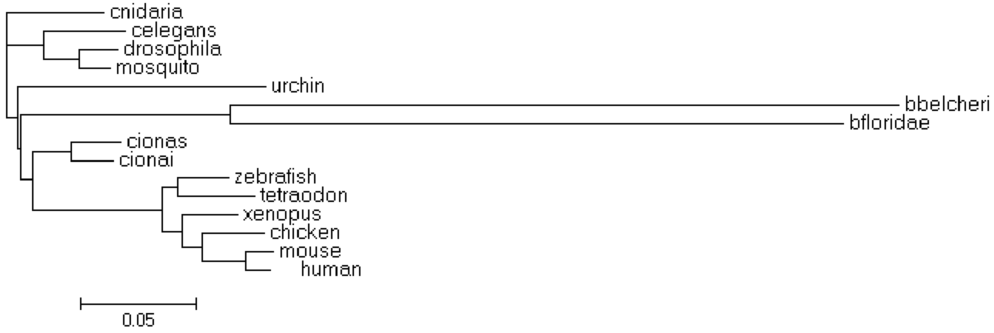

Evalue=1, four-domain combination, clan mode

Supplementary Figure 35. The numbers of novel domain combinations on different lineages.

The numbers of novel domain combinations are superimposed on the known species tree.

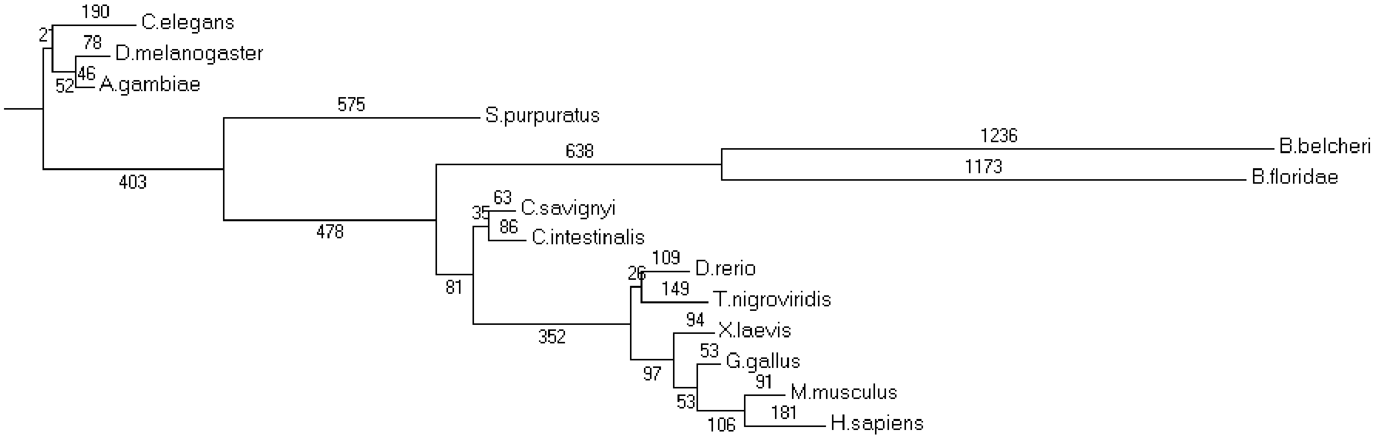

A. Evalue=1, two-domain combination, including vertebrate-specific domain types

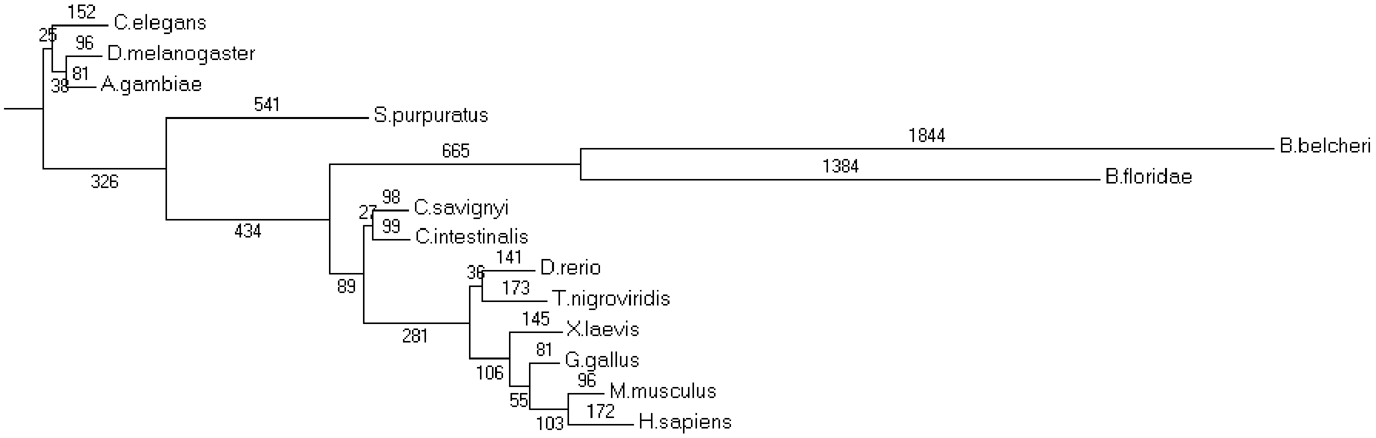

B. Evalue=1, three-domain combination, including vertebrate-specific domain types

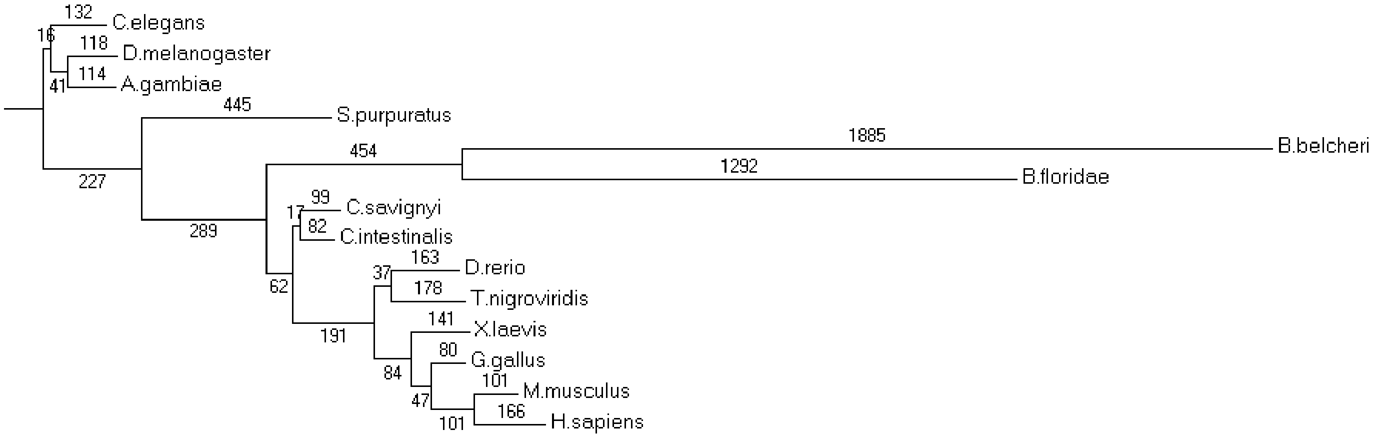

C. Evalue=1, four-domain combination, including vertebrate-specific domain types

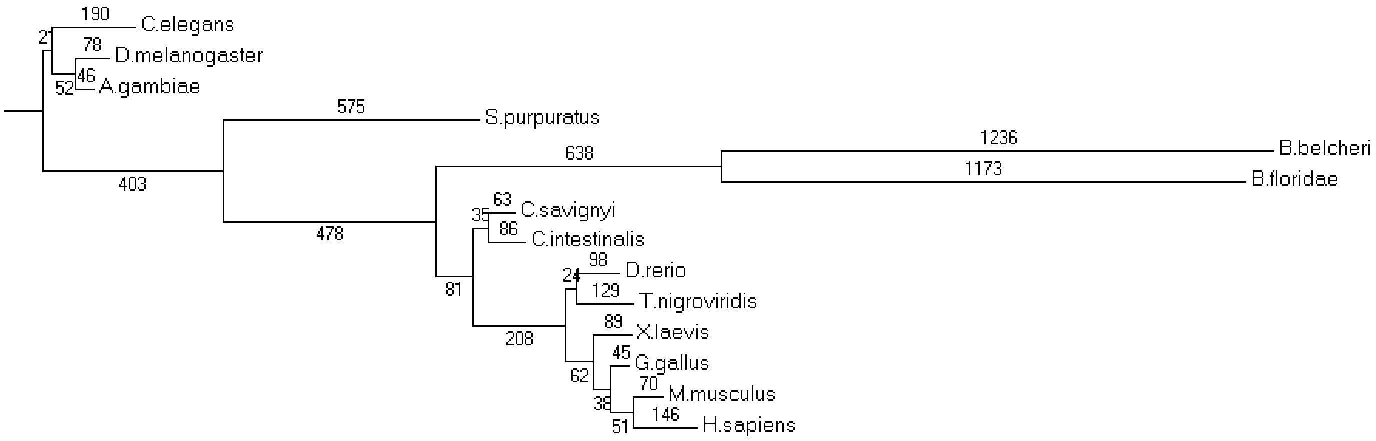

D. Evalue=1, two-domain combination, without vertebrate-specific domain types

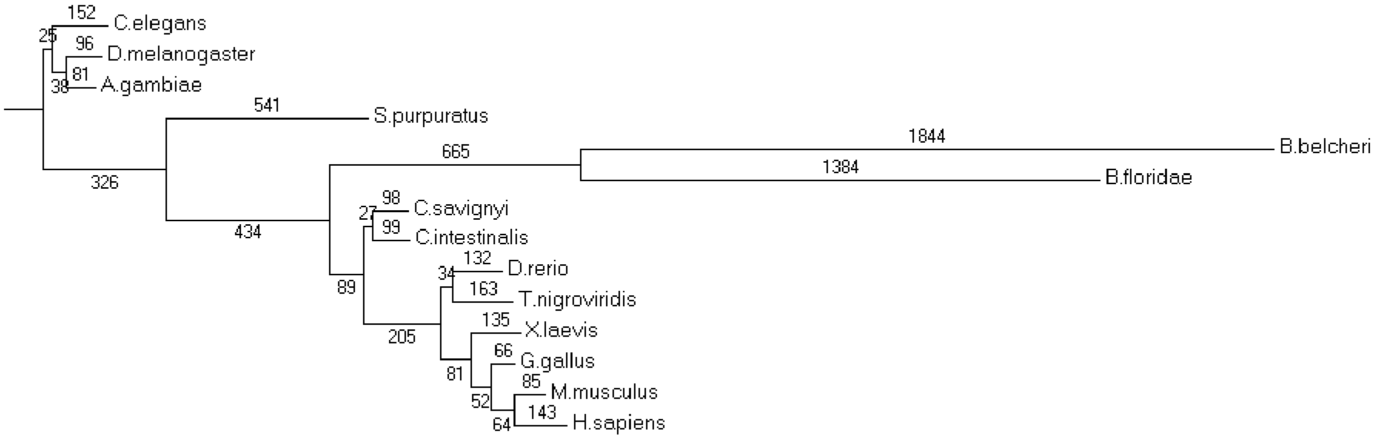

E. Evalue=1, three-domain combination, without vertebrate-specific domain types

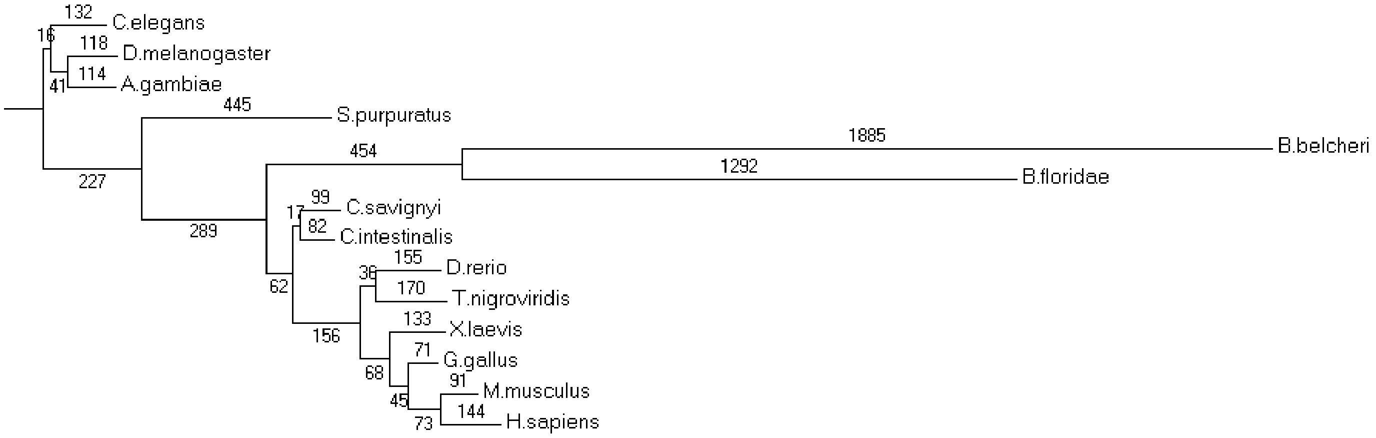

F. Evalue=1, four-domain combination, without vertebrate-specific domain types

Supplementary Figure 36. The proportion of immune-related domains in novel domain pairs

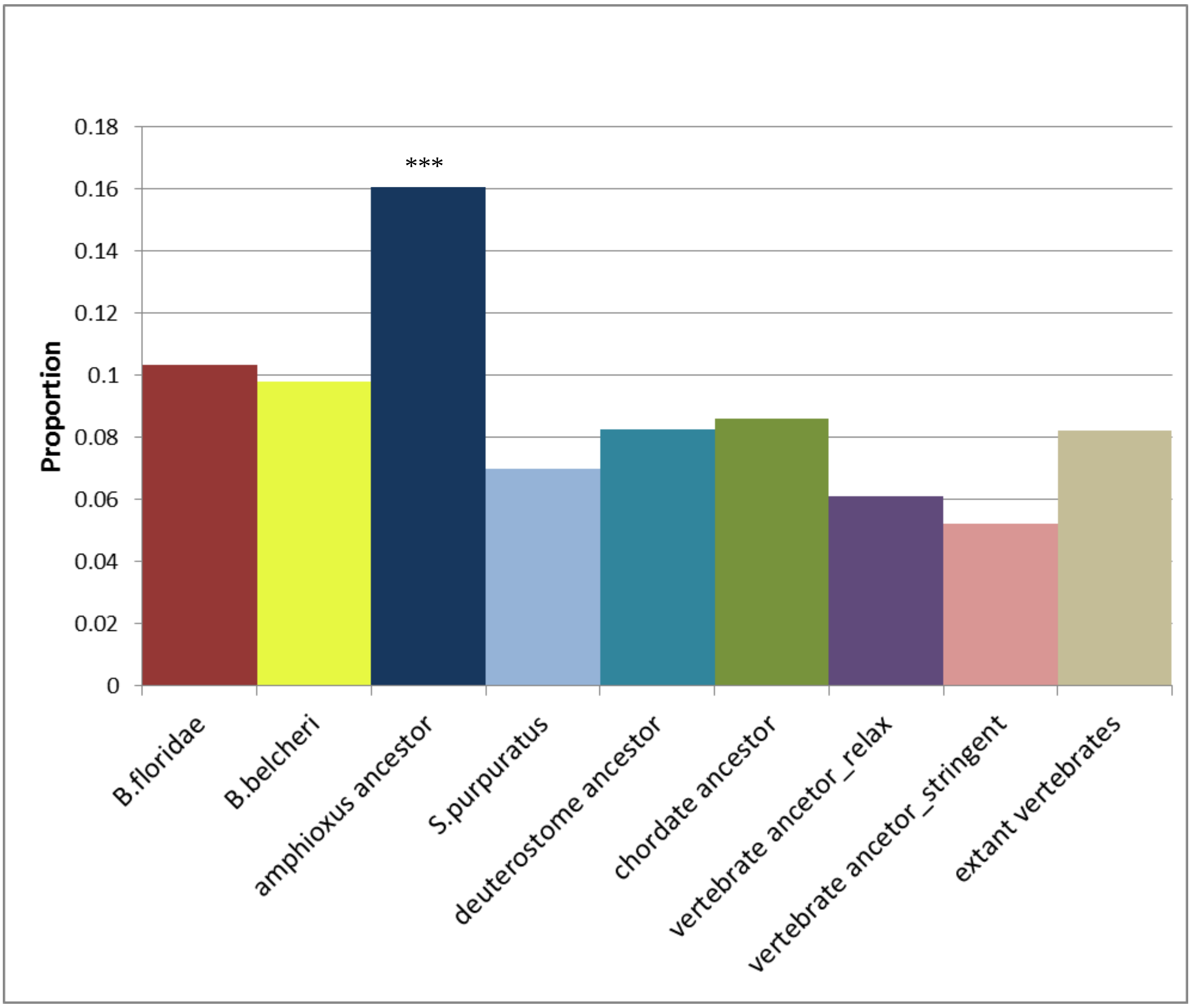

Note: see Supplementary Table 23 for the meaning of the lineage names.

***The proportion is significant higher than other lineages (*p*<1e-16, chi-square test).

Supplementary Figure 37. The most used immune-related domains in novel domain pairs

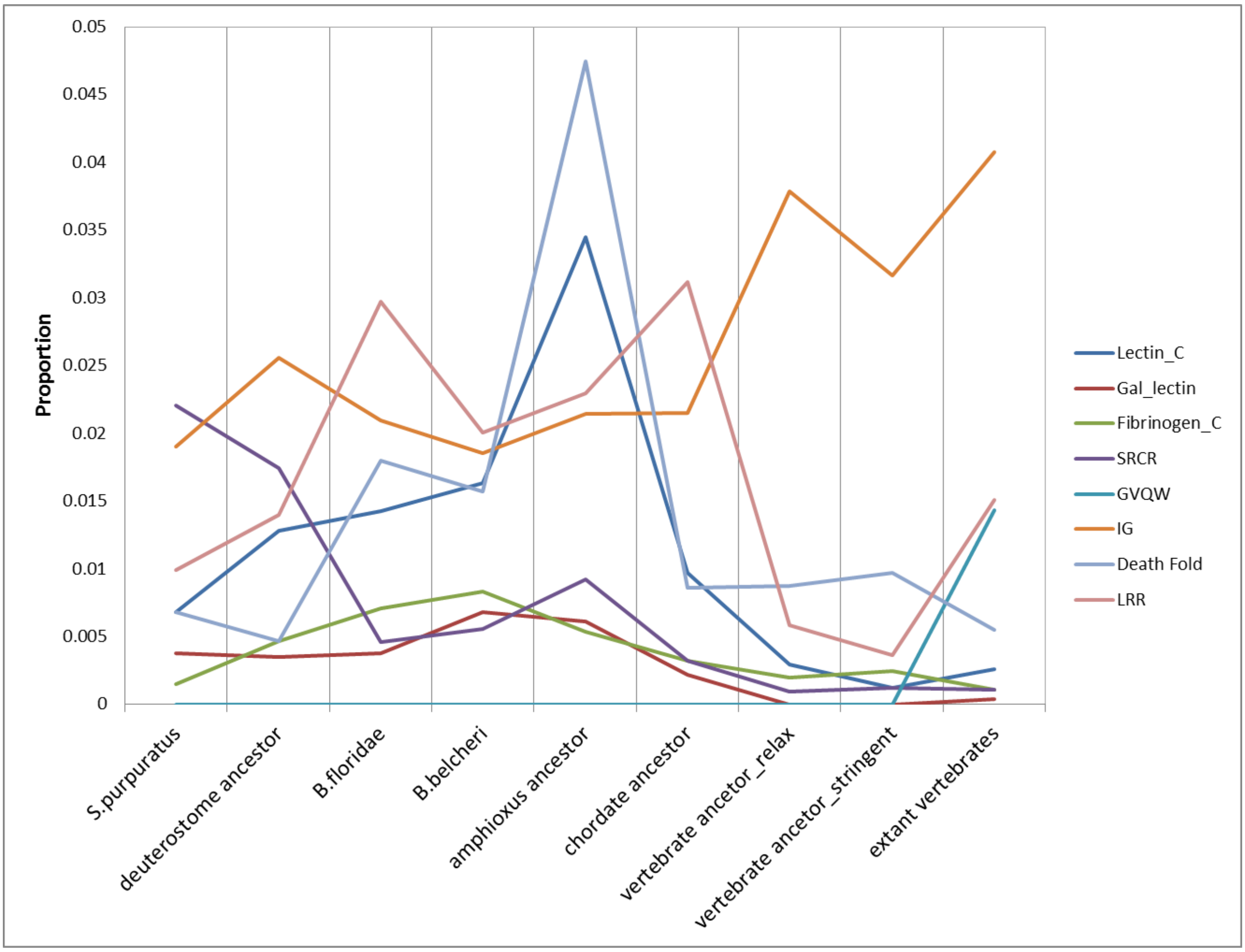

Note: see Supplementary Table 23 for the meaning of the lineage names.

Supplementary Figure 38. Molecular functions for top 50 promiscuous domains

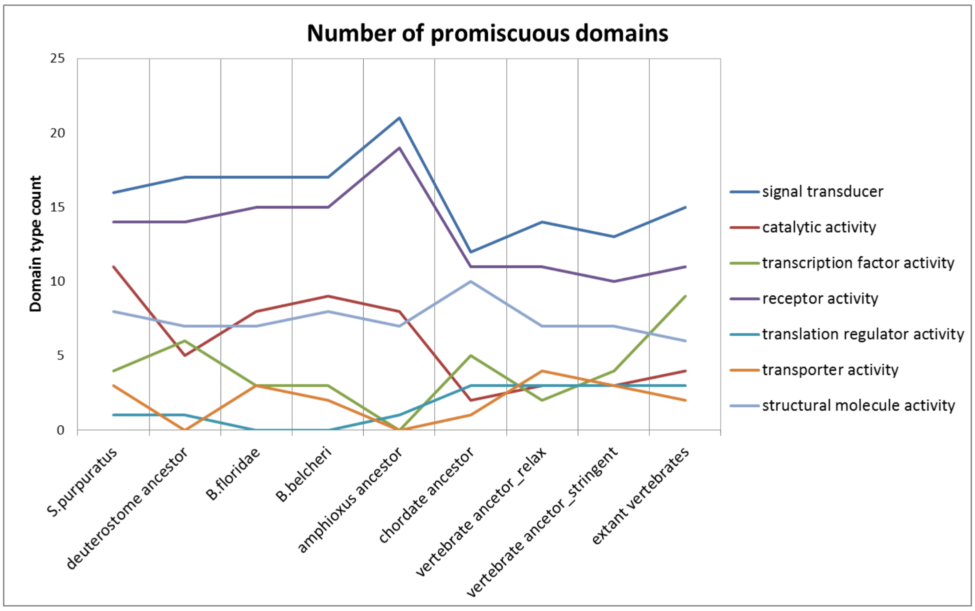

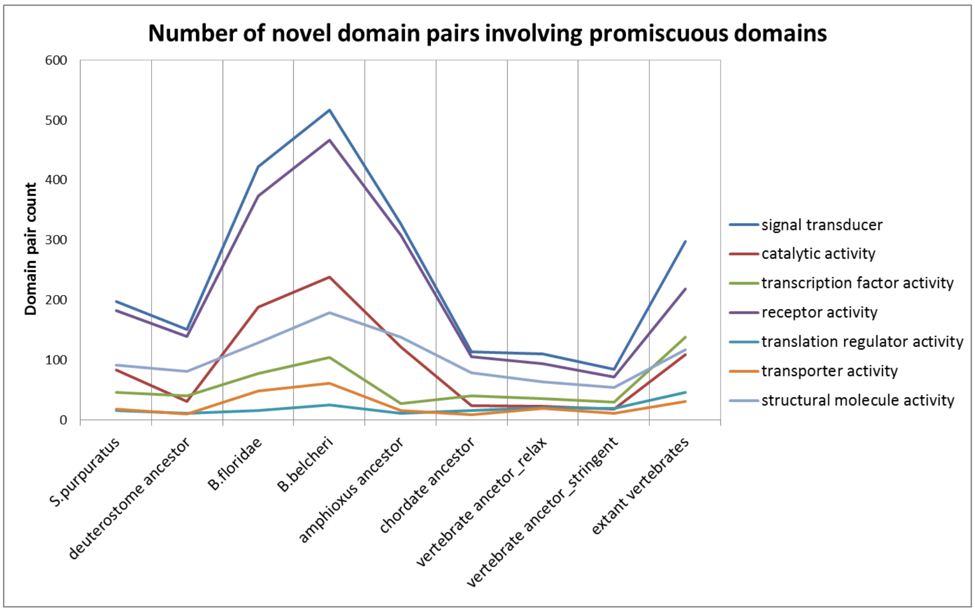

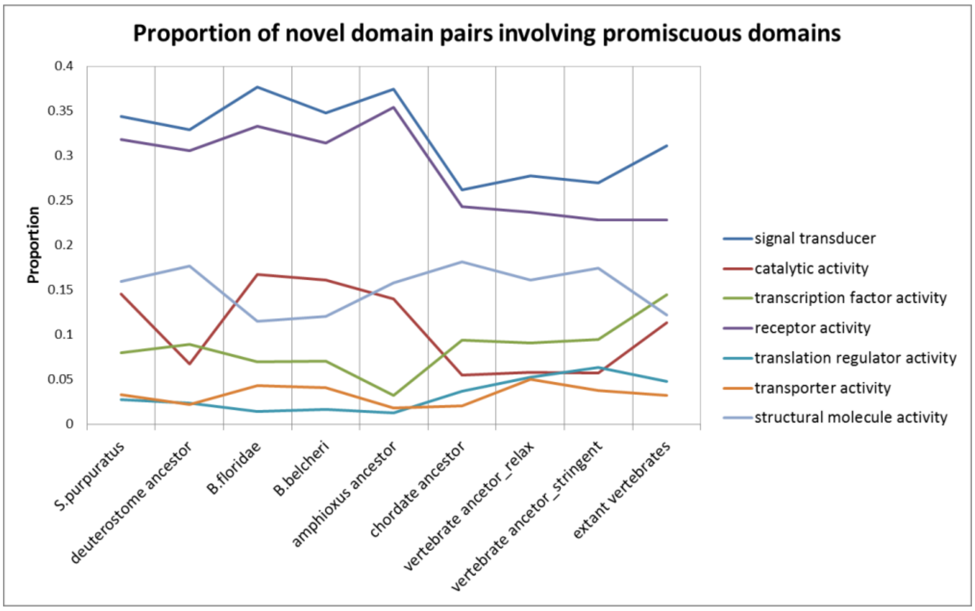

Note: see Supplementary Table 23 for the meaning of the lineage names.

Supplementary Figure 39. Cellular locations for top 50 promiscuous domains

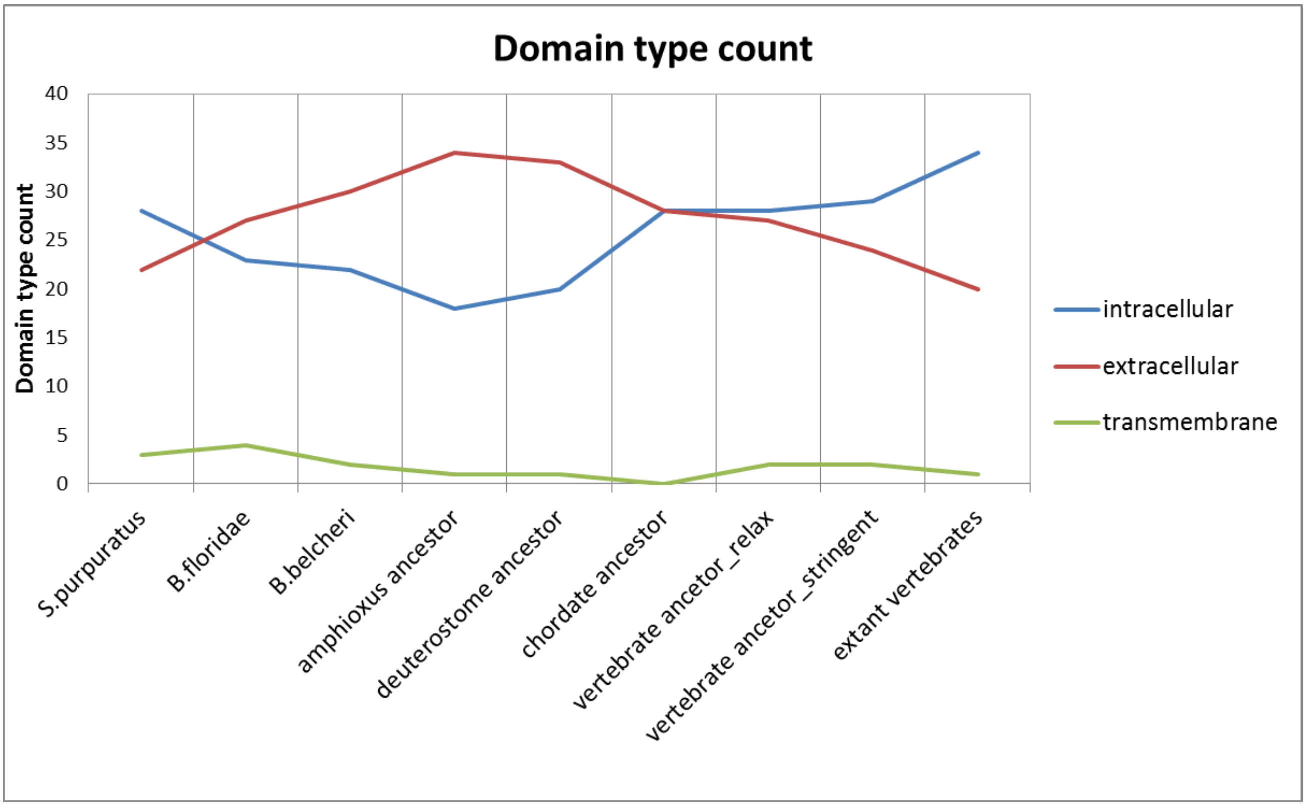

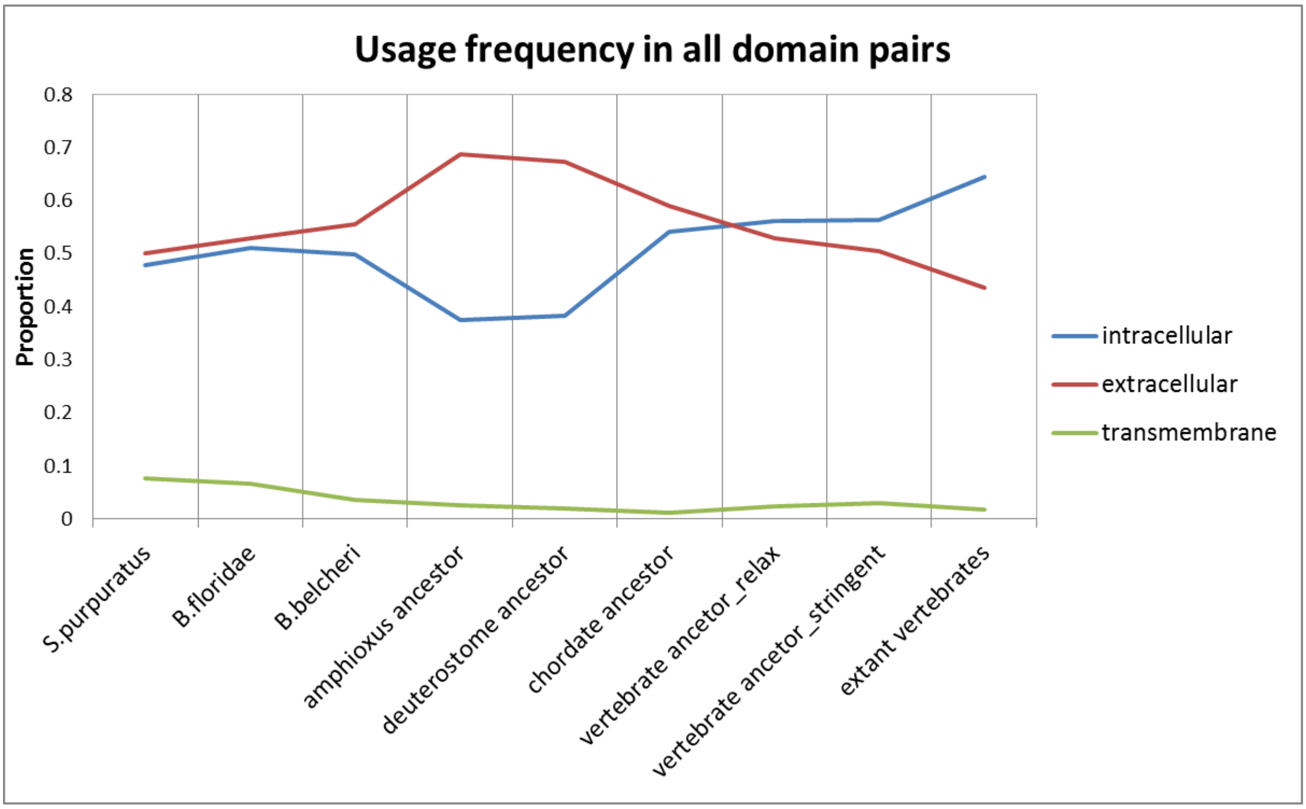

Note: see Supplementary Table 23 for the meaning of the lineage names.

Supplementary Figure 40. Approximate estimation of relative DCJ distances contributed by exon-level rearrangements

Note that only coding exons (or coding DNA sequences (CDS)) are used for this analysis.

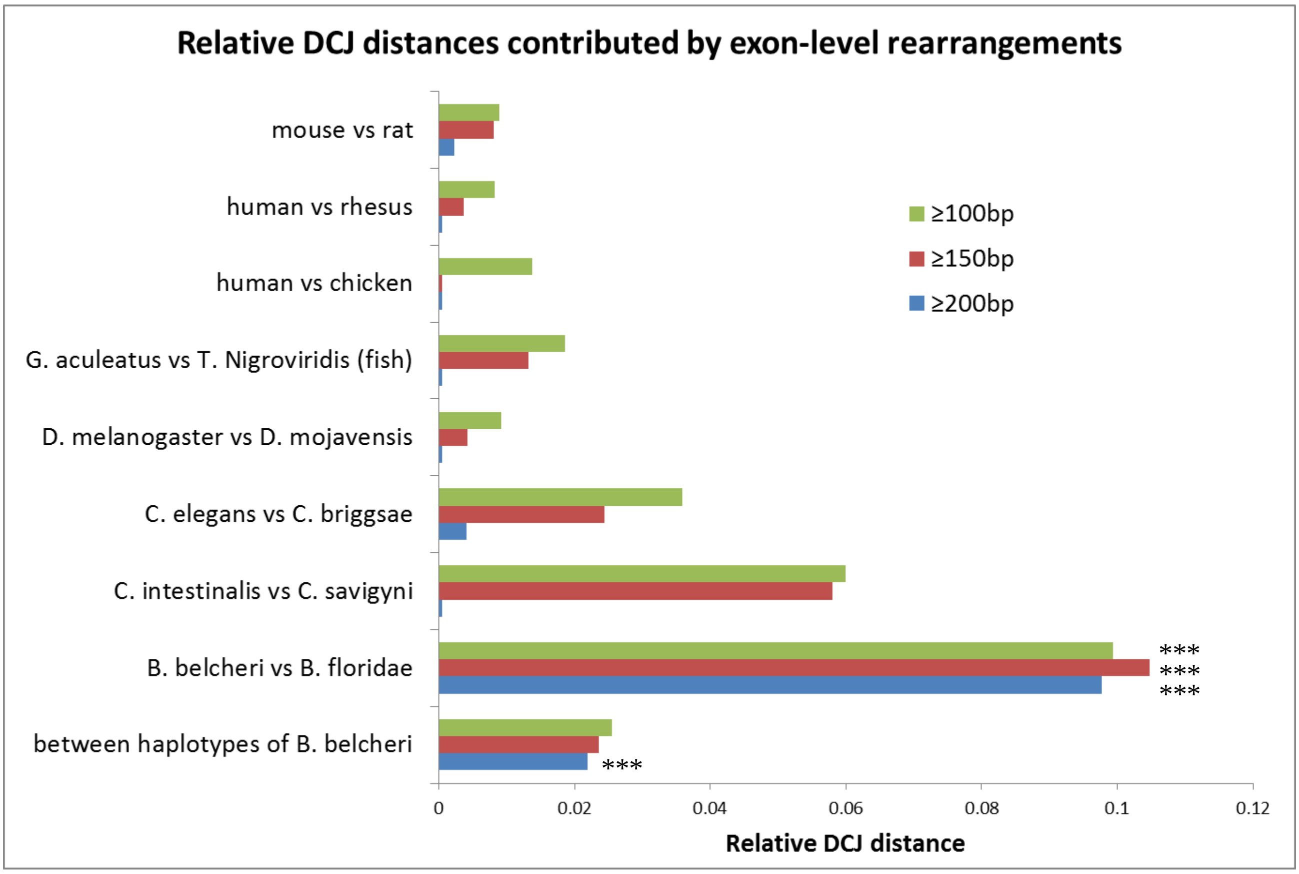

*** indicates significant (p<1e-16, chi-square test) difference for comparisons between lancelets and other species pairs.

Supplementary Figure 41. The fraction of three symmetrical phases for internal exons in different species

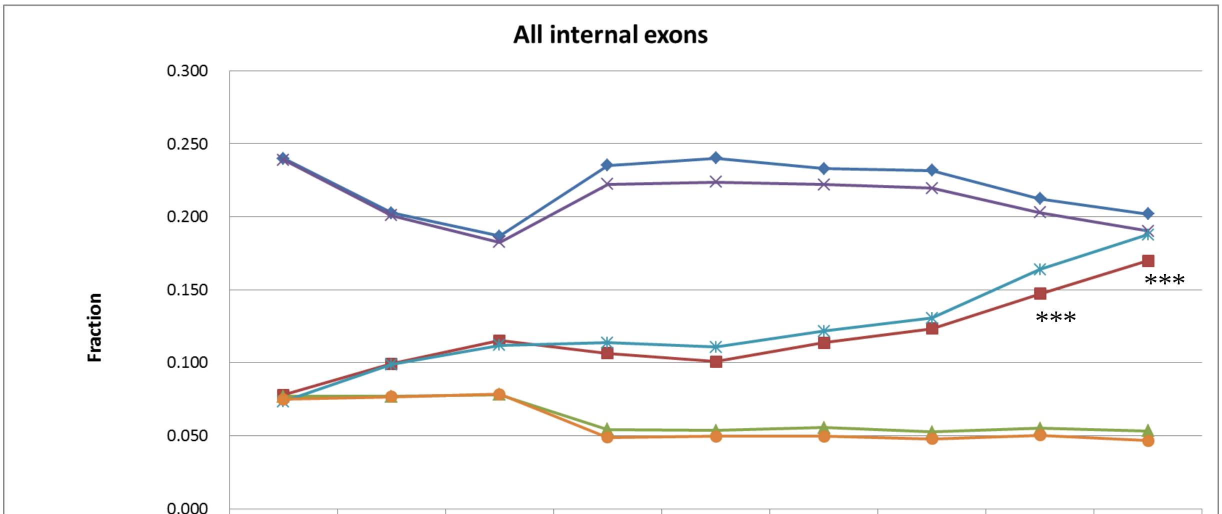

	C. elegans	C. instestinalis	D. melanogaster	stickleback	tetraodon	chicken	human	B. floridae	B. belcheri
phase 0-0; all size	0.240	0.203	0.187	0.235	0.240	0.233	0.232	0.212	0.202
phase 1-1; all size	0.078	0.099	0.115	0.106	0.101	0.114	0.123	0.147	0.170
phase 2-2; all size	0.077	0.077	0.078	0.054	0.054	0.055	0.053	0.055	0.053
phase 0-0; >100bp	0.239	0.201	0.182	0.222	0.224	0.222	0.220	0.203	0.190
phase 1-1; >100bp	0.073	0.099	0.112	0.114	0.111	0.122	0.131	0.164	0.188
phase 2-2; >100bp	0.075	0.077	0.078	0.049	0.050	0.049	0.048	0.050	0.047

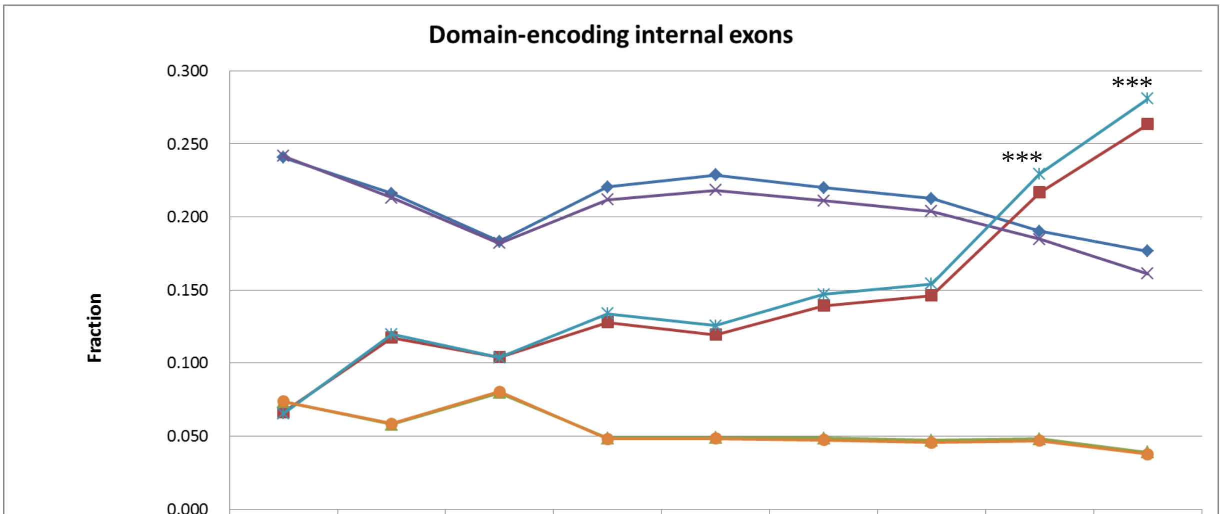

	C. elegans	C. instestinalis	D. melanogaster	stickleback	tetraodon	chicken	human	B. floridae	B. belcheri
phase 0-0; all size	0.241	0.216	0.183	0.220	0.229	0.220	0.213	0.190	0.177
phase 1-1; all size	0.066	0.117	0.104	0.128	0.119	0.139	0.146	0.217	0.263
phase 2-2; all size	0.074	0.058	0.080	0.049	0.049	0.049	0.047	0.048	0.039
phase 0-0; >100bp	0.242	0.213	0.182	0.212	0.218	0.211	0.204	0.185	0.161
phase 1-1; >100bp	0.065	0.120	0.104	0.134	0.126	0.147	0.154	0.229	0.281
phase 2-2; >100bp	0.074	0.059	0.080	0.048	0.048	0.047	0.046	0.047	0.038

Note that *B. floridae* has fewer 1-1 phase internal exons than *B. belcheri*, which is probably caused by incomplete prediction and excess gene fragments.

*** For 1-1 phase exons, there are extremely significance (p<1e-16, chi-square test) difference between lancelets and other species.

Supplementary Figure 42. CNE Sequence identity distribution (identify versus counts).

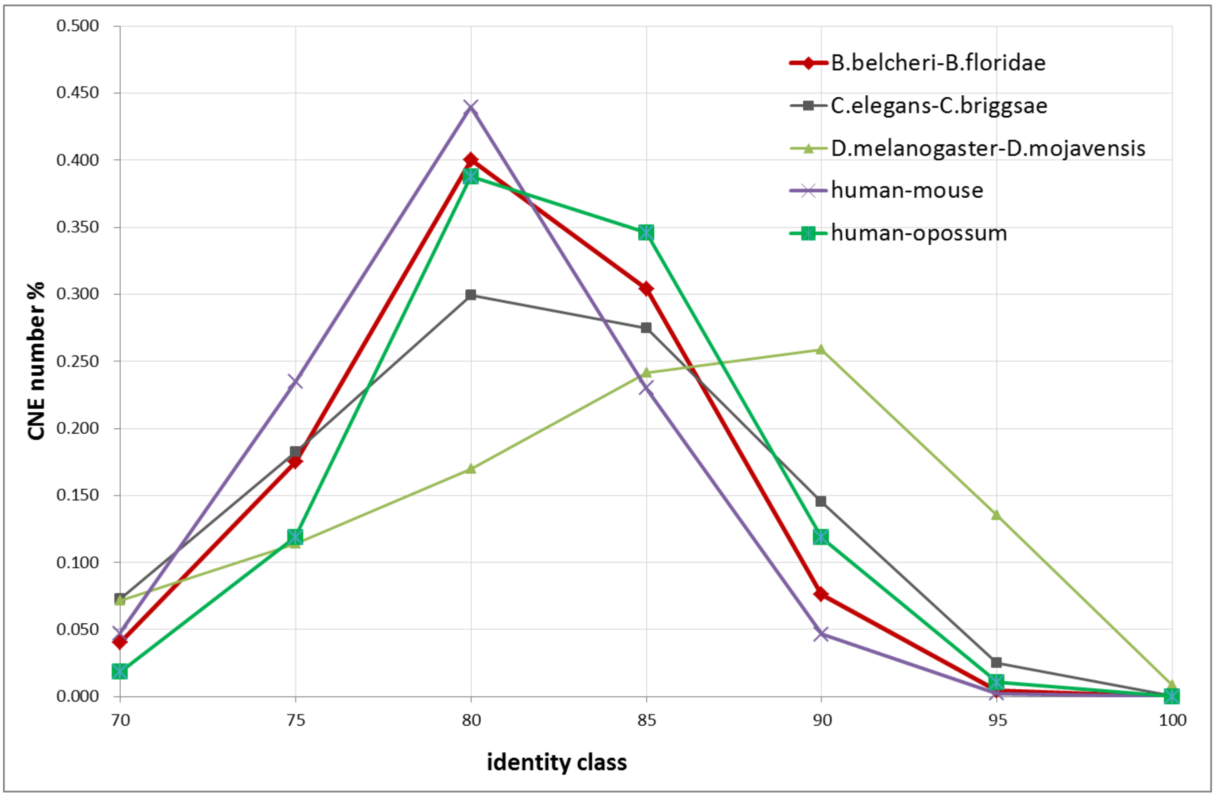

Identity class (%)	B.belcheri -B.floridae	C.elegans -C.briggsae	D.melanogaster -D.mojavensis	Human -mouse	Human -opossum
70	5420	711	1806	17376	2288
75	23615	1783	2868	86611	14759
80	54025	2922	4287	162182	48161
85	41041	2683	6088	84898	42942
90	10298	1417	6531	17151	14701
95	635	244	3415	859	1341
100	12	3	215	2	3

Supplementary Figure 43. CNE Sequence identity distribution (identify versus total length).

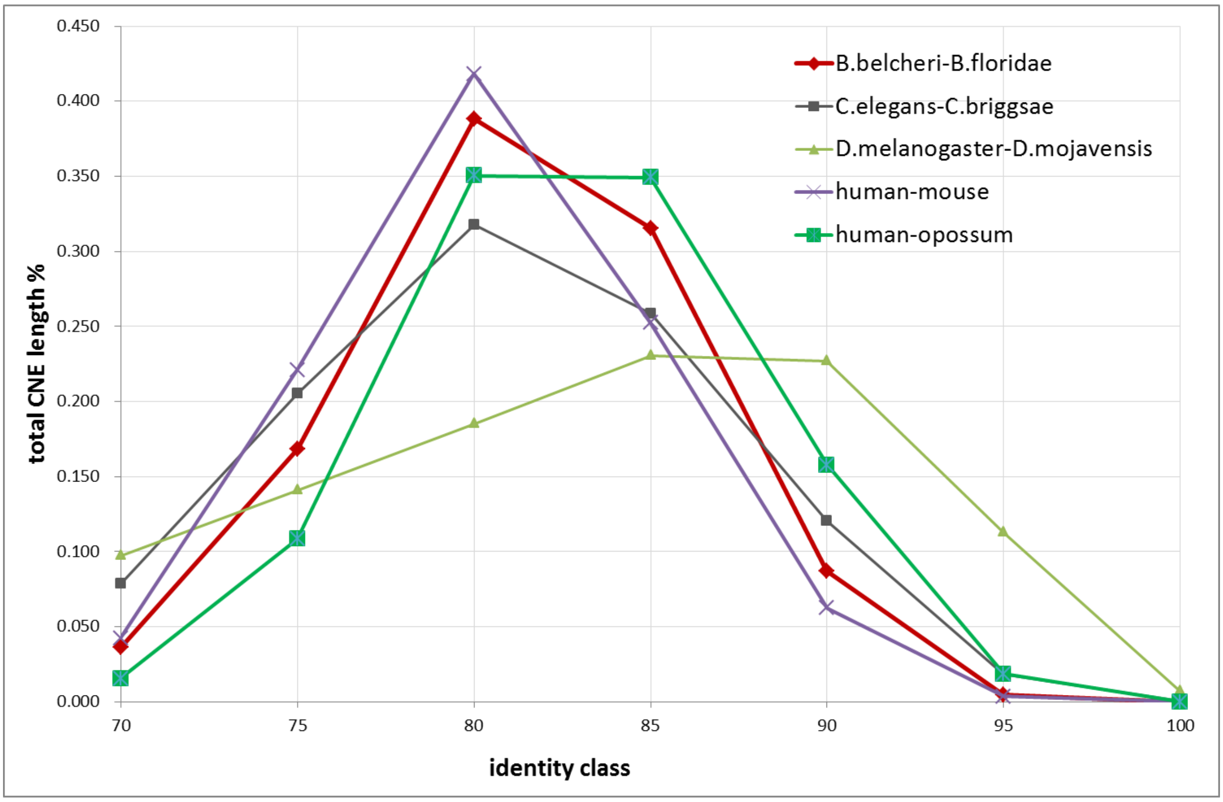

Identity class (%)	B.belcheri -B.floridae	C.elegans -C.briggsae	D.melanogaster -D.mojavensis	Human -mouse	Human -opossum
70	1090142	106578	275812	3616851	429661
75	5042571	277700	400410	18855774	2971733
80	11657115	430429	525341	35684199	9612725
85	9467371	350402	654164	21526789	9582519
90	2604578	163438	643573	5337109	4330405
95	139937	25030	320962	298348	509261
100	2008	266	19387	157	280

Supplementary tables

Supplementary Table 1. Read data sets

A. Genome sequencing on the 454 GS FLX Titanium platform.

insert size	*total length (bp)*	*average length (bp)*	*Total reads count*	*paired-ends count*	*Paired-end proportion*	*Non-duplicated paired-ends count (proportion)* [1]
shotgun	6169149020	364.5	16922833	-	-	
2kb	1765680684	362.3	4873062	3363123	0.69	1175558 (0.35)
3kb	4549373614	332.4	13683939	8754982	0.63	2368918 (0.27)
8kb	1528669219	377.4	4050340	2881810	0.71	905700 (0.31)
20kb	1589992079	376.1	4226653	2895924	0.68	344436 (0.12)

[1]Only paired-end reads with at least 64bp for each ends were counted and used to scaffold the assemblies.

B. Genome sequencing on the Illumina GAIIx platform.

insert size	*total length (bp)*	*average length (bp)*	*Total reads count*	*paired-ends count*	*Paired-end proportion*
340bp	13914864530	2×115	120998822	60499411	-
500bp	9526675210	2×115	82840654	41420327	-
450bp	6214939432	2×115	54999464	27499732	-
600bp	3452743928	2×115	30555256	15277628	-

C. Transcriptome sequencing.

Platform	*Sequencing type*	*Source*	*Raw runs*	*Usable reads or read pairs for mapping*
454 GS FLX Titanium	Shotgun	Adults; mixed embryos	1 run ×3	2,918,945 [1]
Illumina GAIIx	Insert size 300bp (2×115bp)	Different stages of development	1 lane ×8	262,992,523 [2]
		Immune challenged adults	0.5 lane ×3	28,224,038 [2]

[1]Raw 454 reads were quality filtered using sfftools v2.0, and only reads >150bp were retained.

[2]Illumina reads were quality-filtered.

Supplementary Table 2. Genome assembly statistics

	Diploid assembly v7	Haploid assembly v7	Diploid assembly v15	Haploid assembly v15	Diploid assembly v18	Haploid assembly v18 reference	Haploid alt assembly v18 alternative
Scaffold total span bp)	708,200,864	416,219,956	702,393,524	450,740,473	707,122,162	426,108,443	417,037,894
Contig total size (bp)	700,864,312	411,790,424	686,377,301	438,563,617	697,399,180	420,577,928	394,079,242
N-gap total size (bp)	7,336,552	4,429,532	16,016,223	12,176,856	10,508,301	5,530,515	22,958,652
Scaffold number	15,914	5,679	20,509	3,298	10,354	2,307	2,307
Scaffold N50 number	655	125	800	78	751	52	52
Scaffold N25 length (bp)	616,086	1,713,389	554,848	3,253,513	478,784	4,148,982	4,126,612
Scaffold N50 length (bp)	232,747	833,924	150,163	1,497,235	264,466	2,325,619	2,394,960
Scaffold N75 length (bp)	57,767	179,543	27,963	579,503	128,963	1,020,581	1,006,023
Contig number	26,573	12,010	79,255	36,511	49,397	21,504	20,017
Contig N50 number	2,433	1,035	12,205	4,816	6,478	2,569	2,387
Contig N25 length (bp)	152,147	208,749	28,499	46,596	56,197	84,124	84,108
Contig N50 length (bp)	72,664	104,160	16,053	25,074	30,004	45,631	46,422
Contig N75 length (bp)	31,305	46,613	8,578	12,445	14,704	22,302	22437
Depth on all contigs	12.1	-	12.0	-	29.9	-	-
N-gap number	10,659	6,359	58,746	33,213	40,478	19,197	17,710
N-gap average size (bp)	688.3	696.6	272.6	366.6	259.6	288.1	1,296.4

Supplementary Table 3. Estimation of potential misjoins in different haploid assembly versions

		assembly V15 versus V7		assembly V18 versus V7		assembly V18 versus V15
Potential misjoin of scale >100kb :						
	undetermined	177	undetermined	142	undetermined	227
	misjoin on V15	77	misjoin on V18	16	misjoin on V18	27
	misjoin on V7	66	misjoin on V7	77	misjoin on V15	130
Potential misjoin of scale >50kb :						
	undetermined	313	undetermined	243	undetermined	358
	misjoin on V15	104	misjoin on V18	26	misjoin on V18	42
	misjoin on V7	106	misjoin on V7	104	misjoin on V15	162

1. Potential misjoins were first identified by comparing two versions of *B. belcheri* assembly sequences. Then the *B. floridae* draft genome was used as referee to determine on which assembly version a misjoin likely occurred.
2. Comparison was based on three-way all-against-all whole genome alignments, for example, BbelcheriV18-BbelcheriV15-Bfloridae.
3. The scale of a potential misjoin is determined by the flanking alignment length between two *B. belcheri* assemblies, for example, if a misjoin is flanked by 100Kb alignments on both direction, then its scale is >100Kb.
4. To determine the occurring place for a potential misjoin, we required at least 20Kb flanking alignments between the *B. floridae* and the *B. belcheri* genomes. Those misjoins could not met this criteria were classified as “undetermined”.

Supplementary Table 4. Inferred substitution rates and divergence times of selected lineages

A. Estimates of amino acid substitution rates and divergence times of several pairs of closely related species

Species 1	Species 2	Time to most recent common ancestor (Mya) (based on fossils and geological evidence)	Time to most recent common ancestor (Mya) (based on multiple protein sequences, from previous studies)	Time to most recent common ancestor (Mya) (based on multiple protein sequences, from Supplementary Figure 3E)	Substitution per site between the pair based on Supplementary Figure 3C
B. belcheri	*B. floridae*	100-130 [a]	112 [a]	120 (111-129)	0.0586
C. elegans	*C. briggsae*	--	80-100 [b]	82 (80-84)	0.0583
C. intestinalis	*C. savignyi*	--	184 (169-199) [b]	182 (181-182)	0.1017
D. melanogaster	*D. mojavensis*	20-50 [b]	40-100 [b]	89 (74-104)	0.0590
G. aculeatus	*T. nigroviridis*	98-151 [b]	--	147 (134-160)	0.1018
H. sapiens	*G.gallus*	312-331 [b]	--	318 (316-322)	0.0989

[a] 100-130 is based on the geological separation of the Atlantic and Pacific oceans; 112 is based on the analysis of the mitochondrial genome sequences.

[b] Estimated divergence times are taken from the literature .

B. Estimates of amino acid substitution rates and divergence times of several important branches (based on Supplementary Figure 3C, S3E and Supplementary Table 4A)

	Substitution per site	Divergence time (Myr)	1000 * substitution per site / Divergence time
between two lancelets	0.0586	120*2	0.024
the lancelet ancestor	0.1270	502	0.025
between human and chicken	0.0989	319*2	0.016
between tetraodon and stickleback	0.1018	147*2	0.035
the chicken lineage since the split of tetrapods and ray finned fishes	0.0798	420	0.019
the tetraodon lineage since the split of tetrapods and ray finned fishes	0.0954	420	0.023
after the split of vertebrates and lancelets and before the split of jawed and jawless vertebrates	0.0868	144	0.060
after the split of lancelets and vertebrates and before the split of tetrapods and ray finned fishes	0.1170	203	0.058

Supplementary Table 5. The orthologous protein identity and dN/dS ratios in different GO terms (human versus mouse, and Chinese lancelet versus Florida lancelet).

	Human vs mouse		two lancelets				
go_terms	gene number	avg. identity	gene number	avg. identity	avg. dN/dS	avg. dN	avg. dS
**Biology process**							
cell killing	72	74.2	13	86.8	0.0762	0.0066	0.0809
rhythmic process	192	87.5	133	86.5	0.0405	0.0038	0.0760
metabolic process	9050	85.7	5260	85.2	0.0732	0.0055	0.0772
cellular component organization or biogenesis	4397	86.7	2395	85.1	0.0554	0.0046	0.0756
multi-organism process	1208	85.3	627	84.6	0.0738	0.0054	0.0757
locomotion	1261	87.5	667	84.6	0.0703	0.0053	0.0711
positive regulation of biological process	3587	86.7	1690	84.5	0.0685	0.0052	0.0727
cellular process	12452	85.3	6456	84.4	0.0696	0.0053	0.0761
developmental process	4596	86.1	2587	84.2	0.0645	0.0048	0.0710
multicellular organismal process	5734	85.8	3033	84.0	0.0678	0.0051	0.0721
single-organism process	10608	85.2	5368	84.0	0.0691	0.0051	0.0760
negative regulation of biological process	3206	86.5	1569	83.9	0.0666	0.0052	0.0726
regulation of biological process	7906	85.6	3675	83.9	0.0690	0.0050	0.0729
biological regulation	8436	85.7	4002	83.8	0.0684	0.0050	0.0729
localization	4335	86.6	2584	83.6	0.0669	0.0052	0.0753
immune system process	1844	83.7	712	83.6	0.0805	0.0059	0.0688
establishment of localization	3527	86.9	2167	83.5	0.0649	0.0050	0.0751
response to stimulus	6534	85.7	3290	83.4	0.0768	0.0054	0.0737
signaling	4701	86.7	2196	83.4	0.0665	0.0049	0.0703
reproduction	1086	82.7	688	83.1	0.0665	0.0055	0.0749
reproductive process	974	82.9	614	83.0	0.0682	0.0057	0.0742
growth	784	87.1	520	82.7	0.0651	0.0057	0.0699
biological adhesion	945	84.6	420	78.8	0.0838	0.0065	0.0703

	Human vs mouse		two lancelets				
go_terms	gene number	avg. identity	gene number	avg. identity	avg. dN/dS	avg. dN	avg. dS
**Cellular location**							
nucleoid	41	85.4	31	89.1	0.0612	0.0031	0.0789
macromolecular complex	3926	87.1	2210	87.1	0.0547	0.0038	0.0766
membrane-enclosed lumen	2611	86.0	1302	86.9	0.0609	0.0051	0.0811
organelle part	6176	85.9	3376	86.1	0.0647	0.0048	0.0782
virion	8	77.2	11	86.0	0.0262	0.0022	0.0432
virion part	8	77.2	11	86.0	0.0262	0.0022	0.0432
synapse	507	91.1	409	85.6	0.0614	0.0048	0.0676
organelle	10130	85.4	5188	85.5	0.0673	0.0051	0.0768
synapse part	374	91.0	320	85.4	0.0605	0.0049	0.0687
cell	13670	85.1	6620	84.7	0.0688	0.0052	0.0766
cell part	13667	85.1	6619	84.7	0.0688	0.0052	0.0766
cellular component	16198	84.4	7813	84.1	0.0718	0.0055	0.0774
cell junction	783	87.7	450	83.7	0.0563	0.0047	0.0726
membrane	7332	85.0	3763	82.6	0.0800	0.0058	0.0751
membrane part	5733	84.5	2878	81.9	0.0793	0.0060	0.0747
extracellular matrix part	187	83.2	104	78.4	0.0984	0.0084	0.0686
extracellular region	1894	80.2	766	78.2	0.1066	0.0082	0.0728
extracellular region part	1084	81.2	499	77.9	0.1032	0.0078	0.0713
extracellular matrix	418	83.3	232	77.2	0.0926	0.0073	0.0676

go_terms	Human vs mouse gene number	Human vs mouse avg. identity	two lancelets gene number	two lancelets avg. identity	two lancelets avg. dN/dS	two lancelets avg. dN	two lancelets avg. dS
**Molecular function**							
chemoattractant activity	15	88.0	2	92.5	0.0772	0.0087	0.0564
metallochaperone activity	4	85.8	4	92.3	0.0276	0.0019	0.0214
guanyl-nucleotide exchange factor activity	186	86.9	64	87.8	0.0291	0.0021	0.0821
translation regulator activity	23	92.5	7	87.3	0.0327	0.0038	0.0722
nucleic acid binding transcription factor activity	949	86.8	238	87.2	0.0513	0.0032	0.0574
enzyme regulator activity	778	85.9	308	85.5	0.0561	0.0049	0.0819
protein binding transcription factor activity	512	88.0	175	85.2	0.0499	0.0046	0.0783
catalytic activity	5242	86.2	3971	84.9	0.0810	0.0062	0.0794
receptor regulator activity	38	88.4	29	84.4	0.1696	0.0121	0.0777
structural molecule activity	569	85.9	236	84.1	0.0655	0.0044	0.0712
binding	11346	85.3	5685	84.1	0.0717	0.0054	0.0757
molecular function	15353	84.6	7869	83.9	0.0728	0.0056	0.0775
antioxidant activity	66	81.8	36	83.3	0.0649	0.0046	0.0763
channel regulator activity	82	89.0	52	83.0	0.0711	0.0047	0.0732
electron carrier activity	80	86.8	66	81.9	0.0910	0.0078	0.0869
molecular transducer activity	1503	84.5	555	81.1	0.0777	0.0056	0.0684
transporter activity	1133	87.1	837	81.0	0.0705	0.0054	0.0743
receptor activity	1421	83.4	576	79.1	0.0914	0.0068	0.0717
chemorepellent activity	7	96.8	2	69.3	0.0214	0.0027	0.0642

Supplementary Table 6. The numbers of total and TE-containing large polymorphic indels.

	Total length	Total count	TE-containg 1e-5;cov50	TE-containg 1e-10;cov50	TE-containg 1e-5;cov35	TE-containg 1e-20;cov35
150-5000bp	35568501	56605	34899	34430	40902	38031
150-10000bp	42269944	57602	35034	34565	41221	38349
150-20000bp	46412420	57903	35036	34567	41234	38362
200-5000bp	33967149	47358	31101	30842	36441	34784
200-10000bp	40668592	48355	31236	30977	36760	35102
200-20000bp	44811068	48656	31238	30979	36773	35115
300-5000bp	31167554	35862	23701	23604	28162	27461
300-10000bp	37868997	36859	23836	23739	28481	27779
300-20000bp	42011473	37160	23838	23741	28494	27792

TE=transposable elements; Blast Evalue=1e-5, 1e-10 or 1e-20; Blast coverage=50% or 35%.

Supplementary Table 7. The dN/dS ratios for coding sequences in Chinese lancelets.

Blast evalue & coverage against GO proteins	Gene pairs	ng_w	ng_dn	ng_ds	yn_w	yn_dn	yn_ds
Evalue=1e-10;cov=10%	19626	0.1102	0.0075	0.0677	0.1148	0.0075	0.0651
Evalue=1e-10;cov=50%	16108	0.0889	0.0062	0.0696	0.0913	0.0062	0.0675
Evalue=1e-30;cov=50%	12747	0.0822	0.0059	0.0713	0.0842	0.0058	0.0692
Evalue=1e-30;cov=70%	10171	0.0699	0.0052	0.0743	0.0711	0.0051	0.0724
Evalue=1e-50;cov=70%	8613	0.0667	0.0050	0.0749	0.0678	0.0050	0.0730

ng: Nei & Gojobori (1986) method; yn: Yang and Nielsen (2000) method; w: dN/dS.

Supplementary Table 8. Whole-genome re-sequencing and bisulfite sequencing data set

Animal No.	*platform*	*Insert size*	*Clean reads*	*Clean bases*
Bbe01	Hiseq2000; 2x101bp	355bp	210,710,342	21,281,744,542
Bbe03	Hiseq2000; 2x101bp	416bp	219,989,476	22,218,937,076
Bbe06	Hiseq2000; 2x101bp	400bp	234,033,972	23,637,431,172
Bbe23A	Hiseq2500; 2x150bp	420bp	268,232,290	40,234,843,500
Bbe23F	Hiseq2500; 2x150bp	431bp	275,393,528	41,309,029,200
Bbe23A (bisulfite)	Hiseq2000; 2x100bp	384bp	209,761,726	20,976,172,600
Bbe23F (bisulfite)	Hiseq2000; 2x100bp	395bp	233,798,050	23,379,805,000

Supplementary Table 9. Pairwise p-distance between different Chinese lancelet individuals

(A) All gap-free and N-free 6-way alignments were used (50031253bp)

	Xiamen			*Zhanjiang*		
	bbv18ref	bbe23a	bbe23f	bbe01	bbe03	bbe06
bbv18ref		0.0474	0.0476	0.0482	0.0481	0.0483
bbe23a	0.0474		0.0486	0.0488	0.0487	0.0489
bbe23f	0.0476	0.0486		0.0489	0.0490	0.0490
bbe01	0.0482	0.0488	0.0489		0.0482	0.0494
bbe03	0.0481	0.0487	0.0490	0.0482		0.0492
bbe06	0.0483	0.0489	0.0490	0.0494	0.0492	

(B) Gap-free and N-free 6-way alignments in coding regions were used (3230937bp)

	Xiamen			*Zhanjiang*		
	bbv18ref	bbe23a	bbe23f	bbe01	bbe03	bbe06
bbv18ref		0.0313	0.0315	0.0314	0.0315	0.0316
bbe23a	0.0313		0.0320	0.0321	0.0322	0.0323
bbe23f	0.0315	0.0320		0.0320	0.0322	0.0321
bbe01	0.0314	0.0321	0.0320		0.0320	0.0323
bbe03	0.0315	0.0322	0.0322	0.0320		0.0325
bbe06	0.0316	0.0323	0.0321	0.0323	0.0325	

Supplementary Table 10. The composition of repetitive sequences in two lancelet species

Class of TEs	% of *B. floridae* genome (bfv2)[1]	% of *B. belcheri* genome (v18)[1]	No. of non-redundant transcripts in *B.belcheri*[2]	highest expression (FPKM) in *B.belcheri*[3]
**Total DNA Transposons**	**12.64**	**12.74**		
**"Cut and Paste"**	**5.65**	**7.16**		
TcMar/pogo	0.37	0.85	69	23.24
hAT	1.32	2.34	17	1.24
EnSpm	0.49	1.03	16	1.24
PIF/Harbinger	1.82	0.73	6	0.26
PiggyBac	0.68	0.1		
Merlin	<0.01	0.11	3	0.33
Mule/MuDR	0.31	0.21	6	0.97
Kolobok	0.04	0.28	7	0.78
P	0.07	0.18	7	0.78
Sola1/2/3	0.2	0.7	6	7.37
Chapaev	0.03	0.19	6	1.12
Ginger	0.02	0.03		
Academ	0.07	0.1		
Zator	0.03	0.15	15	0.53
Novosib	0.13	0.12		
ISL2eu	0.02	0.01		
IS4eu	0.04	0.02		
ProtoRag	0.01	0.01	2	0.32
**"Rolling circle" Helitrons**	**1.03**	**0.63**	**8**	**6.70**
**"Self-synthesizing" Polinton**	**0.17**	**1.13**	**28**	**1.25**
**MITE**	**0.12**	**1.09**		
**Other**	**5.67**	**2.73**		
**Total retrotransposons**	**9.58**	**10.33**		
**LTR retrotransposons**	**0.72**	**1.07**		
Gypsy	0.28	0.78	63	6.43
BEL/Pao	0.01	0.01	5	1.17
ERV	0.04	0.03		
Copia	0.01	0.01		

Class of TEs	% of *B. floridae* genome (bfv2)[1]	% of *B. belcheri* genome (v18)[1]	No. of non-redundant transcripts in *B.belcheri*[2]	highest expression (FPKM) in *B.belcheri*[3]
Other	0.38	0.24		
**LINEs**	**6.26**	**7.11**		
L1/Tx1	0.24	0.15	38	2.95
L2/Crack	0.44	0.32	15	8.74
L3/CR1	1.52	2.70	197	110.52
RTE/RTEX	1.56	1.84	50	1.51
Jockey	0.23	0.42	34	1.77
REX1	2.16	1.55		
I/LOA	0.01	0.01	12	5.44
Proto2	0.02	0.03		
Daphne	<0.01	<0.01		
R2	0.02	0.02		
Hero/NeSL	0.04	0.04		
Ingi/Vingi	0.01	0.02		
**DIRS**	**0.05**	**0.06**	**12**	**2.58**
**Penelope**	**1.09**	**0.66**	**135**	**20.09**
**SINEs**	**1.46**	**1.43**		
**Other weakly supported TE[4]**				
Ambal, CRE, RandI, Proto1				
Kiri, R4, Tad1				
**Unknown**	**4.41**	**3.92**		
**Total TE**	**26.63**	**26.99**		

[1]The searched conducted using RepeatMasker and a curated TE library (including *de novo* identified *B. belcheri* TE, known deuterstome TE and known *B. floridae* TE).

[2]Transcripts for TE protein components reconstructed by Cufflinks with ~300 million EST reads or read pairs.

[3]The expression level for the highest expressed transcript in the mixed transcriptome (libraries pooled together).

[4]These TE elements have few detected copies (1-60) in genomes and have no evidence of ORF sequences.

Supplementary Table 11. DCJ distances for eight species pairs

Species 1	Species 2	Number of gene pairs used	Number of chromo-somes or scaffolds used for species 1	Number of chromo-somes or scaffolds used for species 2	Number of DCJ rearrangement	Relative DCJ distance	Protein divergence	Divergence time ** (Mya)
C. intestinalis	*C. savignyi*	3619	34	64	1457	0.402	0.0961	180
B.belcheri	*B. floridae*	8806	186*	195*	2000	0.227	0.0554	101(100-130)
C. elegans	*C. briggsae*	7677	6	7	1643	0.214	0.0553	100
D. mojavensis	*D.melanogaster*	6370	27*	6	1424	0.224	0.0559	47-100
G. aculeatus	*T. nigroviridis*	9384	21	21	831	0.088	0.0961	97-150
H. sapiens	*G. gallus*	8486	23	27	1200	0.141	0.0932	300
M. musculus	*H. sapiens*	14058	20	23	759	0.054	-	62-101

* For these species, scaffolds containing more than 30 genes were used in the analysis of genome rearrengements.

** Divergence time for two lancelets is estimated by this study and taken from literatures. Divergence times for other species pairs are taken from literature.

Supplementary Table 12. Orthologous gene families between several species pairs

Species 1	Species 2	50% Identity and 50% coverage			20% Identity and 20% coverage		
		Gene family number	Number of genes involved in species 1	Number of genes involved in species 2	Gene family number	Number of genes involved in species 1	Number of genes involved in species 2
C. intestinalis	*C. savignyi*	5720	6322	6102	7094	8054	7721
B.belcheri	*B. floridae*	12664	15026	15166	14843	18167	17735
C. elegans	*C. briggsae*	11528	12747	12170	13231	15335	14474
D. mojavensis	*D.melanogaster*	9710	10074	10077	11189	11716	11768
G. aculeatus	*T. nigroviridis*	13669	15141	14667	14394	15703	15231
H. sapiens	*G. gallus*	10660	11078	11984	12428	13553	14630
R. macaque	*H. sapiens*	16504	17823	17546	16680	18100	17786

Supplementary Table 13. Gene prediction statistics in two lancelet species

The *B. floridae* gene set is taken from the original paper [1].
The *B. belcheri* gene set contains 30,392 gene models, of which 4,399 models have 7,254 evidence-based alternative splicing isoforms of transcripts.

	B.floridae haploid assembly (% of genome)	*B. belcheri haploid assembly* reference v18 (% of genome)
Genome Size	521,895,125	426,108,443
GeneModels Size	269,183,467(51.5%)	270,660,323(63.5%)
GeneModels Num	28,666	30,392
SingleExon Genes	4,313	3,526
Intron Size	225,021,120(43.11%)	215,348,196(50.5%)
Intron Num	172,731	231,571
Transcripts Num	28,666	37,646
CDS Size	39,885,842(7.6%)	47,983,502(11.3%)
CDS Num	201,398	268,248
Mean CDS per gene	7	8.6
Mean Length of CDS	196	180
Mean Length of Intron	1308	929

Supplementary Table 14. Bulk methylation statistics

	bbe23a-nuclear		bbe23a-mitochondrial		bbe23f-nuclear		bbe23f-mitochondrial	
assembly size	623Mb		15kb		620Mb		15kb	
BS-seq coverage	16X		>1000X		17X		>1000X	
	**total C***	**methylation level**	**total C**	**methylation level**	**total C**	**methylation level**	**total C**	**methylation level**
CG	31903485	21.14%	632	0.314%	31789180	20.76%	621	0.294%
CHG	48179425	0.361%	792	0.332%	47918003	0.327%	770	0.320%
CHH	153836636	0.369%	3993	0.253%	152919067	0.334%	3952	0.270%

*Total callable cytosines with sequence coverage (statistic data from Bis-SNP).

Supplementary Table 15. Total coding DNA sequence (CDS) length for several species

Species	Gene number	CDS length	Species	Gene number	CDS length
N. vectensis	27273	27054876	*C. gigas*	28027	36573873
C. elegans	20389	25398398	*C. briggsae*	21986	26220803
D. mojavensis	14596	21620477	*D.melanogaster*	13768	22640202
C. intestinalis	14180	16742259	*C. savignyi*	11604	13853860
B. floridae	28667	roughly estimated as 39885569~40500060*			
S. purpuratus	n/a**	roughly estimated as 31987518**			
B.belcheri	30392	47983502	*X. tropicalis*	18429	30021039
G. aculeatus	20787	32651055	*T. nigroviridis*	19602	30047244
H. sapiens	21553	35672852	*D. rerio*	26095	41453274
R. macaque	21403	32121086	*G. gallus*	16736	24666339
R. norvegicus	22938	33809809	*M. musculus*	23081	36271978

*The first CDS length for *B. floridae* is directly calculated based on the gene set for the reference haploid genome. Its total length is still large than other species except zebrafish *D. rerio*. However, we believed that this value is highly underestimated because of under-prediction, assembly errors and the less completeness of the reference haploid genome sequence, so we estimed a second CDS length for *B. floridae* haploid genome as followed. Total CDS length for the *B. floridae* diploid genome is 68850102bp and the completeness of two haploid sequences is ~85% (Putnam et al. 2008), therefore we estimated that 68850102/(0.85x2)= 40500060bp. Moreover, an addition of ~15Mb coding sequence fragments were identified from introns and intergenic regions (Supplementary Note 11).

**The purple sea urchin genome assembly is presented as diploid assembly and no haploid assembly is available. The gene number and total CDS length for the diploid assembly are 42420 and 54378780bp [2]. We assumed that the compeleness for the sea urchin haploid assembly is also ~85%, so we estimated the CDS length for haploid genome is 54378780/(0.85x2)=31987518bp.

Supplementary Table 16. Total protein domain length for several species

Species	E-value <1				E-value <1e-5			
	domain type number*	domain type number >2	domain number	Total domain Length	domain type number*	domain type number >2	domain number	Total domain Length
C. intestinalis	3311	1801	18084	2146237	3238	1740	15974	2064721
C. savignyi	3232	1531	15652	1855276	3166	1480	13852	1789158
Cnidaria	4180	2380	31643	3614726	4109	2315	28011	3482092
B.belcheri	**4383**	**2417**	**48700**	**5381314**	**4299**	**2340**	**43827**	**5155557**
B. floridae ******	**4254**	**3707**	**93983**	**8729302**	**4173**	**3639**	**81180**	**8315457**
B.belcheri+B. floridae	**4471**	**-**	**-**	**-**	**4380**	**-**	**-**	**-**
C. elegans	3475	1718	22178	3226314	3408	1664	20404	3149641
Mosquito	3663	1896	21945	2800274	3606	1849	19363	2711559
D.melanogaster	3775	1872	19451	2546665	3719	1809	17223	2470401
C. gigas	4114	2279	33334	3756377	4024	2197	28904	3583077
S. purpuratus ***	3873	3780	68364	7259892	3805	3715	60915	6983901
T. nigroviridis	4258(4051)****	2694	37814	4409233	4212(4002)	2647	34065	4275951
G. gallus	4227(3944)	2428	28582	3335479	4176(3887)	2379	25650	3229864
H. sapiens	4729(4312)	2860	44881	4710863	4664(4245)	2800	39899	4546872
X. xenopus	4354(4103)	2610	37852	4374550	4292(4041)	2559	33687	4232316
M. musculus	4686(4286)	2869	44453	4907806	4637(4228)	2824	39897	4756285
D. rerio	4493(4242)	2872	57550	5853784	4439(4188)	2811	50560	5630372
**All six vertebrates**	**4869(4409)**	**-**	**-**	**-**	**4793(4339)**	**-**	**-**	**-**

* All possible protein isoforms of a species were used to calculate the domain type number.

** The diploid draft genome of *B. floridae* is used here because the reference haploid genome is not complete. The total domain length for haploid genome can be roughly estimated as 8729302/(0.85x2)=5134884bp for E-value<1 and 8315457/(0.85x2)=4891445bp for E-value<1e-5).

*** The diploid draft genome is used for the sea urchin *S. purpuratus* because haploid genome is not available. The total domain length for haploid genome can be roughly estimated as 7259892/(0.85x2)=4270525bp for E-value<1 and 6983901/(0.85x2)=4108177bp for E-value<1e-5).

****() The number inside the parenthesis refers to the domain number excluding vertebrate-specific domain types.

Supplementary Table 17. Total protein domain (plus PfamB) length for several species

Species	E-value <1				E-value <1e-5			
	domain type number*	domain type number >2	domain number	Total domain Length	domain type number*	domain type number >2	domain number	Total domain Length
C. intestinalis	5434	2713	23146	2940059	4842	2375	19199	2684765
C. savignyi	5244	2229	19654	2497521	4712	1943	16504	2307436
Cnidaria	6950	3819	43741	5370605	6257	3355	35585	4792206
B.belcheri	**7648**	**4146**	**68999**	**8241777**	**6912**	**3569**	**57174**	**7430215**
B. floridae* *	**7473**	**6214**	**122447**	**13357142**	**6681**	**5632**	**99625**	**11958001**
B.belcheri+B. floridae	**8142**	**-**	**-**	**-**	**7214**	**-**	**-**	**-**
C. elegans	5426	2525	28561	4297836	4818	2221	24493	3995445
Mosquito	6336	3292	32025	4427900	5811	2903	25313	3954658
D.melanogaster	6702	3272	29498	4379747	6187	2838	23129	3904352
S. purpuratus ***	6492	6299	86034	10059936	5850	5694	71831	9092875
T. nigroviridis	7150	4380	48797	6424637	6670	4029	41970	6006632
G. gallus	7229	4063	37638	5136643	6695	3689	32010	4766113
H. sapiens	8321	4873	57748	7265838	7599	4452	49001	6749292
X. xenopus	7415	4322	48490	6399611	6865	3949	41068	5948186
M. musculus	8200	4875	57434	7359739	7541	4434	48724	6827291
zebrafish	7904	4981	73593	8745098	7267	4498	61442	8013236

* All possible protein isoforms of a species were used to calculate the domain type number.

** The diploid draft genome of *B. floridae* is used here because the reference haploid genome is not complete. The total domain length for haploid genome can be roughly estimated as 13357142/(0.85x2)=7857142bp for E-value<1 and 11958001/(0.85x2)=7034118bp for E-value<1e-5).

*** The diploid draft genome is used for the sea urchin *S. purpuratus* because haploid genome is not available. The total domain length for haploid genome can be roughly estimated as 10059936/(0.85x2)=5917609bp for E-value<1 and 9092875/(0.85x2)=5348750bp for E-value<1e-5).

Supplementary Table 18. Ancient protein domain preservation

Species	Domain type number (E-value<1)	Domain type number (E-value<1e-5)
zebrafish	4178	4123
T. nigroviridis	3993	3943
X. xenopus	4044	3978
G. gallus	3885	3825
M. musculus	4214	4156
H. sapiens	4237	4171
**All six vertebrates**	**4328**	**4260**
B.belcheri	4273	4203
B. floridae (diploid)	4157	4081
**Two lancelets**	**4329**	**4257**

* Total number of ancient domain types is 5117 for E-value <1 and 4994 for E-value <1e-5.

** All possible protein isoforms of a species were used to calculate the domain type number.

Supplementary Table 19. Ancient domain types preserved in amphioxus but lost in vertebrates

There are a total of 144 ancient protein domain types that are preserved in at least one lancelet species but not found in any of six examined vertebrates (tetraodon, zebrafish, xenopus, chicken, mouse and human).
* All possible protein isoforms of a species were used to calculate the domain type number.
** No E-value cutoff is applied here.

pfamID	pfamName	pfamDesc	clanID
PF00722	Glyco_hydro_16	Glycosyl hydrolases family 16	CL0004
PF03825	Nuc_H_symport	Nucleoside H+ symporter	CL0015
PF02958	EcKinase	Ecdysteroid kinase	CL0016
PF03377	Avirulence	Xanthomonas avirulence protein, Avr/PthA	CL0020
PF08713	DNA_alkylation	DNA alkylation repair enzyme	CL0020
PF01637	Arch_ATPase	Archaeal ATPase	CL0023
PF02562	PhoH	PhoH-like protein	CL0023
PF03193	DUF258	Protein of unknown function, DUF258	CL0023
PF13521	AAA_28	AAA domain	CL0023
PF10142	PhoPQ_related	PhoPQ-activated pathogenicity-related protein	CL0028
PF12740	Chlorophyllase2	Chlorophyllase enzyme	CL0028
PF00908	dTDP_sugar_isom	dTDP-4-dehydrorhamnose 3,5-epimerase	CL0029
PF06172	Cupin_5	Cupin superfamily (DUF985)	CL0029
PF13350	Y_phosphatase3	Tyrosine phosphatase family	CL0031
PF04261	Dyp_perox	Dyp-type peroxidase family	CL0032
PF00449	Urease_alpha	Urease alpha-subunit, N-terminal domain	CL0034
PF01645	Glu_synthase	Conserved region in glutamate synthase	CL0036
PF04898	Glu_syn_central	Glutamate synthase central domain	CL0036
PF06415	iPGM_N	BPG-independent PGAM N-terminus (iPGM_N)	CL0036
PF03358	FMN_red	NADPH-dependent FMN reductase	CL0042
PF12902	Ferritin-like	Ferritin-like	CL0044
PF01643	Acyl-ACP_TE	Acyl-ACP thioesterase	CL0050
PF12680	SnoaL_2	SnoaL-like domain	CL0051
PF03417	AAT	Acyl-coenzyme A:6-aminopenicillanic acid acyl-transferase	CL0052
PF01097	Defensin_2	Arthropod defensin	CL0054
PF00150	Cellulase	Cellulase (glycosyl hydrolase family 5)	CL0058
PF00331	Glyco_hydro_10	Glycosyl hydrolase family 10	CL0058
PF00933	Glyco_hydro_3	Glycosyl hydrolase family 3 N terminal domain	CL0058
PF02638	DUF187	Uncharacterised BCR, COG1649	CL0058
PF00759	Glyco_hydro_9	Glycosyl hydrolase family 9	CL0059
PF03663	Glyco_hydro_76	Glycosyl hydrolase family 76	CL0059
PF01041	DegT_DnrJ_EryC1	DegT/DnrJ/EryC1/StrS aminotransferase family	CL0061
PF02353	CMAS	Mycolic acid cyclopropane synthetase	CL0063
PF03492	Methyltransf_7	SAM dependent carboxyl methyltransferase	CL0063
PF05050	Methyltransf_21	Methyltransferase FkbM domain	CL0063
PF01353	GFP	Green fluorescent protein	CL0069
PF01676	Metalloenzyme	Metalloenzyme superfamily	CL0088
PF02995	DUF229	Protein of unknown function (DUF229)	CL0088

pfamID	pfamName	pfamDesc	clanID
PF00529	HlyD	HlyD family secretion protein	CL0105
PF13533	Biotin_lipoyl_2	Biotin-lipoyl like	CL0105
PF03702	UPF0075	Uncharacterised protein family (UPF0075)	CL0108
PF03452	Anp1	Anp1	CL0110
PF09837	DUF2064	Uncharacterized protein conserved in bacteria (DUF2064)	CL0110
PF00982	Glyco_transf_20	Glycosyltransferase family 20	CL0113
PF00440	TetR_N	Bacterial regulatory proteins, tetR family	CL0123
PF05585	DUF1758	Putative peptidase (DUF1758)	CL0129
PF00668	Condensation	Condensation domain	CL0149
PF03174	CHB_HEX_C	Chitobiase/beta-hexosaminidase C-terminal domain	CL0159
PF05345	He_PIG	Putative Ig domain	CL0159
PF01717	Meth_synt_2	Cobalamin-independent synthase, Catalytic domain	CL0160
PF02407	Viral_Rep	Putative viral replication protein	CL0169
PF13539	Peptidase_M15_4	D-alanyl-D-alanine carboxypeptidase	CL0170
PF13462	Thioredoxin_4	Thioredoxin	CL0172
PF12974	Phosphonate-bd	ABC transporter, phosphonate, periplasmic substrate-binding protein	CL0177
PF13188	PAS_8	PAS domain	CL0183
PF03022	MRJP	Major royal jelly protein	CL0186
PF06739	SBBP	Beta-propeller repeat	CL0186
PF03175	DNA_pol_B_2	DNA polymerase type B, organellar and viral	CL0194
PF02018	CBM_4_9	Carbohydrate binding domain	CL0202
PF00553	CBM_2	Cellulose binding domain	CL0203
PF00372	Hemocyanin_M	Hemocyanin, copper containing domain	CL0205
PF10604	Polyketide_cyc2	Polyketide cyclase / dehydrase and lipid transport	CL0209
PF09451	ATG27	Autophagy-related protein 27	CL0226
PF03070	TENA_THI-4	TENA/THI-4/PQQC family	CL0230
PF01771	Herpes_alk_exo	Herpesvirus alkaline exonuclease	CL0236
PF08378	NERD	Nuclease-related domain	CL0236
PF09588	YqaJ	YqaJ-like viral recombinase domain	CL0236
PF13420	Acetyltransf_4	Acetyltransferase (GNAT) domain	CL0257
PF03445	DUF294	Putative nucleotidyltransferase DUF294	CL0260
PF02900	LigB	Catalytic LigB subunit of aromatic ring-opening dioxygenase	CL0283
PF00775	Dioxygenase_C	Dioxygenase	CL0287
PF12949	HeH	HeH/LEM domain	CL0306
PF12867	DinB_2	DinB superfamily	CL0310
PF02152	FolB	Dihydroneopterin aldolase	CL0334
PF01613	Flavin_Reduct	Flavin reductase like domain	CL0336
PF00589	Phage_integrase	Phage integrase family	CL0382
PF01872	RibD_C	RibD C-terminal domain	CL0387
PF03564	DUF1759	Protein of unknown function (DUF1759)	CL0523
PF00391	PEP-utilizers	PEP-utilising enzyme, mobile domain	
PF00484	Pro_CA	Carbonic anhydrase	
PF00547	Urease_gamma	Urease, gamma subunit	
PF00699	Urease_beta	Urease beta subunit	

pfamID	pfamName	pfamDesc	clanID
PF01288	HPPK	7,8-dihydro-6-hydroxymethylpterin-pyrophosphokinase (HPPK)	
PF01326	PPDK_N	Pyruvate phosphate dikinase, PEP/pyruvate binding domain	
PF01469	Pentapeptide_2	Pentapeptide repeats (8 copies)	
PF01493	GXGXG	GXGXG motif	
PF01682	DB	DB module	
PF01730	UreF	UreF	
PF01774	UreD	UreD urease accessory protein	
PF01786	AOX	Alternative oxidase	
PF01894	UPF0047	Uncharacterised protein family UPF0047	
PF01915	Glyco_hydro_3_C	Glycosyl hydrolase family 3 C-terminal domain	
PF02363	C_tripleX	Cysteine rich repeat	
PF02698	DUF218	DUF218 domain	
PF03030	H_PPase	Inorganic H+ pyrophosphatase	
PF03067	Chitin_bind_3	Chitin binding domain	
PF03479	DUF296	Domain of unknown function (DUF296)	
PF03639	Glyco_hydro_81	Glycosyl hydrolase family 81	
PF03989	DNA_gyraseA_C	DNA gyrase C-terminal domain, beta-propeller	
PF04143	Sulf_transp	Sulphur transport	
PF04199	Cyclase	Putative cyclase	
PF04457	DUF504	Protein of unknown function (DUF504)	
PF04536	Repair_PSII	Repair protein	
PF04852	DUF640	Protein of unknown function (DUF640)	
PF05183	RdRP	RNA dependent RNA polymerase	
PF05380	Peptidase_A17	Pao retrotransposon peptidase	
PF05444	DUF753	Protein of unknown function (DUF753)	
PF05497	Destabilase	Destabilase	
PF05551	DUF1519	Protein of unknown function (DUF1519)	
PF05681	Fumerase	Fumarate hydratase (Fumerase)	
PF05683	Fumerase_C	Fumarase C-terminus	
PF05701	DUF827	Plant protein of unknown function (DUF827)	
PF05960	DUF885	Bacterial protein of unknown function (DUF885)	
PF06032	DUF917	Protein of unknown function (DUF917)	
PF06101	DUF946	Plant protein of unknown function (DUF946)	
PF06694	Plant_NMP1	Plant nuclear matrix protein 1 (NMP1)	
PF06869	DUF1258	Protein of unknown function (DUF1258)	
PF06918	DUF1280	Protein of unknown function (DUF1280)	
PF07081	DUF1349	Protein of unknown function (DUF1349)	
PF07173	DUF1399	Protein of unknown function (DUF1399)	
PF07271	Cytadhesin_P30	Cytadhesin P30/P32	
PF08317	Spc7	Spc7 kinetochore protein	
PF08376	NIT	Nitrate and nitrite sensing	
PF08438	MMR_HSR1_C	GTPase of unknown function C-terminal	
PF08570	DUF1761	Protein of unknown function (DUF1761)	
PF08719	DUF1768	Domain of unknown function (DUF1768)	
PF09458	H_lectin	H-type lectin domain	

pfamID	pfamName	pfamDesc	clanID
PF09995	DUF2236	Uncharacterized protein conserved in bacteria (DUF2236)	
PF10017	DUF2260	Uncharacterized conserved protein (DUF2260)	
PF10517	DM13	Electron transfer DM13	
PF10998	DUF2838	Protein of unknown function (DUF2838)	
PF11312	DUF3115	Protein of unknown function (DUF3115)	
PF12345	DUF3641	Protein of unknown function (DUF3641)	
PF13020	DUF3883	Domain of unknown function (DUF3883)	
PF13148	DUF3987	Protein of unknown function (DUF3987)	
PF13164	DUF4002	Protein of unknown function (DUF4002)	
PF13348	Y_phosphatase3C	Tyrosine phosphatase family C-terminal region	
PF13587	DJ-1_PfpI_N	N-terminal domain of DJ-1_PfpI family	
PF13598	DUF4139	Domain of unknown function (DUF4139)	
PF13600	DUF4140	N-terminal domain of unknown function (DUF4140)	
PF13960	DUF4218	Domain of unknown function (DUF4218)	
PF14113	DUF4285	Domain of unknown function (DUF4285)	
PF14124	DUF4291	Domain of unknown function (DUF4291)	
PF14240	YHYH	YHYH protein	

Supplementary Table 20. Ancient domain types preserved in vertebrates but lost in amphioxus

There are a total of 122 ancient protein domain types that are preserved in at least one of six examined vertebrates (tetraodon, zebrafish, xenopus, chicken, mouse and human) but lost in both lancelet species.
* All possible protein isoforms of a species were used to calculate the domain type number.
** No E-value cutoff is applied here.

pfamID	pfamName	pfamDesc	clanID
PF08204	V-set_CD47	CD47 immunoglobulin-like domain	CL0011
PF11700	ATG22	Vacuole effluxer Atg22 like	CL0015
PF06293	Kdo	Lipopolysaccharide kinase (Kdo/WaaP) family	CL0016
PF10037	MRP-S27	Mitochondrial 28S ribosomal protein S27	CL0020
PF13431	TPR_17	Tetratricopeptide repeat	CL0020
PF02689	Herpes_Helicase	Helicase	CL0023
PF07517	SecA_DEAD	SecA DEAD-like domain	CL0023
PF13173	AAA_14	AAA domain	CL0023
PF13245	AAA_19	Part of AAA domain	CL0023
PF09757	Arb2	Arb2 domain	CL0028
PF02525	Flavodoxin_2	Flavodoxin-like fold	CL0042
PF02551	Acyl_CoA_thio	Acyl-CoA thioesterase	CL0050
PF13622	4HBT_3	Thioesterase-like superfamily	CL0050
PF13522	GATase_6	Glutamine amidotransferase domain	CL0052
PF05270	AbfB	Alpha-L-arabinofuranosidase B (ABFB)	CL0066
PF13841	Defensin_beta_2	Beta defensin	CL0075
PF01129	ART	NAD:arginine ADP-ribosyltransferase	CL0084
PF13900	GVQW	Putative binding domain	CL0093
PF08210	APOBEC_N	APOBEC-like N-terminal domain	CL0109
PF01697	Glyco_transf_92	Glycosyltransferase family 92	CL0110
PF13231	PMT_2	Dolichyl-phosphate-mannose-protein mannosyltransferase	CL0111
PF08100	Dimerisation	Dimerisation domain	CL0123
PF09607	BrkDBD	Brinker DNA-binding domain	CL0123
PF13518	HTH_28	Helix-turn-helix domain	CL0123
PF13551	HTH_29	Winged helix-turn helix	CL0123
PF13565	HTH_32	Homeodomain-like domain	CL0123
PF13873	Myb_DNA-bind_5	Myb/SANT-like DNA-binding domain	CL0123
PF09342	DUF1986	Domain of unknown function (DUF1986)	CL0124
PF13582	Reprolysin_3	Metallo-peptidase family M12B Reprolysin-like	CL0126
PF13975	gag-asp_proteas	gag-polyprotein putative aspartyl protease	CL0129
PF00302	CAT	Chloramphenicol acetyltransferase	CL0149
PF13287	Fn3_assoc	Fn3 associated	CL0159
PF13290	CHB_HEX_C_1	Chitobiase/beta-hexosaminidase C-terminal domain	CL0159
PF06827	zf-FPG_IleRS	Zinc finger found in FPG and IleRS	CL0167
PF12826	HHH_2	Helix-hairpin-helix motif	CL0198
PF08562	Crisp	Crisp	CL0213
PF01761	DHQ_synthase	3-dehydroquinate synthase	CL0224
PF01126	Heme_oxygenase	Heme oxygenase	CL0230

pfamID	pfamName	pfamDesc	clanID
PF00895	ATP-synt_8	ATP synthase protein 8	CL0255
PF09190	DALR_2	DALR domain	CL0258
PF12814	Mcp5_PH	Meiotic cell cortex C-terminal pleckstrin homology	CL0266
PF07786	DUF1624	Protein of unknown function (DUF1624)	CL0316
PF03176	MMPL	MMPL family	CL0322
PF13631	Cytochrom_B_N_2	Cytochrome b(N-terminal)/b6/petB	CL0328
PF13394	Fer4_14	4Fe-4S single cluster domain	CL0344
PF12907	zf-met2	Zinc-binding	CL0361
PF07967	zf-C3HC	C3HC zinc finger-like	CL0417
PF08600	Rsm1	Rsm1-like	CL0417
PF12328	Rpp20	Rpp20 subunit of nuclear RNase MRP and P	CL0441
PF00131	Metallothio	Metallothionein	CL0461
PF14392	zf-CCHC_4	Zinc knuckle	CL0511
PF00029	Connexin	Connexin	
PF00159	Hormone_3	Pancreatic hormone peptide	
PF00471	Ribosomal_L33	Ribosomal protein L33	
PF00473	CRF	Corticotropin-releasing factor family	
PF00525	Crystallin	Alpha crystallin A chain, N terminal	
PF00830	Ribosomal_L28	Ribosomal L28 family	
PF00832	Ribosomal_L39	Ribosomal L39 protein	
PF02060	ISK_Channel	Slow voltage-gated potassium channel	
PF02130	UPF0054	Uncharacterized protein family UPF0054	
PF02151	UVR	UvrB/uvrC motif	
PF02161	Prog_receptor	Progesterone receptor	
PF02944	BESS	BESS motif	
PF03066	Nucleoplasmin	Nucleoplasmin	
PF03762	VOMI	Vitelline membrane outer layer protein I (VOMI)	
PF04305	DUF455	Protein of unknown function (DUF455)	
PF04572	Gb3_synth	Alpha 1,4-glycosyltransferase conserved region	
PF04721	DUF750	Domain of unknown function (DUF750)	
PF04724	Glyco_transf_17	Glycosyltransferase family 17	
PF04856	Securin	Securin sister-chromatid separation inhibitor	
PF04988	AKAP95	A-kinase anchoring protein 95 (AKAP95)	
PF05162	Ribosomal_L41	Ribosomal protein L41	
PF05177	RCSD	RCSD region	
PF05391	Lsm_interact	Lsm interaction motif	
PF05428	CRF-BP	Corticotropin-releasing factor binding protein (CRF-BP)	
PF05461	ApoL	Apolipoprotein L	
PF05593	RHS_repeat	RHS Repeat	
PF05612	DUF781	Mouse protein of unknown function (DUF781)	
PF06140	Ifi-6-16	Interferon-induced 6-16 family	
PF06369	Anemone_cytotox	Sea anemone cytotoxic protein	
PF06495	Transformer	Fruit fly transformer protein	
PF06617	M-inducer_phosp	M-phase inducer phosphatase	
PF06637	PV-1	PV-1 protein (PLVAP)	

pfamID	pfamName	pfamDesc	clanID
PF06954	Resistin	Resistin	
PF07160	DUF1395	Protein of unknown function (DUF1395)	
PF07382	HC2	Histone H1-like nucleoprotein HC2	
PF07558	Shugoshin_N	Shugoshin N-terminal coiled-coil region	
PF07896	DUF1674	Protein of unknown function (DUF1674)	
PF07940	Hepar_II_III	Heparinase II/III-like protein	
PF08038	Tom7	TOM7 family	
PF08168	NUC205	NUC205 domain	
PF08202	Mis12_component	Mis12-Mtw1 protein family	
PF08213	DUF1713	Mitochondrial domain of unknown function (DUF1713)	
PF08367	M16C_assoc	Peptidase M16C associated	
PF08374	Protocadherin	Protocadherin	
PF09036	Bcr-Abl_Oligo	Bcr-Abl oncoprotein oligomerisation domain	
PF09166	Biliv-reduc_cat	Biliverdin reductase, catalytic	
PF09263	PEX-2N	Peroxisome biogenesis factor 1, N-terminal	
PF09649	CHZ	Histone chaperone domain CHZ	
PF09666	Sororin	Sororin protein	
PF10344	Fmp27	Mitochondrial protein from FMP27	
PF10359	Fmp27_WPPW	RNA pol II promoter Fmp27 protein domain	
PF10486	PI3K_1B_p101	Phosphoinositide 3-kinase gamma adapter protein p101 subunit	
PF10488	PP1c_bdg	Phosphatase-1 catalytic subunit binding region	
PF10492	Nrf1_activ_bdg	Nrf1 activator activation site binding domain	
PF10578	SVS_QK	Seminal vesicle protein repeat	
PF10582	Connexin_CCC	Gap junction channel protein cysteine-rich domain	
PF11176	DUF2962	Protein of unknown function (DUF2962)	
PF11244	Med25_NR-box	Mediator complex subunit 25 C-terminal NR box-containing	
PF11380	DUF3184	Protein of unknown function (DUF3184)	
PF11413	HIF-1	Hypoxia-inducible factor-1	
PF11901	DUF3421	Protein of unknown function (DUF3421)	
PF12129	Phtf-FEM1B_bdg	Male germ-cell putative homeodomain transcription factor	
PF12162	STAT1_TAZ2bind	STAT1 TAZ2 binding domain	
PF12413	DLL_N	Homeobox protein distal-less-like N terminal	
PF12417	DUF3669	Zinc finger protein	
PF12443	AKNA	AT-hook-containing transcription factor	
PF12610	SOCS	Suppressor of cytokine signalling	
PF12938	M_domain	M domain of GW182	
PF13094	CENP-Q	CENP-A-nucleosome distal (CAD) centromere subunit	
PF13902	R3H-assoc	R3H-associated N-terminal domain	
PF14047	DCR	Dppa2/4 conserved region	

Supplementary Table 21. Novel domain pairs shared between two lancelet species

domainIDs	domainDesc
PF04389;PF01549	Peptidase_M28;ShK
PF00041;PF00531	fn3;Death
PF12796;PF13516	Ank_2;LRR_6
PF13424;PF00651	TPR_12;BTB
PF00059;PF03098	Lectin_C;An_peroxidase
PF00754;PF03142	F5_F8_type_C;Chitin_synth_2
PF03142;PF01822	Chitin_synth_2;WSC
PF13465;PF00168	zf-H2C2_2;C2
PF00093;PF00041	VWC;fn3
PF00046;PF00096	Homeobox;zf-C2H2
PF13385;PF01094	Laminin_G_3;ANF_receptor
PF12661;PF00051	hEGF;Kringle
PF01483;PF00082	P_proprotein;Peptidase_S8
PF02338;PF00531	OTU;Death
PF00193;PF00057	Xlink;Ldl_recept_a
PF11878;PF14180	DUF3398;DOCK_C2
PF01826;PF13330	TIL;Mucin2_WxxW
PF01391;PF00008	Collagen;EGF
PF01826;PF00059	TIL;Lectin_C
PF12248;PF00754	Methyltransf_FA;F5_F8_type_C
PF00024;PF00431	PAN_1;CUB
PF00855;PF01388	PWWP;ARID
PF00084;PF01549	Sushi;ShK
PF01335;PF13765	DED;PRY
PF08477;PF02338	Miro;OTU
PF00629;PF00008	MAM;EGF
PF01066;PF01467	CDP-OH_P_transf;CTP_transf_2
PF00754;PF08685	F5_F8_type_C;GON
PF00069;PF07686	Pkinase;V-set
PF13385;PF00092	Laminin_G_3;VWA
PF00515;PF08336	TPR_1;P4Ha_N
PF13833;PF00400	EF_hand_6;WD40
PF00008;PF02010	EGF;REJ
PF01822;PF03142	WSC;Chitin_synth_2
PF01094;PF13385	ANF_receptor;Laminin_G_3
PF00350;PF07714	Dynamin_N;Pkinase_Tyr
PF00629;PF07645	MAM;EGF_CA
PF00147;PF00059	Fibrinogen_C;Lectin_C
PF00045;PF07690	Hemopexin;MFS_1
PF00090;PF00704	TSP_1;Glyco_hydro_18
PF00992;PF02494	Troponin;HYR
PF00024;PF13855	PAN_1;LRR_8
PF07679;PF00058	I-set;Ldl_recept_b
PF02822;PF01347	Antistasin;Vitellogenin_N
PF07679;PF13516	I-set;LRR_6
PF12662;PF00090	cEGF;TSP_1
PF00053;PF00057	Laminin_EGF;Ldl_recept_a
PF00354;PF00008	Pentaxin;EGF
PF13676;PF00270	TIR_2;DEAD
PF00619;PF00071	CARD;Ras
PF12248;PF00051	Methyltransf_FA;Kringle
PF00538;PF00125	Linker_histone;Histone
PF00362;PF07714	Integrin_beta;Pkinase_Tyr
PF12947;PF00092	EGF_3;VWA
PF00619;PF13676	CARD;TIR_2
PF00084;PF13517	Sushi;VCBS
PF00530;PF13330	SRCR;Mucin2_WxxW
PF00094;PF00008	VWD;EGF
PF12662;PF01390	cEGF;SEA
PF00531;PF12799	Death;LRR_4
PF01392;PF13895	Fz;Ig_2
PF00307;PF00397	CH;WW
PF00071;PF13676	Ras;TIR_2
PF00008;PF12248	EGF;Methyltransf_FA
PF12947;PF01390	EGF_3;SEA
PF00553;PF00759	CBM_2;Glyco_hydro_9
PF00397;PF00612	WW;IQ
PF03445;PF10335	DUF294;DUF294_C
PF00531;PF01436	Death;NHL
PF06119;PF00530	NIDO;SRCR
PF00530;PF07699	SRCR;GCC2_GCC3
PF00053;PF07714	Laminin_EGF;Pkinase_Tyr
PF02140;PF00754	Gal_Lectin;F5_F8_type_C
PF00531;PF02135	Death;zf-TAZ
PF01392;PF00629	Fz;MAM
PF00018;PF06625	SH3_1;DUF1151
PF00754;PF06462	F5_F8_type_C;Hyd_WA
PF00641;PF12773	zf-RanBP;DZR
PF02191;PF00014	OLF;Kunitz_BPTI
PF01230;PF00264	HIT;Tyrosinase
PF00051;PF00084	Kringle;Sushi
PF07051;PF00106	OCIA;adh_short
PF00534;PF08477	Glycos_transf_1;Miro
PF00566;PF00412	RabGAP-TBC;LIM
PF01663;PF01553	Phosphodiest;Acyltransferase
PF00059;PF00082	Lectin_C;Peptidase_S8
PF00030;PF00092	Crystall;VWA
PF01607;PF00008	CBM_14;EGF
PF12799;PF00791	LRR_4;ZU5
PF13385;PF00090	Laminin_G_3;TSP_1
PF01436;PF00169	NHL;PH
PF00629;PF12947	MAM;EGF_3
PF00038;PF10541	Filament;KASH
PF00069;PF00350	Pkinase;Dynamin_N
PF00531;PF01936	Death;NYN
PF01734;PF01477	Patatin;PLAT
PF12780;PF12781	AAA_8;AAA_9
PF00530;PF01392	SRCR;Fz
PF09248;PF01179	DUF1965;Cu_amine_oxid
PF00059;PF00629	Lectin_C;MAM
PF01266;PF13833	DAO;EF_hand_6
PF07593;PF00084	UnbV_ASPIC;Sushi
PF12796;PF00071	Ank_2;Ras
PF00059;PF07699	Lectin_C;GCC2_GCC3
PF00090;PF00100	TSP_1;Zona_pellucida
PF01549;PF00080	ShK;Sod_Cu
PF04142;PF04488	Nuc_sug_transp;Gly_transf_sug
PF13553;PF00534	FIIND;Glycos_transf_1
PF08477;PF00531	Miro;Death
PF07699;PF00530	GCC2_GCC3;SRCR
PF01347;PF02822	Vitellogenin_N;Antistasin
PF02911;PF00378	Formyl_trans_C;ECH
PF10162;PF00754	G8;F5_F8_type_C
PF12796;PF00534	Ank_2;Glycos_transf_1
PF05773;PF00097	RWD;zf-C3HC4
PF00350;PF00531	Dynamin_N;Death
PF13330;PF00093	Mucin2_WxxW;VWC
PF00094;PF12662	VWD;cEGF
PF12248;PF00090	Methyltransf_FA;TSP_1
PF13855;PF00754	LRR_8;F5_F8_type_C
PF03815;PF00059	LCCL;Lectin_C
PF13385;PF12661	Laminin_G_3;hEGF
PF13676;PF01462	TIR_2;LRRNT
PF01477;PF00868	PLAT;Transglut_N
PF12248;PF00629	Methyltransf_FA;MAM
PF00431;PF07699	CUB;GCC2_GCC3

PF12248;PF00147	Methyltransf_FA;Fibrinogen_C
PF01436;PF01335	NHL;DED
PF02014;PF00084	Reeler;Sushi
PF01392;PF00431	Fz;CUB
PF00498;PF00350	FHA;Dynamin_N
PF00059;PF00243	Lectin_C;NGF
PF00754;PF00193	F5_F8_type_C;Xlink
PF00534;PF00531	Glycos_transf_1;Death
PF00092;PF00093	VWA;VWC
PF07995;PF00014	GSDH;Kunitz_BPTI
PF00354;PF07645	Pentaxin;EGF_CA
PF00059;PF12947	Lectin_C;EGF_3
PF00041;PF00051	fn3;Kringle
PF01344;PF00651	Kelch_1;BTB
PF00051;PF00354	Kringle;Pentaxin
PF00024;PF00084	PAN_1;Sushi
PF00082;PF00084	Peptidase_S8;Sushi
PF02201;PF01253	SWIB;SUI1
PF00777;PF00754	Glyco_transf_29;F5_F8_type_C
PF01764;PF01926	Lipase_3;MMR_HSR1
PF13385;PF00354	Laminin_G_3;Pentaxin
PF00071;PF00619	Ras;CARD
PF00531;PF13365	Death;Trypsin_2
PF00536;PF00621	SAM_1;RhoGEF
PF04548;PF01079	AIG1;Hint
PF01826;PF06462	TIL;Hyd_WA
PF00240;PF11976	ubiquitin;Rad60-SLD
PF03028;PF00777	Dynein_heavy;Glyco_transf_29
PF07699;PF07645	GCC2_GCC3;EGF_CA
PF00619;PF13516	CARD;LRR_6
PF00754;PF13923	F5_F8_type_C;zf-C3HC4_2
PF00754;PF10162	F5_F8_type_C;G8
PF13414;PF13086	TPR_11;AAA_11
PF07885;PF03520	Ion_trans_2;KCNQ_channel
PF00059;PF13330	Lectin_C;Mucin2_WxxW
PF02822;PF00051	Antistasin;Kringle
PF00086;PF02191	Thyroglobulin_1;OLF
PF12947;PF00059	EGF_3;Lectin_C
PF07699;PF02494	GCC2_GCC3;HYR
PF00086;PF00059	Thyroglobulin_1;Lectin_C
PF01826;PF00531	TIL;Death
PF12248;PF01390	Methyltransf_FA;SEA
PF04089;PF00082	BRICHOS;Peptidase_S8
PF00199;PF01477	Catalase;PLAT
PF00051;PF02822	Kringle;Antistasin
PF13879;PF00246	KIAA1430;Peptidase_M14
PF07645;PF00020	EGF_CA;TNFR_c6
PF12796;PF00619	Ank_2;CARD
PF00514;PF13676	Arm;TIR_2
PF00051;PF02931	Kringle;Neur_chan_LBD
PF13820;PF01436	Nucleic_acid_bd;NHL
PF13676;PF02820	TIR_2;MBT
PF00059;PF12661	Lectin_C;hEGF
PF01390;PF00050	SEA;Kazal_1
PF00051;PF01421	Kringle;Reprolysin
PF01464;PF13539	SLT;Peptidase_M15_4
PF13895;PF00018	Ig_2;SH3_1
PF01822;PF12248	WSC;Methyltransf_FA
PF00008;PF06119	EGF;NIDO
PF13414;PF03445	TPR_11;DUF294
PF00059;PF00094	Lectin_C;VWD
PF12248;PF00088	Methyltransf_FA;Trefoil
PF08685;PF00051	GON;Kringle
PF07679;PF13855	I-set;LRR_8
PF03146;PF00090	NtA;TSP_1
PF00084;PF07679	Sushi;I-set
PF13330;PF00059	Mucin2_WxxW;Lectin_C
PF00531;PF00041	Death;fn3
PF00041;PF00534	fn3;Glycos_transf_1
PF01096;PF08711	TFIIS_C;Med26
PF00088;PF00051	Trefoil;Kringle
PF02014;PF00232	Reeler;Glyco_hydro_1
PF02014;PF13517	Reeler;VCBS
PF12773;PF00641	DZR;zf-RanBP
PF12248;PF01477	Methyltransf_FA;PLAT
PF01390;PF01663	SEA;Phosphodiest
PF13879;PF00191	KIAA1430;Annexin
PF00024;PF07645	PAN_1;EGF_CA
PF00057;PF00059	Ldl_recept_a;Lectin_C
PF01823;PF00754	MACPF;F5_F8_type_C
PF00531;PF00619	Death;CARD
PF01699;PF00024	Na_Ca_ex;PAN_1
PF00084;PF02931	Sushi;Neur_chan_LBD
PF00051;PF01833	Kringle;TIG
PF05375;PF00093	Pacifastin_I;VWC
PF00168;PF00305	C2;Lipoxygenase
PF01734;PF13499	Patatin;EF_hand_5
PF01392;PF01390	Fz;SEA
PF00641;PF12185	zf-RanBP;IR1-M
PF03133;PF12733	TTL;Cadherin-like
PF01472;PF02201	PUA;SWIB
PF03098;PF00059	An_peroxidase;Lectin_C
PF13855;PF07714	LRR_8;Pkinase_Tyr
PF00010;PF07714	HLH;Pkinase_Tyr
PF00147;PF02931	Fibrinogen_C;Neur_chan_LBD
PF00147;PF00041	Fibrinogen_C;fn3
PF00059;PF01391	Lectin_C;Collagen
PF13895;PF01549	Ig_2;ShK
PF07699;PF07679	GCC2_GCC3;I-set
PF01822;PF00754	WSC;F5_F8_type_C
PF02018;PF00331	CBM_4_9;Glyco_hydro_10
PF00086;PF00008	Thyroglobulin_1;EGF
PF12248;PF01822	Methyltransf_FA;WSC
PF01335;PF13424	DED;TPR_12
PF13385;PF00754	Laminin_G_3;F5_F8_type_C
PF00059;PF01822	Lectin_C;WSC
PF00093;PF05375	VWC;Pacifastin_I
PF00619;PF07679	CARD;I-set
PF00090;PF07699	TSP_1;GCC2_GCC3
PF00908;PF04321	dTDP_sugar_isom;RmlD_sub_bind
PF00112;PF08246	Peptidase_C1;Inhibitor_I29
PF12419;PF00176	DUF3670;SNF2_N
PF03351;PF00008	DOMON;EGF
PF13855;PF00024	LRR_8;PAN_1
PF00043;PF09793	GST_C;AD
PF07978;PF00027	NIPSNAP;cNMP_binding
PF00059;PF01400	Lectin_C;Astacin
PF00355;PF00848	Rieske;Ring_hydroxyl_A
PF07645;PF00094	EGF_CA;VWD
PF00431;PF00051	CUB;Kringle
PF02014;PF02793	Reeler;HRM
PF00754;PF01822	F5_F8_type_C;WSC
PF07714;PF00084	Pkinase_Tyr;Sushi
PF13519;PF00533	VWA_2;BRCT
PF10408;PF00179	Ufd2P_core;UQ_con
PF13410;PF13417	GST_C_2;GST_N_3
PF12248;PF03607	Methyltransf_FA;DCX
PF00531;PF00534	Death;Glycos_transf_1
PF11569;PF00046	Homez;Homeobox
PF01607;PF00059	CBM_14;Lectin_C

PF07699;PF00059	GCC2_GCC3;Lectin_C
PF03901;PF02931	Glyco_transf_22;Neur_chan_LBD
PF00041;PF00211	fn3;Guanylate_cyc
PF07645;PF01049	EGF_CA;Cadherin_C
PF01436;PF02140	NHL;Gal_Lectin
PF00629;PF04564	MAM;U-box
PF00754;PF13330	F5_F8_type_C;Mucin2_WxxW
PF00023;PF00619	Ank;CARD
PF00354;PF02793	Pentaxin;HRM
PF08397;PF00169	IMD;PH
PF00531;PF13516	Death;LRR_6
PF00040;PF00431	fn2;CUB
PF01390;PF00040	SEA;fn2
PF08477;PF00656	Miro;Peptidase_C14
PF02140;PF00051	Gal_Lectin;Kringle
PF00090;PF00530	TSP_1;SRCR
PF00362;PF00041	Integrin_beta;fn3
PF00520;PF12796	Ion_trans;Ank_2
PF02140;PF01436	Gal_Lectin;NHL
PF00092;PF00147	VWA;Fibrinogen_C
PF02494;PF07679	HYR;I-set
PF00629;PF12248	MAM;Methyltransf_FA
PF00041;PF00536	fn3;SAM_1
PF00057;PF00754	Ldl_recept_a;F5_F8_type_C
PF07707;PF00651	BACK;BTB
PF00531;PF13855	Death;LRR_8
PF00536;PF00130	SAM_1;C1_1
PF00629;PF01392	MAM;Fz
PF00059;PF02931	Lectin_C;Neur_chan_LBD
PF00534;PF01436	Glycos_transf_1;NHL
PF03098;PF00354	An_peroxidase;Pentaxin
PF06468;PF00041	Spond_N;fn3
PF00008;PF03567	EGF;Sulfotransfer_2
PF12847;PF13855	Methyltransf_18;LRR_8
PF00515;PF00023	TPR_1;Ank
PF01823;PF00431	MACPF;CUB
PF01392;PF07645	Fz;EGF_CA
PF00097;PF00630	zf-C3HC4;Filamin
PF00090;PF01822	TSP_1;WSC
PF00090;PF09717	TSP_1;CPW_WPC
PF00092;PF00530	VWA;SRCR
PF07719;PF05843	TPR_2;Suf
PF00754;PF03815	F5_F8_type_C;LCCL
PF01392;PF00084	Fz;Sushi
PF12721;PF00107	RHIM;ADH_zinc_N
PF13360;PF01011	PQQ_2;PQQ
PF00024;PF01161	PAN_1;PBP
PF00084;PF00050	Sushi;Kazal_1
PF08016;PF07645	PKD_channel;EGF_CA
PF00560;PF00084	LRR_1;Sushi
PF00431;PF06462	CUB;Hyd_WA
PF01607;PF00084	CBM_14;Sushi
PF00754;PF02010	F5_F8_type_C;REJ
PF00350;PF00534	Dynamin_N;Glycos_transf_1
PF09837;PF00535	DUF2064;Glycos_transf_2
PF00057;PF00193	Ldl_recept_a;Xlink
PF00028;PF00100	Cadherin;Zona_pellucida
PF00051;PF08685	Kringle;GON
PF03445;PF13424	DUF294;TPR_12
PF00001;PF00566	7tm_1;RabGAP-TBC
PF00068;PF00092	Phospholip_A2_1;VWA
PF00057;PF06008	Ldl_recept_a;Laminin_I
PF00188;PF01391	CAP;Collagen
PF00084;PF02932	Sushi;Neur_chan_memb
PF13632;PF00536	Glyco_trans_2_3;SAM_1
PF10609;PF02140	ParA;Gal_Lectin
PF00057;PF00092	Ldl_recept_a;VWA
PF01436;PF10282	NHL;Lactonase
PF07679;PF01607	I-set;CBM_14
PF00084;PF00041	Sushi;fn3
PF07686;PF00093	V-set;VWC
PF00059;PF01826	Lectin_C;TIL
PF13516;PF00531	LRR_6;Death
PF00059;PF00001	Lectin_C;7tm_1
PF00622;PF00658	SPRY;PABP
PF13385;PF00530	Laminin_G_3;SRCR
PF13424;PF03445	TPR_12;DUF294
PF01607;PF13414	CBM_14;TPR_11
PF00531;PF08357	Death;SEFIR
PF00084;PF00090	Sushi;TSP_1
PF03815;PF00530	LCCL;SRCR
PF02338;PF00534	OTU;Glycos_transf_1
PF04261;PF02901	Dyp_perox;PFL
PF12126;PF01436	DUF3583;NHL
PF03024;PF00001	Folate_rec;7tm_1
PF00538;PF03359	Linker_histone;GKAP
PF00245;PF00531	Alk_phosphatase;Death
PF00530;PF00386	SRCR;C1q
PF00041;PF03815	fn3;LCCL
PF00520;PF07885	Ion_trans;Ion_trans_2
PF01822;PF06462	WSC;Hyd_WA
PF00002;PF13330	7tm_2;Mucin2_WxxW
PF01033;PF03098	Somatomedin_B;An_peroxidase
PF02014;PF01390	Reeler;SEA
PF02820;PF13855	MBT;LRR_8
PF01661;PF13923	Macro;zf-C3HC4_2
PF13855;PF00059	LRR_8;Lectin_C
PF14295;PF00754	PAN_4;F5_F8_type_C
PF13553;PF08477	FIIND;Miro
PF14295;PF12248	PAN_4;Methyltransf_FA
PF02845;PF09038	CUE;53-BP1_Tudor
PF00059;PF08685	Lectin_C;GON
PF07645;PF06119	EGF_CA;NIDO
PF06119;PF12662	NIDO;cEGF
PF08336;PF00515	P4Ha_N;TPR_1
PF01823;PF00008	MACPF;EGF
PF00400;PF02239	WD40;Cytochrom_D1
PF02946;PF00385	GTF2I;Chromo
PF08685;PF00754	GON;F5_F8_type_C
PF03098;PF00754	An_peroxidase;F5_F8_type_C
PF03815;PF00051	LCCL;Kringle
PF08826;PF00780	DMPK_coil;CNH
PF00534;PF13424	Glycos_transf_1;TPR_12
PF00975;PF00668	Thioesterase;Condensation
PF03445;PF13176	DUF294;TPR_7
PF00560;PF00531	LRR_1;Death
PF00051;PF07645	Kringle;EGF_CA
PF00084;PF00581	Sushi;Rhodanese
PF00619;PF00041	CARD;fn3
PF01094;PF00060	ANF_receptor;Lig_chan
PF00644;PF00533	PARP;BRCT
PF01392;PF00530	Fz;SRCR
PF00531;PF00071	Death;Ras
PF00536;PF07714	SAM_1;Pkinase_Tyr
PF00188;PF00041	CAP;fn3
PF00858;PF00754	ASC;F5_F8_type_C
PF00040;PF01390	fn2;SEA
PF00041;PF07974	fn3;EGF_2
PF12248;PF00041	Methyltransf_FA;fn3
PF00643;PF02931	zf-B_box;Neur_chan_LBD

PF13639;PF06803	zf-RING_2;DUF1232
PF13519;PF06701	VWA_2;MIB_HERC2
PF00621;PF00791	RhoGEF;ZU5
PF12012;PF00589	DUF3504;Phage_integrase
PF00100;PF01390	Zona_pellucida;SEA
PF00754;PF03098	F5_F8_type_C;An_peroxidase
PF00041;PF13385	fn3;Laminin_G_3
PF13385;PF02140	Laminin_G_3;Gal_Lectin
PF14259;PF01753	RRM_6;zf-MYND
PF00084;PF07714	Sushi;Pkinase_Tyr
PF01826;PF12661	TIL;hEGF
PF00088;PF00041	Trefoil;fn3
PF00057;PF02822	Ldl_recept_a;Antistasin
PF03142;PF07647	Chitin_synth_2;SAM_2
PF00057;PF00053	Ldl_recept_a;Laminin_EGF
PF06312;PF00652	Neurexophilin;Ricin_B_lectin
PF00020;PF07645	TNFR_c6;EGF_CA
PF00531;PF04116	Death;FA_hydroxylase
PF00437;PF12775	T2SE;AAA_7
PF00622;PF00531	SPRY;Death
PF07648;PF00008	Kazal_2;EGF
PF00619;PF00622	CARD;SPRY
PF00059;PF00354	Lectin_C;Pentaxin
PF13855;PF11930	LRR_8;DUF3448
PF03445;PF00515	DUF294;TPR_1
PF01401;PF00057	Peptidase_M2;Ldl_recept_a
PF00041;PF07645	fn3;EGF_CA
PF00059;PF12248	Lectin_C;Methyltransf_FA
PF00307;PF11971	CH;CAMSAP_CH
PF13855;PF00084	LRR_8;Sushi
PF00057;PF13908	Ldl_recept_a;Shisa
PF09294;PF00041	Interfer-bind;fn3
PF01392;PF02931	Fz;Neur_chan_LBD
PF12931;PF07304	Sec16_C;SRA1
PF00051;PF00858	Kringle;ASC
PF10573;PF02854	UPF0561;MIF4G
PF06424;PF13428	PRP1_N;TPR_14
PF00050;PF00014	Kazal_1;Kunitz_BPTI
PF07645;PF01477	EGF_CA;PLAT
PF13893;PF03399	RRM_5;SAC3_GANP
PF05827;PF01299	ATP-synt_S1;Lamp
PF00530;PF00090	SRCR;TSP_1
PF00043;PF00059	GST_C;Lectin_C
PF01753;PF13181	zf-MYND;TPR_8
PF00086;PF00093	Thyroglobulin_1;VWC
PF00628;PF13508	PHD;Acetyltransf_7
PF00704;PF00090	Glyco_hydro_18;TSP_1
PF02671;PF13921	PAH;Myb_DNA-bind_6
PF01663;PF00094	Phosphodiest;VWD
PF13895;PF00053	Ig_2;Laminin_EGF
PF07645;PF00090	EGF_CA;TSP_1
PF10579;PF13424	Rapsyn_N;TPR_12
PF00957;PF07732	Synaptobrevin;Cu-oxidase_3
PF08477;PF00619	Miro;CARD
PF00053;PF00629	Laminin_EGF;MAM
PF05132;PF00619	RNA_pol_Rpc4;CARD
PF05903;PF08324	DUF862;PUL
PF00531;PF13553	Death;FIIND
PF00051;PF01033	Kringle;Somatomedin_B
PF00643;PF08450	zf-B_box;SGL
PF06462;PF00057	Hyd_WA;Ldl_recept_a
PF01885;PF00001	PTS_2-RNA;7tm_1
PF00350;PF00069	Dynamin_N;Pkinase
PF00051;PF12248	Kringle;Methyltransf_FA
PF00071;PF00041	Ras;fn3
PF06462;PF00754	Hyd_WA;F5_F8_type_C
PF13202;PF08976	EF_hand_3;DUF1880
PF01607;PF00089	CBM_14;Trypsin
PF01663;PF00149	Phosphodiest;Metallophos
PF13923;PF12126	zf-C3HC4_2;DUF3583
PF00400;PF00071	WD40;Ras
PF05375;PF00086	Pacifastin_I;Thyroglobulin_1
PF13330;PF12248	Mucin2_WxxW;Methyltransf_FA
PF12248;PF00084	Methyltransf_FA;Sushi
PF00001;PF02210	7tm_1;Laminin_G_2
PF05183;PF13086	RdRP;AAA_11
PF00090;PF13385	TSP_1;Laminin_G_3
PF00069;PF13465	Pkinase;zf-H2C2_2
PF00008;PF00229	EGF;TNF
PF00753;PF12706	Lactamase_B;Lactamase_B_2
PF00046;PF12403	Homeobox;Pax2_C
PF12146;PF12697	Hydrolase_4;Abhydrolase_6
PF08016;PF13855	PKD_channel;LRR_8
PF00041;PF02010	fn3;REJ
PF00046;PF00001	Homeobox;7tm_1
PF00656;PF00531	Peptidase_C14;Death
PF03445;PF13374	DUF294;TPR_10
PF00855;PF10497	PWWP;zf-4CXXC_R1
PF00530;PF00754	SRCR;F5_F8_type_C
PF02140;PF06101	Gal_Lectin;DUF946
PF00061;PF02822	Lipocalin;Antistasin
PF02743;PF01607	Cache_1;CBM_14
PF00058;PF02494	Ldl_recept_b;HYR
PF02140;PF01822	Gal_Lectin;WSC
PF03142;PF00536	Chitin_synth_2;SAM_1
PF00514;PF07819	Arm;PGAP1
PF00057;PF01390	Ldl_recept_a;SEA
PF00057;PF00051	Ldl_recept_a;Kringle
PF01335;PF02338	DED;OTU
PF13330;PF00092	Mucin2_WxxW;VWA
PF00619;PF13895	CARD;Ig_2
PF00514;PF04969	Arm;CS
PF00104;PF00105	Hormone_recep;zf-C4
PF00023;PF08477	Ank;Miro
PF07677;PF07703	A2M_recep;A2M_N_2
PF01823;PF00051	MACPF;Kringle
PF13855;PF13465	LRR_8;zf-H2C2_2
PF00069;PF00067	Pkinase;p450
PF01822;PF00051	WSC;Kringle
PF00534;PF00619	Glycos_transf_1;CARD
PF00852;PF01370	Glyco_transf_10;Epimerase
PF08969;PF00632	DUF1873;HECT
PF13855;PF00089	LRR_8;Trypsin
PF07645;PF02010	EGF_CA;REJ
PF00188;PF00001	CAP;7tm_1
PF13347;PF00899	MFS_2;ThiF
PF00023;PF00560	Ank;LRR_1
PF07699;PF02412	GCC2_GCC3;TSP_3
PF00067;PF01477	p450;PLAT
PF00038;PF00652	Filament;Ricin_B_lectin
PF03770;PF02014	IPK;Reeler
PF00059;PF00053	Lectin_C;Laminin_EGF
PF00248;PF13360	Aldo_ket_red;PQQ_2
PF13895;PF07653	Ig_2;SH3_2
PF06462;PF00059	Hyd_WA;Lectin_C
PF01549;PF01400	ShK;Astacin
PF08685;PF00059	GON;Lectin_C
PF00533;PF12796	BRCT;Ank_2
PF07645;PF03815	EGF_CA;LCCL
PF00069;PF00743	Pkinase;FMO-like

PF00431;PF12248	CUB;Methyltransf_FA
PF00643;PF12810	zf-B_box;Gly_rich
PF00530;PF00229	SRCR;TNF
PF08441;PF00362	Integrin_alpha2;Integrin_beta
PF00858;PF00051	ASC;Kringle
PF12799;PF00531	LRR_4;Death
PF05922;PF00059	Inhibitor_I9;Lectin_C
PF06682;PF00008	DUF1183;EGF
PF00651;PF08938	BTB;DUF1916
PF12248;PF00094	Methyltransf_FA;VWD
PF03062;PF01253	MBOAT;SUI1
PF13330;PF12947	Mucin2_WxxW;EGF_3
PF00088;PF00059	Trefoil;Lectin_C
PF01207;PF05142	Dus;DUF702
PF00431;PF13385	CUB;Laminin_G_3
PF00008;PF13882	EGF;Bravo_FIGEY
PF00008;PF00629	EGF;MAM
PF00041;PF00619	fn3;CARD
PF00754;PF00051	F5_F8_type_C;Kringle
PF06119;PF00059	NIDO;Lectin_C
PF12799;PF13306	LRR_4;LRR_5
PF01347;PF00754	Vitellogenin_N;F5_F8_type_C
PF00041;PF08016	fn3;PKD_channel
PF00629;PF00090	MAM;TSP_1
PF00001;PF00059	7tm_1;Lectin_C
PF00354;PF00754	Pentaxin;F5_F8_type_C
PF00193;PF00088	Xlink;Trefoil
PF01335;PF02263	DED;GBP
PF01822;PF02140	WSC;Gal_Lectin
PF01826;PF12248	TIL;Methyltransf_FA
PF09717;PF00431	CPW_WPC;CUB
PF13676;PF00931	TIR_2;NB-ARC
PF03445;PF07719	DUF294;TPR_2
PF00046;PF13926	Homeobox;DUF4211
PF00051;PF02140	Kringle;Gal_Lectin
PF13424;PF00531	TPR_12;Death
PF01841;PF07732	Transglut_core;Cu-oxidase_3
PF12796;PF00041	Ank_2;fn3
PF00627;PF13893	UBA;RRM_5
PF13088;PF00091	BNR_2;Tubulin
PF07699;PF13385	GCC2_GCC3;Laminin_G_3
PF00051;PF02932	Kringle;Neur_chan_memb
PF07645;PF12248	EGF_CA;Methyltransf_FA
PF00135;PF08376	COesterase;NIT
PF00059;PF13855	Lectin_C;LRR_8
PF00147;PF07679	Fibrinogen_C;I-set
PF06462;PF00092	Hyd_WA;VWA
PF13176;PF13432	TPR_7;TPR_16
PF07686;PF00041	V-set;fn3
PF00619;PF13365	CARD;Trypsin_2
PF03134;PF13868	TB2_DP1_HVA22;Trichoplein
PF00515;PF01582	TPR_1;TIR
PF01549;PF01390	ShK;SEA
PF00024;PF12661	PAN_1;hEGF
PF02932;PF00431	Neur_chan_memb;CUB
PF00041;PF00754	fn3;F5_F8_type_C
PF08016;PF02010	PKD_channel;REJ
PF00059;PF03137	Lectin_C;OATP
PF02135;PF12799	zf-TAZ;LRR_4
PF13385;PF01822	Laminin_G_3;WSC
PF01822;PF00722	WSC;Glyco_hydro_16
PF03815;PF00193	LCCL;Xlink
PF00754;PF12248	F5_F8_type_C;Methyltransf_FA
PF13621;PF00248	Cupin_8;Aldo_ket_red
PF00059;PF01390	Lectin_C;SEA
PF03298;PF01464	Stanniocalcin;SLT
PF00240;PF00111	ubiquitin;Fer2
PF00051;PF07679	Kringle;I-set
PF00106;PF00067	adh_short;p450
PF00051;PF00002	Kringle;7tm_2
PF00002;PF00020	7tm_2;TNFR_c6
PF00060;PF00535	Lig_chan;Glycos_transf_2
PF00100;PF00041	Zona_pellucida;fn3
PF01822;PF13385	WSC;Laminin_G_3
PF12248;PF00059	Methyltransf_FA;Lectin_C
PF13489;PF00067	Methyltransf_23;p450
PF00023;PF00071	Ank;Ras
PF00536;PF13771	SAM_1;zf-HC5HC2H
PF01335;PF13820	DED;Nucleic_acid_bd
PF00084;PF00051	Sushi;Kringle
PF13676;PF00531	TIR_2;Death
PF12714;PF00059	TILa;Lectin_C
PF00071;PF00531	Ras;Death
PF00754;PF01825	F5_F8_type_C;GPS
PF00014;PF13927	Kunitz_BPTI;Ig_3
PF01390;PF03098	SEA;An_peroxidase
PF00406;PF00240	ADK;ubiquitin
PF00001;PF00084	7tm_1;Sushi
PF00094;PF02140	VWD;Gal_Lectin
PF13465;PF02348	zf-H2C2_2;CTP_transf_3
PF00536;PF00788	SAM_1;RA
PF01436;PF06739	NHL;SBBP
PF01335;PF00619	DED;CARD
PF07679;PF07653	I-set;SH3_2
PF13895;PF13676	Ig_2;TIR_2
PF01392;PF02822	Fz;Antistasin
PF01823;PF07699	MACPF;GCC2_GCC3
PF13895;PF01390	Ig_2;SEA
PF13164;PF13499	DUF4002;EF_hand_5
PF12248;PF07645	Methyltransf_FA;EGF_CA
PF00090;PF02931	TSP_1;Neur_chan_LBD
PF00089;PF00008	Trypsin;EGF
PF00084;PF12662	Sushi;cEGF
PF00567;PF00514	TUDOR;Arm
PF00619;PF00643	CARD;zf-B_box
PF00397;PF00017	WW;SH2
PF07645;PF00354	EGF_CA;Pentaxin
PF00067;PF00531	p450;Death
PF10283;PF00644	zf-CCHH;PARP
PF01826;PF01390	TIL;SEA
PF00619;PF08477	CARD;Miro
PF13465;PF00059	zf-H2C2_2;Lectin_C
PF13465;PF00617	zf-H2C2_2;RasGEF
PF00354;PF00059	Pentaxin;Lectin_C
PF00090;PF01607	TSP_1;CBM_14
PF02010;PF08016	REJ;PKD_channel
PF13855;PF01259	LRR_8;SAICAR_synt
PF00058;PF00059	Ldl_recept_b;Lectin_C
PF13374;PF07719	TPR_10;TPR_2
PF01826;PF00431	TIL;CUB
PF12947;PF00041	EGF_3;fn3
PF12661;PF07714	hEGF;Pkinase_Tyr

Supplementary Table 22. The most promiscuous domains in novel domain pairs on different lineages

*See Supplementary Table 23 for the naming of lineage.

B. floridae only	Domain pair count	B. belcheri only	Domain pair count	B. bcheri_&_B. floridae	Domain pair count	S. purpuratus only	Domain pair count
LRR_8	47	Lectin_C	52	Lectin_C	45	SRCR	27
Lectin_C	34	LRR_8	35	F5_F8_type_C	32	7tm_1	25
7tm_1	34	zf-H2C2_2	33	Death	30	Ank_2	22
F5_F8_type_C	29	Ank_2	30	Kringle	30	HYR	20
WD40	27	Kringle	30	fn3	29	fn3	20
CUB	24	fn3	29	Methyltransf_FA	26	EGF	18
Pkinase_Tyr	23	Fibrinogen_C	27	Sushi	24	CUB	14
Pkinase	23	F5_F8_type_C	25	EGF_CA	20	F5_F8_type_C	12
Death	22	Pkinase_Tyr	24	CARD	19	WD40	11
Glyco_transf_2	22	Gal_Lectin	24	EGF	18	7tm_2	10
p450	21	Death	23	TSP_1	18	ZU5	8
NHL	20	Sushi	22	LRR_8	16	Trypsin	8
EGF_CA/EGF	19/19	Pkinase	22	SEA	16	zf-B_box	8
TPR_12	19	EGF_CA	22	WSC	15	Ig_2	8
I-set	18	Glycos_transf_	22	Laminin_G_3	15	Lectin_C	8
Fibrinogen_C	18	CUB	20	Ldl_recept_a	14	NACHT	7
zf-C3HC4_2	17	7tm_1	19	SRCR	14	Death	7
TIR_2/TIR	17/8	zf-B_box	19	CUB	12	Pkinase_Tyr	7
zf-H2C2_2	16	I-set	19	MAM	12	CAP	6
Gal-3-0_sulfotr	16	p450	19	GCC2_GCC3	12	GPS	6
Ig_2	15	Glyco_transf_2	18	I-set	11	Ldl_recept_b	6
DED	15	NHL	18	Fz	11	TPR_1	6
fn3	14	CARD	18	Glycos_transf_1	11	I-set	6
Collagen	14	EGF	17	Gal_Lectin	11	LRR_8	6
MFS_1	14	Gal-3-0_sulfotr	17	NHL	10	GTP_EFTU	5
Ldl_recept_a	13	BTB	17	VWA	10	Peptidase_S8	5
zf-B_box	13	Ig_2	16	Mucin2_WxxW	10	UDPGT	5
PKD_channel	12	EGF_3	16	Pentaxin	10	Ion_trans	5
VWA	11	TSP_1	16	7tm_1	9	zf-C3HC4	5
Ank_2	11	SRCR	15	Pkinase_Tyr	9	Ank_4	5
Sushi	11	WD40	15	TIR_2	9	EF_hand_5	5
BTB	11	Methyltransf_F	15	Ras	9	EGF_3	5
MAM	11	Neur_chan_LB	14	TIL	9	Ank	5
Dynamin_N	11	PAN_1	14	Ig_2	8	Gal_Lectin	5
V-set	11	Ras	14	DUF294	8	p450	5
Neur_chan_me	11	Mucin2_Wxx	14	Neur_chan_LBD	8	RRM_1	5
COesterase	11	Pentaxin	14	PAN_1	8	SH3_1	5
SH3_1	10	SEA	14	Hyd_WA	8	zf-C3HC4_2	5
CARD	10	LCCL	13	CBM_14	8	zf-H2C2_2	5
zf-C2H2	10	Cadherin	13	SAM_1	8	Sushi	5
Cadherin	10	MFS_1	12	Miro	8	Aminotran_1_2	4
TSP_1	9	WSC	11	TPR_12	7	Astacin	4
SRCR	9	adh_short	11	DED	7	Peptidase_C1	4
Mucin2_WxxW	9	PKD_channel	11	Ank_2	7	Galactosyl_T	4
7tm_2	9	cEGF	11	EGF_3	7	zf-C2H2_4	4
Kringle	8	TIR_2/TIR	20	VWD	7	SET	4
WSC	8	Laminin_G_3	11	An_peroxidase	7	Zona_pellucida	4
Fz	8	Ank_4	10	VWC	7	C2	4
Methyltransf_F	8	zf-C3HC4_2	10	LCCL	7	zf-RING_2	4
Neur_chan_LB	8	Kazal_2	10	Antistasin	7	MFS_1	4
Glycos_transf_1	8	fn2	10	Pkinase	6	Neur_chan_LBD	4
Kelch_1	8	Ldl_recept_a	10	Fibrinogen_C	6	PAN_1	4
PAN_1	7	VWA	10	zf-H2C2_2	6	WSC	4
NACHT	7	VWC	9	Dynamin_N	6	PH	4
GCC2_GCC3	7	TPR_11	9	GON	6	TSP_1	4
Ion_trans	7	MAM	9	PLAT	6	Pkinase	4

Deuterostome ancestors	domain pair count	Chordate ancestors	domain pair count	Vertebrate ancestors	domain pair count	any of six vertebrates	domain pair count
EGF	22	TSP_1	15	PDZ	10	I-set	17
SRCR	16	EGF_CA	15	EGF_CA	8	Ig_2	17
CUB	14	LRR_8	13	Pkinase	8	Ank_2	16
F5_F8_type_C	12	VWA	13	fn3	7	zf-C3HC4_2	16
Sushi	12	fn3	12	I-set	7	SH3_1	15
Lectin_C	11	Lectin_C	9	Homeobox	7	Ank_5	15
fn3	11	SH3_1	9	fn2	7	KRAB	15
TSP_1	11	EGF	8	CUB	6	Pkinase	14
Ank_2	11	hEGF	8	PH	6	WD40	14
Kringle	9	Pkinase_Tyr	8	RRM_1	6	zf-C3HC4	14
Ldl_recept_a	9	I-set	7	zf-C4	6	SH3_2	13
VWD	9	SH3_2	7	C1-set	6	TPR_1	13
hEGF	9	Death	7	SAM_2	5	zf-H2C2_2	13
Ig_2	8	WD40	7	CARD	5	RRM_1	12
EGF_CA	7	LRR_1	7	Kunitz_BPTI	5	V-set	12
SEA	7	Laminin_EGF	7	Xlink	5	hEGF	12
TIL	7	PDZ	7	VWA	4	TPR_2	12
VWC	6	Ank_2	6	EGF	4	zf-C2H2	12
LRR_8	5	Kringle	6	SH3_2	4	fn3	11
I-set	5	Pkinase	6	WD40	4	EGF	10
Fz	5	Ank	6	Ig_2	4	zf-RING_2	10
Pkinase_Tyr	5	cEGF	6	IQ	4	LRR_4	10
PAN_1	5	zf-RING_2	6	7tm_1	4	TPR_11	9
SAM_1	5	Collagen	6	PHD	4	Trypsin	9
Thyroglobulin_1	5	GPS	6	Thyroglobulin_1	4	Pkinase_Tyr	9
Trypsin	5	RRM_6	6	V-set	4	Ank	9
Kazal_2	5	CUB	5	Gla	4	LRR_6	9
Ank_4	5	F5_F8_type_C	5	SH3_1	3	C2-set	9
PHD	5	Sushi	5	F5_F8_type_C	3	PH	8
zf-CCHC	5	Ldl_recept_a	5	Sushi	3	PRY	8
TIG	5	Ig_2	5	TPR_11	3	RRM_5	8
VWA	4	zf-C3HC4_2	5	MAM	3	ig	8
Pkinase	4	PH	5	TPR_1	3	TPR_16	8
Ank	4	WSC	5	ZU5	3	TPR_9	8
cEGF	4	EF_hand_5	5	Trypsin	3	TPR_8	8
zf-C3HC4_2	4	IQ	5	HMG_box	3	Helicase_C	8
Ldl_recept_b	4	zf-B_box	5	SH2	3	HEAT_EZ	8
TIR	4	TPR_11	5	EF_hand_6	3	SCAN	8
NACHT	4	TPR_2	5	Cadherin	3	DUF2435	8
SH3_2	4	SAM_2	5	Ion_trans	3	PDZ	7
zf-RING_2	4	zf-C3HC4	5	C2-set_2	3	PHD	7
PH	4	C1_1	5	HR1	3	LRR_1	7
Ank_5	4	zf-RanBP	5	TrkA_N	3	Ank_4	7
zf-CCCH	4	TIL	4	Alpha_kinase	3	FHA	7
TILa	4	VWC	4	fn1	3	TPR_6	7
HMG_box	4	Kazal_2	4	RhoGEF	3	C1-set	6
Death	3	Laminin_G_3	4	TSP_1	2	7tm_1	6
WSC	3	CARD	4	Pkinase_Tyr	2	EF_hand_6	6
Laminin_G_3	3	zf-H2C2_2	4	Ank_2	2	zf-CCCH	6
MAM	3	BTB	4	Kringle	2	Collagen	6
GCC2_GCC3	3	SRCR	3	RRM_6	2	zf-B_box	6
Gal_Lectin	3	SAM_1	3	zf-C3HC4_2	2	BTB	6
Mucin2_WxxW	3	MAM	3	zf-RanBP	2	SAM_1	6
7tm_1	3	Mucin2_WxxW	3	Kazal_2	2	zf-met	6
Ras	3	7tm_1	3	zf-CCCH	2	EGF_2	6
Fibrinogen_C	3	Fibrinogen_C	3	Fibrinogen_C	2	THAP	6

Supplementary Table 23. Pearson correlation tests of the usage pattern of promiscuous domains

Lineage	*S. purpuratus*	*B. belcheri*	*B. floridae*	*Amphioxus ancestor*	*Deuterostome ancestor*	*Chordate ancestor*	*Vertebrate ancestor*	*Any vertebrates*
④*S. purpuratus*	--	0.39	0.39	0.27	0.46	0.21	0.14	0.19
②*B. belcheri*	0.39	--	0.71	0.68	0.43	0.41	0.07	0.03
①*B. floridae*	0.39	0.71	--	0.48	0.33	0.42	0.15	0.09
③Amphioxus ancestor	0.27	0.68	0.48	--	0.57	0.42	0.06	-0.15
⑤Deuterostome ancestor	0.46	0.43	0.36	0.57	--	0.49	0.16	0.12
⑥Chordate ancestor	0.21	0.41	0.42	0.42	0.49	--	0.29	0.26
⑦Vertebrate ancestor	0.14	0.07	0.15	0.06	0.16	0.29	--	0.24
⑧Any vertebrates	0.19	0.03	0.09	-0.15	0.12	0.26	0.24	--

Note: Pearson correlation coefficient is used here.

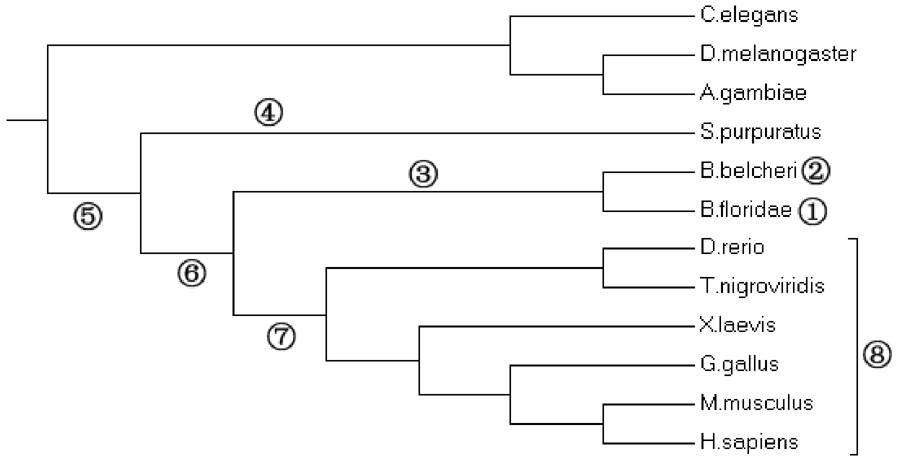

Supplementary Table 24. The rate of coding exon (or CDS) rearrangements for different species pairs

	minimum alignment length[1]	Number of ORF pairs	Number of ORF rearrangements	Relative DCJ distance based on ORF pairs	Number of CDS pairs	Number of CDS rearrangements	Number of rearrangements involving only CDS[2]	Relative DCJ distance involving only CDS
between two haplotypes of *B. belcheri*	>100bp	26,431	1,070	0.040	159,299	5,130	4,060	0.025
	>150bp	26,431	1,070	0.039	90,798	3,214	2,144	0.024
	>200bp	26,271	1,032	0.039	46,423	2,053	1,021	0.022
B. belcheri vs *B. floridae*	>100bp	7,155	1,746	0.238	55,392	7,247	5,501	0.099
	>150bp	7,149	1,742	0.237	28,686	4,747	3,005	0.105
	>200bp	6,769	1,676	0.241	12,761	2,923	1,247	0.098
C. intestinalis vs *C. savigyni*	>100bp	1,843	866	0.460	12,706	1,627	761	0.060
	>150bp	1,843	866	0.460	5,447	1,182	316	0.058
	>200bp	1,837	860	0.458	1,602	650	0	0.000
C. elegans vs *C. briggsae*	>100bp	8,612	1,989	0.231	33,559	3,195	1,206	0.036
	>150bp	8,597	1,986	0.231	20,927	2,498	512	0.024
	>200bp	8,509	1,973	0.232	13,711	2,029	56	0.004
D. melanogaster vs *D.mojavensis*	>100bp	5,035	1,264	0.251	20,555	1,452	188	0.009
	>150bp	5,022	1,261	0.251	13,468	1,317	56	0.004
	>200bp	4,998	1,257	0.251	10,064	1,240	0	0.000
G. aculeatus vs *T. Nigroviridis (fish)*	>100bp	8,892	723	0.081	56,613	1,773	1,050	0.019
	>150bp	8,891	723	0.081	26,370	1,073	350	0.013
	>200bp	8,877	719	0.081	10,396	666	0	0.000
human vs chicken	>100bp	6,672	1,515	0.155	45,909	2,146	631	0.014
	>150bp	6,668	1,510	0.154	20,756	1,311	0	0.000
	>200bp	6,653	1,504	0.154	8,034	829	0	0.000
human vs rhesus	>100bp	15,620	804	0.051	110,103	1,704	900	0.008
	>150bp	15,609	800	0.051	56,987	1,009	209	0.004
	>200bp	15,559	784	0.050	26,578	646	0	0.000
mouse vs rat	>100bp	16,973	1,132	0.067	112,080	2,138	1,006	0.009
	>150bp	16,962	1,124	0.066	58,153	1,593	469	0.008
	>200bp	16,909	1,107	0.065	27,253	1,168	61	0.002

[1]The minimum alignment length used to filter the raw blast results.

[2]The number of rearrangements involving only CDS is ONLY BUT an approximate estimation, which equals to the number of CDS rearrangements minus the number of ORF rearrangements.

Supplementary Table 25. Most common domains biasedly encoded in 1-1 phased internal exons in *B. belcheri*

This table lists the most common domains whose coding exons are significantly biased to 1-1 phase in *B. belcheri*. The corresponding exon numbers in 0-0 phase and in human genome are provided for comparison.
*Asterisks mark the top 10 most common symmetrical domains in human [3].
**These numbers are restricted to exons of at least 200bp.

pfamID	Name	Desc	*B. belcheri*		human	
			phase0-0	phase1-1	phase0-0	phase1-1
CL0001*	EGF	EGF superfamily	22	2696	17	636
PF00084*	Sushi	Sushi domain (SCR repeat)	1	1491	1	251
PF07679	I-set	Immunoglobulin I-set domain	19	358	3	345
PF13895	Ig_2	Immunoglobulin domain	0	156	0	177
PF07686	V-set	Immunoglobulin V-set domain	0	86	0	156
PF08205	C2-set_2	CD80-like C2-set immunoglobulin domain	0	30	0	38
PF13927	Ig_3	Immunoglobulin domain	0	17	0	51
PF00047	ig	Immunoglobulin domain	0	8	0	26
PF00057*	Ldl_recept_a	Low-density lipoprotein receptor domain class A	0	647	0	173
PF00041	fn3	Fibronectin type III domain	17	551	3	322
PF00059	Lectin_C	Lectin C-type domain	8	502	6	29
PF00090	TSP_1	Thrombospondin type 1 domain	9	363	12	66
PF00530	SRCR	Scavenger receptor cysteine-rich domain	1	246	3	64
PF00431*	CUB	CUB domain	7	223	0	57
PF00051*	Kringle	Kringle domain	1	223	0	4
PF00629*	MAM	MAM domain	22	210	1	26
PF00531	Death	Death domain	14	151	0	7
PF00619	CARD	Caspase recruitment domain	4	17	1	14
PF01335	DED	Death effector domain	0	11	0	1
PF01822	WSC	WSC domain	0	131	0	0
PF08016	PKD_channel	Polycystin cation channel	19	129	6	0
PF07699	GCC2_GCC3	GCC2 and GCC3	0	119	1	19
PF00147	Fibrinogen_C	Fibrinogen beta and gamma chains, C-terminal globular domain	9	105	5	17
PF12248	Methyltransf_FA	Farnesoic acid 0-methyl transferase	0	98	0	0
PF00754*	F5_F8_type_C	F5/8 type C domain	8**	87**	8	2
PF02932	Neur_chan_memb	Neurotransmitter-gated ion-channel transmembrane region	18	75	3	21
PF00024*	PAN_1	PAN domain	9	72	0	0
PF13330	Mucin2_WxxW	Mucin-2 protein WxxW repeating region	11	65	0	7
PF02140	Gal_Lectin	Galactose binding lectin domain	0	60	0	3
PF00086	Thyroglobulin_1	Thyroglobulin type-1 repeat	0	57	6	14
PF02494	HYR	HYR domain	0	56	0	1
PF00092*	VWA	von Willebrand factor type A domain	15	54	1	69
PF02822	Antistasin	Antistasin family	0	47	0	1
PF00094	VWD	von Willebrand factor type D domain	15	44	6	7
PF07653	SH3_2	Variant SH3 domain	5	44	7	3
PF03137	OATP	Organic Anion Transporter Polypeptide (OATP) family	3	43	2	33
PF07690	MFS_1	Major Facilitator Superfamily	3	43	5	12
PF01826	TIL	Trypsin Inhibitor like cysteine rich domain	1	42	1	11
PF01549	ShK	ShK domain-like	0	41	0	1
PF00151	Lipase	Lipase	5	39	10	23
PF00534	Glycos_transf_1	Glycosyl transferases group 1	2	38	0	0

PF00093*	VWC	von Willebrand factor type C domain	4	35	4	16
PF00630	Filamin	Filamin/ABP280 repeat	4	33	2	17
PF03098	An_peroxidase	Animal haem peroxidase	3	33	7	6
PF00095	WAP	WAP-type (Whey Acidic Protein) 'four-disulfide core'	0	31	0	14
PF03445	DUF294	Putative nucleotidyltransferase DUF294	2	30	0	0
PF00413	Peptidase_M10	Matrixin	1	28	0	34
PF01344	Kelch_1	Kelch motif	5	28	6	25
PF00040	fn2	Fibronectin type II domain	0	28	0	20
PF00105	zf-C4	Zinc finger, C4 type (two domains)	1	23	0	18
PF01833*	TIG	IPT/TIG domain	3	22	2	7

Supplementary Table 26. Most common domains biasedly encoded in 0-0 phased internal exons in *B. belcheri*

This table lists the most common domains whose coding exons are significantly biased to 0-0 phase in *B. belcheri*. The corresponding exon numbers in 1-1 phase and in human genome are provided for comparison.

*Domains marked with an asterisk are top 10 most common symmetrical (0-0 phase) domains in human.

**Pkinases are not extremely biased to 0-0 phased exons and are large domains usually encoded by >1 exons. They are listed here for viewing because of their abundance.

pfamID	Name	Desc	*B. belcheri*		human	
			phase0-0	phase1-1	phase0-0	phase1-1
CL0465*	Ank	Ankyrin repeat superfamily	783	78	231	45
CL0023*	AAA	AAA-ATPase superfamily	164	15	115	14
PF00069*	Pkinase**	Protein kinase domain	129	37	190	56
PF00400*	WD40	WD domain, G-beta repeat	116	33	110	28
PF07714*	Pkinase_Tyr**	Protein tyrosine kinase	111	62	78	73
PF03028	Dynein_heavy	Dynein heavy chain and region D6 of dynein motor	103	2	63	4
PF00063*	Myosin_head	Myosin head (motor domain)	95	45	126	25
PF01825	GPS	Latrophilin/CL-1-like GPS domain	73	0	12	0
PF01576	Myosin_tail_1	Myosin tail	60	0	41	0
PF00102*	Y_phosphatase	Protein-tyrosine phosphatase	57	1	71	0
PF00651	BTB	BTB/POZ domain	56	5	6	5
PF00520*	Ion_trans	Ion transport protein	46	10	105	20
PF08393	DHC_N2	Dynein heavy chain, N-terminal region 2	45	2	28	0
PF00071*	Ras	Ras family	43	10	42	6
PF00435*	Spectrin	Spectrin repeat	43	0	63	2

Supplementary Table 27. Statistics of genomic translocations in lancelet genomes

Note 1: the statistics is based on the chainNet reciprocally-best whole-genome alignments.

Note 2: for primates, only autochromosomes were used for alignments.

	B. belcheri* versus *B. floridae	**Between two haplotypes of *B. belcheri***	**Human versus rhesus**	**Human versus chimpanzee**
Genome size	426Mb	426Mb	2881Mb	2881Mb
Aligned length %	-	~70%	~85%	~90%
Total translocations	7,034	6,244	5,179	1,779
translocations of 100-50000bp	6,782	5,713	4,981	1,659
Rates of translocation of 100-50000bp (events per Mbp alignments)	-	19.2	2.0	0.6
total translocations length	16.9Mb	6.7Mb	10.8Mb	4.5Mb
translocations containing coding exons	3,097 ***	1,056 ***	310	158
translocations containing domain exons	1,047 ***	293 ***	172	76

***These numbers are significantly higher than their corresponding numbers in primates ($p<1e-16$, chi-square test).

Supplementary Table 28. Phase bias of coding exon contained in translocations

A. Between *B. belcheri* and *B. floridae*

	Total exon count	Middle exon count	Middle exons containing no domains		Middle exons containing domains	
			0-0 phase	1-1 phase	0-0 phase	1-1 phase
All translocations	9496	4807	879 (18.3%)	837 (17.4%)	353 (14.2%)	789 (31.7%)
Translocations containing ≤10 exons	4298	1889	319 (16.9%)	362 (19.2%)	131 (13.0%)	352 (34.9%)
Translocations containing ≤3 exons	1735	633	116 (18.3%)	136 (21.5%)	39 (11.2%)	156 (45.0%)
Translocations containing single exon	704	255	46 (18.0%)	55 (21.6%)	16 (12.0%)	68 (51.1%)

B. Within *B. belcheri* (between two haploid genome sequences)

	Total exon count	Middle exon count	Middle exons containing no domains		Middle exons containing domains	
			0-0 phase	1-1 phase	0-0 phase	1-1 phase
All translocations	1459	608	102 (16.8%)	89 (14.6%)	28 (9.4%)	123 (41.4%)
Translocations containing ≤10 exons	895	315	49 (15.65)	55 (17.5%)	14 (10.1%)	60 (43.5%)
Translocations containing ≤3 exons	543	157	25 (15.9%)	26 (16.6%)	7 (9.2%)	39 (51.3%)

C. Between human and rhesus

	Total exon count	Middle exon count	Middle exons containing no domains		Middle exons containing domains	
			0-0 phase	1-1 phase	0-0 phase	1-1 phase
All translocations	502	192	41 (21.4%)	28 (14.6%)	33 (20.8%)	20 (12.6%)
Translocations containing ≤10 exons	359	120	28 (23.3%)	14 (11.7%)	26 (23.9%)	16 (14.7%)
Translocations containing ≤3 exons	187	50	8 (16.0%)	4 (8.0%)	11 (23.9%)	4 (8.7%)

D. Between human and chimpanzee

	Total exon count	Middle exon count	Middle exons containing no domains		Middle exons containing domains	
			0-0 phase	1-1 phase	0-0 phase	1-1 phase
All translocations	249	96	17 (17.7%)	12 (12.5%)	16 (18.6%)	11 (12.8%)
Translocations containing ≤10 exons	211	78	13 (16.7%)	10 (12.8%)	12 (17.1%)	10 (14.3%)
Translocations containing ≤3 exons	71	30	7 (23.3%)	4 (13.3%)	5 (31.3%)	2 (12.5%)

Supplementary Table 29. Common domain types encoded in shuffled exons

*Note that only the translocations that contain ≤3 coding exons were counted in this analysis.

B. belcheri vs *B. floridae*		Within *B. belcheri*		Human vs rhesus		Human vs chimpanzee	
Name	count	Name	count	Name	count	Name	count
LRR_8	25	Lectin_C	7	V-set	10	SSDP	2
Sushi	24	EGF	7	7tm_4	6	UCH	2
7tm_1	21	fn3	6	p450	6	CTP_transf_2	2
Lectin_C	18	F5_F8_type_C	5	UDPGT	3	Ribosomal_L19e	2
Fibrinogen_C	14	Sushi	4	HAP1_N	2	NPIP	2
EGF	14	CUB	4	Ribosomal_S5	2	Calponin	2
Death	11	zf-C2H2	3	KRAB	2	PDZ	1
EGF_CA	11	EGF_3	3	RCC1	2	Pkinase	1
UDPGT	10	Death	3	MIF	2	Trypsin	1
NHL	10	Fibrinogen_C	2	zf-H2C2_2	2	IL8	1
Methyltransf_FA	9	TSP_1	2	Perilipin	2	RNase_T	1
F5_F8_type_C	9	Lipase_GDSL_2	2	Ras	2	DUF1220	1
Exo_endo_phos	9	CO_deh_flav_C	2	Carb_anhydrase	2	Inositol_P	1
Gal-3-0_sulfotr	8	SCAN	2	Cys_knot	2	7tm_4	1
p450	7	Glycos_transf_1	2	WD40	2	p450	1
Glyco_transf_29	6	Pkinase	2	DUF2359	1	BEN	1
Glycos_transf_1	6	GIY-YIG	2	HnRNPA1	1	DnaJ	1
VWA	6	zf-C3HC4	2	Ferritin	1	Amino_oxidase	1
DED	6	Dam	2	G-gamma	1	Defensin_propep	1
SRCR	5	7tm_1	2	SNF	1	Ribosomal_S2	1
WSC	5	DED	2	MHC_I	1		
Collagen	4	CLCA_N	2	Fork_head	1		
Ldl_recept_a	4	LRAT	2	Fructosamin_kin	1		
Pkinase	4	DUF3504	2	Laminin_G_2	1		
Pkinase_Tyr	4	SAP	2	Homeobox	1		
Ig_2	4	p450	2	NUDIX	1		
EGF_3	4	EGF_CA	2	zf-Tim10_DDP	1		
EcKinase	4	LRR_6	1	SCAN	1		
Nucleic_acid_bd	4	Ank	1	P16-Arc	1		
T4_deiodinase	3	Phage_integrase	1	Profilin	1		
Ank	3	EamA	1	V1R	1		
TCTP	3	DEAD	1	SBP_bac_3	1		
zf-C3HC4_2	3	MANEC	1	FKBP_C	1		
TSP_1	3	CBFD_NFYB_HMF	1	RRM_1	1		
Kringle	3	Glyco_transf_29	1	Pkinase_Tyr	1		
PC-Esterase	3	PAN_1	1	5_nucleotid	1		
Lipase_GDSL_2	3	BTB	1	IL8	1		
K_tetra	3	PARP	1	RA	1		
Trypsin	3	Tmem26	1	VPS9	1		
fn3	3	CENP-B_N	1	AA_permease_2	1		
TIL	3	NHL	1	Atg8	1		
Cupin_8	3	K_tetra	1	Serpin	1		
Patched	3	TPR_12	1	ATP-synt_G	1		
DUF2045	3	DUF1891	1	TAF4	1		
Alpha_kinase	3	TIR_2	1	HAD_2	1		
Neurexophilin	3	Cu-oxidase_3	1	Ribosomal_S5_C	1		
FMO-like	3	Pkinase_Tyr	1	GTP_EFTU	1		
Rad52_Rad22	3	AIG1	1	Cystatin	1		
adh_short_C2	3	Neur_chan_LBD	1	adh_short	1		
Xlink	3	PLAC8	1	DUF1220	1		

Supplementary Table 30. Association of retrotranscriptases/transponsaes and micro-translocations

	RVT number in reference genome	RVT number in Genome per Mbps	RVT number associated with translocations (relative%)	RVT number associated with translocation per Mbps
Within *B. belcheri*	2300	5.37	365 (16%) ***	21.3 ***
Human VS Rhesus	20883	7.25	80 (0.38%)	4.49
Human VS Chimpanzee	20883	7.25	36 (0.17%)	6.52

	TNP number in reference genome	TNP number in Genome per Mbps	TNP number associated with translocations (relative%)	TNP number associated with translocation per Mbps
Within *B. belcheri*	415	0.97	51 (12%) ***	3.15 ***
Human VS Rhesus	2926	1.02	22 (0.75%)	1.23
Human VS Chimpanzee	2926	1.02	10 (0.34%)	1.81

Note 1: RVT=reverse transcriptase or retrotranscriptase; TNP=transponsase; Mbps=million base pairs.

*** These numbers are significantly higher than their corresponding numbers in primates (p<1e-16, chi-square test).

Supplementary Table 31. Total length of CNE candidates (conserved alignments) in five species pairs.

A. Stringent criteria*.

Parameter set 1*<br>Unit: base pairs	B. belcheri (versus B. floridae)	C. elegans (versus C. briggsae)	D. melanogaster (versus D. mojavensis)	human (versus mouse)	human (versus opossum)
genome size	426108443	100286070	168736537	3101788170	3101788170
all repeats	111126564	20611575	48882888	1446623535	1446623535
all CDS**	47983502	26621146	22858926	53048880	53048880
aligned repeats	707790	131128	45063	4590310	677695
aligned CDS	30757489	11236528	10796231	29858023	20555816
aligned CNE	45440901	3027725	6670794	106174711	33471985
N gaps	5461660	0	0	234350393	234350395
aligned repeats %	0.17	0.13	0.03	0.15	0.02
aligned CDS %	7.22	11.20	6.40	0.96	0.66
aligned CNE %	10.66	3.02	3.95	3.42	1.08
aligned CNE+CDS%	17.88	14.22	10.35	4.39	1.74

*Special parameter settings for Lastz and chainNet: --masking=0, --hspthresh=3000, --ydrop=9400, --gappedthresh=3000, --gap=400, 30, --step=1, --seed=12of19, --identity=80, and the score matrix "100 -300 -300 -300; -300 100 -300 -300; -300 -300 100 -300; -300 -300 -300 100".

**For the human genome, CDS includes the exons of protein pseudogenes, while for other genomes, pseudogene CDS is not considered.

B. Relaxed criteria*.

Parameter set 2*<br>Unit: base pairs	B. belcheri (versus B. floridae)	C. elegans (versus C. briggsae)	D. melanogaster (versus D.mojavensis)	human (versus mouse)	human (versus opossum)
genome size	426108443	100286070	168736537	3101788170	3101788170
all repeats	111126564	20611575	48882888	1446623535	1446623535
all CDS**	47983502	26621146	22858926	53048880	53048880
aligned repeats	1295778	298587	79445	12200135	1056521
aligned CDS	32114219	14133610	13259266	31162985	22812367
aligned CNE	63190734	5188359	10526736	178900727	48516514
N gaps	5460973	0	0	234350393	234350395
aligned repeats %	0.30	0.30	0.05	0.39	0.03
aligned CDS %	7.54	14.09	7.86	1.00	0.74
aligned CNE %	14.83	5.17	6.24	5.77	1.56
aligned CNE+CDS%	22.37	19.27	14.10	6.77	2.30

*Special parameter settings for Lastz and chainNet: --masking=0, --hspthresh=3000, --ydrop=9400, --gappedthresh=3000, --gap=400, 30, --step=1, --seed=12of19, --identity=80, and the score matrix "100 -200 -200 -200; -200 100 -200 -200; -200 -200 100 -200; -200 -200 -200 100".

**For the human genome, CDS includes the exons of protein pseudogenes, while for other genomes, pseudogene CDS is not considered.

Supplementary Table 32. Total length of refined CNE candidates in five species pairs.

	B. belcheri (versus B. floridae)	C. elegans (versus C. briggsae)	D. melanogaster (versus D.mojavensis)	human (versus mouse)	human (versus opossum)
genome size	426108443	100286070	168736537	3101788170	3101788170
coarse CNE length	45440901	3027725	6670794	106174711#	33471985##
<75bp	6782290	1375417	3432906	12433719	4006304
adjacent to CDS	6179707	248689	83839	6956979	1675110
Blast hit to protein, tRNA, rRNA, etc	2337567**	12073	28716	755567	247049
refined CNE length*	30003722	1353843	2839649	85319227	27436584
refined CNE length %	7.04	1.35	1.68	2.75	0.88
refined CNE count	135046	9763	25211	369079	124195
average length	222.2	138.7	112.6	231.2	220.9

* CNE candidates that are <70% identity, <75bp, adjacent to CDS or homologous to known proteins/tRNA/rRNA/snoRNA/scRNA/snlRNA were removed.

** Protein hits accounted for 2,272,249bp.

If all protein gene exons are removed, this value will be reduced to 96465841bp (~9.7Mb smaller).

If all protein gene exons are removed, this value will be reduced to 29744189bp (~3.7Mb smaller).

Supplementary Table 33. Lancelet microRNA genes confirmed in *B. belcheri* genome assemblies.

For the annotation and precursor sequences of these microRNA genes, the audience is referred to Chen et al's work[4].

microRNA ID	*notes*	*microRNA ID*	*notes*
bbe-mir-7/bbe-mir-7_star	not in Bbv18ref	bbe-mir-92a-1/bbe-mir-92a-1_star	
bbe-mir-s26-2	not in Bbv18ref	bbe-mir-92a-2/bbe-mir-92a-2_star	
bbe-mir-s31	not in Bbv18ref	bbe-mir-92b/bbe-mir-92b_star	
bbe-mir-133/bbe-mir-133_star	not in bfv1	bbe-mir-92c	
bbe-mir-29a	not in bfv1	bbe-mir-96/bbe-mir-96_star	
bbe-mir-29b	not in bfv1	bbe-mir-99a/bbe-mir-99a_star	
bbe-mir-375	missed	bbe-mir-s10	
bbe-mir-s26-1	missed	bbe-mir-s11	
bbe-mir-s5/bbe-mir-s5_star	missed	bbe-mir-s1-1	
bbe-mir-s40	missed	bbe-mir-s1-2	
bbe-mir-100/bbe-mir-100_star	filtered as repeats/CDS	bbe-mir-s12/bbe-mir-s12_star	
bbe-mir-184	filtered as repeats/CDS	bbe-mir-s13	
bbe-mir-278	filtered as repeats/CDS	bbe-mir-s14/bbe-mir-s14_star	
bbe-mir-92a-3		bbe-mir-s15/bbe-mir-s15_star	
bbe-mir-s46		bbe-mir-s16	
bbe-mir-s47/bbe-mir-s47_star		bbe-mir-s17	
bbe-mir-s7		bbe-mir-s18	
bbe-let-7a-1		bbe-mir-s19	
bbe-let-7a-2/bbe-let-7a-2_star		bbe-mir-s2	
bbe-mir-1/bbe-mir-1_star		bbe-mir-s20	
bbe-mir-10a/bbe-mir-10a_star		bbe-mir-s21	
bbe-mir-10b		bbe-mir-s22/bbe-mir-s22_star	
bbe-mir-124		bbe-mir-s23/bbe-mir-s23_star	
bbe-mir-125/bbe-mir-125_star		bbe-mir-s24	
bbe-mir-129/bbe-mir-129_star		bbe-mir-s25	
bbe-mir-135a-1/bbe-mir-135a-1_star		bbe-mir-s27/bbe-mir-s27_star	
bbe-mir-135a-2/bbe-mir-135a-2_star		bbe-mir-s28	
bbe-mir-135b-1		bbe-mir-s29	
bbe-mir-135b-2		bbe-mir-s3	
bbe-mir-137		bbe-mir-s30	
bbe-mir-183		bbe-mir-s32/bbe-mir-s32_star	
bbe-mir-190/bbe-mir-190_star		bbe-mir-s33	
bbe-mir-200a/bbe-mir-200a_star		bbe-mir-s34	
bbe-mir-200b/bbe-mir-200b_star		bbe-mir-s35	
bbe-mir-210/bbe-mir-210_star		bbe-mir-s36	
bbe-mir-216/bbe-mir-216_star		bbe-mir-s37-1	
bbe-mir-217/bbe-mir-217_star		bbe-mir-s37-2	
bbe-mir-219/bbe-mir-219_star		bbe-mir-s37-3	

microRNA ID	*notes*	*microRNA ID*	*notes*
bbe-mir-22		bbe-mir-s38/bbe-mir-s38_star	
bbe-mir-25/bbe-mir-25_star		bbe-mir-s39-1	
bbe-mir-252a/bbe-mir-252a_star		bbe-mir-s39-2	
bbe-mir-252b		bbe-mir-s4/bbe-mir-s4_star	
bbe-mir-281/bbe-mir-281_star		bbe-mir-s41/bbe-mir-s41_star	
bbe-mir-31		bbe-mir-s42	
bbe-mir-33-1/bbe-mir-33-1_star		bbe-mir-s43/bbe-mir-s43_star	
bbe-mir-33-2/bbe-mir-33-2_star		bbe-mir-s44/bbe-mir-s44_star	
bbe-mir-34a-1		bbe-mir-s45	
bbe-mir-34a-2		bbe-mir-s48/bbe-mir-s48_star	
bbe-mir-34b-1		bbe-mir-s49	
bbe-mir-34b-2		bbe-mir-s50	
bbe-mir-34c		bbe-mir-s51/bbe-mir-s51_star	
bbe-mir-449b-1/bbe-mir-449b-1_star		bbe-mir-s52	
bbe-mir-449b-2/bbe-mir-449b-2_star		bbe-mir-s53/bbe-mir-s53_star	
bbe-mir-71		bbe-mir-s6	
bbe-mir-9-1/bbe-mir-9-1_star		bbe-mir-s8/bbe-mir-s8_star	
bbe-mir-9-2/bbe-mir-9-2_star		bbe-mir-s9	
bbe-mir-141			

Supplementary Table 34. The thirty CNE-enriched genomic regions in Chinese lancelets

ID	Scaffold	start	end	length	Gene count	CNE count	Gene range	Comment
A01	6	4473136	5376164	903028	34	674	240210R..240540R	
A02	53	2221	547290	545069	24	531	223830F..224060F	
A03	18	1654438	2553172	898734	39	730	084540R..084920R	
A04	35	518874	1554773	1035899	41	889	171120F..171520F	Hox genes
A05	10	1701853	2817089	1115236	47	961	010390R..010850R	
A06	5	1564577	2257742	693165	39	817	212620F..213000F	
A07	2	3501968	4351942	849974	37	816	097340R..097700F	
A08	23	2614485	3289044	674559	38	789	118610R..118980R	
A09	48	266484	1876382	1609898	57	1088	208580F..209140R	
A10	43	793387	1653080	859693	38	761	197800R..198170F	
A11	4	711567	1686165	974598	43	763	185050R..185470R	
A12	54	282708	1050185	767477	39	793	226010F..226390F	
A13	19	1834508	2716414	881906	44	883	090240F..090670F	
A14	33	1309494	2103787	794293	28	493	165470F..165740F	
B01	30	1266448	1914467	648019	32	490	155170F..155480R	
B02	71	873449	1731202	857753	22	340	264460R..264670F	
B03	3	2982943	3464154	481211	28	408	149410R..149680F	
B04	3	7439258	8152958	713700	31	425	153240F..153540F	
B05	34	146490	783984	637494	36	587	167530R..167880F	
B06	2	4808437	5241867	433430	23	358	098110F..098330R	
B07	74	1138049	1676588	538539	29	456	268310F..268590F	
B08	99	572765	1330130	757365	36	608	303500F..303850F	
B09	23	2152183	2592668	440485	24	383	118350R..118580F	
B10	51	1607649	2208027	600378	32	486	220810R..221120F	LBP,BPI,TLR,histanmine receptor
B11	8	1576105	2107432	531327	25	417	275980F..276220F	
B12	17	3306985	4142176	835191	37	660	078590R..078950F	
B13	4	2868189	3318812	450623	28	427	186240F..186510F	
B14	7	4307379	4949490	642111	37	572	260970R..261330R	
B15	47	98929	646640	547711	32	516	206420R..206730F	
B16	41	302731	1196268	893537	40	576	193270F..193660R	

Supplementary Table 35. The GO analysis of the CNEs conserved between lancelets and humans

Cross-phyla conserved CNEs show enrichment in the vicinity (±10kb) of protein genes of certain GO functions. Numbers show the enrichment change level. Highlighted numbers are significant at *P*<0.01 (chi-square tests).

	Human		lancelet	
go_terms	>45bp change%	>30bp change%	>45bp change%	>30bp change%
reproduction	-19.7	-13.9	3.2	6.5
metabolic process	-4.4	0.6	-11.2	-8.3
immune system process	-20.9	-12.5	-8.2	8.7
growth	78.4	62.3	9.7	10.0
reproductive process	-11.2	-4.8	-1.2	1.8
biological adhesion	126.0	73.7	43.6	28.6
signaling	22.7	37.6	19.8	16.5
multicellular organismal process	29.0	33.0	17.6	18.6
developmental process	43.8	42.3	23.0	24.5
locomotion	71.6	66.5	26.6	21.4
positive regulation of biological process	42.7	40.8	36.1	22.3
negative regulation of biological process	23.8	30.7	30.8	23.0
regulation of biological process	11.7	16.9	13.7	8.1
response to stimulus	3.7	13.8	9.6	7.3
localization	16.1	16.3	16.3	12.8
establishment of localization	2.6	8.2	12.6	7.2
biological regulation	9.3	15.5	13.1	7.7
cellular component organization or biogenesis	21.7	20.7	20.7	23.9
membrane	5.4	3.2	-2.4	-2.1
extracellular region	-6.1	-11.6	-9.4	-7.0
cell	0.3	2.6	6.7	4.5
cell junction	75.7	59.3	51.1	47.2
extracellular matrix	6.3	30.2	35.5	29.5
membrane-enclosed lumen	4.4	9.8	11.7	2.8
macromolecular complex	3.3	6.3	14.4	14.8
organelle	1.0	4.6	8.8	7.0
extracellular region part	-16.7	-12.3	14.4	11.4
organelle part	-3.5	3.1	6.1	6.4
membrane part	2.6	0.6	-12.8	-8.4
synapse part	230.9	168.4	8.3	16.0
cell part	0.3	2.6	6.7	4.6
synapse	170.2	142.9	28.8	26.4
protein binding transcription factor activity	56.2	66.8	27.6	25.3
nucleic acid binding transcription factor	127.9	100.9	96.2	81.3
catalytic activity	-9.0	-4.4	-10.1	-8.7
receptor activity	5.0	6.5	-34.8	-23.4
structural molecule activity	-33.0	-34.1	18.7	20.1
transporter activity	-6.2	-3.3	-15.0	-11.4
binding	6.2	9.3	7.8	5.2
enzyme regulator activity	-21.8	19.1	35.2	41.4
molecular transducer activity	11.3	11.8	-14.9	-11.2

Supplementary Note 1 Background of the Chinese amphioxus and its relationships with the Japanese and Malaysian amphioxus

The lancelet, also called amphioxus, belonging to the subphylum Cephalochordata, represents the living basal lineage of the phylum Chordata (which includes three subphyla, Cephalochordata, Urochordata and Vertebrata) [5]. Lancelets are filter-feeders dwelling in the shallow sandy sea floor along coastlines. The first species of lancelet was described by Pallas in 1774, and there are now 31 known species inhabiting seashores around tropical and temperate oceans [6,7]. Lancelets are currently widely distributed along the Chinese coastline, from Qingtao to Beihai (Supplementary Figure 1A). In fact, the entire coastal area, from Northern Japan to Southeast Asia, is the natural habitat for this genus (Supplementary Figure 1A).

The habitats of three amphioxus species (*B. belcheri, B. japonicum and B. malayanum*) overlap in the coastal area between Xiamen and Beihai.

Traditionally, all lancelets in Chinese seas are collectively referred to as Chinese amphioxus and were considered to comprise a single species, *Branchiostoma belcheri*; those distributed in Qingdao waters were treated as a sub-species named *Branchiostoma belcheri tsingtauense*. These concepts have recently proved inaccurate.

Xiamen, a coastal city in China, has long been famous for its abundance of lancelets [8]. *Branchiostoma belcheri* was first reported in Xiamen waters in 1932 [9] and was once believed to include all lancelets in the region. However, it was recently discovered that there are two similar but distinct lancelet species in the region [10,11]. One species is mainly distributed from Xiamen to Beihai, in the sub-tropical oceans, whereas the second species is genetically the same as lancelets from the northern Chinese (like QingDao waters) and Japanese seas, suggesting that the second species mainly inhabits temperate oceans (Supplementary Figure 1). Lancelets from Qingdao waters and Japanese waters were traditionally considered *B. belcheri tsingtauense*, a sub-species of *B. belcheri*. Now, according to the priority rules of nomenclature, the first species is entitled to the name *Branchiostoma belcheri*, and the second species has been renamed *Branchiostoma japonicum* [10,12].

Studies of *Cyt b* gene sequences and 12S rRNA gene sequences have revealed significant divergence between *B. belcheri* and *B. japonicum* [13-15]. In addition, cytotaxonomic analyses found that the diploid chromosome numbers were 2n=40 in *B. belcheri* and 2n=36 in *B. japonicum* [16] (and, for comparison, 2n=38 in *B. floridae*). Further experiments confirmed that the two species are apparently reproductively isolated, which explains how the two species can dwell in the same habitat (Xiamen waters) but maintain independence. In addition, the two species in Xiamen show traces of morphological differences: 1) the rostral fin is slightly round with an obtuse end in *B. belcheri* but elliptical with a cuspate end in *B. japonicum*; 2) the number of preanal fin chambers is more than 80 in *B. belcheri* but normally less than 70

in *B. japonicum*, and the chambers are slender in the former but stout in the latter; 3) the caudal fin of *B. belcheri* is narrower than that of *B. japonicum*, and the angles between the dorsal and super-caudal fins and between the preanal and sub-caudal fins are obtuse in *B. belcheri* but acute in *B. japonicum* [10]. However, these morphological differences are not absolute because in reality, approximately 20% of individuals collected from the wild could not be unambiguously assigned to either species based on morphology.

A foreign species, *Branchiostoma malayanum*, which mainly dwells in Malaysian waters (Southeast Asia), is occasionally found along the southern China seashore, near Hong Kong, for example [11,17], hence suggesting the frequent incursions of this tropical species.

Although *B. belcheri* has been shown to share more morphological similarities with *B. japonicum* than *B. malayanum*, molecular comparisons using 12S RNA genes and AFLP markers indicated that *B. belcheri* is less different from *B. malayanum* than from *B. japonicum* [11].

Geographically, the Chinese amphioxus population is mainly distributed along the Southern China coastline from Xiamen to Beihai, which extends at least 1,200 kilometers (Supplementary Figure 1A). Because the habitat of Chinese amphioxus should extend to the north of Xiamen, to the west of Beihai and to Taiwan Island, we estimated that the actual habitat could extend over 2,000 kilometers. According to our surveys, Chinese amphioxus are often present at a density of hundreds of individuals per square meter in certain locations, such as the Xiamen waters (24.51°E, 118.26°N), the Maoming waters (21.41°E,111.19°N) and the Zhanjiang waters (20.95°E, 110.55°N); hence, we estimated that the actual population could consist of at least billions of individuals. Our analyses of the 1.2 kb non-coding mitochondrial DNA between *nad5* and *nad6* failed to distinguish subpopulations from Xiamen and Beihai – two habitats located approximately 1,200 kilometers apart (Supplementary Figure 1B). This suggests that the genetic structure of the natural population of the Chinese lancelet is weak or absent.

In this study, the species sequenced was *B. belcheri* from the Xiamen waters, a lancelet that shows typical characteristics of sub-tropical marine animals, such as a larger body size, faster developmental speed and a longer breeding season (relative to the temperate species *B. japonicum*) [18]. However, no comparisons of development or reproduction have been conducted between *B. belcheri* and another co-habitant, the tropical species *B. malayanum*.

B. belcheri was the first lancelet to be raised in captivity for multiple generations [18]. Methods for year-round reproduction and spawning induction have also recently been developed for *B. belcheri* [19,20]. Furthermore, a recent attempt suggests that TALENs can be used to induce mutagenesis at specific genomic loci in this species [21]. The availability of on-schedule embryonic materials and direct mutagenesis approaches will accelerate the process of establishing amphioxus as a model organism suitable for experimental biology.

Supplementary Note 2 Genome sequencing and assembly for the Chinese amphioxus *Branchiostoma belcheri*

Sample collection and DNA isolation

Specimens of outbred male adult *B. belcheri* were collected in July 2008 from Huangcuo (24°27′07″N, 118°10′27″E) in the Xiamen Rare Marine Creature Conservation Areas, China (Supplementary Figure 1). Animals were kept in filtered running seawater for 24 hours to facilitate cleaning of the body and emptying of the digestive tract. Ripe gonads full of sperm were harvested from a single large healthy male that was approximately 2-3 years old and 4 centimeters long. Genomic DNA was purified from the gonads using the DNeasy™ blood and tissue kit (QIAGEN). Quality and quantity were evaluated by Nanodrop and agarose electrophoresis. A total of 280 μg DNA was obtained from the single male, with a fragment size of 30-40 kb.

Construction and sequencing of shotgun and paired-end libraries

Two platforms of next-generation sequencing technologies, the 454 platform and the Illumina platform, were used to sequence the diploid genome of the selected male lancelet.

The 454 dataset was sequenced by GS FLX Titanium chemistry. Both shotgun and paired-end libraries were prepared using Roche’s protocols and GS FLX Titanium series kits. The total reads dataset consisted of 17 million shotgun reads and 27 million paired-end reads, which were generated from multiple shotgun libraries and multiple paired-end libraries, with insert sizes of 2 kb, 3 kb, 8 kb and 20 kb (Supplementary Table 1).

The Illumina dataset consisted of four paired-end libraries with insert sizes of 340-600 bp. All libraries were constructed according to the Illumina protocols. Each library was subjected to 2x 115 bp paired-end sequencing on the Genome Analyzer IIx (GAIIx). A total of 145 million paired-end reads were obtained, yielding approximately 33 Gb (Supplementary Table 1).

Genome size estimation

The genome size was estimated by the k-mer method as described [22,23]. In brief, genome size (G) can be determined by dividing the total amount of sequenced bases (T) by the sequencing depth (D). The sequencing depth (D) can be estimated by the formula $D=E*L/(L-K+1)$, where L is the average read length, K is the k-mer size, and E is the peak coverage depth for the given K. The peak coverage depth may decrease with longer k-mer size; therefore, an optimal combination of K and E should be inferred by analyzing the k-mer distribution profiles.

Quality-filtered 454 reads, including both shotgun and paired-end reads, were extracted with the Newbler assembler[24]. Both the filtered 454 reads and a subset of the Illumina reads were subjected to k-mer distribution profiling. The calculation revealed that the k-mer depth

peaked at 35.1, with a k-mer size of 20, a total read length of 36 Gb and a mean read length of 143 bp, which therefore gave an estimate of 884 Mb for the sequenced genome.

Given that both haplotypes of the chosen individual were sequenced together and that the average heterozygosity between two haplotypes was 4-5% (Supplementary Note 4), the haploid genome size was assumed to be half the estimates, namely, 442 Mb, as a close estimate to the actual haploid genome size.

In addition, a cytometry analysis of the sperm cells yielded an estimated haploid genome size of 440 Mb.

This haploid genome size is considerably smaller than that reported for the Florida amphioxus, *B. floridae* (500~520 Mb) [1]. This size difference was also confirmed by our later intron size comparison between the two species (Supplementary Note 9).

Haploid genome assembly version 7: initial attempt

Lancelets are marine species with high allelic polymorphisms due to their large effective breeding population. The individual sequenced here exhibited ~5% heterozygosity in its diploid genome (Supplementary Note 4) plus a large quantity of repetitive sequences (~30% of the genome).

Whole-genome shotgun (WGS) assembly for highly polymorphic diploid genomes is generally difficult and does not reach the same level of quality as assemblies for haploid genomes or diploid genomes with low levels of polymorphism[25-29]. The difficulty is caused by sequencing two closely related haplotypes together. The process is even more challenging when the next generation sequencing (NGS) technologies are used in place of Sanger methods [30] because short read length, higher error rates and new types of sequencing errors exacerbate the problem [31].

We deemed that haplotypes could be better resolved by longer reads, whereas base-level errors could be rectified by a high depth of short reads. Therefore, we generated 30x 454 reads and 70x Illumina reads (Supplementary Table 1).

SOAPdenovo assembly. SOAPdenovo2 is a de Bruijn graph-based assembler designed for short-read (next-generation) sequencing[32]. An early study showed that SOAPdenovo could not adequately assemble the polymorphic oyster diploid genome (with a polymorphism rate of 1.3%) from pure high depth (155x) of short reads, but when combined with fosmid pooling and other methods, SOAPdenovo managed to produce an oyster genome assembly with contig and scaffold N50 sizes of 19kb and 401kb, respectively[33]. Here we applied SOAPdenovo on our sequencing data. Different kmer sizes (33-95bp) and different combinations of datasets (50xIllumin reads, 70x Illumina reads and 30x 454reads+70x Illumina reads) were attempted, and the resulting assemblies spanned 600-900 Mb, with contig N50 sizes of 3-4 kb.

Newbler assembly. All 454 read data were assembled using Newbler [34] version 2.3 with pre-defined settings for large genomes. The resulting assembly spanned 599 Mb, consisting of 23,481 scaffolds with an N50 size of 144 kb, and 94,475 contigs with an N50 size of 8 kb. This assembly was excessively fragmented and reduced, i.e., its span was close to neither the diploid genome size nor the haploid genome size.

Celera assembly. The Celera Assembler with the Best Overlap Graph (CABOG) [35] can be used to assemble hybrid datasets (Sanger+454, 454+Illumina and PacBio+Illumina, etc.). We first applied this program on the 454 reads dataset. Special parameters for CABOG included: utgErrorRate=0.03; overlapper=mer; merSize=22; unitigger=bog; doExtendClearRanges=2; stoneLevel=2; doResolveSurrogates=1; cgwDemoteRBP=1; and toggle=0.

Diploid assembly version 7. The initial CABOG diploid assembly had a scaffold N50 size of 232 kb and a contig N50 size of 17 kb. GapCloser [36] version 1.04 was used to fill the N-gaps with Illumina paired-end reads. The final resulting diploid assembly spanned 708 Mb, with a contig N50 size of 73 kb and a scaffold N50 size of 232 kb (Supplementary Table 2).

Reference haploid assembly version 7. The v7 diploid assembly contained redundant alleles, remained highly fragmented and was infested with numerous middle-to-large-scale mis-assemblies. Two notorious types of error in polymorphic diploid assemblies are the tandem mis-assembly of alleles and mis-joins of unrelated genomic portions that violate the large-scale (>100 kb) colinearity between alleles [37]. To automate and seek an optimized solution for these problems, we developed HaploMerger, a pipeline containing a series of algorithms and programs designed to remove assembly errors and infer reference haploid assemblies from a given diploid assembly [31]. The original HaploMerger (i.e., release_20110720) was used to process the v7 diploid assembly with default parameters. A total of 413 tandem mis-assemblies (>10 kb), accounting for 8.6 Mb, were removed, and a total of 132 major mis-joins (>50 kb) were detected. For each mis-join, one of the two implicated scaffolds was selected to break up based on a set of heuristics that prefer to preserve sequence continuity [31]. This breaking scheme is far from perfect because it may cause HaploMerger to erroneously break the correct scaffold. In the end, HaploMerger produced a reference haploid assembly spanning 416 Mb, with a scaffold N50 size of 833 kb and a contig N50 size of 104 kb (Supplementary Table 2).

Quality inspection of the v7 assembly. The v7 assembly provided an opportunity to assess our assembly strategy. (1) For polymorphic read data, a high error rate allowance is confused with true polymorphism, inevitably causing excessive allele collapsing, assembly errors and short scaffolds when further complicated by short read length, repeats, and sequence duplications. (2) Linking the diploid assembly with 20 kb paired-end reads did not yield significant improvement. It appeared that excessive assembly errors and the presence of multiple alleles prevent effective scaffolding. (3) We observed many more small tandem mis-assemblies (<10 kb) than expected. (4) To avoid false positives, HaploMerger by default does not process potential small-scale mis-joins (<50 kb). However, the use of a high

“utgErrorRate” seemed to cause excessive (potential) mis-joins, with a large portion classified as “small scale” (<50 kb) due to the short scaffold size. (5) When two scaffolds violate large-scale colinearity (e.g., >50 kb mis-join), the default action of HaploMerger (release_20110720) is to break one of the two scaffolds without consulting the mate-pair graph or the contig-scaffold layout. This may erroneously break the correct scaffold and conceal the problematic scaffold. (6) The huge contig N50 size suggests that GapCloser version 1.04 is too aggressive in closing N-gaps.

Haploid genome assembly version 15: hierarchical scaffolding

After inspection of assembly version 7, we redesigned our assembly strategy and created a new assembly.

Step 1. **Diploid assembly.** CABOG was used to create a diploid assembly from all 454 shotgun reads and paired-end reads with insert sizes of 2 kb, 3 kb and 8 kb. Other specific parameters included: utgErrorRate=0.02; overlapper=mer; merSize=22; unitigger=bog; doExtendClearRanges=2; stoneLevel=2; doResolveSurrogates=1; and cgwDemoteRBP=1. This generated a new diploid assembly (version 15) with a scaffold N50 size of 150 kb and a contig N50 size of 16 kb (Supplementary Table 2).

Step 2. **Haploid assembly.** An expansion kit for HaploMerger was used to create a haploid assembly from the diploid assembly. The new HaploMerger module can detect and remove tandem-assembled alleles larger than 100 bp. A total of 1460 tandem mis-assemblies accounting for 7.9 Mb were removed from the diploid assembly. For large-scale (>50 kb) mis-joins, the new HaploMerger attempts to interrogate paired-end linking information to determine which scaffold should be broken up and to consult the contig-scaffold layout to decide the breakpoint position. Specifically, the scaffold receiving less link support (<1/3 of those of another scaffold) across the breakpoint within a 50 kb range is selected for breaking. If the paired-end links favor neither of the scaffolds, then both scaffolds are broken. A total of 167 mis-joins were processed.

Step 3. **Hierarchical scaffolding.** Bambus [38] version 2.33 was used to further scaffold the haploid assembly (produced in Step 2) with all 20 kb paired-end reads. Before linking, the haploid assembly was first masked by WindowMasker [39]. Paired-end reads were then mapped to the scaffolds using GMAP [40]. After the mapping was performed, three consecutive steps of filtering were employed: (1) duplicated reads (both ends mapped to nearly the same positions of another read) were filtered; (2) reads of non-unique mapping were removed; and (3) reads overlapping with masked regions were discarded. Bambus was then used to link the scaffolds. Two special parameter settings were used to guarantee quality: at least 2 reads were required to link two scaffolds (default=2); and only 2 standard deviations of the insert size were allowed (default=3). Finally, a custom script was used to estimate the sizes of new N-gaps.

Step 4. **N-gap filling.** A new version of GapCloser (version 1.12) was used to close N-gaps in the derived assembly with all Illumina paired-end reads. According to the manual, GapCloser version 1.12 is less aggressive and more accurate than

GapCloser version 1.04. Moreover, we filtered Illumina reads with QUAKE [41] before feeding them to GapCloser. By default, GapCloser tries to narrow a gap by extending sequences, even when there is no expectation of closing it. We found that these extended sequences were more error-prone than those in the closed gaps; therefore, we used a custom script to remove these sequences. In the end, we obtained a haploid assembly of 450 Mb, with a scaffold N50 size of 1.5 Mb and a contig N50 size of 25 kb (Supplementary Table 1).

Quality comparison of haploid assembly versions 7 and 15. A statistical comparison of the two assembly versions is presented in Supplementary Table 2. A major improvement of version 2 over version 1 was the nearly doubled scaffold N50 size. The most notorious errors for polymorphic assembly are large-scale mis-joins. To detect large-scale discrepancies between the two assembly versions, we used LASTZ [42] and chainNet [43] to create pairwise whole-genome alignments between the two versions. A total of 320 large-scale (>100 kb) colinearity violations (or mis-joins) were identified from the alignments. To determine which version contained the mis-join, we also aligned each assembly version against the draft genome version 2 of the Florida amphioxus *B. floridae* [1]. The genome of Florida amphioxus served as an out-group due to its relatively conserved genomic structure and divergence time of ~110 million years from Chinese amphioxus. The 3-way comparison pinpointed 77 potential mis-joins in the v15 assembly and 66 in v7; and the remaining 177 potential mis-joins could not be determined (Supplementary Table 3). Nevertheless, these results suggested that despite the much longer scaffold size, the v15 assembly carried more potential mis-joins than the v7 assembly.

Haploid genome assembly version 18: hybrid methods

A comparative analysis of assembly versions 7 and 15 provided more information on how to fine-tune the assembly strategy described above. After testing a series of data and strategic combinations, we achieved a final assembly strategy (Supplementary Figure 2). The important changes are described below:

Hybrid assembly with multi-platform data. In assembly versions 7 and 15, Illumina paired-end reads were used only for gap filling. After extensive tests, we found that a hybrid assembly involving both 454 reads and Illumina reads can be very effective at increasing assembly accuracy and continuity. We believe that several factors may contribute to this property: (1) higher depth; and (2) the fact that per base quality increases not only because of higher depth but because Illumina reads correct specific sequencing errors inherent in 454 reads and *vice versa*, which, in turn, helps paired-end reads be placed in the correct positions.

Updates for HaploMerger. 1) The contig-scaffold layout was used to evaluate tandem mis-assemblies and suppress false positives; 2) in addition to large-scale mis-joins (>50 kb), middle-scale mis-joins (30-50 kb) were also detected and processed; 3) we no longer considered the mate-pair graph or the contig-scaffold layout because they can be misleading (we observed more potential mis-joins in version 15 than in version 7). On the other hand, we reasoned that the correct linkage of contigs should be re-linked in the second round of scaffolding; hence, the algorithm now breaks up both scaffolds involved

in a possible mis-join; and 4) tandem assemblies on the haploid assembly can now be detected and processed.

Diploid assembly. The version 18 diploid assembly was created using both 454 reads (shotgun, 2 kb, 3 kb and 8 kb paired-end) and Illumina reads (340 bp and 500 bp paired-end). The new assembly pipeline is shown in Supplementary Figure 2. The hybrid diploid assembly spans 707 Mb, with a scaffold N50 size of 264 kb and a contig N50 size of 30 kb.

Reference (or primary) haploid assembly. The hybrid diploid assembly was processed by HaploMerger. A total of 2,149 tandem assemblies (>100 bp; in total accounting for ~17.90 Mb or ~2.5% assembly size) were detected and removed by consulting the self-alignments and contig-scaffold layout. A total of 159 events of middle- to large-scale mis-joins (>30 kb) were detected, and all implicated scaffolds were broken up. Finally, HaploMerger selected the longer alleles for the initial haploid assembly, which was further scaffolded using 20 kb paired-end reads as described above (except this time requiring at least 4 mate-pairs to establish a link, thereby suppressing false positives). After scaffolding, HaploMerger was used to remove newly derived tandem mis-assemblies (588 events, accounting for 2.89 Mb). Finally, GapCloser version 1.12 was used to close N-gaps in the assembly. By default, GapCloser tries to narrow a gap by extending sequences, even when there is no hope of closing the gap. These extended sequences were more error-prone than those in closed gaps and were therefore discarded. The “gap-filling” contigs created by GapCloser were generally less accurate and hence marked with the prefix “GF_” in the companion AGP file. The final reference (or primary) haploid assembly has better N50 sizes for both scaffold and contig, namely, 2.3 Mb and 46 kb, respectively (Supplementary Table 2).

Alternative haploid assembly. HaploMerger produced ~291 Mb alignments (>500 bp) after solving the allelic relationships in the diploid assembly. This means that approximately 65~70% of the loci in the primary haploid assembly have an alternative allele. This proportion is significantly lower than that of the draft diploid genome of *B. floridae* [1], where 77~85% of genomic loci have an alternative allele. We believe that the difference reflects the nature of current next generation sequencing (NGS) technologies, where shorter read lengths and a higher sequencing error rate cause more severe allele sequence collapsing and demote many more sequences to degenerate status. We created an alternative haploid assembly by replacing corresponding loci in the primary haploid assembly with available alternative alleles.

Comparison of large-scale mis-joins in different haploid assembly versions. Based on 3-way whole-genome alignments, a total of 384 large-scale (>100 kb) colinearity violations (or mis-joins) were identified between haploid assembly versions 18 and 15. Using the draft genome version 2 of Florida amphioxus (*B. floridae*) as a reference, we pinpointed 27 possible mis-joins in assembly version 18 and 130 in version 15; the remaining 227 potential mis-joins could not be determined (Supplementary Table 3). This result suggests that assembly version 18 is much better than version 15 in terms of large-scale mis-assemblies. We also compared version 18 with version 7, testing at the smaller scale size of 50 kb; these results showed a similar trend (Supplementary Table 3). Based on these results, we estimated

that there was less than one potential mis-join (>100 kb) in every 6.5 Mb in the v18 assembly. Note that this estimate could be an overestimate due to mis-joins in the Florida lancelet genome assembly, alignment artifacts, true large-scale genetic variation, and other assembly errors, such as large indels taken for colinearity violations.

The completeness of the v18 assembly

Raw Illumina paired-end reads (~23 Gb) were aligned to the reference+alternative assembly and the reference assembly using GSNAP [44], which was run in the DNA mode with the provided insert sizes and all other default parameters. As individual reads, 99.82% of the Illumina reads were successfully mapped to the reference+alternative assembly. As read pairs, 98.32% were successfully mapped in the correct direction and distance range (i.e., concordant match). On the other hand, though 99.31% of the individual reads could be mapped to the reference haploid assembly, only 89.30% of the read pairs were mapped in the correct direction and distance range. These results suggest a large quantity of allele-specific reads, which is a reflection of the high polymorphism of the amphioxus diploid genome.

In addition, EST contigs, which were assembled from ~3 million 454 FLX Titanium reads (Supplementary Note 8), were aligned to the v18 assembly using NCBI-BLASTN [45]. Of 52,961 contigs with at least 300 base pairs and exactly one apparent open reading frame (ORF), 98.85% had at least one alignment of at least 80% identity and 25% coverage to the diploid assembly (or 96.55% at 80% identity and 50% coverage). The reference haploid assembly showed similar completeness (96.13% at 80% identity and 50% coverage, or 98.59% at 80% identity and 25% coverage), suggesting that the reference haploid assembly inferred by HaploMerger was almost as complete as the original diploid assembly in terms of protein-coding gene content.

Supplementary Note 3 Divergence between two lancelet species

Curation of multigene protein alignments

To evaluate the amino acid substitution rates and divergence times between two lancelets and other species, we extracted orthologous protein-coding gene families from fifteen selected species using a modified reciprocal best hit (RBH) method as suggested by Putnam *et al*[1] (see Supplementary Note 7 for more details). The initial ortholog families based on lancelets and humans were used as anchors to identify orthologs from 12 other species. At least 50% sequence coverage was required for every orthologous protein pair and only one species was allowed to be absent in each protein family. The final resulted dataset contains 729 ortholog families. CLUSTALW2 [46] was used to create multiple alignments for each family, and all alignments were concatenated to form an all-in-one alignment (alignment 1), which contained 729 ortholog families and a total of 403,674 sites. Alignment 2 (with 245,205 sites) was further created by removing the less-conserved sites (158,469 sites) from alignment 1 using Gblocks[47]. Finally, we removed sites with indels from alignment 2 and retained only ortholog families with representatives in all 15 species, which gave alignment 3 (with 72,795 sites from 513 ortholog families). Alignment 3 was used to estimate phylogenetic relationships, amino acid substitution and divergence times, whereas alignment 1 and 2 were only used for the estimation of amino acid substitution.

Protein-based phylogenetic reconstruction

Both Bayesian and maximum likelihood analyses were used for phylogenetic reconstruction. Bayesian analysis were carried out on alignment 3 using Phylobayes[48] v3.3 with the CAT model, which is a mixture model especially devised to account for site-specific features of protein evolution and hence particularly well suited for large multigene alignments. We ran four independent chains with random starting trees for over 20,000 Monte Carlo iterations (with the first 10,000 burin-in cycles removed), and they converged to the same tree topology (Supplementary Figure 3A). For maximum likelihood analysis, we first ran ProtTest3[49] on alignment 3 to select the best-fit model. The recommended model is LG+I+G+F, namely, the LG[50] amino acid substitution matrix plus invariant sites, Gamma distribution (under four rate categories) and empirical amino acid frequencies. PhyML[51] v3.1 was then run on alignment 3 to infer tree topology and branch length. Special settings for PhyML included 200 bootstrap replicates and the BEST topology search method. PhyML produced the same tree topology as obtained by the Bayesian method (Supplementary Figure 3B). The estimated Gamma-shape parameter and the invariant fraction were 0.83 and 0.16 respectively. Finally, amino acid substitution per site (branch length) based on alignment 1 and 2 was also calculated using PhyML based on the tree topology obtained from alignment 3 (Supplementary Figure 3C-D).

The analysis shows that both Bayesian and maximum likelihood methods recovered the same tree topology (Supplementary Figure 3A-B). And the relative amino acid substitution rates (branch length divided by total tree length) inferred from alignment 1, 2 and 3 were consistent with each other (Supplementary Figure 3B-D). Branch length indicates that the

protein divergence of two lancelets is comparable to the selected species pairs of worms (*C. elegans* and *C. briggsae*) and fruit flies (*D. melanogaster* and *D. mojavensis*), but is approximately half the divergence of those selected species pairs of tunicates (*C. savignyi* and *C. instestinalis*), fishes (tetraodon and stickleback) and human versus chicken (Supplementary Figure 3B-D). Since alignment 3 tends to bias to highly conserved amino acids and alignment 1 is overwhelmed by fast-evolving sites, we reasoned that the tree based on alignment 2 may provide a balanced estimation of amino acid substitution (Supplementary Figure 3C and Supplementary Table 4).

Protein-based divergence time analysis

We then used Bayesian methods to estimate the divergence time of two lancelet species between two lancelets based on alignment 3 and the inferred phylogenetic topology. Fossil-based divergence time constraints taken from literatures[52-54] were imposed on this dating analysis (human-chicken 312-331Mya, tetraodon-stickleback 98-151Mya, human-ray-finned fish 416-422Mya, tunicate-human >485Mya, lancelet-human >485Mya, echinoderm-vertebrate >521Mya, echinoderm-hemichordate >485Mya, drosophila-nematode >541Mya, protostome-deuterostome >558Mya). Lower bounds were used as hard bounds, whereas upper bounds, if available, were increased by 10% to make them “softer”. Two molecular dating programs and five parameter sets were run on the obtained tree topology and alignment 3 (Supplementary Figure 3E). PhyTime[55] v3.1 (from the package of PhyML) was run under the autocorrelated relaxed clock model with these parameters: the GBS rate model (Geometric Brownian + Stochastic), the LG matrix, Gamma distribution (16 categories), the multivariate normal approximation and 1,000,000 iterations (with 30% as burn-ins). Phylobayes[48] v3.3 was run for >100,000 iterations (with 30% as burn-ins) on four different parameter sets: the log-normal autocorrelated relaxed clock model with a uniform (1) or a birth-death (2) prior on divergence times, and the uncorrelated gamma model with a uniform (3) or a birth-death (4) prior on divergence times.

As expected, autocorrelation models (PhyTime and the Phylobayes parameter sets 1 and 3) produced similar results which were different from those of uncorrelation models (the Phylobayes parameter sets 2 and 4) (Supplementary Figure 3E). Anyway, two sets of estimates were largely consistent with each other and the differences were within the acceptable range. Both clock models agreed that the divergence time of two lancelet species should be in the range of 111-130 Myr (mean=120 Myr) (Supplementary Figure 3E). In consistence, the divergence time of two lancelets was estimated to be 112 Myr based on mitochondrial genome sequences[17,56]. And the geological separation time between Atlantic and Pacific oceans (the respective habitats of two lancelet species) is 100-130Myr[57].

A comparison of the amino acid substitution rates and the divergence times in six selected pairs of species (two lancelets, two worms, two fruit flies, two tunicates, two fishes and human versus chicken) was provided in Supplementary Table 4A.

Furthermore, as shown in Supplementary Figure 6, the distribution of pairwise divergence for all 1:1 ortholgous protein pairs shows that the protein divergence between two lancelets is

larger than human versus mouse (62-101 Mya) and human versus sheep (95-113 Mya), but smaller than human versus opossum (125-138 Mya). Therefore, both the amino acid substitution rate and the divergence time of two lancelet species are comparable to those of human versus sheep and human versus oppossum.

Amino acid substitution rates in different chordate lineages

According to the above phylogenetic analysis, tunicates are the fastest evolvers, while cephalochordates have the shortest branch length, and vertebrates fall in the intermediate range (Supplementary Figure 3A-D). These observations are consistent with previous reports.

Vertebrates went through fast substitution rates in the period between the split of cephalochordates and vertebrates and the separation of jawed and jawless vertebrates. In modern vertebrates, protein evolution is actually in a similar pace as lancelets (Supplementary Table 4B). This is also confirmed in our later analysis (the section "Pairwise orthologous protein divergence" below).

Supposing that the lancelet lineage diverged from other chordates 623 Mya and that the separation of the Florida and Chinese lancelet occurred 120 Mya, then using the distances shown in Supplementary Figure 3C, we can calculate that the amino acid substitution were largely the same before and after the divergence of two lancelet species (Supplementary Table 4B).

Supposing that the vertebrate-urochordate lineage diverged from the lancelet lineage 623 Mya, and that the separation of jawless and jawed vertebrates occurred 420 Mya, then using the distances shown in Supplementary Figure 3C, we can calculate that the amino acid substitution rates before the separation of jawless and jawed vertebrates are 2-4 times higher than that after this point (Supplementary Table 4B).

Moreover, considering that human and chicken diverged 319 Mya, and that the Florida and Chinese lancelets diverged 120 Mya, then using the distance showed in Supplementary Figure 3C, we can calculate that average amino acid substitution rates in human or chicken after their divergence is lower than those in Florida or Chinese lancelets (Supplementary Table 4). This is also confirmed in our later analysis (the section "Pairwise orthologous protein divergence" below).

Protein sequence divergence between the two lancelet species

Pairwise alignments of 11,589 orthologous gene pairs (1:1), which covered at least 60% of the protein length, were used for protein sequence identity analysis (Supplementary Figure 4). These calculations revealed that the mean protein identity between two lancelets is 81.2%, with a median of 84.0%. The sequence identity of approximately 30% of the protein pairs is higher than 90%.

Coding sequence divergence between the two lancelet species

We also converted the protein alignments into the corresponding coding sequence alignments and re-calculated sequence identities (Supplementary Figure 4). The estimated mean coding DNA identity between the two lancelets is 79.5%, with a median of 83.0%. Approximately 16% of the coding sequence pairs have an identity of more than 90%.

Intron sequence divergence between the two lancelet species

Using the above-obtained 11,589 orthologous gene pairs for the two lancelets, we further extracted a set of 23,021 high-confidence orthologous intron pairs. For each intron pair, we performed a pairwise BLASTN analysis (with a penalty of -1 and a cutoff E-value of 10) and recorded the alignment identity and coverage. The results showed that only 1.5% of the intron pairs could produce an alignment covering more than 50% of the intron length (Supplementary Figure 5). A plot of identity against coverage confirmed that the intron sequences of the two lancelets have virtually no similarity (Supplementary Figure 5). In particular, among the 12,533 (54% of the total intron pairs) aligned intron pairs with at least 66% identity, 88% (11,024) could not produce an alignment covering >25% of the intron length.

Protein sequence divergence in different functional categories

To evaluate how protein sequence divergence varies between different functional categories, we classified proteins into functional categories (GO terms) and calculated their mean protein protein identities and d_N/d_S ratios (Supplementary Table 5). In the “cellular component” class, extracellular and cell membrane-bound proteins are the most divergent, whereas proteins within the nuclei, macromolecular complexes and membrane-enclosed lumens are the least divergent. In terms of molecular function, those involved in signaling and transducer activity evolve at the fastest pace. As to biological process, proteins associated with rhythm, metabolism, cellular component biogenesis and organization are highly conserved; in contrast, proteins related to signaling transduction, growth, immunity and anti-stimulus, reproduction and adhesion are the most divergent (Supplementary Table 5).

Pairwise orthologous protein divergence

In addition to the analysis of core protein-coding gene divergence, we wanted to understand the divergence of orthologous protein pairs between closely related species, which could yield a more complete picture of recent protein evolution. We selected six species pairs for comparison. The soft-masked genome sequences and the complete protein set of opossum and sheep were downloaded from ENSEMBL. We identified 1:1 orthologous protein-coding gene pairs that covered at least 50% of the protein length from each species pair. We then plotted their distance (simple computed by 100-Identity) against the normalized accumulated gene number (Supplementary Figure 6). The following species pairs were evaluated:

	Gene pairs	Average identity
D. melanogaster–D. mojavensis	9887	73.11
C. elegans–C. briggsae	13056	75.49
Two lancelets	15123	81.01
Human-opossum	15233	77.55
Human-mouse	16767	84.14
Human-sheep	16372	84.58

The results show that the orthologous protein divergence between the two lancelet species is between that of human versus opossum and human versus mouse or sheep (Supplementary Figure 6). The divergence time between human and mouse (61.5-100.5 Myr) is shorter than that between human and sheep (95.3-113 Myr), but the mouse is known to evolve faster than the sheep or human. The divergence time between human and opossum is estimated to be 124.6-138.4 Myr. Chinese and Florida lancelets are thought to have split 120 (111-130) Mya. We varied the parameters for the analysis but obtained similar results. Therefore, the protein evolutionary rate of lancelets is roughly as fast as, if not faster than, mammals.

By comparing the distribution of protein divergence in six species, we found that lancelets have relatively more divergent protein sequence pairs than human versus other tetrapods. This trend is most clear for human versus opossum: though the protein identity of human versus opossum is on average 3.5% higher than that of the two lancelets (see the above table), they have a similar fraction of protein pairs with an identity higher than 50%. This diversifying pattern is also reflected by the fact that though >90% of genes have homologs between the two lancelets (Supplementary Note 9), only ~50% formed stable orthologous pairs in this analysis.

Supplementary Note 4 Polymorphism within the population

Global statistics

To characterize the heterozygosity, a hybrid assembly (version 18) was created from all 454 and Illumina read data using CABOG. The resulting diploid assembly spanned 707 Mb, with a scaffold N50 size of 264 kb, a contig N50 size of 30 kb and a contig sequencing depth of 30x coverage. Allelic relationships within the diploid assembly were reconstructed using HaploMerger [31]. HaploMerger also used the LASTZ and chainNet programs to generate pairwise reciprocal-best alignments for each allele pair. The chainNet alignments (>500 bp) spanned a total of 291 Mb. Approximately 272 Mb of long alignments (>10 kb) were further refined by MUSCLE [58], of which 182 Mb alignment stretches (>1000 bp) free of both sequencing gaps and overlapping alignment gaps were used for SNP and indel polymorphism analysis.

The mean difference rate between the aligned allele sequences is 13.31%, with 4.02% as single nucleotide mismatches and 9.29% as small indel-caused length differences (indels of size ≤300 bp; 96.4% were <50 bp and accounted for 4.90% of the length differences). If indels were treated as point differences, the nucleotide substitution rate increased to 4.39% and the indel rate decreased to 0.98%, giving a total mean allelic polymorphism rate of 5.37% — more than 50 times the rate in humans but comparable to the rates of the Florida lancelet (~4.0%) and the tunicate *C. savignyi* (~5.6%) [1,29].

The alignments were analyzed using a 50 bp sliding window with a step size of 25 bp (Supplementary Figure 4), from which we observed a mean allelic difference of μ=2.67, with a variance of σ^2=6.84. This level of polymorphism is closer to a geometric distribution (μ=2.67, σ^2=9.79) than to a Poisson distribution ($\mu=\sigma^2$=2.67). Further analyses with window sizes of 100 bp and 200 bp showed similar variation patterns (Supplementary Figure 7-9). Therefore, the polymorphism rate between the two haplotypes is not only high but also highly variable across regions. In particular, 50% of the 50 bp regions contribute only 10% of the polymorphic sites, whereas 20% of the 50 bp regions account for over 50% of the polymorphic sites. From the point of genome assembly, such large regional variation will make the assembly of the lancelet polymorphic diploid genome much more difficult than the assembly of mixed data from two closely related species (e.g., human and rhesus).

According to the coalescent theory, divergence between species usually fits a Poisson distribution [59], whereas divergence between haplotypes in a freely mixing population of constant size tends to be geometrically distributed. According to this theory, to produce a polymorphism rate of approximately 5.4%, an effective population size of millions of individuals is required (for a mutation rate of 1e-9: $N_e=\theta/(4\mu)\approx 0.054/(4*10^{-9})=13.5*10^6$; for a mutation rate of 1e-8: $N_e=\theta/(4\mu)\approx 0.054/(4*10^{-8})=1.35*10^6$). Such a size is immense but possible in the animal kingdom. For example, similar polymorphism rates and distribution patterns have been observed in other marine invertebrates, such as Florida amphioxus [1] and *Ciona savignyi* [29].

Indels are common differences between haplotypes and affect one tenth of the alignment length despite an occurrence rate of only 0.98%. The size distribution of polymorphic indels obeys the power law (Supplementary Figure 10), consistent with indels between mammalian genomes [60]. Remarkably, 6-bp indels are excessively more common than expected and contribute the highest total sequence differences among all indel sizes (Supplementary Figure 10).

The two haplotypes also differ by many large indels that span hundreds and thousands of base pairs. We used the original chainNet alignment (the original net file created by HaploMerger) to analyze these large indels. In particular, we examined polymorphic indels 200-1500 bp in length and found a total of 28,652 indel events that contributed to 4.3% of the total alignment length difference (Figure 10).

We also analyzed the distribution of length of ungapped alignments between haplotypes (Supplementary Figure 11). This analysis revealed that the mean length of ungapped alignments is 95 bp and that, in terms of total length, the most abundant length of ungapped alignments is 36 bp.

A total of 9,490 translocation events (>100 bp and inversions excluded) were detected between the two haplotypes using the chainNet alignments, accounting for over 12.5 Mb (~4.3%) of the alignments. The size distribution of these translocations roughly obeys the power law (Supplementary Figure 12), with 3,087 cases larger than 1000 bp.

We detected a total of 700 inversion events (>100 bp and other translocations excluded) that account for over 2.5 Mb (~0.85%) of the alignments. The size distribution of these inversions roughly obeys the power law (Supplementary Figure 13), with 255 cases larger than 1000 bp.

Finally, in another analysis (Supplementary Note 13), we show that the rate of structural variations (translocations and inversions) within Chinese lancelets is ~10 times higher than that between human and rhesus (~5% sequence divergence between human and rhesus) and ~30 times higher than that between human and chimpanzee (~1.5% sequence divergence between human and chimpanzee).

Large polymorphic indels and transposable element activity

We visually examined 310 random polymorphic indels (>200 bp) and found that 210 could be identified as potential transposable element (TE) insertions or deletions. This visualization also revealed that while many alignment gaps can be readily ascribed to TE activities (Supplementary Figure 14), many others are caused by N-gaps, reciprocal gaps and possibilities not readily explained (e.g., mis-assemblies).

We then created a new chainNet alignment between the two final haploid assemblies. Here, there were 36,859 events of large polymorphic indels (300-10000 bp), affecting 37,868,997 bp of the genome assembly. A comparison with the TE annotation showed that 65-77%

(depending on different criteria) of indels are associated with TE activity (Supplementary Table 6).

Synonymous and nonsynonymous polymorphisms in Chinese lancelets

High-confidence pairwise DNA alignments for coding regions were created for each protein-coding gene pair from the two haploid assemblies. Gaps and Ns were removed from the alignments. Alignments of genes were treated separately or as concatenated alignments, depending on the analysis. PAML v4.5 was used to infer the synonymous diversity (dS) and non-synonymous diversity (dN) and their ratios. Both the Nei & Gojobori (1986) method and the Yang and Nielsen (2000) method were used.

There are some difficulties inherent in finding exact, genuine 1:1 orthologous gene pairs: 1) the gene models often fragment into pieces; 2) some gene families underwent multiple tandem duplications that make it difficult to determine orthologous gene pairs; 3) both polymorphic (selected) duplications, deletions and non-functionalization of genes are present in different haplotypes; and 4) there is some fraction of false predictions and false frame calling. We therefore focused on those gene pairs with clear hits to the gene ontology (GO) protein database. One advantage of this procedure is that it allows us to directly assess d_N/d_S ratios in different functional categories (GO terms).

As shown in Supplementary Table 7, the average synonymous diversity for Chinese lancelet genes was estimated to be 0.070-0.075, depending on different criteria; the corresponding d_N/d_S ratio was 0.067-0.089, compared with 0.07 for *C. savignyi*[61], 0.14 for zebrafish[62], 0.15 for *D. melanogaster*[63] and 0.35 for human[64].

The average d_N/d_S ratios for proteins of different functional categories are also given in Supplementary Table 5.

Supplementary Note 5 Whole-genome re-sequencing of five Chinese lancelets

Sample collection and re-sequencing

We collected five additional adult Chinese lancelets for whole-genome re-sequencing and bisulfite sequencing. The procedures of sample collection and DNA isolation was the same as described in Supplementary Note 2, except that here we purified DNA from the whole body without gonads. Samples were collected from two locations: Xiamen and Zhanjiang (near Beihai) that are ~1000 kilometers apart (Supplementary Figure 1; Supplementary Note 1).

Three animals from Zhanjiang were sequenced by the Illumina Hiseq2000 platform (2x 101 bp); approximately 30 G filtered data were generated for each animal (Supplementary Table 8).

Two animals were collected from Xiamen, the same place where the lancelet for the reference genome assembly was collected. These two animals were first sequenced by the Illumina Hiseq2500 platform (2x 151 bp); approximately 45 G filtered data were generated for each animal (Supplementary Table 8).

Multiple whole-genome alignment of six individual genome sequences

The diploid genome status and the high heterozygosity of the Chinese lancelet genome posed great difficulties for our genome-wide comparison between individual genome sequences. For example, common tools for short-read mapping and SNP calling were designed based on genomes with lower polymorphism rates (e.g., the human genome). To work around this predicament, we used the multiple whole-genome alignment approach.

First, we used the Celera assembler [35] to create a *de novo* diploid genome assembly for each re-sequenced individual lancelet. The procedure and parameter settings were basically the same as described in Supplementary Note 2, except that we used the BOGART module, which is supposed to handle short Illumina reads better, and we did not perform hierarchical assembly and gap-filling. The obtained diploid assemblies (scaffolds plus degenerate contigs) range from 600-750 Mb, with a scaffold N50 length range between 2 and 6 kb and a contig depth of over 30x coverage. For comparison, we also used the SOAPdenovo assembler [32] for the task, which produced assemblies with smaller contig N50 lengths.

Second, we created reciprocal-best pairwise whole-genome alignments between the reference genome and the re-sequenced genomes using the LASTZ-chainNet method. To guarantee the SNP calling accuracy, we masked low-quality nucleotides (i.e., quality value <40) in the assemblies based on the quality files provided by the Celera assembler. Repeats in all genome sequences were then soft-masked, and LASTZ was used to create whole-genome DNA alignments. LASTZ was tuned to maximum sensitivity and specificity with the following

special parameter settings: –masking=0, –hspthresh=3000, –ydrop=3400, –gappedthresh=3000, –gap=400,30, –step=1, –seed=12of19, –identity=90, and the score matrix “100 -300 -300 -300; -300 100 -300 -300; -300 -300 100 -300; -300 -300 -300 100”. In addition, to obtain a minimum alignment identity of 90%, we also required at least 1000 matches to further suppress false alignments. The LASTZ alignments were processed into reciprocal-best single-coverage chainNet alignments according to UCSC’s documentation (also implemented in our HaploMerger software [31]). Special parameters for axtChain and chainNet included –linearGap=medium, –minScore=15000.

Third, we created six-way multiple whole-genome alignments including the reference genome and the five re-sequenced genomes. Multiple alignments were constructed using TBA (parameters: E, null, P, multic, a guide tree ((bbv18ref, bbe23a, bbe23f), (bbe01, bbe03, bbe06)) and all others as defaults) [65]. Finally, only those alignment blocks containing all six individuals were kept for further study. This procedure is similar to our previous work [66].

SNP rates, population structure and natural selection

The six-way alignment contains ~50 Mb gap-free and N-free alignments. This is only slightly more than 1/8 of the total genome size. Potential causes responsible for this small set of alignments include: 1) the fragmented re-sequenced genome assemblies and our requirement of at least 1000 bp matches for each pairwise alignment; 2) the repeat-masking; 3) multiple alignment blocks for analysis were required to contain all six individuals and be longer than 200 bp; and 4) the exclusion of gap-containing alignments, N-containing alignments and alignments near the terminals of an alignment block (20 bp).

This six-way alignment contains three individuals from the Xiamen population (including Bbv18ref (the reference genome), Bbe23a and Bbe23f) and three individuals (Bbe01, Bbe03 and Bbe06) from the Zhanjiang population. Our analysis revealed that in this alignment, the SNP rates per nucleotide (=p-distance) between any two individuals were almost the same (Supplementary Table 34). Though Bbe23a and Bbe23f were slightly more similar to the reference genome than Bbe01/03/06, the difference is trivial. We also analyzed protein-coding regions (~3.2 Mb) and obtained similar results (Supplementary Table 9). These findings suggested that the genetic structure within Chinese lancelets is very weak, if not absent, despite their large habitat (Supplementary Note 1), consistent with the analysis of mitochondrial sequences (Supplementary Figure 1B; Supplementary Note 1).

As estimated for the ~50 Mb gap-free and N-free multiple alignments, the average SNP rates between two individuals (i.e., nucleotide diversity) were 4.86%, close to the estimate obtained from the individual used for the reference genome assembly (namely, between the reference genome assembly and the alternative assembly). This rate is dropped to 3.19% when we only counted protein-coding regions (Supplementary Table 9).

We performed a Tajima's Neutrality Test with the protein-coding region alignments (~3 Mb). The segregating sites per nucleotide is 0.031629, the nucleotide diversity $E(\pi)$ is 0.031808, and Tajima's D is 0.043276. This suggests that under the neutral theory, there has not been a

recent bottleneck crisis, and no recent population admixture has occurred in Chinese lancelet populations. The use of all alignment sites for this analysis yielded the same conclusion.

We also performed the d_N/d_S analysis using the protein-coding region alignments (~3.2 Mb), as described in Supplementary Note 4. This analysis revealed that the d_N/d_S ratio between individuals is approximately 0.083, i.e., very strong purifying selection (Supplementary Table 35) and consistent with the rate between the two haplotypes of a single individual (Supplementary Table 7).

Supplementary Note 6 Repeats analysis

Identification of transposable elements (TEs) in *B. belcheri* and *B. floridae*

Two *de novo* repeat family identification and modeling packages, RepeatModeler (http://www.repeatmasker.org/RepeatModeler.html) and REPET [67], were used to identify novel repeat families from the diploid genome. RepeatModeler invokes three programs (RECON [68], RepeatScout [69] and Tandem Repeat Finder [70]) to identify novel repeats and then combines their results into a non-redundant set of repeat families. RepeatModeler initially reported 1178 candidate families. After manually filtering out protein-coding genes, we obtained a set of 481 novel repeat families. In addition, the TEdenovo pipeline from the REPET package produced a set of 7639 non-redundant clusters of novel repeat sequences.

The same procedure was applied to the genome sequence of *B. floridae* to obtain a set of candidate repeat families.

We also downloaded the repeat library of *B. floridae* from the JGI website (http://genome.jgi-psf.org/Brafl1/Brafl1.download.ftp.html) and extracted known repeat families of other deuterostomes from the RepBase dataset (repeatmaskerlibraries-20130422.tar.gz) [71]. The two datasets were combined.

The two *de novo* datasets (*B. belcheri* and *B. floridae*) and the downloaded dataset were combined into a single library, which was then used to screen amphioxus genome sequences using RepeatMasker [72]. RMBlast and option –s were used in the homologous search.

Genome-wide profile of amphioxus TEs

Window-based analysis by WinMasker [39] was first employed to estimate the proportion of repetitive DNA sequences in the reference genomes of *B. floridae* and *B. belcheri*. This analysis showed that 29.5-30.2% of the reference genomes could be repetitive DNA sequences.

We then used RepeatMasker and the curated TE library to screen the *B. belcheri* reference genome. According to the search results, satellites, simple repeats, and TEs constitute at least 27.6% of the diploid assembly. In the reference assembly, TEs comprise of 26.9% of the genome. The discrepancy between the WinMasker results and the RepeatMasker results suggest that there are some unknown repetitive sequences not identified by RepeatMasker. Notably, DNA transposons (12.7%) are more abundant than retrotransposons (10.3%), which is different from mammals but similar to some invertebrates (Supplementary Figure 15).

The same procedure was applied to the *B. floridae* reference genome. Its composition pattern largely recapitulates that of the genome of *B. belcheri*, with ~26.6% TE content in total, 12.6% for DNA transposons and 9.5% for retrotransposons (Supplementary Figure 15), hence suggesting a conserved trend for TE evolution in the amphioxus lineage. Note that the

genome sizes of the two lancelets differ by 15-20%.

The composition and classification of amphioxus TEs

We proceeded to identify and classify the lancelet TEs into superfamilies. However, because the lancelet TE content is a very complex entity, here we chose to mainly focus on TE families that have homologs in other species and encode proteins that are necessary for TE mobilization. In other words, non-autonomous TEs, including miniature inverted repeat TEs (MITEs) and short interspersed nuclear elements (SINEs), were not considered in this study if they do not clearly belong to any protein-coding TE families. In addition, though at least 4-7% of the genome content could consist of unknown TEs (Supplementary Table 10), the identification of novel TEs or lancelet-specific TEs was not our purpose here.

Though TEs account for 26-30% of the contents of both the Florida and the Chinese lancelet genome assemblies, only a few intact (complete) or nearly intact autonomous (protein-coding) TEs could be identified. Most TEs presented as fragments, overlapped or nested within each other and containing defective coding regions or completely devoid of coding sequences. As a side effect, this phenomenon not only makes it difficult to identify TEs but makes TE numbers and TE total length counting less reliable. Three possibilities may account for this phenomenon.

1. Errors in the genome assembly. However, this is unlikely to be the major reason because the phenomenon occurs in both the Florida and the Chinese lancelet genome assemblies, and the Florida lancelet assembly was created using the traditional Sanger method, and the Chinese lancelet assembly was sequenced to a depth of 100x coverage (containing both 454 reads and Illumina reads) and showed good continuity (contig N50 size=46 Kb). In fact, even in the well-resolved regions (no gaps, no N, no simple repeats) in the current lancelet assembly, intact TE copies are still rare. Our assembly's contig N50 size (~46 kb) is better than that of many non-polymorphic genome assemblies. The phenomenon thus appears not entirely attributable to the assembly quality.
2. Another possibility is that intact TEs hid in the "dark" regions of the genome, e.g., the heterochromatin and the regions crammed with TEs and simple repeats; these regions are intractable to the whole-genome shotgun strategy. We estimated that the dark matter could include 442 Mb-(426 Mb+416 Mb)/2=21 Mb.
3. A third possibility is that all of the TE content in lancelets is in fact produced by a few active intact autonomous TEs.

In Florida and Chinese lancelets, we, respectively identified 1,233 and 1,087 TEs containing complete or partial TE proteins. An analysis of the protein architectures of these elements let us unambiguously identify 19 TE superfamilies: 5 LTR superfamilies (Gypsy, Copia, BEL/Pao, DIRS and Penelope), 4 LINE superfamilies (L1, I/LOA, REX1 and R2), and 10 DNA transposon superfamilies (TcMar/pogo, hAT, PiggyBac, PIF/Harbinger, Mule/MuDR, Merlin, EnSpm, Chapaev, Helitron and Polinton).

By phylogenetic analysis and comparison with the RepBase data, we further identified 1 LTR

superfamily (ERV), 8 LINE superfamilies (L2/Crack, L3/CR1, Jockey, RTE/RTEX, Proto2, Hero/NeSL, Daphne and Ingi/Vingi) and 11 DNA transposon superfamilies (Academ, Ginger, Kobolok, ISL2eu, IS4eu, P, Zator, Novosib, and Sola 1, 2 and 3). Note that protein domains for ERV and Novosib remain undetected thus far.

In an analysis of large polymorphic indels, we identified DNA transposons that encode the recombination activating gene 1 and 2 (RAG1 and RAG2).

In total, we identified at least 40 TE superfamilies (18 retrotransposons and 22 DNA transposons) that are all conserved in the *B. belcheri* and *B. floridae* genomes (Supplementary Table 10) and could be found in other species. It is also apparent that no TE members or families underwent drastic expansions or contractions (Supplementary Table 10), as previously reported in *B. floridae* [73]. Taken together, lancelets have a higher TE diversity than vertebrates and other invertebrates. However, there are certain compositional differences in different TE superfamilies between the two lancelet species (Supplementary Table 10).

In addition to the 40 high-confidence superfamilies, by comparison with RepBase, we also detected some small DNA fragments with weak homology to other TE superfamilies, including Ambal, CRE, RandI, Proto1, Kiri, R4 and Tad1.

Expression of TE protein-encoding transcripts

We assembled ~300 million EST reads or read pairs using Cufflinks and Trinity (Supplementary Note 8). The assembled transcripts were compared with our curated TE protein set using BLASTX with a cutoff expectation value of 1e-5. Under these cutoff criteria, there were only four superfamilies with no detectable expression: ERV, Copia and Novisib (these three have no detectable protein coding sequences in the current Chinese lancelet genome assemblies) and ISL2EU. When we lifted the cutoff criteria to 40% coverage and 40% identity, a total of 1,251 TE protein-encoding transcripts from 28 superfamilies were identified. At 50% coverage and 50% identity, a total of 757 TE protein-encoding transcripts were identified, distributed in 26 of the 40 TE superfamilies (Supplementary Table 10). At 60% coverage and 60% identity, 411 transcripts from 26 TE superfamilies were identified.

We also attempted to identify retrotranscriptases and transposases from the reference haploid genome of *B. belcheri* (version 18) using RPS-BLAST with all retrotranscriptase/transpose Pfam domains. Under the cutoff E-value of 1e-5 and with at least 55% coverage and 50 amino acids, we obtained 2,300 retrotranscriptase gene fragments and 415 transposase gene fragments. Comparison of these gene fragments with the raw EST genome mapping data, we found that 68% of the retrotranscriptase gene fragments and 73% of the transposase gene fragments were transcribed.

Relationship between polymorphic indels and TEs

We identified 36,859 large polymorphic indels (≥300 bp and ≤10,000 bp) between the two Chinese lancelet haploid assemblies. These indel sequences were compared to the curated TE

library using BLASTN. At an E-value of 1e-10 and a coverage of 30%, 28,481 (77%) indels could be ascribed to TE insertions, whereas at an E-value of 1e-10 and a coverage of 50%, 23,836 (65%) indels could be ascribed to TE insertions (Supplementary Table 6; Supplementary Note 4). Further analyses showed that only three TE superfamilies have no representative in these indel sequences: Merlin, Novosib and ERV. This analysis also led to the identification of the long-lost, legendary RAG transposon.

Supplementary Note 7 Genome rearrangement

The amphioxus genome has been shown to share deep conservation of global architecture with vertebrate genomes [1]. To understand the pattern of the evolution of the amphioxus genome architecture, we compared genome rearrangements in eight species pairs.

Orthologous gene/protein families for each species pair

We selected seven pairs of species for gene (protein)-based genome rearrangement analysis (Supplementary Table 12). Genome sequences, proteins and GFF files for *B. floridae* were downloaded from the JGI website (http://genome.jgi-psf.org/Brafl1/Brafl1.home.html). Data for the other species were downloaded from ENSEMBL (http://www.ensembl.org/), release 64. Orthologous gene families for each pair of species were identified using a modified reciprocal best hit (RBH) method similar to the protocol previously described [1].

First, for each pair of species, A and B, all-against-all reciprocal BLASTP was performed on all protein sequences for both directions (species A to species B and *vice versa*). For a gene with multiple protein variants, all variants were subjected to BLASTP but only the best hit among all variants was selected to represent the gene. Segments of alignments between two genes were concatenated, and the cutoff criteria were set to 60% identity and 40% coverage.

Second, orthologous gene pairs between each species pair were identified using the RBH method. If the best hit of gene A1 in species A is gene B1 in species B, i.e., S(A1,B1)=S(A1), where $\mathrm{S(A1)} = \max_{\mathrm{C\ in\ species\ B}}[\mathrm{S(A1,C)}]$, and the best hit of gene B1 in species B is also gene A1 in species A, i.e., S(A1,B1)=S(B1), where $\mathrm{S(B1)} = \max_{\mathrm{C\ in\ species\ A}}[\mathrm{S(B1,C)}]$, then A1 and B1 form an orthologous gene pair.

Third, we used a C-value of 0.7 to include the second best hit. For a pair of genes S(A1,B2), the C-value is calculated as follows: C(A1,B2)=S(A1,B2)/max(S(A1),S(B2)). If C(A1,B2)>0.7 and S(A1)>S(B2), B2 will join with A1 and B1 to form a larger orthologous gene group. This process was continued until there was no more new joining.

Oxford grams

An Oxford gram showing gene rearrangements was drawn according to the orthologous gene relationships. The X-axis shows the position of genes from species A, and the Y-axis shows the position of genes from species B. Each gene pair in the orthologous gene groups corresponds to a point in the Oxford gram. Note that a gene may have multiple points to show its second-best hits.

Because the draft genomes of *B. belcheri* and *B. floridae* are only available at the scaffold level, we used a method to cluster the orthologous scaffolds. For each pair of scaffolds (FA1

in species A, FB2 in species B), each orthologous gene pair (A1,B1) was assigned a pair of values (a1,b1): a1=1 if A1 is in FA1 and 0 otherwise; b1=1 if B1 is in FB1 and 0 otherwise. A Fisher's exact test with Bonferroni correction was applied on all pairs of values to generate a p-value for each pair of scaffolds or chromosomes. The dissimilarity is defined as -log(P), where P is the p-value for the pair of scaffolds. The scaffolds were then bidirectionally clustered using a hierarchical cluster method, implemented by the function 'hclust' in R (http://r-project.org).

Double cut and join (DCJ) distances

Genome rearrangement events can be measured using the edit distance, which is defined as the minimum number of rearrangement events necessary to transform one genome into another. Double cut and join (DCJ) distance and its efficient calculation were introduced by Yancopoulos (2005) [74]. DCJ distance differs from other distance metrics in that it includes chromosomal fusion, fission, inversion, translocation, and block interchange in a single model and allows simpler algorithms for calculations.

The orthologous gene pairs for each species pair were used to infer DCJ distances. The calculation followed the standard algorithm [74] and was implemented in our software AliquotG[75] (http://mosas.sysu.edu.cn/genome/download_softwares.php#). For the amphioxus genomes, only those scaffolds containing >30 genes were used for calculation.

Patterns of gene rearrangement in amphioxus

The urochordate pair, *C. intestinalis* and *C. savignyi*, shows the most drastic changes in genome architecture, with a DCJ distance up to 0.4 (Supplementary Table 11), whereas the human-chicken pair and the fish pair show the lowest genome rearrangement rates relative to their protein divergences (Supplementary Table 11). The amphioxus pair, the worm pair (*C. elegans and C. briggsae*) and the fly pair (*D.melanogaster* and *D. mojavensis*) show similar DCJ distances and protein divergences (Supplementary Table 11), suggesting that the genome rearrangement rate of the amphioxus lineage is similar to those of the protostome invertebrates. Because both amphioxus genomes were separated into hundreds of scaffolds, the rearrangement rates for the amphioxus lineage could be overestimated. However, the scaffold number used for amphioxus is approximately (186/2+195/2)=191, or one tenth of the total rearrangement events observed between the two lancelets (Supplementary Table 11), suggesting that the overestimation will not be more than 10%.

DCJ distance does not discriminate between large-scale and small-scale rearrangements. Large-scale rearrangements, including chromosome fusion, fission, and genes translocating to a distant site (e.g., another chromosome), often tend to shatter the original gene synteny, whereas small-scale rearrangements usually scramble the local gene order and hence leave syntenic relationships maintained on a large scale. Therefore, we further visually compared the syntenic relationships of these closely related species pairs by plotting their chromosomal homology on Oxford grids (Figures S16-21).

The results show that rearrangements in worms and fruit flies are highly restricted within chromosome arms, whereas in urochordates, both large-scale and small-scale rearrangements are common. Vertebrates have low rates of rearrangements, but a substantial number of genes have translocated outside their original chromosomes.

Through Oxford grams and hierarchical clustering of scaffolds, we observed that the rearrangement pattern in the amphioxus lineage is more similar to that of worms and fruit flies than those of vertebrates: more rearrangements are restricted within a small scale. This pattern partly explains why amphioxus still shares a high degree of synteny conservation with vertebrate genomes, despite their divergence time of over 550 million years.

Vertebrate rearrangement rates slowed down after the 2R-WGD

We next wanted to estimate how many DCJ gene rearrangement events occurred in vertebrates after the two rounds of whole-genome duplication (2R-WGD) in the early evolution of this lineage. This problem can be addressed by solving the genome aliquoting problem with double cut and join metrics [76]. We have developed an improved heuristic algorithm (i.e., AliquotG) for the genome aliquoting problem for 2 rounds of duplication (R=4) [75], but AliquotG version 1 can only handle those genes with all four ohnologs (duplicates from a whole-genome duplication) retained, i.e., AliquotG cannot use genes with only 2-3 ohnologs left. Here we developed an upgraded AliquotG algorithm (version 1.5, available on demand), which can use genes with 2-4 duplicates in a single genome to infer the pre-2R-WGD gene order.

The procedure is similar to the previous report [75]. Briefly, all proteins (including variants) from a vertebrate were BLASTed (the RBH method described above) against themselves and against the lancelet proteins (E-value: 1e-5; -F “m S”). The lancelet proteins were used to identify the 1:2, 1:3 and 1:4 paralogs. These paralogs were used to estimate the number of rearrangement events that have occurred since the 2R-WGD. Three vertebrate species were analyzed: human, mouse and chicken. Zebrafish and other teleost fishes were not used because they underwent another lineage-specific whole-genome duplication that aliquotG cannot handle. The rearrangement distances between human and mouse, mouse and chicken and human and chicken were also calculated.

These DCJ rearrangement rates were then used for distance tree reconstruction with the Neighbor-join method (Supplementary Figure 22). Considering that human and chicken diverged 312-33 Mya, human and mouse diverged 61-102 Mya, and the 2R-WGD event happened 450-500 Mya, we estimate that the relative rearrangement rate between the 2R-WGD and the human-chicken divergence was 4-6 times faster than the rates after the human-chicken divergence (p<1e-10, chi-square test) (Supplementary Figure 22).

Synteny of the Hox and the protoMHC gene clusters in lancelets

As shown above, the lancelet genome displays an average genome-wide gene rearrangement rate (0.23 per gene) close to those of other invertebrates and a local gene order scrambling

pattern that is also similar to other examined invertebrates.

However, the local rearrangement rate is highly variable in lancelets. One remarkable example is the Hox gene cluster, which contains 17 genes (Hox1-15 and EvxA and B) and shows no rearrangement between the two lancelet species.

Another notable example is the protoMHC region. The origin of the vertebrate MHC region, or the MHC big bang, represents a critical event in vertebrate evolution and the rise of adaptive immunity [77-79]. Here, by comparative analysis of different assembly versions of the Chinese lancelet genome, we identified the entire protoMHC region (in three corrected scaffolds). This region contains 269 MHC-related genes that are conserved between the human and lancelet genomes. Remarkably, though this protoMHC region shares good synteny with the four human MHC paralogous regions, the gene rearrangement rate in the lancelet protoMHC region is as high as 120/269=0.45 per gene, twice as high as the average genome-wide rearrangement rate in the lancelet genomes. In addition to the 269 MHC-related genes, we also used all genes in this region to recalculate the local gene rearrangement distance, or 382/816=0.47. Taken together, there is active local gene order scrambling in this protoMHC region. This active rearrangement was most likely important for the so-called MHC big bang in vertebrates, from which the MHC type I &II molecules and the Ig C1 domain derive [79].

Supplementary Note 8 Transcriptome sequencing and processing

RNA preparation and sequencing

Due to the small body size of lancelets, we collected RNA samples from multiple individuals. Total RNA samples purified from harvested tissues using the QIAGEN RNeasy plus midi kit were treated with Promega DNaseI and then used for mRNA isolation with the Oligotex mRNA mini kit (QIAGEN). The obtained polyA mRNA samples were analyzed by Nanodrop and Agilent Bioanalyzer 2100 (using RNA Nano 6000 chip) to ensure an OD260/280 above 1.8 and an RIN above 8.5.

For 454 sequencing, three random-primed cDNA libraries (2 from adult bodies and 1 from embryos of various developmental stages) were prepared using random hexamers and the Roche cDNA synthesis system. Sequencing was performed following the GS FLX Titanium protocol and yielded approximately 3 million high-quality titanium reads (Supplementary Table 1).

In addition, eleven cDNA libraries were synthesized using the Truseq™ RNA sample preparation kit and sequenced by an Illumina Genome Analyzer IIx. Eight of the libraries were derived from different developmental stages (from eggs to the adult stage), and the other three were from adult guts challenged by different bacteria. A total of ~291 million high-quality read pairs were obtained (Supplementary Table 1).

De novo transcript assembly

Three 454 titanium reads of expression sequence tags (ESTs) were assembled into ~90,000 non-redundant EST contigs using Newbler. Contigs shorter than 300 bp were discarded. FrameDP [80] was used to correct frameshifts and identified 52,961 contigs with exactly one protein-encoding open reading frame (ORF). These EST contigs were used to assess the completeness of the genome assembly (Supplementary Note 2).

Illumina RNA-seq data were also assembled using Trinity [81]. These data were compared with the genome-based transcript assembly and used for gene identification.

Genome-based transcript assembly

One of the state-of-the-art algorithms for genome-based transcriptome assembly is the combination of Bowtie2 [82], Tophat [83] version 2 and Cufflinks [84] version 2. However, far less than 40% of the 2x 115 bp Illumina read pairs (with unpaired and discordant alignments excluded) could be mapped to the genome using this pipeline, indicating that the pipeline is not tuned for highly polymorphic genomes.

One way to increase the successful mapping ratio is to trim the read length down to 50 bp, which led to over 70% concordant matches. However, this practice gives up virtually all advantages of the long read length.

Another way to accommodate high polymorphism rates is to relax the alignment parameters. First, we tweaked the Tophat parameters: –min-anchor-length 8; –splice-mismatches=1; –genome-read-mismatches=50; –segment-length=27; –segment-mismatches=3; and –read-mismatches=50. We then modified the Bowtie parameters: -L 18 -N 0 -i C, 1, 0 –score-min L, -0.6, -1.0 –rdg 4, 2 –rfg 4, 2 -D 20 -R 3, which increased the alignment sensitivity and allowed for low-scored alignments with more mismatches and indels. Tophat does not relay all Bowtie parameters and does not implement custom Bowtie parameters in the segment search stage; therefore, we had to work around the problem by wrapping up the executable "bowtie2-align" with a shell script that enforced the custom parameters. These tweaks significantly slowed the mapping speed by over 10-fold but did successfully map over 70% of the full-length Illumina read pairs to the genome concordantly. The alignments were fed to Cufflinks for genome-based transcript assembly and reference annotation-based transcript (RABT) assembly.

The statistics of the EST mapping against genome is shown in Supplementary Figure 23.

Supplementary Note 9 Protein-coding gene prediction and annotation

Ab initio prediction and evidence-based prediction

We aligned the 52,961 EST contigs with exactly one ORF to the haploid assembly version 2 using PASA [85] version 2011_05_20. PASA reported a total of 2883 high-quality full-length transcripts. The *ab initio* gene finders, Augustus [86] and GlimmerHMM [87], were trained on this exon set to achieve sensitivity and specificity of 78-81% and 78-81%, respectively.

The protein set of *B. floridae* was first aligned to the haploid assembly using GenBlastA [88]. GeneWise [88] version 2 was used to refine the initial protein alignments and to predict the corresponding gene structures.

The Bowtie2-Tophat-Cufflinks and GMAP/GSNAP-Cufflinks pipelines were used to create a genome-based transcript assembly for the RNA-seq dataset. Reference-based and *de novo*-assembled transcripts were incorporated into the Augustus prediction using the Augustus protocol (http://bioinf.uni-greifswald.de/bioinf/wiki/pmwiki.php?n=Augustus.IncorporateESTs).

Finally, multiple prediction sets, including PASA alignments, protein alignments, Cufflinks alignments, *ab initio* datasets from Augustus and GlimmerHMM, and RNA-seq-based predictions by Augustus, were combined into a non-redundant gene set using EVidenceModeler [85] version r03062010.

Gene model refinement using RNA-seq

We extracted the intron splice site data from the initial gene set and used them to guide a second round of RNA-seq mapping. The new mapping data and the initial gene set were combined using the Cufflinks protocol for reference annotation-based transcript (RABT) assembly. Finally, the obtained RABT assembly was fed to Augustus for a new round of evidence-based prediction. In this round of prediction, Augustus was allowed to predict evidence-based alternatively spliced isoforms.

Annotation and characterization of the predicted gene set

The refined gene set consists of 30,392 gene models. The use of a large quantity of RNA-seq data helped to predict 7,254 evidence-based alternatively spliced isoforms from 4,399 gene models. These numbers could be an overestimation because of pseudogenes, over-prediction, unrecognized transposable elements, fragmented genes and gene fragmentation at contig or scaffold boundaries. However, we also estimated that at least a thousand protein-coding genes were supported by ESTs but failed to be correctly captured by our prediction pipeline because of overlapping with the other gene models. The basic statistics are provided in Supplementary Table 13.

Gene models were annotated by homology comparison with other proteins in the InterPro

database [89]. Under the default setting, 22,008 proteins (68%) were annotated by InterProScan [90], of which 18,650 proteins (57%) were assigned at least one gene ontology (GO) term [91]. In addition, 19,537 proteins (60%) were assigned to KEGG pathways [92] (Supplementary Figure 24).

Gene models were also compared with proteins from *B. floridae* and other model organisms (*C. elegans*, *D. melanogaster*, zebrafish, chicken, mouse and human) using BLASTP. Of the 30,392 models, 27,581 models have at least one hit with an E-value of 1e-5, 26,863 models have at least one hit with an E-value of 1e-10, and 25,363 models have at least one hit with an E-value of 1e-20. Finally, up to 18,167 models have high-confidence nominal orthologs in the *B. floridae* genome.

Intron sizes and genome sizes differ between the two lancelet species

K-mer methods suggest that the genome size of *B. belcheri* is approximately 442 Mb, consistent with the range of the v18 reference haploid assembly. Therefore, the genome of *B. belcheri* is 15-20% smaller than that of *B. floridae*. However, this difference might be an artifact due to several factors. First, the k-mer method provides a coarse estimation, especially for highly polymorphic genomes. Second, the size of the haploid assembly is affected by the completeness and the precision of N-gap size estimation. Third, short read length and higher error rates may cause more repeat collapsing, hence making the assembly smaller. Finally, if allelic redundancy remains in the haploid assembly, it may inflate the assembly size.

We assumed that major size differences between the two lancelet species should lie in the intergenic regions and introns. As the intergenic regions are not easy to compare, we focused on introns. We determined that the mean length of all introns in *B. floridae* is approximately 400 bp longer than in *B. belcheri* (Supplementary Table 13), but this is still a very coarse estimation.

We next sought a strict pairwise intron comparison. We first obtained 9,961 highly reliable reciprocal best-hit (RBH) orthologous gene pairs with an identity >60% and a length coverage >60%, from which 3,976 RBH pairs were further filtered because of inconsistent exon-intron configuration. Using these orthologous gene pairs, we identified a set of high-confidence orthologous intron pairs, of which intron pairs containing N-gaps were excluded from further analysis. The comparison revealed that on average, 60% of *B. floridae* introns are longer than their corresponding introns in *B. belcheri*. This pattern is consistent in different intron positions (Supplementary Figure 25). Therefore, we concluded that in accordance with a smaller genome size, the intron size of *B. belcheri* is on average smaller than that of *B. floridae* (p<1e-16, pairwise t-test).

Supplementary Note 10 Single base-resolution methylomes of two Chinese lancelets

General design

Treatment of genomic DNA with sodium bisulfite (BS) causes massive cytosine-to-adenosine conversions, posing a considerable challenge for accurately mapping short BS-seq reads to the genome [93]. The task is even more difficult for the Chinese lancelet genome because with an average difference of 5% between the reference genome and the bisulfite-sequenced genome, short BS-seq reads simply cannot be correctly mapped to the reference genome.

To overcome this difficulty, we produced both re-sequencing data and bisulfite-sequencing data for the same lancelet individual. We first created a *de novo* assembly for the diploid genome of the selected individual. We then mapped the short BS-seq reads to this genome assembly using a wild-card bisulfite aligner (only uniquely mapped read pairs were retained) and called methylated cytosines using the default procedure of Bis-SNP [94,95]. Finally, we created a whole-genome alignment between the reference haploid genome and the re-sequenced genome assembly (Supplementary Note 5). This alignment permitted us to project the methylation patterns onto the reference genome.

To provide a biological duplicate and to reveal variation in methylation patterns between individuals, we selected two unrelated adult lancelet individuals for this study. Instead of using certain tissues and organs, we measured the average methylation level of the whole animal body.

Sample collection and whole-genome bisulfite sequencing

The two animals collected from Xiamen for re-sequencing were also used for whole-genome bisulfite sequencing. The procedures of sample collection and DNA isolation were the same as described in Supplementary Note 2, except that here we purified DNA from the whole body without gonads. The purified genomic DNA of these two animals was used for re-sequencing by the Illumina Hiseq2500 platform (2x 150 bp); approximately 45 G filtered data were generated for each animal (Supplementary Table 8). The same DNA samples were then subjected to standard whole-genome bisulfite sequencing on the Illumina Hiseq2000 platform (2x 100 bp) at the Beijing Genomics Institute (BGI; http://www.genomics.cn/en/index); approximately 20-23G filtered data were produced for each animal (Supplementary Table 8).

General methylation patterns in lancelet genomes

We obtained ~16-fold read coverage for the two individual diploid genomes (~610 Mb). We assessed the methylation levels in three sequence contexts: CG, CHG and CHH (where H is A, C or T). In both genomes, the overall genome-wide methylation levels were 21% for CG, 0.36% for CHG and 0.37% for CHH (Supplementary Table 14). The methylation level for

CGs is similar to those observed in the plant *Arabidopsis* (24%) and urochordate *Ciona intestinalis* (21.6%) [96,97]. Of ~31 million callable CG sites, ~30% have detectable methylation (i.e., passed the default Bis-SNP filtering). The level varies from 1-100% (Supplementary Figure 26). Because we used entire animal bodies for analysis, this variation suggests highly differential methylation in different cell types or even in different cells of the same type. Nevertheless, ~55% of mCGs are highly methylated (80-100%) (Supplementary Figure 26).

However, the non-CG methylation was rather weak and may represent false-positive signals. To evaluate the false-positive methylation rates, we analyzed the unmethylated mitochondrial genome. With an over 1000-fold read depth, the mitochondrial genome shows false-positive rates of 0.29-0.31% (CG), 0.32-0.33% (CHG) and 0.25-0.27% (CHH) (Supplementary Table 14). Therefore, we concluded that the observed non-CG methylation in the two lancelet genomes is too weak to be distinguishable from false-positive rates. There are two explanations for the low non-CG methylation. One is that non-CG methylation is supposed to be absent in the adult body, as observed in human fetal lung fibroblast IMR90 cells [98], the body of the pufferfish *Tetraodon nigroviridis* and the muscle tissue of the urochordate *Ciona intestinalis* [97]. Another explanation is that lancelets might lack the mechanism (i.e., CHG methyltransferase (CMT)) for non-CG methylation.

Methylation patterns in TEs and other functional regions

We analyzed the relative CG methylation level (total methylation level divided by the number of CG sites) and the absolute CG methylation level (total methylation level divided by sequence length) in different functional regions (Supplementary Figure 27).

In contrast to the genome-wide relative methylation level of 21% and the genome-wide absolute methylation level of 11%, intergenic regions showed a relative methylation level of 10% and an absolute methylation level of 5.6%, which we here considered the background methylation level in the lancelet genome.

Coding DNA sequences (CDS) displayed the highest methylation levels (33% relative and 31% absolute). This finding is consistent with observations in vertebrates and plants. It has been proposed that high methylation may prevent aberrant transcriptional initiation within gene bodies [99]. However, the methylation level of introns (~23%) was much lower than that of CDS, and introns contain many fewer CG sites (1 in 25 bp versus 1 in 10 bp in CDS and 1 in 19 bp genome-wide), which is also reflected in the even lower absolute methylation level of introns (9%). This suggests that aberrant transcription could be more frequently initiated from within introns.

The lowest methylation level was observed upstream of gene bodies (=CDS+intron), where transcriptional initiation and protein binding are known to be allowed.

Interestingly, we observed an elevated methylation level in transposable elements (TEs) that was much higher than that of intergenic regions and introns and just second to that of CDS (p<1e-16, chi-square test) (Supplementary Figure 27). This indicates that CG methylation

likely plays a role in TE suppression in lancelets. Note that this pattern is very different from the patterns observed in other invertebrates (including the urochordate *C. intestinalis* and insects), which show hypomethylation in TE regions [97].

Supplementary Note 11 Proteome size and diversity

Sizes of proteome and transcriptome

The 30,392 gene models collectively account for a total of ~48 Mb coding DNA sequences (CDS), larger than any other examined vertebrate or invertebrate genome (Supplementary Table 15). Though this number could be an overestimate of the true gene content because of pseudogenes, over-prediction, and transposable elements (as TE proteins affected 709 gene models), 27,581 and 25,363 models have homologs from other species with E-values of at least 1e-5 and 1e-20, respectively. Up to 18,167 models have nominal orthologs in the *B. floridae* genome.

To assess how many CDS are supported by ESTs, we mapped all RNA-seq data (~300 million reads or paired-end reads) to the genome using GMAP/GSNAP [40] and the default settings except counting best hits only. This analysis suggested that 70% of the genomic loci could be transcribed (i.e., were covered by at least one EST).

Because the high polymorphism and the relatively short read length (2x 115 bp) for Illumina paired-end reads may cause false alignments, to minimize false positives and obtain a lower bound, in the following statistics, we only considered those paired-end reads that could be mapped to the genome with the correct orientation and distance, or, in other words, concordantly mapped mate-pairs. The new analysis showed that 62% of the genomic loci were covered by ≥1 EST, 50% by ≥2 ESTs, and 34% by ≥5 ESTs. Furthermore, 94%, 91% and 84% of total CDS nucleotides were covered by ≥1, ≥2 and ≥5 ESTs, respectively (Supplementary Figure 23). In terms of CDS number, 95%, 92% and 86% of all CDS sequences were covered by ≥1, ≥2 and ≥5 ESTs, respectively (Supplementary Figure 23). These statistics suggest that a substantial proportion of the predicted genes could be transcribed.

We next divided the Chinese lancelet reference genome into five regions: CDS, the 2000 bp upstream of genes, the 2000 bp downstream of genes, introns and intergenic regions. We determined that 67%, 2%, 20%, 6% and 5% of the EST reads mapped to these regions, respectively (Supplementary Figure 23). Note that we assigned ESTs to a certain region following this priority: CDS, intron, downstream, upstream and then intergenic regions, which clearly biased the counts to CDS and genic regions. However, this analysis confirms the pervasive transcription of the Chinese lancelet genome. Similarly pervasive transcription has been observed in humans (~62% of the genome covered by processed mRNAs) but not in fruit flies[100,101], suggesting that pervasive transcription could be a chordate-specific feature. Compared with the much higher sequencing depth and better designs for pervasive transcription analyses in humans and fruit flies [100,101], our observation for lancelets was based on a much smaller RNA-seq dataset (~120× of the genome and ~300 million reads or read pairs restricted to a few tissues and developmental stages [Supplementary Table 1]). Because this smaller dataset covers only 50-70% of the lancelet reference genome, we speculate that lancelets might have an even more pervasive transcription pattern than humans.

Notably, the total CDS length of the draft genome of the Florida lancelet is ~8 Mb smaller but still larger than that of other invertebrates and vertebrates (except zebrafish). By searching the non-coding regions of the Florida lancelet reference genome with orthologous proteins from both lancelets, we identified an additional ~15 Mb coding sequence fragments (though some could be pseudogene fragments); therefore, we believe that the smaller proteome size of the Florida lancelet is attributable to assembly errors, under-prediction, lack of sufficient EST evidence, and the lower completeness of the draft genome of the Florida lancelet.

Pfam domain catalogs

We compared the Pfam domain catalogs of 16 species. We observed that many novel domains were only present in certain protein isoforms. Hence, for a gene with multiple protein isoforms available, all sequence isoforms were used in this study.

All deduced *B. belcheri* proteins from the gene models were compared with the Pfam database [102] using HMMER 3.0 [103]. Approximately 20,693 predicted genes have at least one detectable Pfam-A domain (transposable elements excluded). Approximately 4,383 domain types were detected in the *B. belcheri* proteome, contributing approximately 5.4 million amino acids (Supplementary Table 16). This domain type number and total sequence length are much higher than those of other known invertebrates (Supplementary Table 16). The total sequence length is also greater than all examined vertebrates (human, mouse, chicken, *Xenopus*, pufferfish) except zebrafish (Supplementary Table 16). The zebrafish has more domain sequences because it underwent an extra round of WGD during its early evolution and retains many duplicated gene copies from that WGD.

As some potential domains may not be covered by the Pfam-A dataset, we included the Pfam-B dataset for further analysis and discovered similar patterns (Supplementary Table 17). Notably, this analysis demonstrated that approximately 22,927 predicted genes have at least one detectable domain type (Pfam-A plus Pfam-B).

Ancient domain type preservation and loss

The Pfam database is biased towards vertebrates, particularly mammals, and includes many vertebrate-specific domains. To reduce this bias, we focused on ancient protein domain types, which in this study refer to the domain types present in any of the following eight invertebrates: the sea anemone *Nematostella vectensis*, the worm *C. elegans*, the insects *D. melanogaster* and *Anopheles gambiae*, the sea urchin *Strongylocentrotus purpuratus*, the oyster *C. gigas*, and the urochordates *C. intestinalis* and *C. savignyi*. We compared the occurrence of ancient domain types in amphioxus and vertebrates and determined that lancelets preserved more ancient domain types than vertebrates (at the cutoff E-values of 1 and 1e-5; Supplementary Table 18).

We also queried how many ancient domain types are preserved in lancelet species but lost in vertebrate species and *vice versa*. We first compared lancelets with the mouse and human

lineages and found ~193 ancient domain types preserved in the amphioxus lineage but lost in the mouse and human lineages. In reverse, ~112 ancient domain types were lost in the amphioxus lineage but retained in the mouse and human lineages. We then extended the comparison to six representative vertebrates, including the pufferfish, zebrafish, *Xenopus*, chicken, mouse and human. This analysis revealed that ~144 ancient domain types were preserved in the amphioxus lineage but lost in all six vertebrates. In contrast, ~122 ancient domain types were lost in amphioxus but preserved in at least one of the six vertebrates. These domain types are listed in Supplementary Table 19-20 (the default cutoff E-value was applied).

Direct assessment of protein domain diversity

Our analysis showed that the lancelet genomes contain many more Pfam-A domain types than other invertebrates (*N. vectensis*, *C. elegans*, *D. melanogaster*, *A. gambiae*, *S. purpuratus*, *C. gigas*, *C. intestinalis* and *C. savignyi*). Moreover, the lancelet genomes contain even more Pfam-A domain types than some vertebrates (*Xenopus*, chicken and *Tetraodon*) (Supplementary Table 17). We suspected that the lancelet genomes would indeed have maintained more domain types than vertebrates because according to our analysis of the Pfam-A domain dataset, there were ~460 domain types present in any of the six examined vertebrates (human, mouse, chicken, *Xenopus*, *Tetraodon* and zebrafish) but absent in the eight examined invertebrates (the sea anemone *N. vectensis*, the worm *C. elegans*, the insects *D. melanogaster* and *Anopheles gambiae*, the sea urchin *Strongylocentrotus purpuratus*, the oyster *C. gigas*, the urochordates *C. intestinalis* and *C. savignyi*, and the two lancelets). After removing these vertebrate-specific domains, we found that the lancelet genomes contained more domain types than any single vertebrate (Supplementary Table 16).

There are two major ways to give rise to a novel domain type: one is for a novel domain type to arise from unstructured protein sequence; the other is that a pre-existing domain accumulates mutations and finally becomes sufficiently divergent to form a novel domain type. The latter accounts for many vertebrate-specific domains, such as the IGV from IG types and the PYRIN domain derived from the Death/CARD/DED domains. In other words, the domain diversity in a proteome can reflect the sequence divergence.

We directly compared domain diversity between human, mouse, zebrafish, ascidians and amphioxus using Blastclust. We extracted domain sequences (all Pfam-A domain types included) and used Blastclust to cluster them. More clusters for a proteome may indicate higher diversity. To minimize artifacts caused by small domains/motifs and fragmented sequences, we used only domain types with at least 60 amino acids and required a protein sequence to cover 55% of the domain length. Two combinations of parameters were used for Blastclust: 1) –L 0.8 (min-coverage>80%) –S 50 (identity 50%) –b T (require coverage on both neighbors); and 2) –L 0.8 –S 40 –b T.

The first result (all Pfam-A domain types, i.e., both ancient domain types and vertebrate-specific domain types) shows that both lancelet species have more domain clusters than human, mouse or zebrafish (Supplementary Figure 28). Because the Pfam-A dataset is

severely biased towards vertebrates, to reduce the bias, we re-performed the clustering analysis using only ancient domain types. This new analysis gave similar results (Supplementary Figure 29). Note that we also analyzed the human+mouse dataset, which shows nearly the same number of clusters as those of human and mouse, suggesting that the clustering analysis is quite stable. For *B. floridae*, we used the diploid assembly instead of the haploid assembly because many domain types are missing in the haploid assembly for *B. floridae*. This operation nearly doubled the sequence number for *B. floridae*, but no obvious inflation occurred in the cluster number and thus further confirmed the effectiveness of this clustering analysis.

Taken together, our results suggest that lancelet genomes contain higher protein sequence diversity than those of vertebrates or invertebrates.

De novo identification of novel domains in amphioxus

We reasoned that because the Pfam domain database [104] is biased towards vertebrate proteins, the amphioxus proteome should contain many domains that have not yet been included in the Pfam database. These novel domains can be classified into two groups: one that is conserved in lancelets and other invertebrates and another that is conserved in the lancelet lineage only.

Here we attempted to glimpse of the unknown domain repertoire of amphioxus (mainly focusing on the second group). We used a *de novo* method to identify novel domains shared between the two lancelet species. We identified all Pfam-A domain sequence segments in the haploid *B. belcheri* proteome and the diploid *B. floridae* proteome using HMMER3.0 [103]. These segments were removed from the protein set. By doing so, the remaining sequences were also broken down into segments.

The protein segments from the two lancelet species were pooled together and subjected to all-against-all BLASTP with the filter on. The results were used by Blastclust to group homologous segments into clusters. The custom parameters for Blastclust were 50% identity (-S 50) and 80% coverage (-L 0.8) for both sides (-b T). We also required that an acceptable cluster should contain at least 40 amino acids and have 2 representative sequences from *B. belcheri* and 3 from *B. floridae*. This method should be effective for the two lancelets because of their divergence time of 100-130 Myr and the fact that the two species have basically no similarity in neutral sequences (Supplementary Note 3 and Supplementary Figure 5). The resulting dataset contains a total of 941 clusters, or candidate novel domain families. CLUSTALW2 was used to create multiple alignments for each cluster.

Each cluster was compared with the NCBI NR database and the Pfam database (Pfam-A+Pfam-B). Among the 941 clusters, 553 hit proteins of other species (E-value>1), 89 to Pfam-A and 213 to Pfam-B. These clusters were excluded from further analysis. In addition, we also removed clusters containing only signal peptides (detected by SignalIP 4.0) and transmembrane regions (detected by TMHMM2.0). After this filtering, we obtained a set of 375 candidate families of novel domains.

Of these 375 candidate families, 138 (with copies in 774 proteins in *B. belcheri*) were annotated as intrinsically unstructured or disordered protein sequences using IUPred [105]. This fraction (~30%) is consistent with the early observation that >30% of proteins in eukaryotic cells can be classified as intrinsically unstructured [106]. Unstructured protein sequences may function in protein-protein interactions and/or give rise to novel domains [107]. The other 237 candidate families (with copies in 1,070 proteins in *B. belcheri*) mostly co-existed with other known domains. In Supplementary Figure 30 and 31, we show the positions and related protein architectures for the 20 longest and the 10 largest domain families, respectively.

We should note that the method used here only provided a glimpse of the novel domain repertoire because one can expect that many potential novel domains fail the detection process. For example, novel domains that occur once in the genome would not be reported through this design. In addition, as a reference, in many invertebrate genomes, over 50% of domain types occur only once; in mammalian genomes, this proportion is approximately 40%.

Protein evolution and the immune and stress repertoire

Protein identity between the two lancelet species varies between different functional categories (Supplementary Table 5). The most divergent categories include extracellular components, adhesion, signaling, death and the immune system; these proteins interact with microenvironments and microorganisms and thus could be under strong diversifying/positive selection. The categories reproduction and growth were also among the most divergent. Because lancelets occupy a relatively stable ecological habitat, reproduce by mass spawning and usually live as a large population, we speculate that a major drive for protein divergence in lancelets is the intense intraspecies competition in growth rates and reproductive capability. In line with this, in the Xiamen waters, a habitat shared by Chinese and Japanese lancelets, the two species differ significantly in reproductive behavior (Supplementary Note 1). An analysis of the d_N/d_S ratios in the Chinese lancelet showed a similar trend: the highest ratios were present in extracellular components, adhesion, signaling, death and the immune system (Supplementary Table 5). Interestingly, the categories of reproduction and growth were not among the top rank of d_N/d_S ratios but rather showed higher rates in both $\mathrm{d_N}$ and $\mathrm{d_S}$ for most of the other categories, suggesting their evolution has indeed accelerated. Overall, these protein divergence patterns are basically consistent with those of vertebrates (e.g., human versus mouse) (Supplementary Table 5).

In Florida lancelets, many protein families display species-specific expansion and diversification [108,109], as also observed in Chinese lancelets. However, there are substantial differences between the two lancelets in the expansion magnitude, the proportions of orthologous pairs and the protein divergence of different protein families. Here we focused on the immune and stress repertoire that has expanded to comprise over 10% of the lancelet protein-coding genes [108,110]. Because of the limited experimental evidence and vague demarcation, immune/stress families are hereby defined by sequence similarity and may include proteins involved in apoptosis, signaling, adhesion and the extracellular matrix. While many families have similar gene numbers in the two lancelets, others, such as LRRIG,

FBG and PGRP, display different levels of expansion (Supplementary Figure 32). Moreover, some families include mostly orthologous genes, some contain mostly species-specific genes, and the others consist of half orthologous and half species-specific genes (“half-half”). At one extreme, transcription factors, kinases, and certain signal transducers and oxidative defense enzymes (e.g., NOX, GPX and PRDX) predominantly consist of orthologous genes. At the opposite extreme, TLR, NLR, SRCR, FBG and CTL contain a large proportion of species-specific genes. In vertebrates, SRCR, FBG and especially CTL proteins are implicated in many functions, such as pattern recognition, effector, stress response, adhesion and the extracellular matrix, whereas TLRs are dedicated innate receptors. In most cases, each vertebrate has exactly one ortholog for every vertebrate TLR lineage [111]. Unlike the situation in vertebrates, TLRs in lancelets include a large proportion (~85%) of species-specific genes. This contrast indicates that lancelet TLRs are neither conventional innate receptors as functionally fixed as vertebrate TLRs nor somatically diversified receptors like vertebrate BCRs/TCRs. We tentatively define lancelet TLRs as a type of “diversified innate receptor”. As for the “half-half” catalog, examples include TNF/TNFR, TIR adaptors, Death-fold domain genes and complement-related proteins (e.g., C1q, MASP and CCP). In lancelets, protein divergence is also highly variable across different families (Supplementary Figure 32). If the immune process is divided into sequential phases, the protein divergence shows a fast-narrowing trend from extracellular spaces to nuclei. Taken together, these gene expansions, diversifications, evolutionary dynamics and conservation patterns may collectively provide the necessary plasticity for host defense in the lancelet.

Supplementary Note 12 Domain combinations

We analyzed domain combinations in fifteen species, including *N. vectensis*, *C. elegans*, *D. melanogaster*, *A. gambiae*, *S. purpuratus*, *B. belcheri*, *B. floridae*, *C. savignyi*, *C. intestinalis*, *D. rerio*, *T. nigroviridis*, *X. laevis*, *G. gallus*, *M. musculus*, and *H. sapiens*. The protein set for the diploid genome of *S. purpuratus* was downloaded from NCBI. The protein set for the diploid genome of *B. floridae* (version bfv1) was retrieved from JGI (http://genome.jgi-psf.org/Brafl1/Brafl1.home.html). Protein sets for all other genomes were obtained from ENSEMBL (http://www.ensembl.org/), release 64.

Domain architectures were identified by searching the protein sequences against the Pfam database [104] using HMMER3 [103]. We observed that, especially in vertebrates, many novel domain combinations were only present in certain protein isoforms, which suggests that creating multiple alternative splice isoforms is an important way of generating novel domain combinations. Hence, for a gene with multiple protein isoforms available, all sequence isoforms were used in this study.

To suppress artifacts, we attempted to filter non-reliable hits by setting difference cutoff E-values. After multiple tests, we chose to use two values, 1 for a relaxed search and 1e-5 for a stringent search. These methods provided similar results. Any tandem array of the same domain type was compressed into one. For short domains or motifs (usually containing <20 aa), we required at least two consecutive modules or >40 aa hit length to justify the existence of the domain/motif. Signal peptides and transmembrane regions were not considered in this analysis. Finally, we took into account the domain order in a gene, which means that different orders of two adjacent domains were considered two different combinations.

Phylogenetic reconstruction based on domain combinations

At the cutoff E-values of 1 and 1e-5, we identified a total of 12,652 and 10,901 cases of two-domain combinations from the fifteen species, respectively. If the clan mode was used instead, the numbers were 8,993 and 8,271, respectively. In addition, we analyzed three- and four-domain combinations. To determine whether the gain and loss of domain combinations reflects evolution, we converted the presence and absence of the domain combinations of a species into a sequence and then used it for phylogenetic reconstruction with MEGA4 [112] and the Maximum Evolution (ME) method.

From these results, we drew several conclusions (Supplementary Figure 33). First, the gain and loss of domain combinations are largely consistent with the evolution of species. The only violation was caused by urochordates (*C. savignyi*, *C. intestinalis*), which were clustered with other protostomes due to the short branch length. The short branch length is obviously ascribed to massive gene losses in this lineage. Second, the E-value had little effect on the tree topologies. Third, the domain mode and the domain clan mode yielded similar results.

Gain and loss (or turnover) rates of domain combinations

We used the well-recognized species tree to guide the estimation of turnover rates of domain combinations along different speciation paths. The baseml (which implements the maximum likelihood method) program from PAML [113] was used for this purpose. The simplest model, JC69, was used for calculation. Two E-value settings (1 and 1e-5) and both the domain mode and the domain clan mode were attempted.

The results showed that both the vertebrate ancestor and lancelets experienced elevated turnover rates (long branches). This pattern was later slowed down in modern vertebrates. The results also showed that the turnover rate is much higher in the amphioxus lineage than in other lineages and more than twice that of the vertebrate lineage (Supplementary Figure 34). Furthermore, the difference is even larger for three- and four-domain combinations, which is mathematically expected if lancelets do have elevated domain rearrangement rates.

Novel domain combinations in lancelets

We proceeded to calculate the number of novel domain combinations specifically contained in a given lineage but not found in any other lineage. These numbers were manually counted and marked on the species trees (Supplementary Figure 35). For internal branches, we required that a novel domain combination would be counted only if it could be found in all of its directly linked subordinate branches. For example, for the branch leading to the lancelet, urochordates and vertebrates, a novel domain combination should be present in both the amphioxus branch and the vertebrate-urochordate branches. As an exception, for the branch leading to the six vertebrates, a novel domain combination had to be simultaneously present in the fish group, the mammal group and the chicken-*Xenopus* group. Note that many domain combinations could arise independently in different lineages rather than be inherited from ancestors, and these combinations should therefore be considered "novel". However, our method would exclude these combinations, leaving only unambiguous lineage-specific novel combinations.

Our results showed that in the early evolution of deuterostomes, chordates and vertebrates, there was a rapid accumulation of novel domain combinations. Both lancelets have two-fold more novel two-domain combinations (or domain pairs) than any of the six vertebrates (Supplementary Figure 35A). The difference is even more prominent for three- and four-domain combinations (Supplementary Figure 35B-C), suggesting a dramatically elevated rate of domain reshuffling in the amphioxus lineage.

We then excluded the vertebrate-specific domain types and recalculated the number of novel domain pairs (Supplementary Figure 35D-F), which resulted in a significant decrease in the vertebrate lineage. Therefore, a large proportion (33-50%) of novel domain pairs in vertebrates were considered "novel" only because of vertebrate-specific domain types.

We then focused on novel domain pairs in lancelets. The lancelet *B. belcheri* has 1,874 domain pairs never found in other examined lineages, of which 638 were shared between the

two lancelet species (Supplementary Table 21). As the divergence between lancelets and vertebrates occurred approximately 550 million years ago, and the two lancelets diverged approximately 100-130 million years ago, we inferred that the average novel domain pair gain before the divergence of the two lancelets was 638/(540-100)=1.5 per million years, and the average novel domain pair gain after the divergence of the two lancelets was (1236+1173)/(2*101)=11.9 per million years. The difference in these two rates suggests a very high turnover rates for the newly derived domain pairs, or, in other words, new domain pairs were not only gained quickly but also lost quickly.

The most used domains in novel domain pairs

We further investigated which domains are most actively involved in creating novel domain pairs, or, in other words, the most promiscuous domains in novel domain pairs. The top 50 most promiscuous domains in novel domain pairs in several important lineages are listed in Supplementary Table 22. For all examined lineages, the most promiscuous domains include EGF, Sushi, LRR, IG, Fn, Ank, TPR, and Pkinase. Different lineages also have their own favorable domains, for example, lancelets tend to use Lectin_C, Death/CARD/DED, F5_F8_type_C, and Kringle to form their novel domain pairs.

We found that the novel pairs shared between the two lancelets are 2 to 3-fold more abundant in immunity-related domains than other lineages (Supplementary Figure 36), which is consistent with previous studies [108,109]. This suggests that a large proportion of the conserved novel domain pairs in amphioxus were produced for host defense purposes. Interestingly, we also found relatively fewer immunity-related domains in those novel domain pairs restricted to one lancelet species (Supplementary Figure 36). A possible explanation is that these species-specific novel domain pairs were newly created by unbiased or less-biased selection of domain types and that natural selection has not yet had enough time to effectively reshape the composition. In addition, these patterns suggest that natural selection plays an important role in shaping the domain combination repertoire.

Among the most commonly used immunity-related domains, lancelets tend to use Lectin_C, Fibrinogen_C, LRR, Gal_lectin and Death-fold domains to create novel domain pairs. SRCR is also frequently used by the lancelet but not as frequently as the sea urchin or the deuterostome ancestor (Supplementary Figure 37). IG domains are most used by vertebrates. However, IG domains are actually the only domain type that is frequently used by all examined lineages (Supplementary Figure 37).

The top 50 promiscuous domains in novel domain pairs were then classified according to their molecular functions (Supplementary Figure 38). The two largest categories are signal transducers and receptors. In lancelets, these two domain categories are used even more frequently to create novel domain pairs (Supplementary Figure 38). In addition, relatively more catalytic domains were also used by lancelets (Supplementary Figure 38).

The top 50 promiscuous domains in novel domain pairs were next classified according to

their cellular locations (Supplementary Figure 39). This analysis revealed that amphioxus uses more extracellular domains, whereas vertebrates tend to create novel domain pairs with intracellular domains (Supplementary Figure 39).

Finally, we observed that the common ancestor of chordates, the common ancestor of deuterostomes and the amphioxus lineage used a similar set of promiscuous domains for novel domain pairs, while the vertebrate lineage used a different set. This observation became more evident after we performed a series of Pearson correlation tests on the usage patterns for the promiscuous domain sets between different lineages (Supplementary Table 23). We infer that the amphioxus lineage is more similar to the chordate and deuterostome ancestors in terms of gaining novel domain pairs.

Supplementary Note 13 Dynamic sequence shuffling

Rearrangement rates at the exon level

The excessive novel domain combinations in the lancelet genomes prompted us to wonder whether excessive rearrangements occur at the sub-genic level but failed to be reflected by the rearrangement rates calculated based on genes.

Because of the lack of independent function and regulatory elements that are usually associated with complete genes, shuffled or rearranged exons are under a very different selection regime from that of rearranged genes and may show disparate patterns.

We first analyzed the rates of exon-level rearrangements in eight species pairs, including human versus chicken, human versus rhesus (*Rhesus macaque*), pufferfish (*Tetraodon nigroviridis*) versus stickleback (*Gasterosteus aculeatus*), *C. elegans* versus *C. briggsae*, *Ciona intestinalis* versus *C. savignyi*, *Drosophila melanogaster* versus *D. mojavensis*, rat versus mouse, and *B. belcheri* versus *B. floridae*. The rate of exon rearrangements between the two haplotypes of the *B. belcheri* genome was also calculated for comparison.

All coding exon sequences, or coding DNA sequences (CDS), were extracted from each genome. BLASTN and the RBH method (described in Supplementary Note 9) were used to identify nominal orthologous CDS sequences for each species pair. Special parameters for BLASTN included “-q 2” and “-F m D”. The nominal orthologous CDS pairs for each species pair were used to calculate the number of double-cut-and-join (DCJ) rearrangement events, as described in Supplementary Note 7. To obtain a baseline for comparison, the ORF (open reading frame) sequences were also extracted and used to calculate rearrangements at the genic level.

The results are summarized in Supplementary Table 24. The relative DCJ distances estimated by ORF sequences were not unusual but rather consistent with those calculations based on protein sequences (Supplementary Table 12). On the other hand, a comparison of the two lancelet species revealed thousands of rearrangements at the exon level, many more than any other species pair (Supplementary Table 24).

To distinguish individual exon rearrangements from rearrangements involving entire genes, we subtracted the number of ORF rearrangements from the number of exon rearrangements. This calculation gave an estimate of the number of the rearrangement events that occurred at the exon level. Divided by the total number of coding exons, we obtained the relative DCJ distance contributed by exon-level rearrangements.

Supplementary Figure 40 shows that the relative DCJ distance specific to coding exons is approximately 0.1. This number is 10-100 times higher than two mammal pairs (human-rhesus and rat-mouse), and 3-20 times higher than that calculated for *C. elegans* and *C. briggsae*, which have nearly the same divergence time and global gene rearrangement rate

as the two lancelet species. The urochordate *C. intestinalis* and *C. savignyi* are well known for their rapid genome rearrangements, but the estimated exon rearrangement distance between them was still not comparable to that between lancelets (p<1e-16, chi-square test).

The relatively short length of exon sequences might cause false rearrangement events. To reduce this effect, we filtered the orthologous exon pairs by their alignment length and re-performed the analysis. The relative DCJ distances between the two lancelets were consistent under different cutoff alignment lengths, i.e., 100, 150 and 200 bp. However, for other species pairs, the distance sharply dropped to near zero when filtering was applied (Supplementary Figure 40 and Supplementary Table 24), which can be explained by two mutually compactible possibilities: a) shorter exons are more prone to false positives; and b) rearrangements of longer exons are scarce and even unfavorable in species other than lancelets.

Finally, to assess exon rearrangements at the population level, we compared the two haploid assemblies of the *B. belcheri* genome. The estimated relative DCJ distance contributed solely by exon rearrangements is 0.022-0.025, much higher than that for human versus rhesus or human versus chimpanzee (Supplementary Figure 40 and Table 24).

Global patterns of exon phase and exon expansion

Based on the phase of the flanking introns, there are nine different types of exon phases, including symmetrical 0-0, 1-1 and 2-2, and asymmetrical 0-1, 0-2, 1-0, 1-2, 2-0 and 2-1. When exon translocations (cut-and-paste or copy-and-paste), exon tandem duplications or deletions occur, exons of symmetrical phase are favored by natural selection because no immediate frame-shifts are introduced. Furthermore, early studies showed that shuffled exons encoding protein domains are significantly biased to the 1-1 phase combination in the human genome [3].

We analyzed the phase types of internal exons from eight species and found that the internal exons of lancelets are significantly biased to the 1-1 phase type. If restricted to exons encoding Pfam domains and at least 100 bp long, the 1-1 phase type accounts for over 28% of all exons, nearly twice the exon number of the 0-0 phase type and the exon number of the 1-1 phase type in human (Supplementary Figure 41). This suggests that in lancelets, natural selection favors domain exons over non-domain exons for shuffling and expansion. The logical explanation is that domain exons are more “useful” in producing proteomic diversity and plasticity (e.g., diverse domain combinations).

We then examined the composition of domain types that are significantly biased towards 1-1 phased exons (Supplementary Table 25) and 0-0 phased exons (Supplementary Table 26). Lancelets and humans share the same set of common domain types in 1-1 phased exons, suggesting that the pattern of domain type usage in 1-1 phased exons could be an ancient, conserved feature in the chordate phylum. We found that the top 10 most common symmetrical (1-1 phased) domain families involved in domain shuffling in the human genome [3] are also ranked high on the promiscuous domain list (Supplementary Table 22).

This suggests that promiscuous domains tend to disseminate via 1-1 phase exons. These families include EGF, Sushi, CUB, VWA, VWC, F5_F8_type_C, PAN_1, MAM, TIG, Kringle and Ldl_recept_a. Notably, nearly all internal IG domains from both vertebrates and lancelets are encoded in 1-1 phased exons, suggesting that the large expansion of IG domains in chordates (especially in vertebrates) is mainly disseminated through 1-1 phased exons.

The 1-1 phased exons appear more active in expansion than the 0-0 phased exons because both their absolute numbers and ratios of 1-1 versus 0-0 phase are higher (Supplementary Table 25-26). Some domain families (e.g., EGF, Sushi, IG, fn3, CUB, VWA, PAN_1, IG, Ldl_recept_a, etc.) are expanded in both vertebrates and lancelets, though the expansion is generally more intense in lancelets. However, other families are specifically expanded in amphioxus, including Lectin_C, Fibrinogen_C, Gal_lectin, SRCR, Death-fold domains, TSP_1, Kringle, WSC, TIL, F5_F8_type_C, PKD_channel, Mucin2, Glycos_transf_1, Methyltransf_FA, and GCC2_GCC3. All of these domains are actively involved in novel domain combinations and are listed in Supplementary Table 22.

In addition, there are several 0-0 phase-bound expanded domains active in forming novel domain pairs, including Ank, Pkinase, Pkinase_Tyr (weak), WD40, BTB, and Ras (Supplementary Table 22 and 26).

Finally, it is worth noting that several domain families active in novel domain pairs show no apparent bias in phase types, including LRR, 7tm_1 and P450.

Analysis of exon-level shuffling events between lancelet species

We chose the chainNet method to find confident genomic rearrangements. This method was previously used to identify genomic rearrangements between the mouse and human genomes [43].

The two lancelet haploid genome sequences were first repeat-masked, and then LASTZ was used to create whole-genome DNA alignments. LASTZ was tuned to the high-sensitivity mode with the following special parameter settings: –masking=0, –hspthresh=3000, –ydrop=3400, –gappedthresh=3000, –gap=400,30, –step=1, –seed=12of19, –identity=75, and the score matrix "100 -225 -225 -225; -225 100 -225 -225; -225 -225 100 -225; -225 -225 -225 100". The LASTZ alignments were processed into reciprocal-best single-coverage chainNet alignments according to UCSC's documentation. Special parameters for axtChain and chainNet include –linearGap=loose, –minScore=2000 and –minSpace=50.

Unlike the RBH method, which intends to find the best hit between individual exon or gene sequences, the whole-genome chainNet method takes into account both non-exon sequences and syntenic information. Hence, the chainNet method generally reports fewer but higher-confidence rearrangements. In addition, the chainNet method is not affected by errors in gene and domain annotations that can occur in draft genomes.

We did not distinguish between cut-and-paste and copy-and-paste mechanisms because both

can create novel gene structures and novel domain combinations.

From the pairwise alignments between the two lancelet genomes, we detected 6,782 translocation events (inversions excluded) 100-50,000 bp in length, of which 3,097 events contained coding exons and 1,047 contained domain-encoding exons (Supplementary Table 27). The 3,097 translocations harbor a total of 14,280 exons, of which 10,592 are middle exons and biased towards the 1-1 phase (with 21.0% exons as 1-1 phase versus 18.5% as 0-0 phase). The bias is even stronger for domain-encoding middle exons, with 31.0% exons as 1-1 phase and 17.7% as 0-0 phase.

It is difficult to separate exon shuffling from gene shuffling that happened between two species, especially when the gene-based DCJ distance is as high as 0.23 (Supplementary Table 12). However, because there should be no mechanistic boundary between the two types of shuffling except for sequence length, we posited that the smaller the translocation size, the more likely it is an exon-level shuffling event. We thus identified all translocation events that contain ≤10 exons and filtered for exons at least 100 bp long with 80% alignment coverage. We then compared the extent of exon phase bias between different translocation sizes (Supplementary Table 28). These results showed that the smaller the translocation size, the higher the phase bias towards 1-1. For translocations containing single domain-encoding exons, 50% of the exons showed the 1-1 phase combination. Supplementary Table 29 lists the common domain types encoded in these translocations, which are basically the same set of domains actively involved in novel domain pairs (i.e., promiscuous domains; Supplementary Table 22).

Analysis of exon-level shuffling events within the *B. belcheri* diploid genome

The same chainNet method and parameters were used to create reciprocal-best single-coverage chainNet alignments for the two haploid sequences of the *B. belcheri* diploid genome (the v18 reference assembly versus the v18 alternative assembly). From the alignments, we identified 6,244 translocation events (inversions excluded), of which 5,713 were within the range of 100-50,000 bp (Supplementary Table 27). Among the 5,713 translocation events, 1,056 events (18.5%) contained coding exons, and 293 (5.0%) contained domain-encoding coding exons.

For comparison, we also identified translocations by applying the same method to two species pairs, human versus rhesus and human versus chimpanzee (Supplementary Table 27).

Human and rhesus have a genomic sequence divergence of ~5%, close to that between the two *B. belcheri* haploid sequences. The number of translocation events (4,981) between human and rhesus is close to that of the lancelet diploid genome, though they have ~10-fold more total alignable sequences than the lancelet alleles. Moreover, only 310 of the 4,981 translocations contain coding exons, and only 173 contain domain-encoding exons (Supplementary Table 27). These statistics are even smaller between human and chimpanzee, likely due to their more recent sequence divergence (Supplementary Table 27). Based on the statistics presented in Supplementary Table 27, we estimate that the potential exon shuffling

rate is over 10 times higher in lancelets compared with humans.

We identified all exons involved in these translocation events and compared their phase bias and composition (Supplementary Table 28 and 29). Shuffled exons in the *B. belcheri* genome are clearly biased to the 1-1 phase combination, whereas those from the human genome show no such bias.

Association of transposases/retrotranscriptases and micro-translocations

We also looked into the relationship between TEs and micro-translocations in the Chinese lancelet genome.

Using RPS-BLAST and Pfam domains for transposases and retrotranscriptases (55% coverage and an E-value of <1e-5), we identified 415 transposase gene fragments and 2,300 retrotranscriptase gene fragments in the *B. belcheri* genome. Using the same method, we identified 2,926, 2,861 and 2,416 transposase gene fragments and 20,883, 18,845 and 16,164 retrotranscriptase fragments in the human, chimpanzee and rhesus genomes, respectively.

We next assessed whether these TE fragments co-localized with the identified micro-translocations (plus the upstream and downstream 1,000 bp). We found that in the *B. belcheri* genome, transposases and retrotranscriptases were significantly enriched around micro-translocations (Chi-square test, p<1e-16), whereas such enrichment was not observed between human and rhesus or human and chimpanzee (Supplementary Table 30). This suggests that in the *B. belcheri* genome, TE activity might play a role in driving micro-translocations.

It has been suggested that in vertebrates, gene fusion is much more important than transposition for domain gains [114]. However, in the Chinese lancelet genome, we observed a high enrichment of transposase (12%) and retrotranscriptase (16%) gene fragments in the translocation regions – 10-fold higher than the enrichment in the translocation regions between human versus rhesus (Chi-square test, p<1e-16) (Supplementary Table 30). This is further evidence that TEs might have a more important role in micro-translocations in lancelets.

Supplementary Note 14 Conserved non-coding elements

Methods to identify conserved non-coding elements

We used the reciprocal-best whole-genome alignment method to identify conserved non-coding elements (CNEs) between two genome sequences. The aforementioned LASTZ-chainNet method [43] was used for this task. Unlike the reciprocal-best BLAST method, the reciprocal-best LASTZ-chainNet method takes synteny into account and permits translocations and inversions, which is conservative but increases the search sensitivity.

The two genome sequences were first repeat-masked, and then LASTZ was used to create whole-genome DNA alignments. LASTZ was tuned to the high sensitivity mode with the following special parameter settings: –masking=0, –hspthresh=3000, –ydrop=9400, –gappedthresh=3000, –gap=400,30, –step=1, –seed=12of19, –identity=80, and the score matrix “100 -300 -300 -300; -300 100 -300 -300; -300 -300 100 -300; -300 -300 -300 100”. The LASTZ alignments were processed into reciprocal-best single-coverage chainNet alignments according to UCSC’s documentation. Special parameters for axtChain and chainNet included –linearGap=loose, –minScore=1000 and –minSpace=20. These settings maintain high search sensitivity but suppress low identity alignments (only alignments with 80%identity are considered). A set of relaxed parameters were also used for comparison, using the scoring matrix “100 -200 -200 -200; -200 100 -200 -200; -200 -200 100 -200; -200 -200 -200 100”.

Clearly, the method used herein does not distinguish between different types of conserved elements, including coding regions, pseudogenes, cis-regulatory elements, microRNAs, long non-coding RNAs, some transposable elements, etc. To exclude snoRNAs, snRNAs, tRNAs, rRNAs, scRNAs, snlRNAs, coding exons, repetitive sequences and the regions flanking these sequences, we consulted the annotation file (the gtf/gff3 files corresponding to the genome sequences) and compared the sequences to known proteins, protein-coding transcripts, and tRNA/rRNA/snoRNA/scRNA/snlRNA libraries.

The obtained CNEs contained cis-regulatory elements, microRNAs and long non-coding RNAs. We did not distinguish between these categories in this study. These three types of CNEs control the timing, quantity, and regions of gene expression as well as post-transcriptional regulation.

We identified CNEs between Chinese and Florida lancelets. For comparison, we performed parallel searches for CNEs in other four species pairs: human versus mouse, human versus opossum, the worms *C. elegans* versus *C. briggsae*, and the insects *D. melanogaster* versus *D. mojavensis*.

Identification of CNE candidates from five species pairs

Using the method described above, we found that non-repeating, non-CDS, conserved, and

reciprocal-best alignments comprised 11% (45.4 Mb) of the lancelet genome, 3% (3 Mb) of the *C. elegans* genome, 4% (6.7 Mb) of the *D. melanogaster* genome, and 3.4% (106 Mb; versus mouse) or 1.1% (33.5 Mb; versus opossum) of the human genome (Supplementary Table 31A). The relative lack of conserved non-coding regions in worms and fruit flies agrees with previous reports. However, it was surprising to observe that the lancelet genome contains such an abundance of CNE contents, not only in terms of relative abundance (11% of the genome) but also in the absolute amount (45.4 Mb) – even more than the amount (33.5 Mb) in the human genome, as identified by comparison with the opossum genome. Note that the human genome is six times larger than that of the lancelet. For the record, the divergence of the two lancelets (100-130 Myr) falls between the divergence of human versus mouse (62-100 Myr) and human versus opossum (125-138 Myr) (see Supplementary Note 3). A similar trend was obtained using a less stringent alignment scheme (Supplementary Table 31B).

We then refined the CNE candidate sets by removing sequences shorter than 75 bp, with a sequence identity lower than 70%, adjacency to coding sequences, or blast hits to proteins or rRNAs/tRNAs/snoRNAs, etc. (Supplementary Table 32).

We first removed a large fraction (15%; 6.8 Mb) of sequences (<75 bp) from the lancelet CNE candidates. It appears that short sequences account for a larger proportion of the total candidate CNE contents in non-vertebrates (lancelets, worms and fruit flies) than in humans (Supplementary Table 32). Nevertheless, because short sequences are more prone to false positives, we removed them from the list of CNE candidates.

We then removed another fraction (14%; 6.2 Mb) of the lancelet CNE candidates because they were adjacent to coding regions and could be 5/3'-UTRs, unidentified alternative spliced exons or unpredicted exon parts (Supplementary Table 32). Similar numbers of CDS-adjacent CNE candidates were found in the human genome (7 Mb versus mouse or 1.7 Mb versus opossum) and turned out to be mostly 5/3'-UTRs. For the record, because we could not correctly predict the protein-coding gene-related non-coding exons (which are mostly 5/3'-UTRs) in the lancelet genome, to achieve a fair comparison, we did not distinguish between conserved non-coding exons and other CNEs. As a result, the human CNE candidates include a large proportion of protein-coding gene-related non-coding exons (~9.7 Mb for human versus mouse and ~3.7 Mb for human versus opossum).

We also removed CNE candidates with blast hits (1e-5) to known proteins or tRNAs/rRNAs/snoRNAs/scRNAs/snlRNAs (Supplementary Table 32). This caused the removal of 2.27 Mb of conserved protein-coding sequences from the lancelet CNE candidates. This amount is 10 times higher than the corresponding amounts in the human (versus opossum), worm and fruit fly genomes. This result implies that a large proportion of conserved coding regions in the lancelet genome could remain unpredicted, confirming the observation that lancelets have an enormous proteome (Supplementary Note 11).

Estimation of CNE search sensitivity using known microRNA genes

An early study identified 113 microRNA genes in lancelets [4]. Here we used these genes to evaluate the sensitivity of our CNE detection methods. The v18 reference assembly of the Chinese lancelet genome contains 110 of the 113 microRNA genes. In the refined CNE candidate dataset (Supplementary Table 32), we recovered 103. Among the seven missing genes, three are not present in the reference genome of the Florida lancelet. Therefore, our method recovered 103/107=96% of the microRNA genes.

These microRNAs are listed in Supplementary Table 33. For the annotation and precursor sequences of these microRNA genes, the reader is referred to the work of Chen et al. [4].

Analysis of lancelet CNE candidates

The final CNE dataset (Supplementary Table 32) recapitulates the discovery from the initial dataset (Supplementary Table 32): lancelet genomes are surprisingly abundant in CNEs, even more than those conserved between human and opossum.

The average length of the lancelet CNEs is similar to that of humans (220-230 bp) but twice that of invertebrates (p<1e-16, t-test) (Supplementary Table 32), suggesting that lancelet and human CNEs might share more compositional properties.

Given their important roles in gene regulation, CNEs are often associated with protein-coding genes of certain functional categories. We used the GO functional classification system to analyze the functional categories of genes that tend to be associated with lancelet CNEs. We found that lancelet CNEs tend to be enriched around genes associated with certain cell compartments (synapse, cell junction and extracellular) and with certain molecular functions (biological adhesion, locomotion, developmental process and signaling). This preference is similar to those of humans, worms and fruit flies; therefore, despite their abundance, lancelet CNEs exhibit ordinary functional patterns.

We next compared the sequence identity distributions of CNEs from five species pairs (Supplementary Figure 42). In theory, one may expect that as two species diverge further (either due to longer separation times or elevated evolutionary rates), the more important and more functionally constrained CNEs will tend to be retained; on the other hand, for less-diverged species, neutral or nearly neutral sites may still maintain a weak sequence similarity, hence resulting in a larger fraction of low-identity alignments. We found that the CNE repertoire for human and mouse contains the largest proportion of low-identity sequences, whereas the CNE repertoire between the two fruit flies contains the largest fraction of high-identity sequences. This observation is consistent with the protein sequence divergence rates (Supplementary Note 3). Remarkably, the sequence identity distribution of the lancelet CNEs falls between those of human versus mouse and human versus opossum (Supplementary Figure 42-43), perfectly recapitulating their protein divergence patterns (Supplementary Figure 6; Supplementary Note 3). This once again confirms that the divergence of Chinese and Florida lancelets occurred between those of human versus mouse

and human versus opossum. Our findings also suggest that the CNEs detected for human versus mouse represent an overestimate relative to the two lancelet genomes.

Identification of CNE-enriched regions in lancelets

We proceeded to identify the regions with high densities of CNEs within the lancelet genomes. We used a sliding window of 20 genes with a step size of 1 gene to search for CNE-enriched regions. The windows were ranked by their CNE density, and the top 5% windows were considered CNE-enriched regions.

This method revealed 30 CNE-enriched regions (Supplementary Table 34). These regions cover a total of 1040 (3%) protein-coding gene models, extend over 22.5 Mb (5% of the genome) and contain 18,697 CNEs (16% of all CNEs). A GO analysis of these protein-coding genes revealed no special functional preference (though development-related genes were slightly elevated by 5%, and metabolism-related genes were reduced by 5%). One of these regions harbors a set of genes involved in acute epithelial immune responses (LBP, BPI, TLR and the histamine receptor). Another region contains the HOX gene cluster, which was previously reported to contain a high density of CNEs [115].

Identification of CNEs shared between lancelets and humans

The CNEs from human versus opossum and the two lancelets (Supplementary Table 32) were compared to identify those CNEs conserved across subphyla. NCBI-BLASTN was used for this comparison, with the special parameters “-q -1, -F T -e 1e-2” and a minimum sequence identity of 60%.

We found that despite the great abundance of CNEs in both lancelets and humans, very few CNE motifs are shared between them.

If we required a minimum alignment length of 45 bp for the shared CNEs (as used in a previous study [116]), only 1,086 (or 704 if a reciprocal-best hit was required) lancelet CNEs had homologs in the human CNE repertoire. This number is far below 1% of the total number of CNEs in lancelets or humans (Supplementary Table 32). However, this number is two times higher than that (432) identified between lancelets and mice using a different method [116].

If we lowered the minimum alignment length to 30 bp, the resulting shared CNE number was 3,553 (or 2,029 if a reciprocal-best hit was required).

We then used GO analysis to evaluate whether these highly conserved CNEs were preferably associated with certain protein-coding genes (Supplementary Table 35). We found that these CNEs were significantly enriched in the vicinity of protein-coding genes involved in biological adhesion, signaling, multicellular organismal process, developmental process, locomotion, regulation of biological process, cellular component organization or biogenesis, cell junction, synapse, protein binding transcription factor activity and nucleic acid binding

transcription factor activity (Supplementary Table 35). In general, the enrichment appeared higher in the human genome than in the lancelet genome, though we could not rule out the possibility that this observation is because the BLAST-based GO annotation for lancelet genes is less reliable than that for human genes.

Supplementary references

1 Putnam, N. H. *et al.* The amphioxus genome and the evolution of the chordate karyotype. *Nature* **453**, 1064-1071 (2008).

2 Sodergren, E. *et al.* The genome of the sea urchin Strongylocentrotus purpuratus. *Science* **314**, 941-952 (2006).

3 Kaessmann, H., Zollner, S., Nekrutenko, A. & Li, W. H. Signatures of domain shuffling in the human genome. *Genome Res* **12**, 1642-1650 (2002).

4 Chen, X. *et al.* Identification and characterization of novel amphioxus microRNAs by Solexa sequencing. *Genome Biol* **10**, R78 (2009).

5 Delsuc, F., Brinkmann, H., Chourrout, D. & Philippe, H. Tunicates and not cephalochordates are the closest living relatives of vertebrates. *Nature* **439**, 965-968 (2006).

6 Nishikawa, T. A new deep-water lancelet (Cephalochordata) from off Cape Nomamisaki, SW Japan, with a proposal of the revised system recovering the genus Asymmetron. *Zoolog Sci* **21**, 1131-1136 (2004).

7 Poss, S. & Boschung, H. Lancelets (Cephalochordata: Braanchiostomatidae): how many species are valid? *Israel Journal of Zoology* **42**, S-13-S-66 (1996).

8 Light, S. F. Amphioxus Fisheries near the University of Amoy, China. *Science* **58**, 57-60 (1923).

9 Boring, A. & Li, H. Is the Chinese amphioxus a separate species? *Peking Natural History Bulletin* **6**, 9-18 (1932).

10 Zhang, Q. J., Zhong, J., Fang, S. H. & Wang, Y. Q. Branchiostoma japonicum and B. belcheri are distinct lancelets (Cephalochordata) in Xiamen waters in China. *Zoolog Sci* **23**, 573-579 (2006).

11 Chen, Y., Cheung, S., Kong, R. & Shin, P. Morphological and molecular comparisons of dominant amphioxus populations in the China Seas. *Mar Biol* **153**, 189-198 (2007).

12 Xiao, Y., Zhang, Y., Gao, T., Yabe, M. & Sakurai, Y. Phylogenetic relationships of the lancelets of the genus Branchiostoma in China inferred from mitochondrial genome analysis. *African Journal of Biotechnology* **7**, 3845-3852 (2008).

13 Xu, Q., Ma, F. & Wang, Y. Morphological and 12S rRNA Gene Comparison of Two Branchiostoma Species in Xiamen Waters. *J Exp Zoolog B Mol Dev Evol* **304**, 259-267 (2005).

14 Wang, Y., Xu, Q., Peng, X. & Zhou, H. Taxonomic status of amphioxus Branchiostoma belcheri in Xiamen Beach estmated by homologous sequence of Cyt b gene. *Acta Zool Sinica* **50**, 60-66 (2005).

15 Zhong, J. *et al.* Complete mitochondrial genomes defining two distinct lancelet species in the West Pacific Ocean. *Mar Biol Res* **5**, 278-285 (2009).

16 Zhang, Q., Guang, L., Yi, S. & Wang, Y. [Chromosome Preparation and Preliminary Observation of Two Amphioxus Species in Xiamen]. *Zoolog Res (in Chinese)* **30**, 131-136 (2009).

17 Nohara, M., Nishida, M., Manthacitra, V. & Nishikawa, T. Ancient phylogenetic separation between Pacific and Atlantic cephalochordates as revealed by mitochondrial genome analysis. *Zoolog Sci* **21**, 203-210 (2004).

18 Zhang, Q. J. *et al.* Continuous culture of two lancelets and production of the second filial generations in the laboratory *Journal of experimental zoology* **308**, 464-472 (2007).

19 Li, G., Shu, Z. & Wang, Y. Year-round reproduction and induced spawning of Chinese amphioxus, Branchiostoma belcheri, in laboratory. *PLoS One* **8**, e75461 (2013).

20 Li, G., Yang, X., Shu, Z., Chen, X. & Wang, Y. Consecutive spawnings of Chinese amphioxus,

Branchiostoma belcheri, in captivity. *PLoS One* **7**, e50838 (2012).
21 Li, G. *et al.* Mutagenesis at specific genomic loci of amphioxus Branchiostoma belcheri using TALEN method. *Journal of genetics and genomics = Yi chuan xue bao* **41**, 215-219 (2014).
22 Star, B. *et al.* The genome sequence of Atlantic cod reveals a unique immune system. *Nature* **477**, 207-210 (2011).
23 Li, R. *et al.* The sequence and de novo assembly of the giant panda genome. *Nature* **463**, 311-317 (2010).
24 Wheeler, D. A. *et al.* The complete genome of an individual by massively parallel DNA sequencing. *Nature* **452**, 872-876 (2008).
25 Jones, T. *et al.* The diploid genome sequence of Candida albicans. *Proc Natl Acad Sci U S A* **101**, 7329-7334 (2004).
26 Holt, R. A. *et al.* The genome sequence of the malaria mosquito Anopheles gambiae. *Science* **298**, 129-149 (2002).
27 Dehal, P. *et al.* The draft genome of Ciona intestinalis: insights into chordate and vertebrate origins. *Science* **298**, 2157-2167 (2002).
28 Aparicio, S. *et al.* Whole-genome shotgun assembly and analysis of the genome of Fugu rubripes. *Science* **297**, 1301-1310 (2002).
29 Vinson, J. P. *et al.* Assembly of polymorphic genomes: algorithms and application to Ciona savignyi. *Genome Res* **15**, 1127-1135 (2005).
30 Pop, M. Genome assembly reborn: recent computational challenges. *Brief Bioinform* **10**, 354-366 (2009).
31 Huang, S. *et al.* HaploMerger: reconstructing allelic relationships for polymorphic diploid genome assemblies. *Genome Res* **22**, 1581-1588 (2012).
32 Lin, Y. *et al.* Comparative studies of de novo assembly tools for next-generation sequencing technologies. *Bioinformatics* **27**, 2031-2037 (2011).
33 Zhang, G. *et al.* The oyster genome reveals stress adaptation and complexity of shell formation. *Nature* **490**, 49-54 (2012).
34 Margulies, M. *et al.* Genome sequencing in microfabricated high-density picolitre reactors. *Nature* **437**, 376-380 (2005).
35 Miller, J. R. *et al.* Aggressive assembly of pyrosequencing reads with mates. *Bioinformatics* **24**, 2818-2824 (2008).
36 Li, R. *et al.* De novo assembly of human genomes with massively parallel short read sequencing. *Genome Res* **20**, 265-272 (2010).
37 Small, K. S., Brudno, M., Hill, M. M. & Sidow, A. A haplome alignment and reference sequence of the highly polymorphic Ciona savignyi genome. *Genome Biol* **8**, R41 (2007).
38 Pop, M., Kosack, D. S. & Salzberg, S. L. Hierarchical scaffolding with Bambus. *Genome Res* **14**, 149-159 (2004).
39 Morgulis, A., Gertz, E. M., Schaffer, A. A. & Agarwala, R. WindowMasker: window-based masker for sequenced genomes. *Bioinformatics* **22**, 134-141 (2006).
40 Wu, T. D. & Watanabe, C. K. GMAP: a genomic mapping and alignment program for mRNA and EST sequences. *Bioinformatics* **21**, 1859-1875 (2005).
41 Kelley, D. R., Schatz, M. C. & Salzberg, S. L. Quake: quality-aware detection and correction of sequencing errors. *Genome Biol* **11**, R116 (2010).
42 Schwartz, S. *et al.* Human-mouse alignments with BLASTZ. *Genome Res* **13**, 103-107 (2003).

43 Kent, W. J., Baertsch, R., Hinrichs, A., Miller, W. & Haussler, D. Evolution's cauldron: duplication, deletion, and rearrangement in the mouse and human genomes. *Proc Natl Acad Sci U S A* **100**, 11484-11489 (2003).

44 Wu, T. D. & Nacu, S. Fast and SNP-tolerant detection of complex variants and splicing in short reads. *Bioinformatics* **26**, 873-881 (2010).

45 Cameron, M., Williams, H. E. & Cannane, A. Improved gapped alignment in BLAST. *IEEE/ACM Trans Comput Biol Bioinform* **1**, 116-129 (2004).

46 Thompson, J. D., Gibson, T. J. & Higgins, D. G. Multiple sequence alignment using ClustalW and ClustalX. *Curr Protoc Bioinformatics* **Chapter 2**, Unit 2 3 (2002).

47 Castresana, J. Selection of conserved blocks from multiple alignments for their use in phylogenetic analysis. *Mol Biol Evol* **17**, 540-552 (2000).

48 Lartillot, N. & Philippe, H. A Bayesian mixture model for across-site heterogeneities in the amino-acid replacement process. *Mol Biol Evol* **21**, 1095-1109 (2004).

49 Darriba, D., Taboada, G. L., Doallo, R. & Posada, D. ProtTest 3: fast selection of best-fit models of protein evolution. *Bioinformatics* **27**, 1164-1165 (2011).

50 Le, S. Q. & Gascuel, O. An improved general amino acid replacement matrix. *Mol Biol Evol* **25**, 1307-1320 (2008).

51 Guindon, S. & Gascuel, O. A simple, fast, and accurate algorithm to estimate large phylogenies by maximum likelihood. *Syst Biol* **52**, 696-704 (2003).

52 Benton, M. J. & Donoghue, P. C. Paleontological evidence to date the tree of life. *Mol Biol Evol* **24**, 26-53 (2007).

53 Chen, J. Y. *et al.* The first tunicate from the Early Cambrian of South China. *Proc Natl Acad Sci U S A* **100**, 8314-8318 (2003).

54 Butterfield, N. J. Hooking some stem-group "worms": fossil lophotrochozoans in the Burgess Shale. *Bioessays* **28**, 1161-1166 (2006).

55 Guindon, S. Bayesian estimation of divergence times from large sequence alignments. *Mol Biol Evol* **27**, 1768-1781 (2010).

56 Nohara, M., Nishida, M. & Nishikawa, T. New complete mitochondrial DNA sequence of the lancelet Branchiostoma lanceolatum (Cephalochordata) and the identity of this species' sequences. *Zoolog Sci* **22**, 671-674 (2005).

57 J, G. Plate-Tectonic Maps of the Phanerozoic. *Phanerozoic Reef Patterns. SEPM Special Publication* **72**, 21-75 (2002).

58 Edgar, R. C. MUSCLE: multiple sequence alignment with high accuracy and high throughput. *Nucleic Acids Res* **32**, 1792-1797 (2004).

59 Waterston, R. H. *et al.* Initial sequencing and comparative analysis of the mouse genome. *Nature* **420**, 520-562 (2002).

60 Fan, Y. *et al.* Patterns of insertion and deletion in Mammalian genomes. *Curr Genomics* **8**, 370-378 (2007).

61 Small, K. S., Brudno, M., Hill, M. M. & Sidow, A. Extreme genomic variation in a natural population. *Proc Natl Acad Sci U S A* **104**, 5698-5703 (2007).

62 Guryev, V. *et al.* Genetic variation in the zebrafish. *Genome Res* **16**, 491-497 (2006).

63 Fay, J. C., Wyckoff, G. J. & Wu, C. I. Testing the neutral theory of molecular evolution with genomic data from Drosophila. *Nature* **415**, 1024-1026 (2002).

64 Wang, J. *et al.* The diploid genome sequence of an Asian individual. *Nature* **456**, 60-65 (2008).

65 Blanchette, M. *et al.* Aligning multiple genomic sequences with the threaded blockset aligner. *Genome Res* **14**, 708-715 (2004).

66 Huang, S., Li, J., Xu, A., Huang, G. & You, L. Small insertions are more deleterious than small deletions in human genomes. *Hum Mutat* **34**, 1642-1649 (2013).

67 Flutre, T., Duprat, E., Feuillet, C. & Quesneville, H. Considering transposable element diversification in de novo annotation approaches. *PLoS ONE* **6**, e16526 (2011).

68 Bao, Z. & Eddy, S. R. Automated de novo identification of repeat sequence families in sequenced genomes. *Genome Res* **12**, 1269-1276 (2002).

69 Price, A. L., Jones, N. C. & Pevzner, P. A. De novo identification of repeat families in large genomes. *Bioinformatics* **21 Suppl 1**, i351-358 (2005).

70 Benson, G. Tandem repeats finder: a program to analyze DNA sequences. *Nucleic Acids Res* **27**, 573-580 (1999).

71 Jurka, J. *et al.* Repbase Update, a database of eukaryotic repetitive elements. *Cytogenet Genome Res* **110**, 462-467 (2005).

72 Tarailo-Graovac, M. & Chen, N. Using RepeatMasker to identify repetitive elements in genomic sequences. *Curr Protoc Bioinformatics* **Chapter 4**, Unit 4 10 (2009).

73 Canestro, C. & Albalat, R. Transposon diversity is higher in amphioxus than in vertebrates: functional and evolutionary inferences. *Brief Funct Genomics* **11**, 131-141 (2012).

74 Yancopoulos, S., Attie, O. & Friedberg, R. Efficient sorting of genomic permutations by translocation, inversion and block interchange. *Bioinformatics* **21**, 3340-3346 (2005).

75 Chen, Z., Huang, S., Li, Y. & Xu, A. AliquotG: an improved heuristic algorithm for genome aliquoting. *PLoS One* **8**, e64279 (2013).

76 Warren, R. & Sankoff, D. Genome aliquoting with double cut and join. *BMC Bioinformatics* **10 Suppl 1**, S2 (2009).

77 Vienne, A. *et al.* Evolution of the proto-MHC ancestral region: more evidence for the plesiomorphic organisation of human chromosome 9q34 region. *Immunogenetics* **55**, 429-436 (2003).

78 Abi-Rached, L., Gilles, A., Shiina, T., Pontarotti, P. & Inoko, H. Evidence of en bloc duplication in vertebrate genomes. *Nat Genet* **31**, 100-105 (2002).

79 Abi Rached, L., McDermott, M. F. & Pontarotti, P. The MHC big bang. *Immunol Rev* **167**, 33-44 (1999).

80 Gouzy, J., Carrere, S. & Schiex, T. FrameDP: sensitive peptide detection on noisy matured sequences. *Bioinformatics* **25**, 670-671 (2009).

81 Grabherr, M. G. *et al.* Full-length transcriptome assembly from RNA-Seq data without a reference genome. *Nat Biotechnol* **29**, 644-652 (2011).

82 Langmead, B. & Salzberg, S. L. Fast gapped-read alignment with Bowtie 2. *Nat Methods* **9**, 357-359 (2012).

83 Trapnell, C., Pachter, L. & Salzberg, S. L. TopHat: discovering splice junctions with RNA-Seq. *Bioinformatics* **25**, 1105-1111 (2009).

84 Trapnell, C. *et al.* Transcript assembly and quantification by RNA-Seq reveals unannotated transcripts and isoform switching during cell differentiation. *Nat Biotechnol* **28**, 511-515 (2010).

85 Haas, B. J. *et al.* Automated eukaryotic gene structure annotation using EVidenceModeler and the Program to Assemble Spliced Alignments. *Genome Biol* **9**, R7 (2008).

86 Stanke, M., Diekhans, M., Baertsch, R. & Haussler, D. Using native and syntenically mapped cDNA alignments to improve de novo gene finding. *Bioinformatics* **24**, 637-644 (2008).

87 Majoros, W. H., Pertea, M. & Salzberg, S. L. TigrScan and GlimmerHMM: two open source ab initio eukaryotic gene-finders. *Bioinformatics* **20**, 2878-2879 (2004).

88 She, R., Chu, J. S., Wang, K., Pei, J. & Chen, N. GenBlastA: enabling BLAST to identify homologous gene sequences. *Genome Res* **19**, 143-149 (2009).

89 Hunter, S. *et al.* InterPro in 2011: new developments in the family and domain prediction database. *Nucleic Acids Res* **40**, D306-312 (2012).

90 Quevillon, E. *et al.* InterProScan: protein domains identifier. *Nucleic Acids Res* **33**, W116-120 (2005).

91 Ashburner, M. *et al.* Gene ontology: tool for the unification of biology. The Gene Ontology Consortium. *Nat Genet* **25**, 25-29 (2000).

92 Kanehisa, M. & Goto, S. KEGG: kyoto encyclopedia of genes and genomes. *Nucleic Acids Res* **28**, 27-30 (2000).

93 Krueger, F., Kreck, B., Franke, A. & Andrews, S. R. DNA methylome analysis using short bisulfite sequencing data. *Nat Methods* **9**, 145-151 (2012).

94 Bock, C. Analysing and interpreting DNA methylation data. *Nat Rev Genet* **13**, 705-719 (2012).

95 Liu, Y., Siegmund, K. D., Laird, P. W. & Berman, B. P. Bis-SNP: Combined DNA methylation and SNP calling for Bisulfite-seq data. *Genome Biol* **13**, R61 (2012).

96 Cokus, S. J. *et al.* Shotgun bisulphite sequencing of the Arabidopsis genome reveals DNA methylation patterning. *Nature* **452**, 215-219 (2008).

97 Zemach, A., McDaniel, I. E., Silva, P. & Zilberman, D. Genome-wide evolutionary analysis of eukaryotic DNA methylation. *Science* **328**, 916-919 (2010).

98 Lister, R. *et al.* Human DNA methylomes at base resolution show widespread epigenomic differences. *Nature* **462**, 315-322 (2009).

99 Zilberman, D., Gehring, M., Tran, R. K., Ballinger, T. & Henikoff, S. Genome-wide analysis of Arabidopsis thaliana DNA methylation uncovers an interdependence between methylation and transcription. *Nat Genet* **39**, 61-69 (2007).

100 Graveley, B. R. *et al.* The developmental transcriptome of Drosophila melanogaster. *Nature* **471**, 473-479 (2011).

101 Djebali, S. *et al.* Landscape of transcription in human cells. *Nature* **489**, 101-108 (2012).

102 Finn, R. D. *et al.* The Pfam protein families database. *Nucleic Acids Res* **38**, D211-222 (2010).

103 Eddy, S. R. Accelerated Profile HMM Searches. *PLoS Comput Biol* **7**, e1002195 (2011).

104 Punta, M. *et al.* The Pfam protein families database. *Nucleic Acids Res* **40**, D290-301 (2012).

105 Dosztanyi, Z., Csizmok, V., Tompa, P. & Simon, I. IUPred: web server for the prediction of intrinsically unstructured regions of proteins based on estimated energy content. *Bioinformatics* **21**, 3433-3434 (2005).

106 Gsponer, J. & Babu, M. M. The rules of disorder or why disorder rules. *Prog Biophys Mol Biol* **99**, 94-103 (2009).

107 Dyson, H. J. & Wright, P. E. Intrinsically unstructured proteins and their functions. *Nat Rev Mol Cell Biol* **6**, 197-208 (2005).

108 Huang, S. *et al.* Genomic analysis of the immune gene repertoire of amphioxus reveals extraordinary innate complexity and diversity. *Genome Res* **18**, 1112-1126 (2008).

109 Holland, L. Z. *et al.* The amphioxus genome illuminates vertebrate origins and cephalochordate biology. *Genome Res* **18**, 1100-1111 (2008).

110 Huang, S. *et al.* The evolution and regulation of the mucosal immune complexity in the Basal chordate amphioxus. *J Immunol* **186**, 2042-2055 (2011).

111 Roach, J. C. *et al.* The evolution of vertebrate Toll-like receptors. *Proc Natl Acad Sci U S A* **102**, 9577-9582 (2005).

112 Tamura, K., Dudley, J., Nei, M. & Kumar, S. MEGA4: Molecular Evolutionary Genetics Analysis (MEGA) software version 4.0. *Mol Biol Evol* **24**, 1596-1599 (2007).

113 Yang, Z. PAML 4: phylogenetic analysis by maximum likelihood. *Mol Biol Evol* **24**, 1586-1591 (2007).

114 Buljan, M., Frankish, A. & Bateman, A. Quantifying the mechanisms of domain gain in animal proteins. *Genome Biol* **11**, R74 (2010).

115 Pascual-Anaya, J., D'Aniello, S. & Garcia-Fernandez, J. Unexpectedly large number of conserved noncoding regions within the ancestral chordate Hox cluster. *Development genes and evolution* **218**, 591-597 (2008).

116 Hufton, A. L. *et al.* Deeply conserved chordate noncoding sequences preserve genome synteny but do not drive gene duplicate retention. *Genome Res* **19**, 2036-2051 (2009).
